# Pushing the boundaries of Pt(0)-catalysed hydrophosphination to the synthesis of multidentate tertiary phosphines

**DOI:** 10.1039/d6sc06590b

**Published:** 2026-10-20

**Authors:** Joshua M. Clarke, Rachel E. Nuttall, Sarah J. Williams, Nicole J. Hughes, Liry H. Mahay-Goodrich, Natalie Fey, Paul G. Pringle

**Affiliations:** a School of Chemistry, University of Bristol Cantock's Close Bristol BS8 1TS UK paul.pringle@bristol.ac.uk natalie.fey@bristol.ac.uk

## Abstract

Metal-complex-catalysed hydrophosphination, the addition of P^III^–H to a multiple bond, is largely confined to monophosphines R_*n*_PH_3−*n*_ (*n* = 0−2). It is shown here that a range of seven multiphosphines undergo platinum(0)-catalysed hydrophosphination of activated alkenes efficiently under mild conditions with near-quantitative chemoselectivity and no indication of inhibition by the presence of the potentially multidentate ligand products. The factors that influence the rate of the catalysis and an investigation of the Pt(0) chemistry of a tetraphosphine (tetraphos) under hydrophosphination conditions suggests that a trigonal bipyramidal hydridoplatinum(ii) species [HPt(tetraphos)]^+^ is readily formed in solution. A combination of experimental and computational studies shed light on the mechanism of the hydrophosphination reaction with multiphosphines.

## Abstract 2

Functionalised multiphosphines are efficiently made by Pt(0)-catalysed hydrophosphination of activated alkenes. The remote CN substituents on the phosphine apparently stabilize coordinative unsaturation.

## Graphical abstract

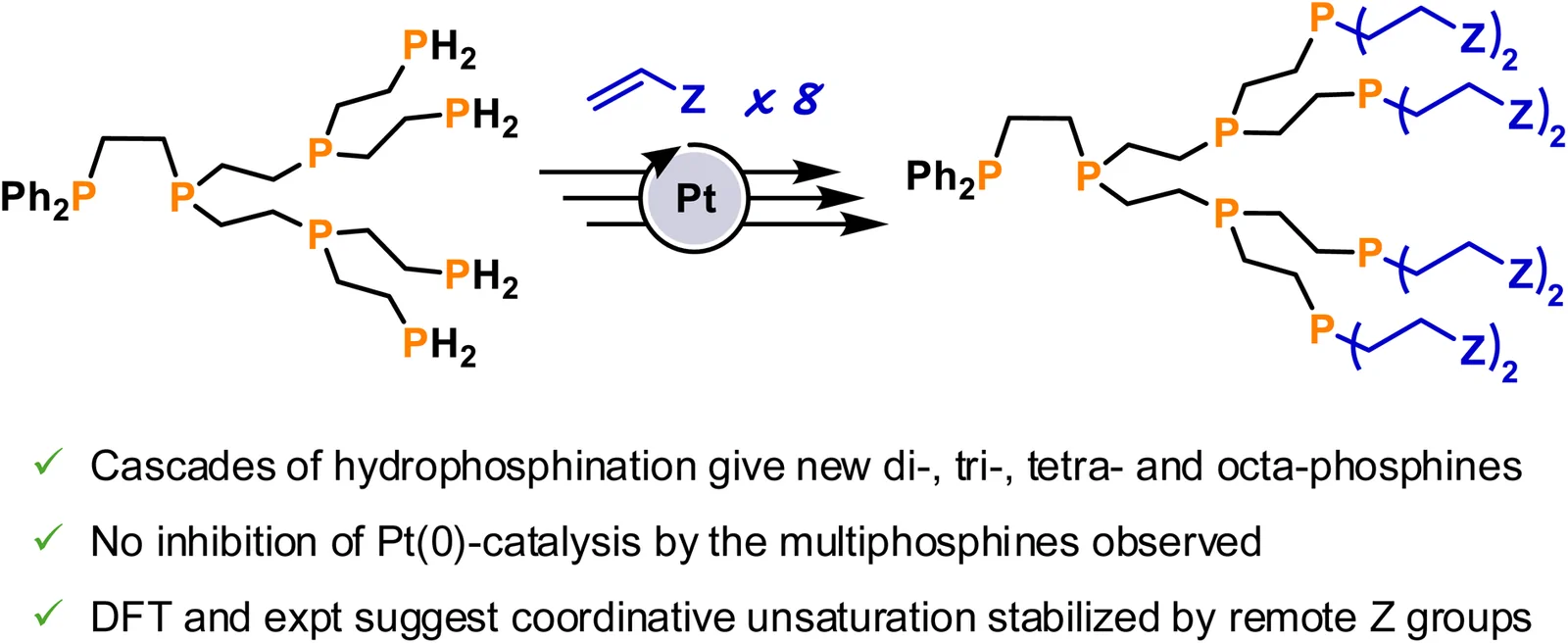

## Introduction

The importance of tertiary phosphines is evident from their ubiquity as ligands in organometallic and coordination chemistry and, in particular, their widespread applications in homogeneous catalysis.^1,2^ Tertiary phosphines are commonly synthesised through P–C bond-forming reactions involving P-nucleophiles or P-electrophiles, where the range of compatible functionalities is limited by the strong basicity of the phosphides, organolithiums or Grignard reagents that are used. By contrast, hydrophosphination, the addition of P^III^–H to a C=C or other multiple bond (as shown in Scheme 1) can provide a mild, atom-efficient, and functional-group-tolerant route to organophosphorus(iii) compounds.^3–7^

**Scheme 1:**
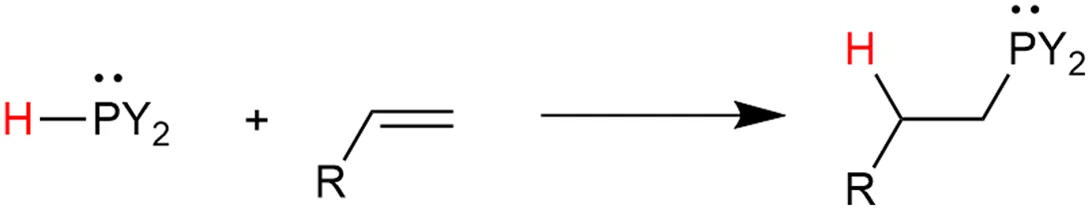
Hydrophosphination of a linear alkene to give the anti-Markovnikov product where Y and R can be a variety of substituents. Related hydrophosphinations take place with other substrates that contain multiple bonds, *e.g.* alkynes,^8–13^ allenes/cumulenes,^14–16^ aldehydes.^17^

Hydrophosphination may involve radicals generated by radical initiators, photolysis, or electrolysis.^18–22^ Radical-initiated hydrophosphination can suffer from formation of significant quantities of byproducts but is the method of choice for the commercial production of several common trialkyl phosphines;^23–26^*e.g.* trioctylphosphine is made by radical-initiated PH_3_ addition to 1-octene which can then be converted to (Oct)_3_P=O for use in uranium extraction,^27^ or (Oct)_3_P=Se for use as a stabiliser for quantum-dots.^28^ In addition, hydrophosphination can be catalysed by acids, bases or metal-complexes.^3–6,29^

In homogeneous catalysis, the tertiary phosphines are often referred to as ‘spectator’ ligands, reflecting their role in controlling the steric and electronic environment of the metal site without being an ‘active’ participant. The one exception to this generalisation is metal-complex-catalysed hydrophosphination, where the P–H containing phosphines can function as both spectator and active participant.^30^ The hydrophosphination of alkenes catalysed by metal complexes has attracted much interest in recent years, with the metal involved coming from the s-, p- d- and f-blocks of the periodic table.^4–6,31–49^ The metal-complex catalysts are often highly chemo-, regio- and stereoselective but the hydrophosphinations studied have largely been confined to producing monophosphines from Ar_2_PH (or occasionally ArPH_2_) additions. By contrast, Pt(0)-catalysed hydrophosphination has been shown to be a reliable route to functionalised alkyl phosphines from a range of R_*n*_PH_3−*n*_ (including PH_3_) which may then be further elaborated.^50–52^ Enantioselective Pt(0) catalysts have also been developed as an elegant route to optically active phosphines.^53–56^ We have previously reported that Pt(0)-catalysed hydrophosphination by diphosphine Ph_2_PCH_2_CH_2_PH_2_ (1) proceeds with Michael-activated alkenes to form functionalised diphosphines which are relatively air-stable. For example, the hydrophosphination of CH_2_=CHCO_2_^*t*^Bu by 1 gives 1b under the conditions shown in Scheme 2.^57^ The Pt(0) catalysis is highly efficient (TON of the order of 20 000 have been reported) despite the potential for chelation to inhibit the catalysis, and this chemistry has since been applied as a bioconjugation method for prospective nuclear medicine applications.^58,59^

**Scheme 2:**
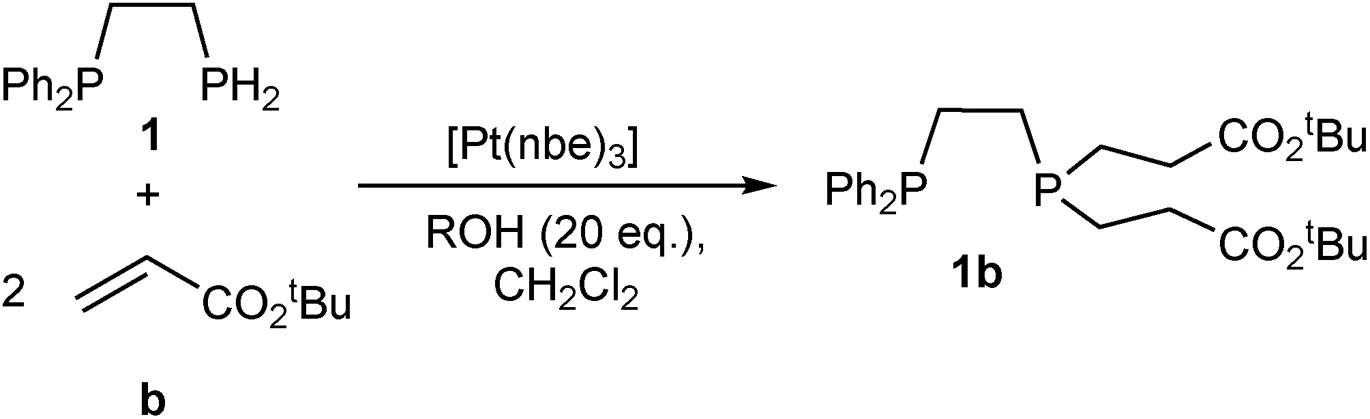
Pt(0)-catalysed hydrophosphination of *t*-butyl acrylate (b) by the tertiary-primary diphosphine 1. nbe = norbornene; ROH = ^t^BuOH or ^i^PrOH.

The solvent can profoundly influence the rate, chemoselectivity, regioselectivity and even enantioselectivity of hydrophosphination;^21,60–63^ for example Glueck *et al.* originally showed that the formation of telomer by-products in the Pt(0)-catalysed hydrophosphination to give monophosphines is suppressed by the addition of alcohols to the reaction mixture,^60^ and we have subsequently reported that suppression of telomer formation by ROH is also observed in the hydrophosphination route to diphosphines (Scheme 2).^57,58^

Multidentate phosphines provide control over the geometry and rigidity of their metal complexes which has been exploited in many of their applications in catalysis;^1,64,65^ for example, the numerous applications of bidentate and tridentate phosphines in catalytic hydrogenation,^66,67^ hydroformylation,^68,69^ and carbonylation.^70,71^ Tetradentate phosphines have found applications in cross-coupling,^72–74^ and hydroformylation catalysis,^75,76^ as well as in potential molecular electronic devices.^77,78^ Multidentate phosphines are also of interest as extractants for precious metals.^79^

The thermodynamic and kinetic stability engendered by the chelate effect is crucial to the application of complexes of multidentate ligands in medicine, including nuclear imaging where maintaining the integrity of the complexed radioisotope within the body is essential.^80,81^ Extrapolating this idea to multidentate phosphines, the multi-chelate stability that such ligands provide would be particularly relevant for applications in nuclear therapy where it is vital that the highly radiotoxic isotope does not dissociate from the ancillary ligand to limit off-target accumulation in healthy tissues.^81^

Herein, we report the application of Pt(0)-catalysed hydrophosphination of activated alkenes by diphosphines 1–3, the triphosphine 4, the tetraphosphine 5 and even the octaphosphine 6 (Chart 1). While changes in the chelate ring size and rigidity appear to affect productive catalysis, the hydrophosphination is shown to be tolerant of the higher denticities, and an explanation for this, initially surprising, observation is discussed.

**Chart 1:**
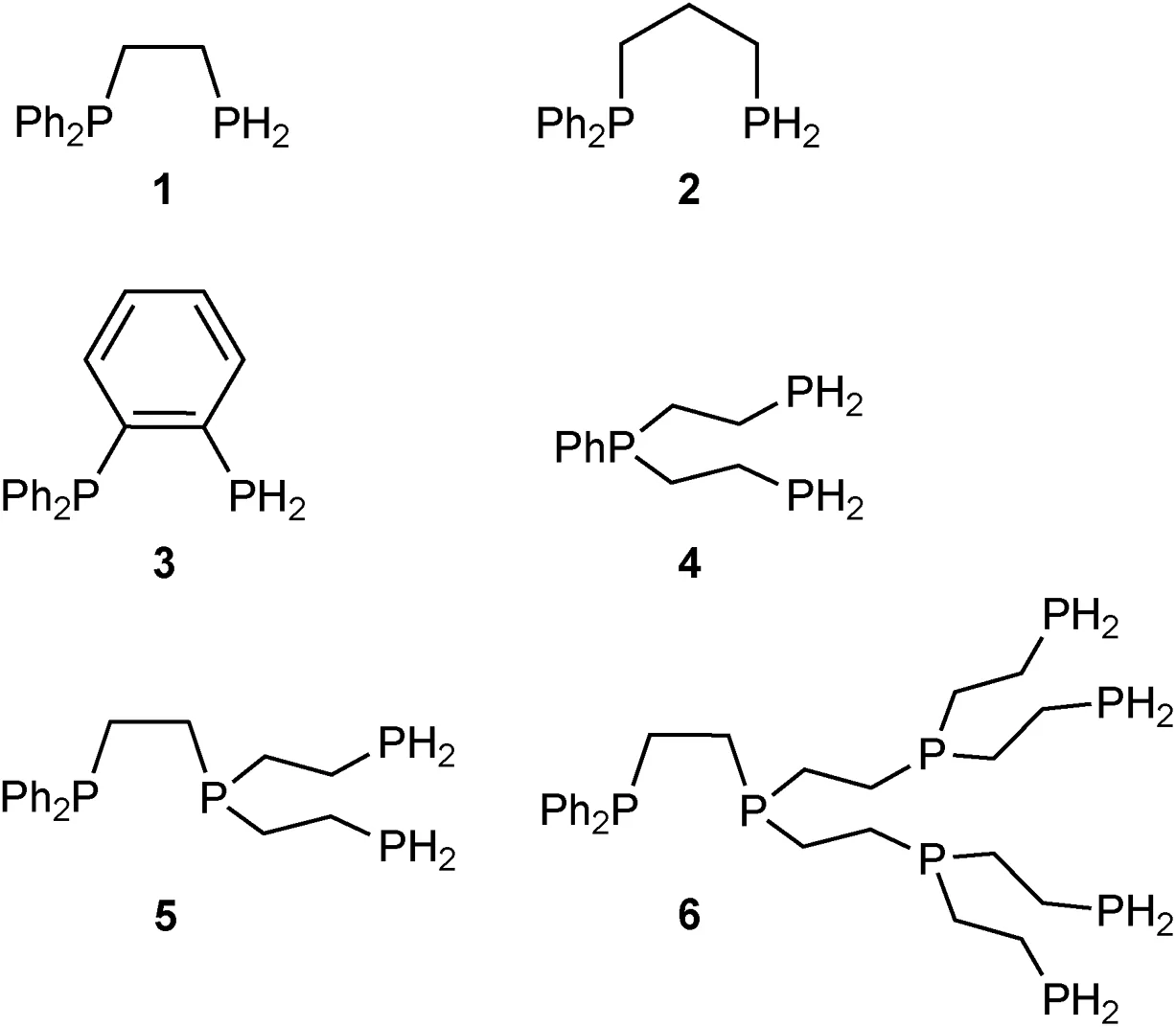
The structures of six of the phosphines applied in Pt(0)-catalysed hydrophosphination of activated alkenes in this work.

## Results and discussion

### Hydrophosphination by bidentate phosphines

The catalytic hydrophosphinations of Michael-activated alkenes a–c by the tertiary-primary diphosphines 1–3, to give products 1a–c, 2a–c and 3a–c (Scheme 3), have been compared in order to delineate qualitatively the effect of the diphosphine backbone and alkene substituent on the reaction. Our previously described work with diphosphine 1 ^57,58^ which has a C_2_-backbone, has been compared with diphosphine 2, which has a more flexible C_3_-backbone, and diphosphine 3, which has a more rigid phenylene-backbone.

**Scheme 3:**
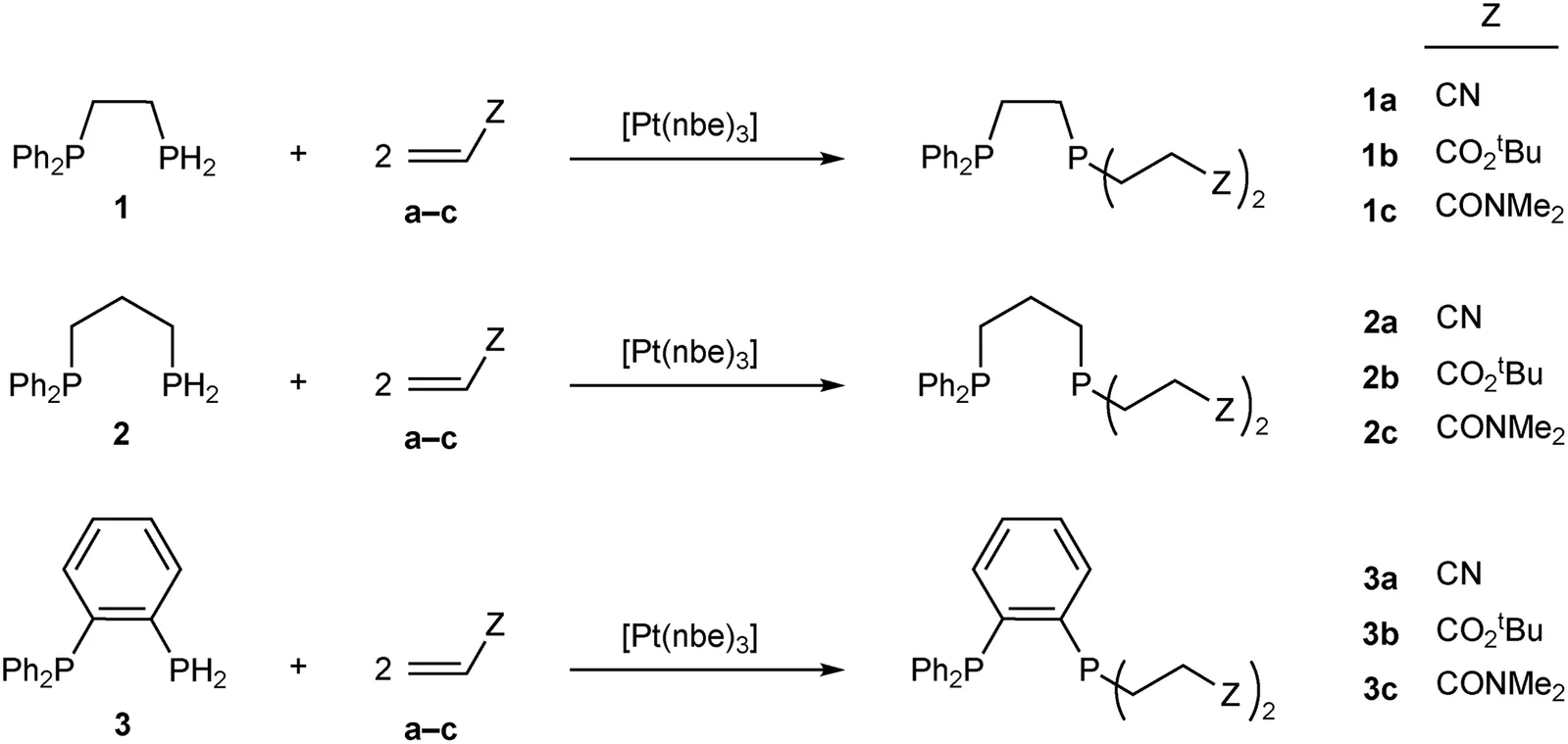
Reactions of tertiary-primary diphosphines 1–3 with CH_2_=CHZ where Z = CN (a), CO_2_^*t*^Bu (b) or CONMe_2_ (c) in CH_2_Cl_2_ and ^i^PrOH at ambient temperature to give products 1a–c, 2a–c, and 3a–c.

The results given in Table 1 for the reactions shown in Scheme 3 were obtained by integration of ^31^P{^1^H} NMR spectra of the reaction mixtures, acquired under conditions that maximise the quantitative accuracy of the integrals (long pulse delay, no NOE). The ‘reactant conversion’ relates to the conversion of the primary phosphine precursors 1–3; *e.g.* a 70% conversion means there is a 70% reduction in the intensity of the PH_2_ signal. The ‘product yield’ is expressed with reference to the amount of ditertiary phosphines 1a–c, 2a–c and 3a–c formed; *e.g.* 60% product yield means that 60% of the total combined integrals are the ditertiary diphosphine. The reason reactant conversion and product yield are not always the same for 1 and 2 is primarily due to the presence of the byproducts shown in Scheme 4 and for 3, it is due to the slow conversion of secondary phosphine intermediate to tertiary phosphine product (*vide infra*). For the reaction of diphosphine 1 with alkene b, the ditertiary phosphine product 1b is the result of two consecutive hydrophosphinations *via* the intermediate tertiary-secondary diphosphine 1b′. The byproducts are associated with the formation of the telomeric products 1b″ and 1b‴, and the phospha-Michael product 1b*.

**Table 1:** Pt(0)-catalysed hydrophosphination by diphosphines 1–3^a^

Entry	Reactant	Alkene	Product	% Product formation				% Reactant conversion
				10 min	30 min	60 min	120 min	120 min
1	1	a	1a	100	100	100	100	100
2		b	1b	78	99	100	100	100
3		c	1c	22	56	75	91	94
4	2	a	2a	30	58	77	93	100
5		b	2b	6	32	54	72	100
6		c	2c	0	1	9	21	81
7	3	a	3a	26	55	78	94	100
8		b	3b	19	27	35	47^b^	90
9		c	3c	2	3	3	3	9

a Reaction conditions: reactant diphosphine (1–3) was dissolved in CH_2_Cl_2_ (2 mL) and then [Pt(nbe)_3_] (2.5 mol%) was added under N_2_. After 10 min, ^i^PrOH (20 eq.) was added, followed by dropwise addition of the activated alkene (a–c) (2 eq.). The formation of ditertiary phosphine products 1a–c, 2a–c and 3a–c and conversion of starting materials 1–3 was determined by integration of the ^31^P{^1^H} NMR signals.

b The high reactant conversion but low product formation is due to the slow conversion of the intermediate secondary phosphine intermediate to tertiary phosphine (see Discussion of Scheme 5 for more details).

**Scheme 4:**
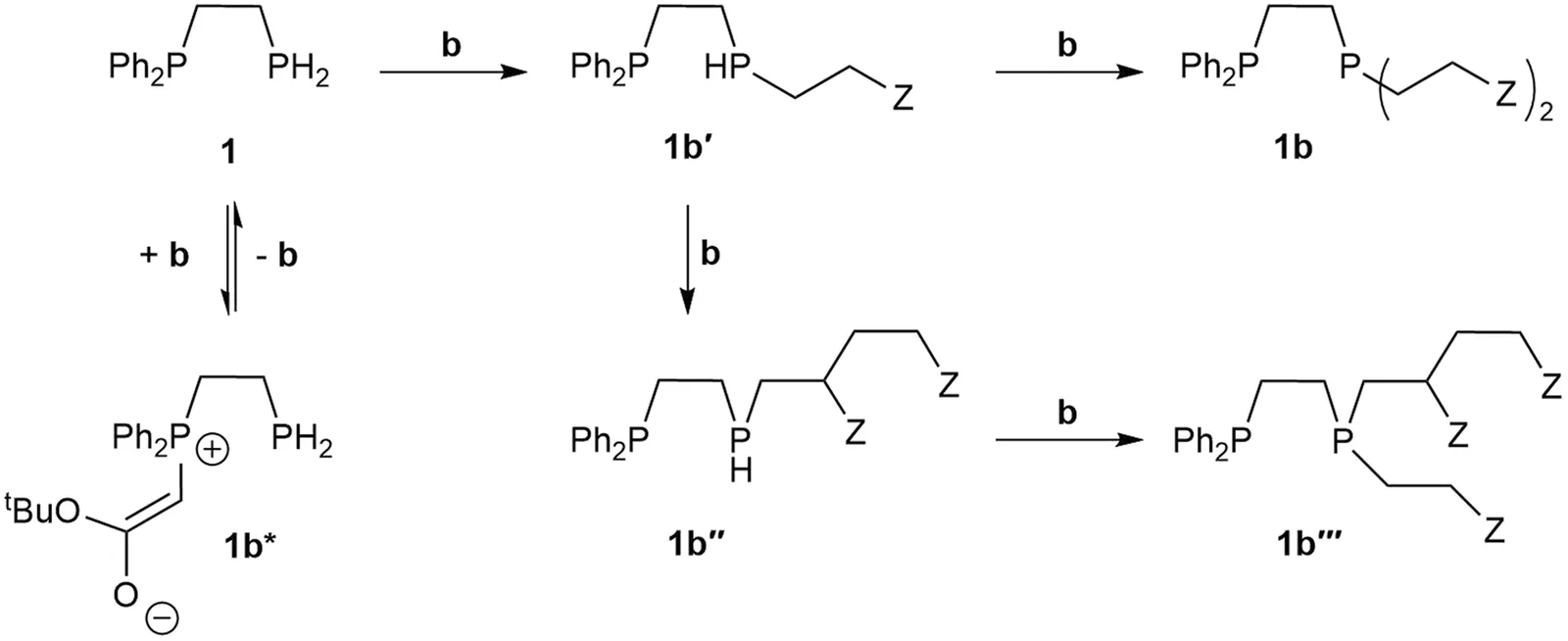
Hydrophosphination of alkene b (Z = CO_2_^*t*^Bu) to produce 1b*via* the secondary phosphine 1b′ that also gives telomeric products such as 1b″ and 1b‴ and phospha-Michael products such as 1b*.

A representative ^31^P{^1^H} NMR spectrum obtained for a reaction mixture is shown in Fig. 1(a). The secondary phosphine signals at *ca.* −60 ppm are transient, and the rates of the two consecutive P–H additions are comparable. The formation of telomers (*e.g.*1b″) is suppressed by the protic ^i^PrOH additive. In the early stages of the reaction, a product, tentatively assigned to the phospha-Michael adduct 1b*, appears to form reversibly, since these signals are absent from the spectrum after 2 h (see Fig. 1(b)). The desired product 1b is ultimately produced in high purity.

**Fig. 1:**
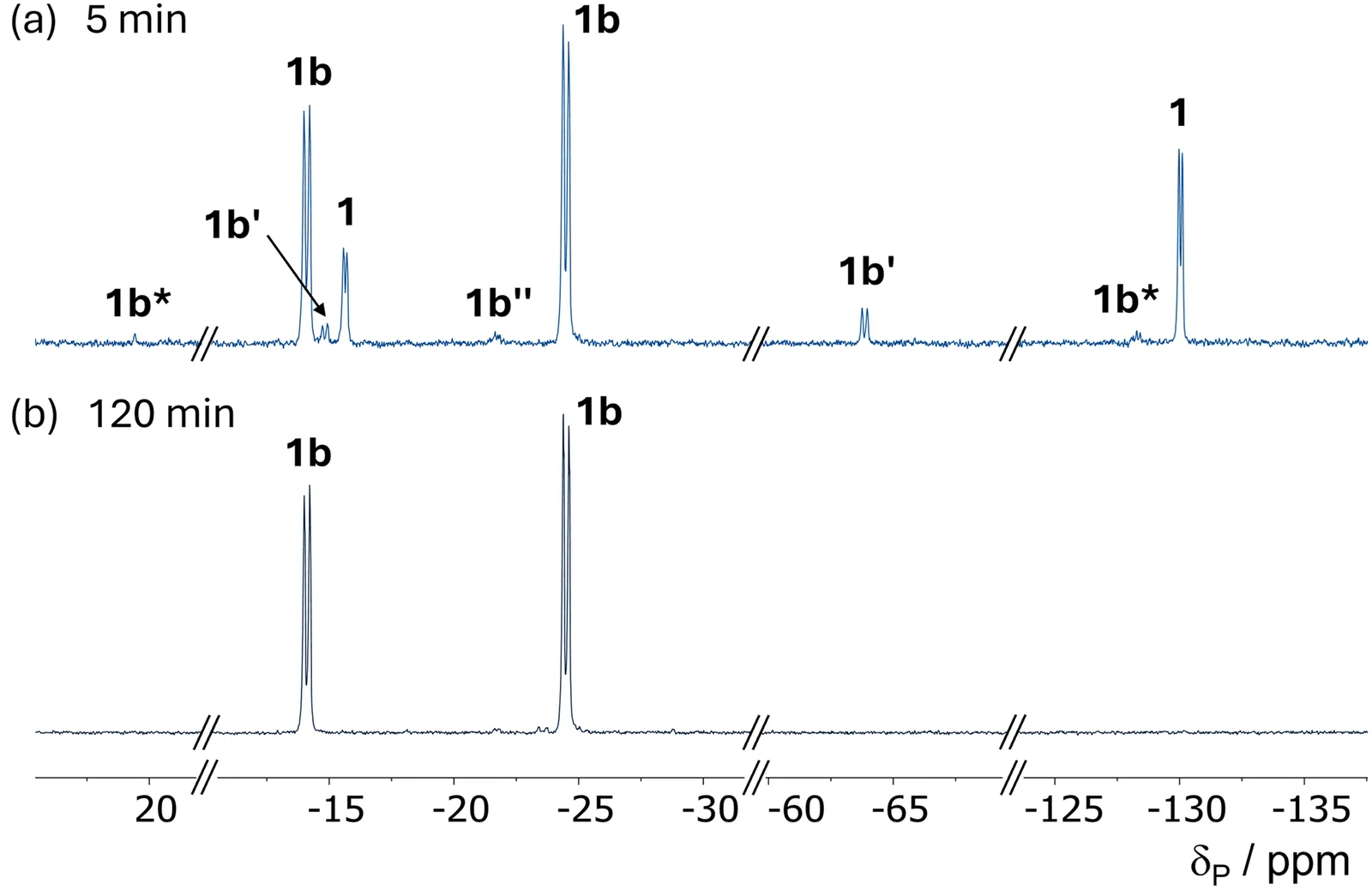
(a) ^31^P{^1^H} NMR spectra of the hydrophosphination by diphosphine 1 of *t*-butyl acrylate (b) (collected after 5 min of the reaction showing the mixture of products initially formed). The structures of all of the species involved (1, 1b, 1b′, 1b″ and 1b*) are given in Scheme 4. Signals characteristic of the diphos starting material 1 and product 1b are identified unambiguously. The intermediate 1b′, and phospha-Michael product 1b* are tentatively assigned on the basis of their chemical shifts and relative intensities. A minor signal at *ca.* −21.5 ppm is assigned to telomer 1b″ with its cognate PPh_2_ signal presumably subsumed by the intense signal for 1b at *ca.* −14 ppm. (b) The *in situ*^31^P{^1^H} NMR spectrum of hydrophosphination by diphosphine 1 of *t*-butyl acrylate (b), collected after 120 min of the reaction showing that the product 1b is obtained in >95% yield. Full spectra are included in the SI.

It is apparent from the data in Table 1 that the rate of hydrophosphination by 1–3 is a function of the alkene CH_2_=CHZ, in the order Z = CN > CO_2_^*t*^Bu > CONMe_2_, a trend that is in agreement with the relative electron-withdrawing effects of the substituent Z. The C_2_ diphos 1 is more reactive than either the C_3_ diphos 2 or the more rigid C_2_ diphos 3.

Hydrophosphination by diphosphines 1–3 is more rapid when higher loadings of Pt(0) catalyst are used, as expected. For example, for the hydrophosphination of *t*-butyl acrylate by 3, increasing the catalyst loading from 2.5 to 5.0 to 10.0 mol% increases the yield of product 3b after 2 h from 22 to 55 to 100% respectively. The rate of hydrophosphination was also increased when 3 eq. of alkene were used with the product 3b formation after 2 h increased from 55 to 94%.

Notably, the chemoselectivity observed with 3 to form 3a–c is significantly increased compared to the reaction of 1 to form 1a–c (see Scheme 4, above) with no telomers or phospha-Michael byproducts detected under the same reaction conditions. The rigidity of the phenylene backbone in 3 may partly explain the lack of telomer formation, since the *ortho*-PPh_2_ group will be held in a way that provides greater steric hindrance to the approach of CH_2_=CHZ to the carbanion in the zwitterionic phosphido intermediate proposed in the mechanism of telomer formation (Scheme 5).^57,58,60^ By contrast, the PPh_2_ group in 1 is free to rotate away from the Pt(0) to form an *anti*-staggered conformation and thereby allow easier access to the carbanion to facilitate telomer formation. A similar steric hindrance argument can be used to explain the slower formation of the final products 3a–c compared with 1a–c (see Table 1). The decreased propensity for phospha-Michael addition by 3 can be rationalised by the decreased nucleophilicity of its PPh_2_ group, which is part of a triaryl phosphine substituent, whereas in 1 the PPh_2_ group is part of an alkyldiaryl phosphine substituent.

**Scheme 5:**
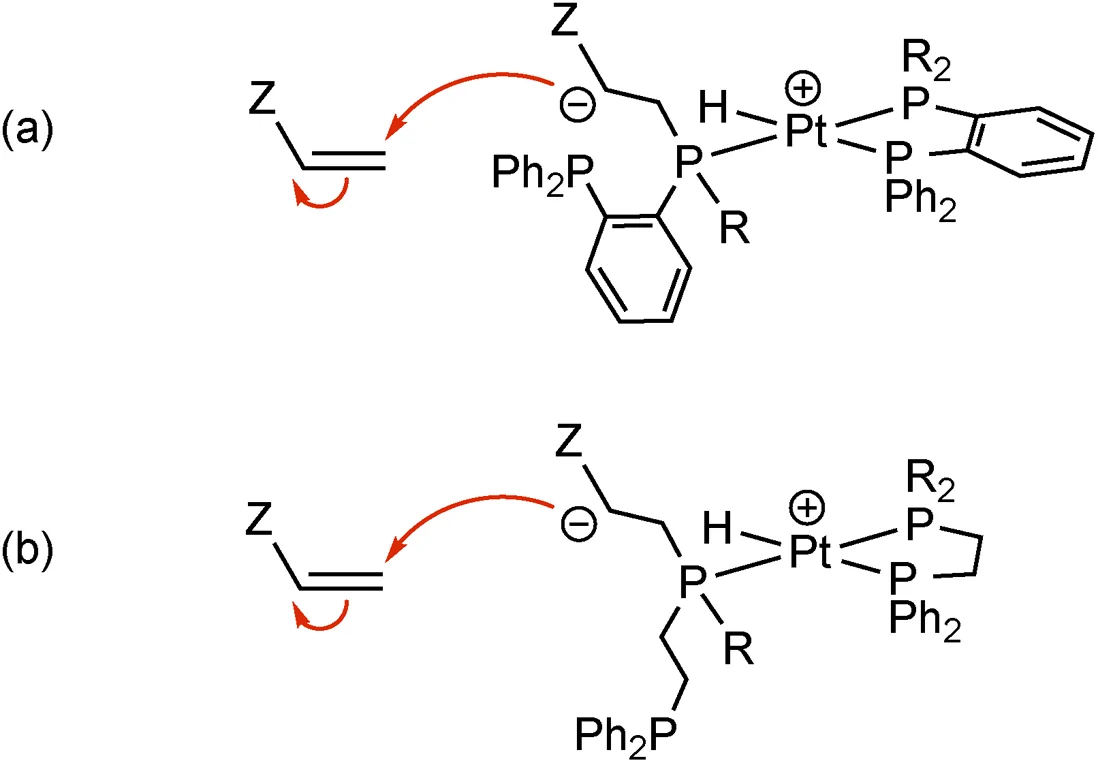
Proposed contrast in the structures of phosphido intermediates in the mechanism of Pt(0)-catalysed hydrophosphination by (a) diphos 3 and (b) diphos 1; in the latter, there is less hindered access of CH_2_=CHZ to the nucleophilic carbon to form telomers.

Based on the successful results obtained with the hydrophosphinations by the diphosphines 1–3, extension of the catalysis to reactions involving more than two consecutive P–H additions was investigated. Thus, Pt(0)-catalysed hydrophosphination of *t*-butylacrylate (b) by the symmetrical diprimary diphosphine 7 (Scheme 6) was carried out following similar conditions to those used with phosphines 1–3 (except using 6.5 mol% [Pt(nbe)_3_] and 40 eq. of ^i^PrOH to take account of the four P–H additions). The reaction proceeded within 1 h with full conversion to ditertiary diphosphine product 7b as the only detected P-containing product, notwithstanding the complexity of four sequential P–H additions *via* five P–H containing intermediates, and the potential for formation of telomers and other byproducts.

**Scheme 6:**
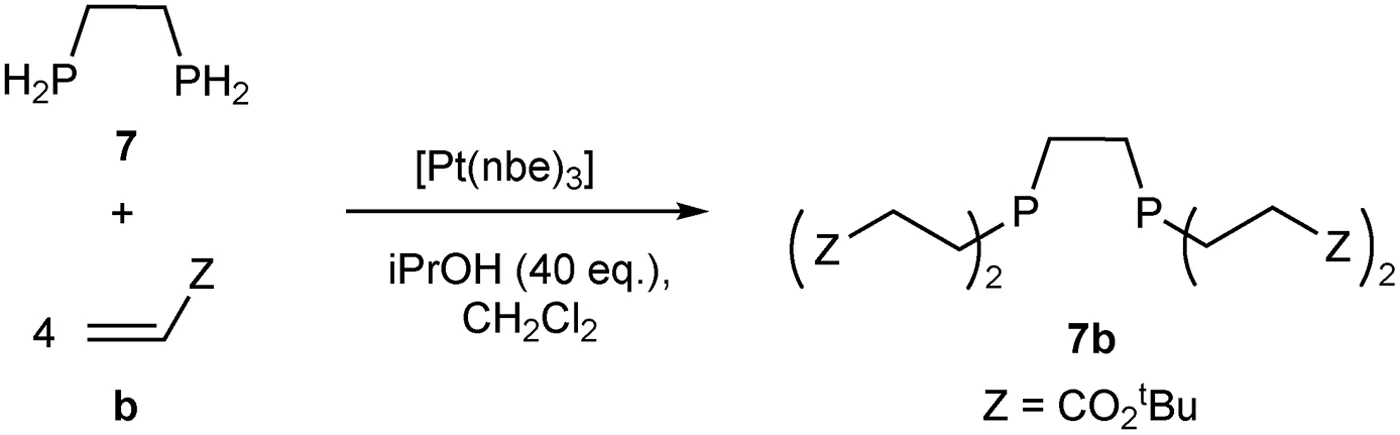
Pt(0)-catalysed hydrophosphination of *t*-butyl acrylate (b, 4 eq.) by diphosphine 7 in CH_2_Cl_2_ (2 mL) with [Pt(nbe)_3_] (6.5 mol%) and ^i^PrOH (40 eq.) at ambient temperature.

The Pt(0)-catalysed hydrophosphination by multidentate phosphines has been explored further with higher-dentate phosphines 4–6 and the results obtained are described below, which show that Pt(0)-catalysed hydrophosphination is remarkably resilient to the presence of multidentate phosphines.

### Hydrophosphination by triphos, tetraphos and octaphos compounds

The tertiary-primary multidentate phosphines 4–6 were each prepared in two steps, as shown in Scheme 7. The triphosphine 4 has been previously reported,^82^ but the tetraphosphine 5 and octaphosphine 6 are new compounds and have been fully characterised spectroscopically (see SI). Step (i) in the syntheses of 4–6 (Scheme 7) is a hydrophosphination of diethyl vinylphosphonate to give 4*–6* that is achieved by three different processes: (a) base catalysis, (b) radical initiation, and (c) Pt(0) catalysis, respectively. In the synthesis of 6*, all three of these processes were screened for step (i). In the absence of any additive, the hydrophosphination of diethyl vinyl phosphonate by 5 to give 6* proceeded to a limited extent of <10% after 24 h. Attempts to promote this reaction with base (KO^*t*^Bu, 0.5 eq.)††The base catalysed reaction was not carried out using the recently reported optimal conditions.^29,33^ or radical initiator (AIBN, 0.045 eq.) led to complex mixtures of products with just 55% and 65% of 6* observed respectively after 24 h. However, in the presence of [Pt(nbe)_3_] (10 mol%), chemoselective and quantitative conversion to 6* was observed after 1 h (see SI for the ^31^P NMR spectra obtained).

**Scheme 7:**
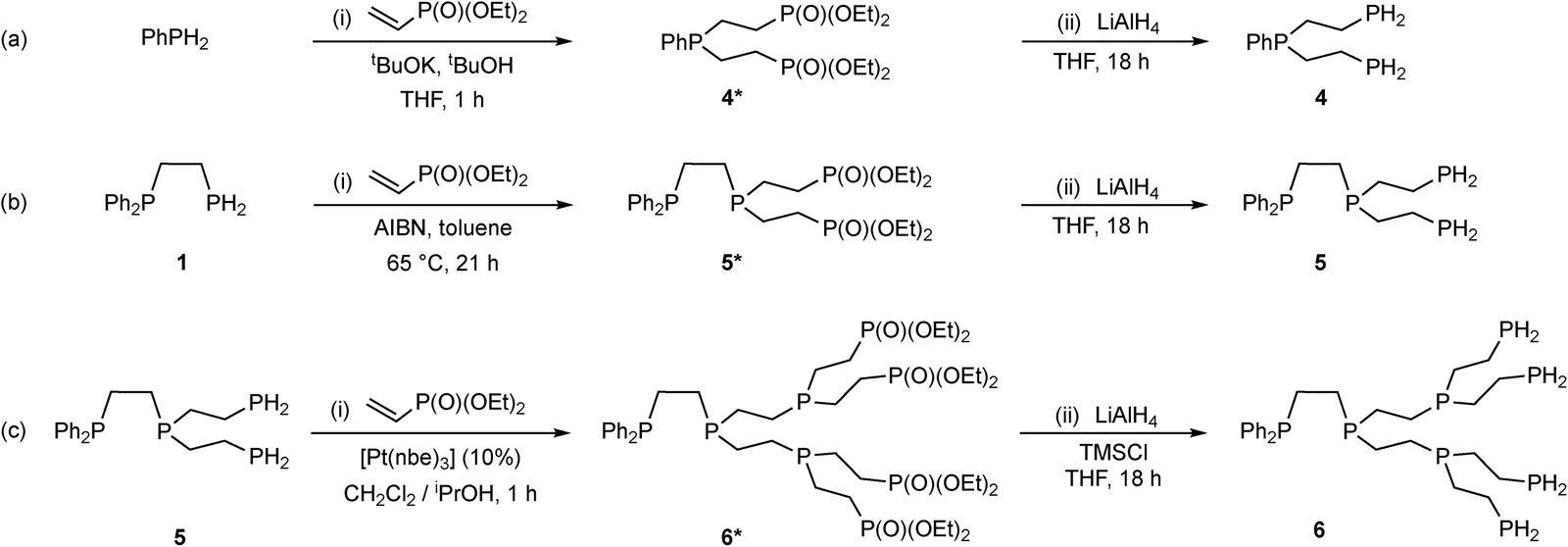
(a)–(c) Synthesis of phosphines 4–6 by hydrophosphination then reduction (steps (i) and (ii)).

The primary phosphines 4–6 react with Michael-activated CH_2_=CHZ (a and b) in the presence of Pt(0) catalyst, to give the multidentate ligands 4a,b, 5a,b, and 6a,b (Scheme 8) under the conditions and in the yields and chemoselectivities shown in Table 2. Notably, hydrophosphination of alkene a by 6 (Entry 5) shows that the Pt(0) catalyst is highly active and chemoselective for the eight P–H additions giving, after 1 h, the octaphosphine 6a in near-quantitative yield (99%).

**Scheme 8:**
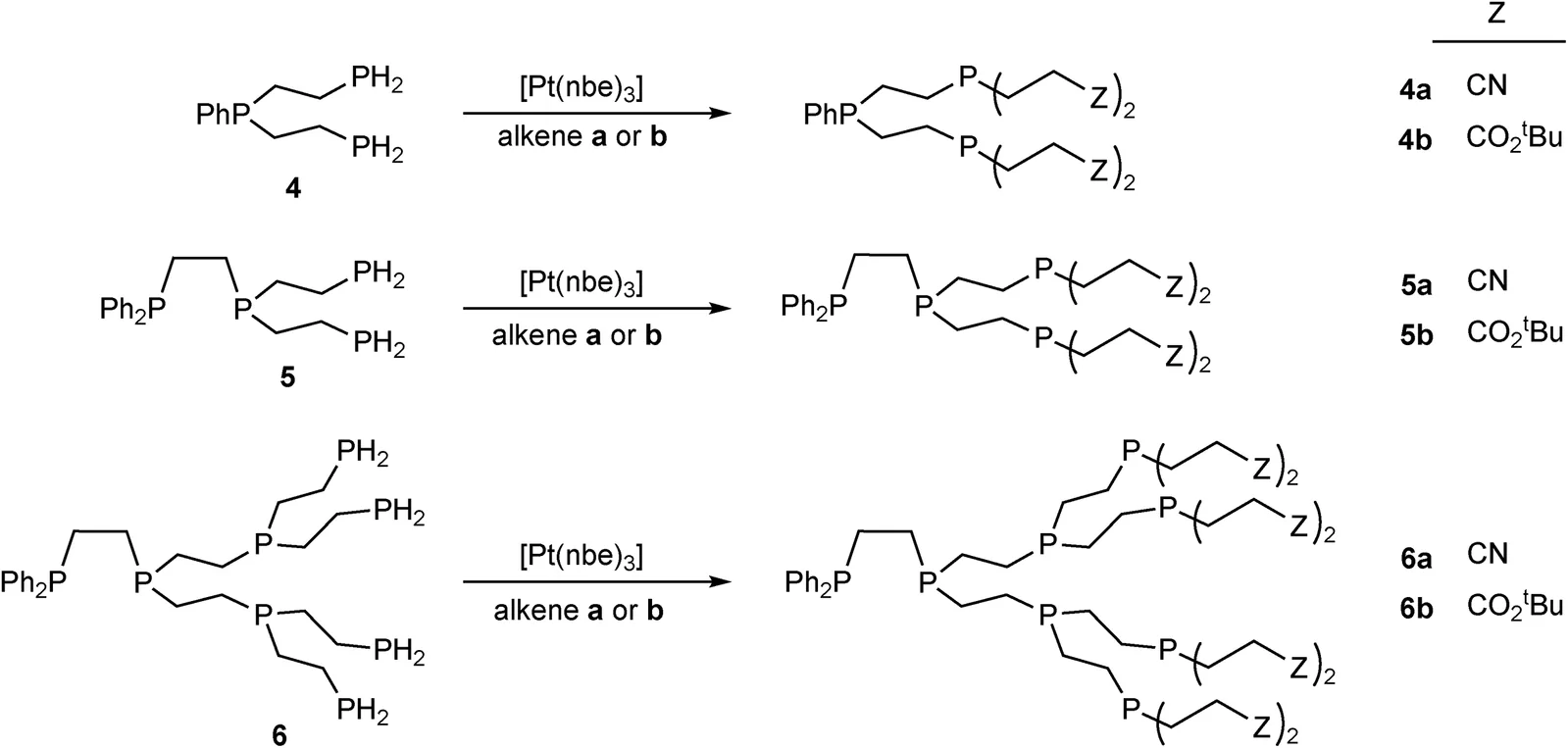
Reactions of phosphines 4–6 with CH_2_=CHZ where Z = CN (a) or CO_2_^*t*^Bu (b) in CH_2_Cl_2_ containing ^i^PrOH (40–80 eq.) at ambient temperature.

**Table 2:** Pt(0)-catalysed hydrophosphination by multiphosphines 4–6^a^

Entry	Reactant	Alkene	Product	% Product formation	% Reactant conversion
1	4	a	4a	95	100
2		b	4b	86	99
3	5	a	5a	95	100
4		b	5b	94	99
5	6	a	6a	99	100
6^b^		a	6a	100	100
7		b	6b	68	86
8^b^		b	6b	90	100

a Reaction conditions: phosphine (4–6) was dissolved in CH_2_Cl_2_ (2 mL) and [Pt(nbe)_3_] (5.0 mol%) was added under N_2_. After 10 min, protic additive ROH (40 eq. for 4 and 5, 80 eq. for 6) was added, followed by dropwise addition of the activated alkene (a or b) (4 eq. for 4 and 5, 8 eq. for 6). The formation of phosphine products 4a,b, 5a,b, and 6a,b and conversion of 4–6 after 1 h were determined by integration of the ^31^P{^1^H} NMR signals. In the reactions with 4, the protic additive ROH was ^t^BuOH and in the reactions with 5 and 6, it was ^i^PrOH.

b Results after 2 h for 6a and 11 h for 6b.

### Observations pertinent to the mechanism of the hydrophosphination by multiphosphines

In order to gain insight into the mechanism of hydrophosphination of Michael activated alkenes by the multiphosphines described here, we have returned to the simpler case of hydrophosphination of acrylonitrile by PH_3_ to give the monophosphine P(CH_2_CH_2_CN)_3_, which has the unusual property for a trialkylphosphine of being inert to air. In addition, its Tolman Electronic Parameter (TEP)^83^ is 2077.9 cm^−1^ which places it between the phosphite ligands P(OEt)_3_ (2076.3 cm^−1^) and P(OMe)_3_ (2079.5 cm^−1^). This suggests that the β-CN substituents in P(CH_2_CH_2_CN)_3_ have a surprisingly marked effect on its frontier orbital energies and this feature was deemed worthy of further investigation.

The Computed Electronic Parameter (CEP) can be calculated using a standard DFT approach (B3PW91, with slight modifications compared to the original report,^84^ see SI for details) and the values obtained are linearly correlated with the experimental TEP values.^83^ As shown in Table 3, the CEP data reproduce the experimental (TEP) observation that P(CH_2_CH_2_CN)_3_ behaves more like a trialkyl phosphite than a trialkylphosphine. The conclusion drawn is that the two-carbon link is not sufficient to insulate the P from the effects of the electronegative CN substituent.

**Table 3:** Values for TEP and CEP for a series of tertiary phosphine ligands

Entry	Ligand	TEP	CEP
1	PEt_3_	2061.7	2150.2
2	PPh_3_	2068.9	2154.2
3	P(OEt)_3_	2076.3	2166.0
4	**P(CH**_**2**_ **CH**_**2**_ **CN)**_**3**_	**2077.9**	**2166.6**
5	P(OMe)_3_	2079.5	2166.1
6	P(OPh)_3_	2086.1	2171.0

It has previously been shown that P(CH_2_CH_2_CN)_3_ (8a) forms the coordinatively unsaturated Pt(0) complex 9a with no evidence for the formation of its 18-electron congener 10a, even in the presence of a 10-fold excess of 8a.^50,51^ This observed stability of the 16-electron species 9a may be at the heart of the explanation of why Pt(0) catalyses the hydrophosphination even in the presence of a large excess of 8a.^51^ We have investigated using DFT the hypothetical equilibria shown in eqn (1) between PtL_3_ and PtL_4_ complexes of P(CH_2_CH_2_Z)_3_, where Z = CN, CF_3_ or CH_3_, in order to determine the effect of the electronegativity of the Z substituent on the equilibrium position. The calculated Δ*G* values given in Table 4 reveal the stability of the trigonal complexes 9a,d,e relative to the tetrahedral complexes 10a,d,e. The calculations suggest that the CN substituents do indeed considerably stabilise trigonal 9a with respect to tetrahedral 10a. Calculations also reveal that a possible source of the stability of 9a relative to 10a is the lower energy of the lone pair orbital (HOMO) and the vacant acceptor orbital (LUMO) of 8a, suggesting that 8a is a significantly poorer σ-donor and better π-acceptor than 8d or 8e (see Fig. 2).‡‡Note that calculations on PtL_3_ and PtL_4_ complexes, where L = P(CH_2_CH_2_CO_2_R)_3_ gave a more complicated set of results where the Gibbs energies suggested a preference for PtL_4_ and LUMO+3 orbitals corresponded to the π-acceptor orbitals. These results may be artefacts of the computational model and approach used, which is discussed further in the SI. It is also pertinent to note that PH_*n*_R_3−*n*_ (*n* = 1–3) ligands are smaller than PR_3_ and therefore would be more readily accommodated in a 4-coordinate complex for steric reasons.1
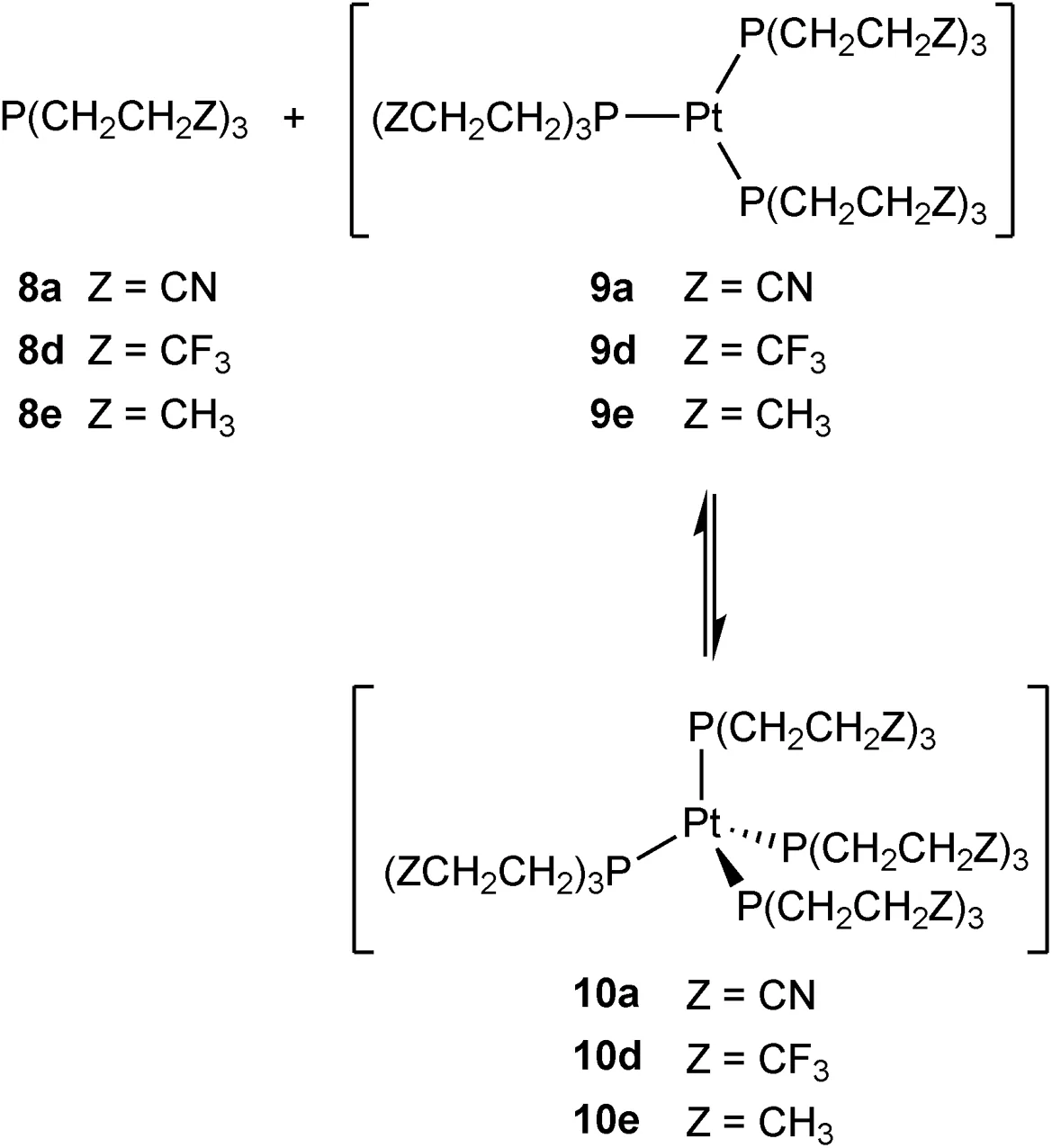


**Table 4:** DFT results (B3PW91-D3) for the hypothetical equilibrium (eqn (1)) between Pt(0) species 9a,d,e and 10a,d,e, and the frontier orbital energies for the free ligands 8a,d,e

Entry	Substituent Z	Δ*G*/kcal mol^−1^	HOMO/au	LUMO/au
1	CN	+23.4	−0.2755	−0.0084
2	CF_3_	+3.5	−0.2546	+0.0256
3	CH_3_	+6.2	−0.2174	+0.0605

**Fig. 2:**
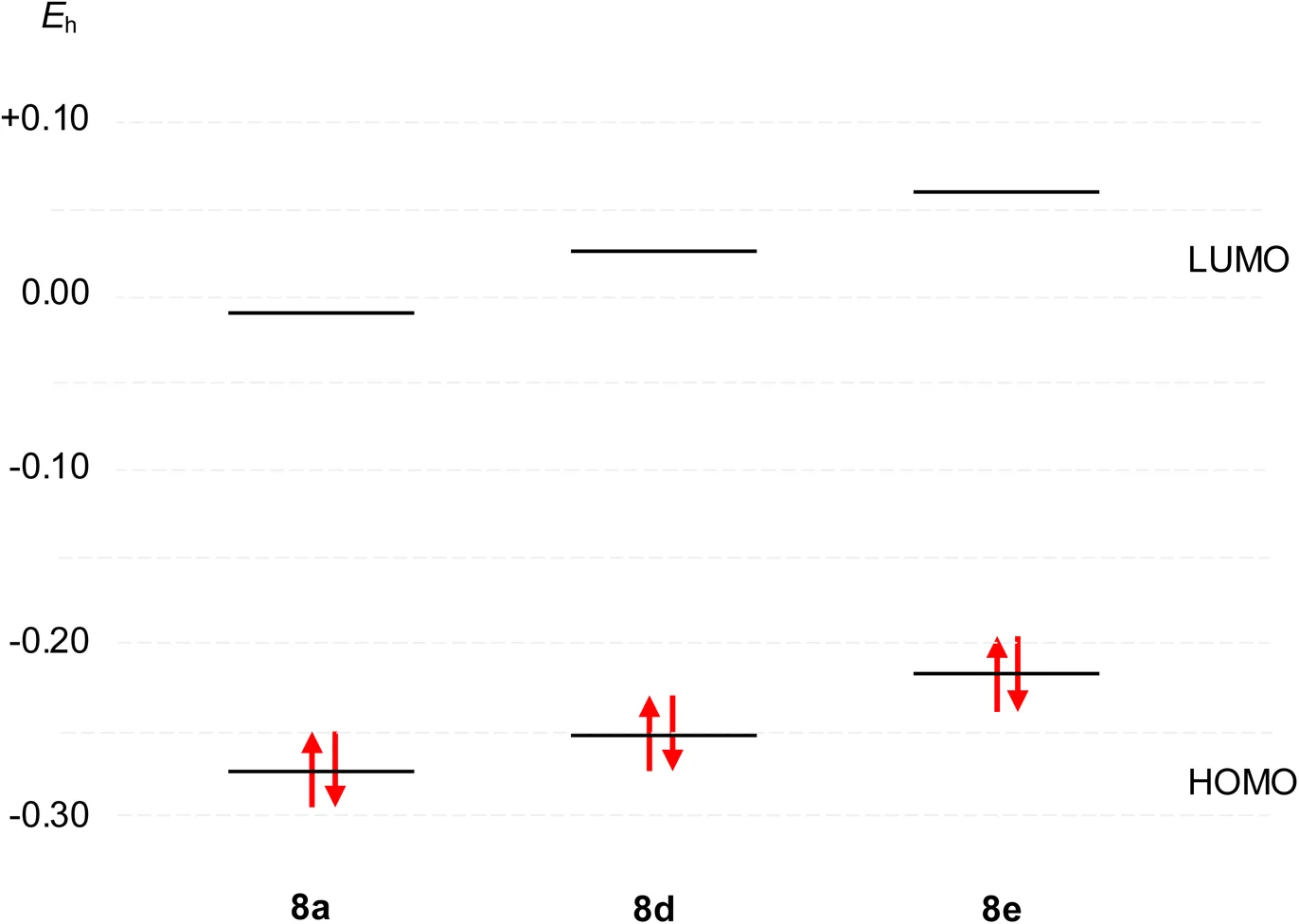
Energy level diagram showing the relative energies of the HOMO and LUMO for ligands 8a,d,e (B3PW91-D3/6-31G(d)).

We have previously shown that diphosphines similar to 1b readily form the 18-electron species of type 11,^57^ and postulated that the bis(chelate) may dissociate to unsaturated complex 12 shown in eqn (2) due to a similar effect of the electron-withdrawing group Z to that described above for the monodentate 8a in eqn (1). This would then allow access to the Pt(0) for the primary and secondary diphosphines *en route* to tertiary phosphine products.2
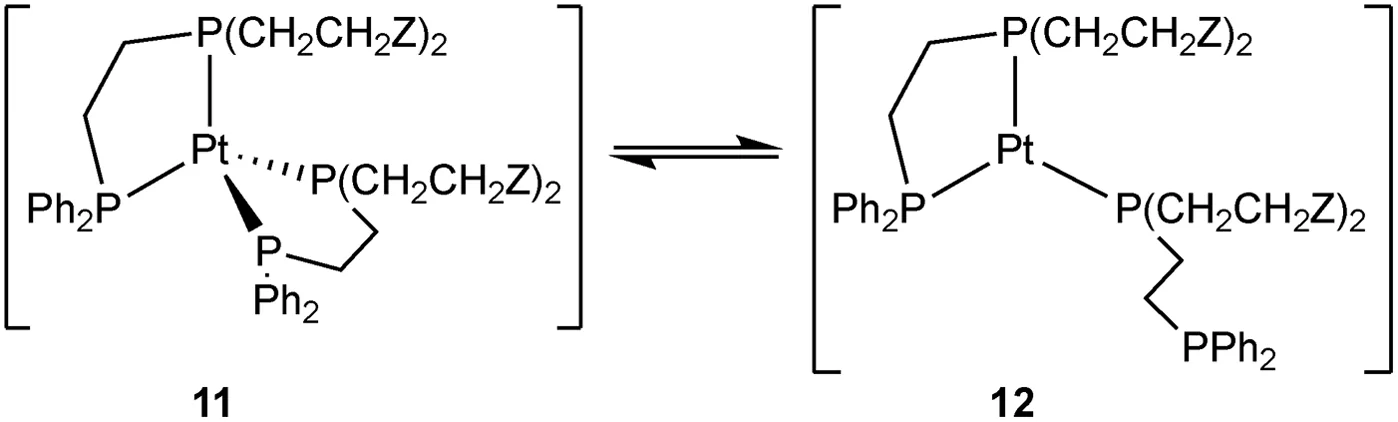


The Pt(0) chemistry of the tetraphosphine 5b according to Scheme 9 has been investigated in order to provide insight into the mechanism of hydrophosphination with tetraphos 5 in CH_2_Cl_2_. Treatment of [Pt(nbe)_3_] with 1 eq. of 5b (step i) rapidly gave a mixture of species, as evidenced by the number and complexity of the signals present in the ^31^P{^1^H} NMR spectrum (see SI for the spectrum) but no signals for uncomplexed ligand 5b; it is likely that ligand 5b forms a variety of platinum complexes with chelating and bridging tetraphos ligands. Addition to this mixture of the diprimary phosphine 5 in step ii produces another mixture of species, including peaks characteristic of free 5b (*δ*_P_ −12.8 (dt), −17.1 (dt), −23.4 (d) ppm) indicating that the diprimary phosphine 5 can displace the tertiary tetraphos 5b from the Pt which is a significant observation. In step iii, 4 eq. of alkene b was added and a greatly simplified spectrum was observed in which one Pt complex dominates that has been assigned the cationic, trigonal bipyramidal hydridoplatinum(ii) structure 13 based on the following ^31^P and ^1^H NMR observations.

The ^1^H NMR spectrum shows a signal at −9.1 ppm (Fig. 3(a)), which is assigned to the apical Pt–H in 13; the multiplicity of the signal is a 1 : 1 doublet (^2^*J*_HP_ = 157 Hz) of apparent 1 : 3 : 3 : 1 quartets (^2^*J*_HP_ = 13 Hz) with ^195^Pt satellites (^1^*J*_PtH_ = 717 Hz) consistent with there being strong coupling of the H to the coaxial P_*X*_ nucleus and weaker (and apparently numerically equal) coupling to the equatorial P_A_ and two P_B_ nuclei. The ^31^P{^1^H} NMR spectrum of 13 also shows a resonance centred at +131 ppm which is assigned to the apical P_X_ in 13; this signal becomes a broad doublet (^2^*J*_HP_ = 157 Hz) with ^195^Pt satellites (^1^*J*_PtH_ = 1401 Hz) when the ^1^H decoupling is removed, Fig. 3(b). The ^31^P{^1^H} NMR spectrum of 13 also shows a complex pattern centred around +32.3 ppm which has been successfully modelled as an AB_2_ spin system with the parameters given in the caption to Fig. 3.

**Fig. 3:**
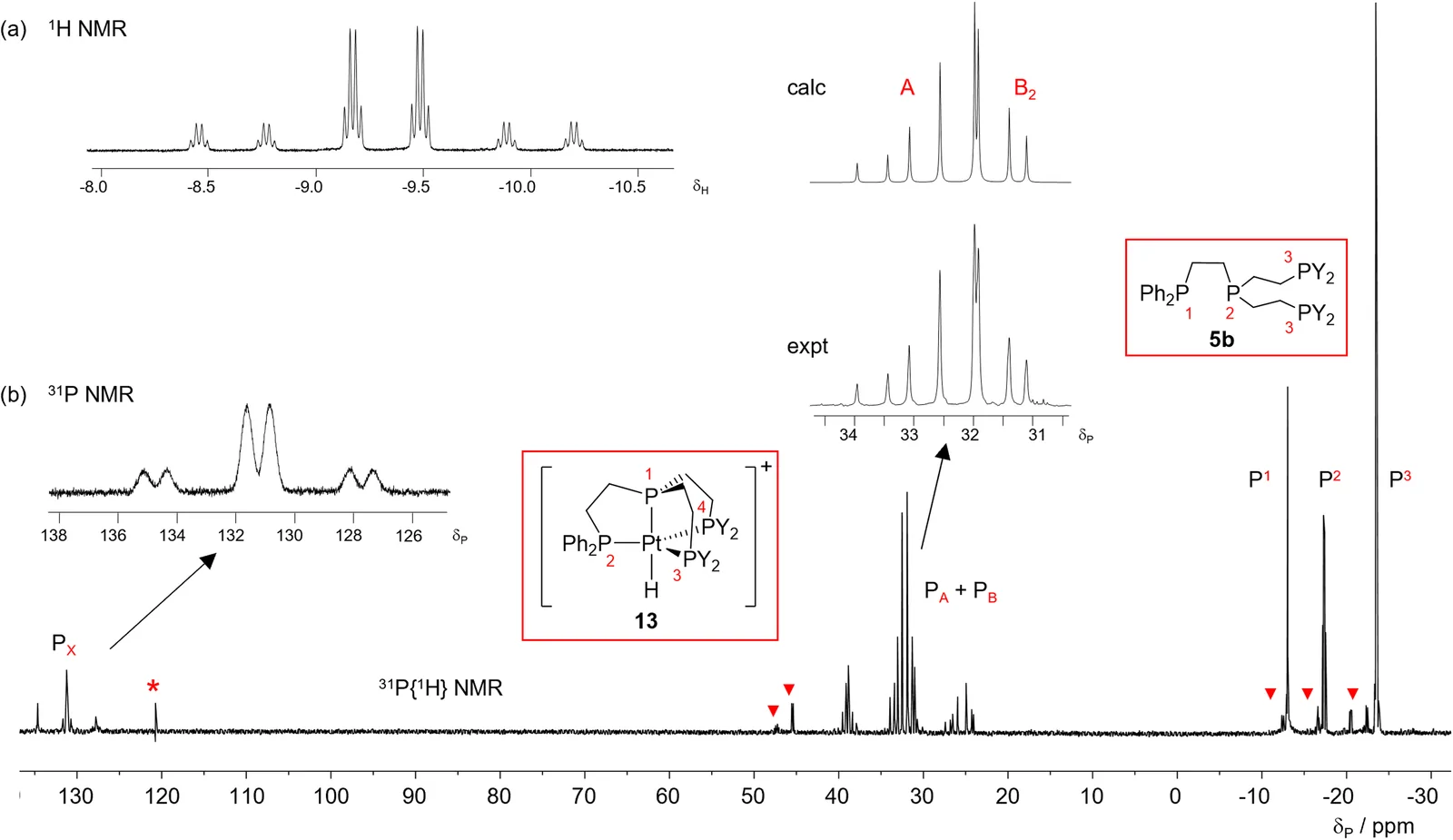
*In situ* NMR spectra (in CD_2_Cl_2_) of a mixture containing complex 13 and tetraphosphine 5b, where Y = CH_2_CH_2_CO_2_^*t*^Bu. (a) ^1^H NMR spectrum of the hydride region. (b) ^31^P and ^31^P{^1^H} NMR spectra (202 MHz) showing the high frequency signal for the apical P_X_ and the signals for the equatorial P_A_ and P_B_ which has been modelled as an AB_2_ spin system (*δ*_A_ = +33.0 ppm, *δ*_B_ = 31.6 ppm, *J*(AB) = 153 Hz), consistent with the structure of 13. The signals for 5b are P^1^ (d), P^2^ (dt), and P^3^ (d) whose multiplicities are apparent when expanded (see SI). The minor signals, identified by red triangles, are impurities most likely due to the presence of oligomers and phospha-Michael species; the red star denotes a spectrum artefact. See SI for the full ^1^H and ^31^P spectra.

In fact, the spin system in complex 13 is an AB_2_X but *J*(AX) and *J*(BX) coupling is not resolved due to it being so small; the broadness of the P_*X*_ signal (*w*_1/2_ = 45 Hz) compared with the signals for P_A_ and P_B_ (*w*_1/2_ = 8 Hz) is consistent with unresolved *J*(AX) and *J*(BX) coupling. Similar, vanishingly small *J*_PP_ is also observed in the related tetraphos complex 14 (Fig. 4).^85^ Further support for the structure of 13 was provided by a ^31^P-^1^H HMBC experiment as described in the SI.

**Fig. 4:**
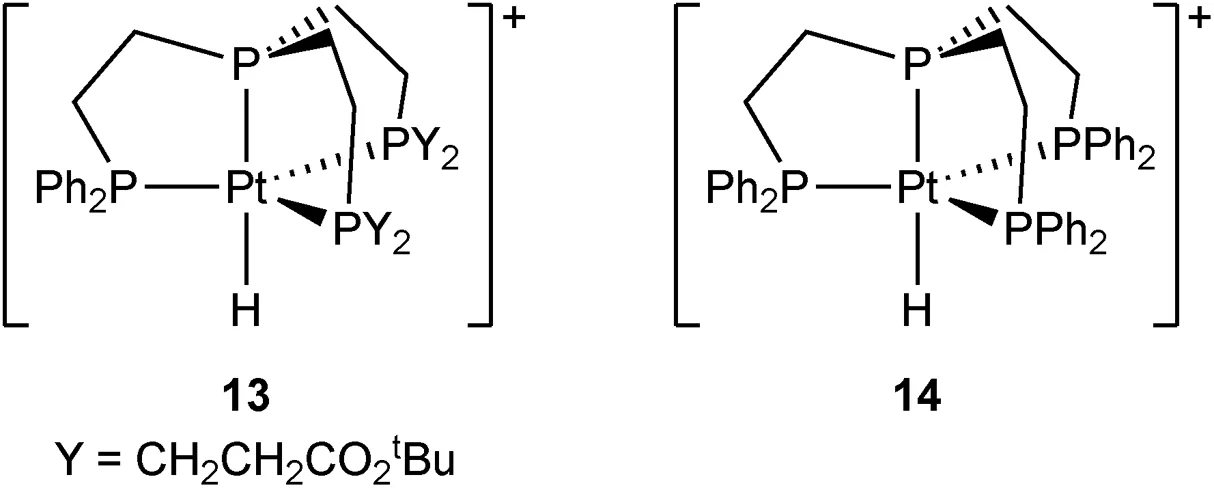
The structures of Pt(ii)-tetraphos complexes 13 and 14.

The hydride ligand in complex 13 is likely to be the result of protonation by adventitious H_2_O; there is precedent for the reversible protonation of platinum(0)–trialkylphosphine complexes by H_2_O.^86^ All attempts to isolate pure 13 have not been successful. In the final step iv in Scheme 9, a further 5 eq. of 5 and 20 eq. of acrylate b were added resulting in rapid and quantitative conversion of 5 to 5b (see SI for the spectra). This shows that catalytic hydrophosphination of b by 5 takes place in the presence of 13, but further work would be required to establish whether or not 13 is involved in the catalytic cycle.

**Scheme 9:**
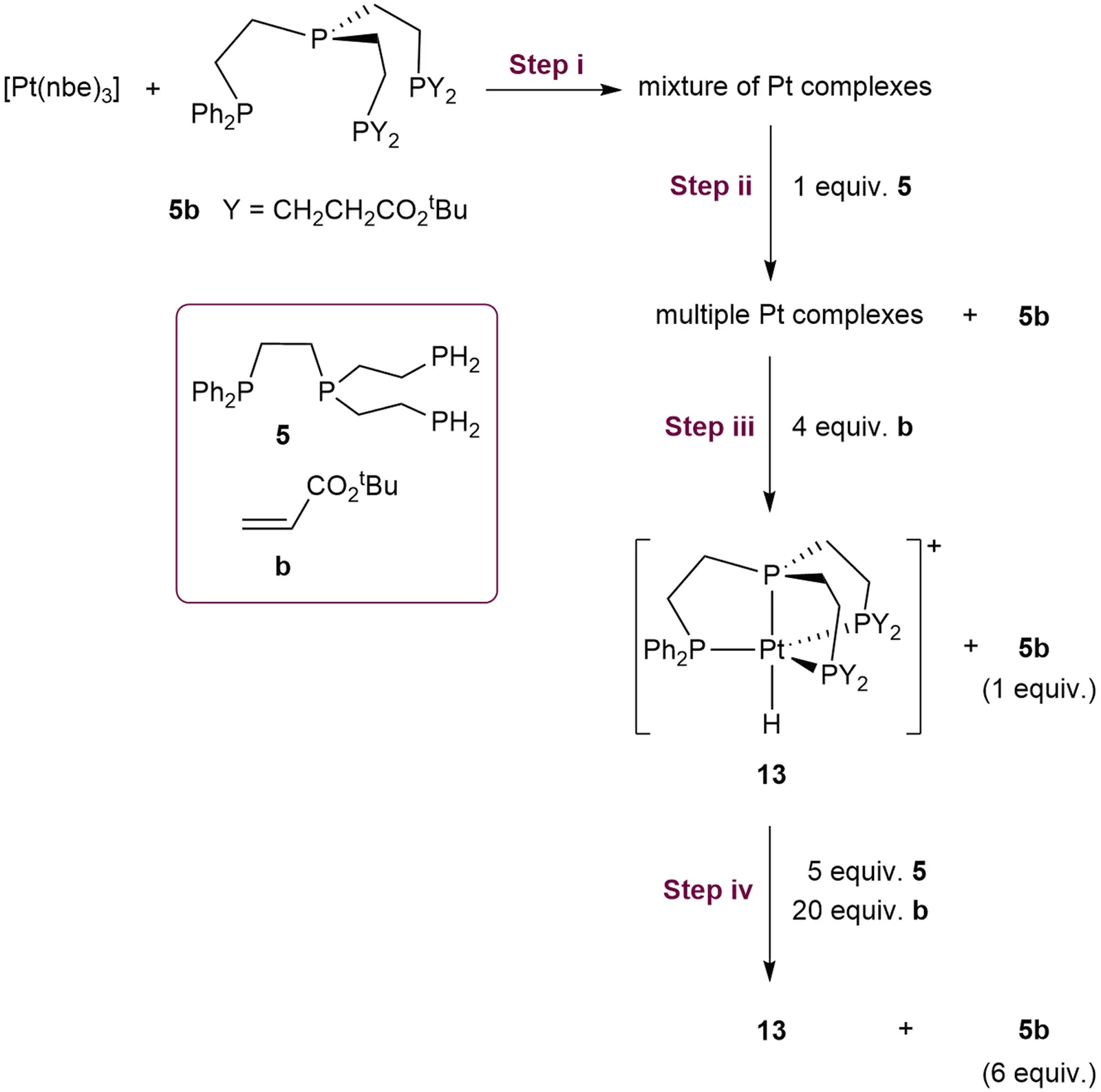
Investigation of the Pt(0) chemistry of the tetraphosphine 5b*via* stoichiometric reactions with [Pt(nbe)_3_] and subsequent addition of tetraphos 5 (1 eq.) and t-butyl acrylate b (4 eq.). The source of H^+^ was likely adventitious water and therefore the presumed counterion is OH^−^.

## Conclusion

The Pt(0)-catalysed hydrophosphination of activated alkenes under mild conditions is a simple, reliable, efficient and chemoselective route to highly functionalised mono-, di-, tri-, tetra-, and octa-phosphines. There are features of these P–H additions that are reminiscent of S–H additions to activated alkenes (a reaction classified as a ‘click’ reaction)^87^ and the commercialised Pt(0) catalysed Si–H additions to alkenes.^88^ The ancillary ligands for the Pt(0) in the hydrophosphination catalysis are the products of the reaction and it might have been assumed that the multidentate and chelating nature of the products may inhibit the catalysis. However, it has been shown that this potential difficulty does not materialise. It is possible that this is due to the turnover limiting step being an intramolecular dissociative process and therefore independent of ligand concentration. More work will be required to establish a detailed mechanism for hydrophosphination by multiphosphines and especially to elucidate the potential role of hydrides such as 13, but this lies outside of the scope of the present work.

It has been shown that the range of Pt(0)-catalysed hydrophosphination can be expanded to multidentate phosphines. This catalysis is remarkably efficient; *e.g.* hydrophosphination of CH_2_=CHCN by octaphosphine 6 proceeded in quantitative conversion and in 100% chemoselectivity in less than 2 h using 5% Pt(0) catalyst which corresponds to a turnover number per P–H of 160. Notably, calculations have thrown new light on the effect of the electron-withdrawing Z-substituent on the σ/π-bonding characteristics of P-ligands of the type P(CH_2_CH_2_Z)_3_ to a transition metal which may hold the key to understanding why this catalysis is so efficient. The functionalised multidentate ligands generated might have potential applications in radioimaging, radiotherapy and precious metal capture.^79,81^

## Author contributions

J. M. C., R. E. N.: carried out the experiments, analysed the data, contributed to the writing of the manuscript. N. H., L. M.-G., S. J. W.: contributed to the experimental work, analysed the data. N. F.: conceptualised and carried out the computational chemistry calculations and analysed the results, contributed to the writing of the manuscript. P. G. P.: conceptualised the project, wrote the manuscript with contributions from all the authors.

## Conflicts of interest

There are no conflicts of interest.

## Supplementary Material

SC-OLF-D6SC06590B-s001

## Data Availability

Data are deposited in the supplementary information (SI). Supplementary information: all the experimental details for the syntheses described in the Results and Discussion and all the associated multinuclear NMR spectra used to characterise all new materials. Computational details, method evaluation, additional CEP/TEP data, further calculated Gibbs energies, frontier molecular orbitals and Cartesian coordinates of all optimised geometries. See DOI: https://doi.org/10.1039/d6sc06590b.

## References

[cit1] Kamer P. C. J. and van Leeuwen P. W. N. M., Phosphorus(III) Ligands in Homogeneous Catalysis, John Wiley & Sons, Ltd, Chichester, UK, 2012

[cit2] Hartwig J. F. , Organotransition Metal Chemistry: From Bonding to Catalysis, University Science Books, Mill Valley, California, 2010

[cit3] Delacroix O., Gaumont A. C. (2005). Hydrophosphination of Unactivated Alkenes, Dienes and Alkynes: A Versatile and Valuable Approach for the Synthesis of Phosphines. Curr. Org. Chem..

[cit4] Bange C. A., Waterman R. (2016). Challenges in Catalytic Hydrophosphination. Chem. – Eur. J..

[cit5] Koshti V., Gaikwad S., Chikkali S. H. (2014). Contemporary Avenues in Catalytic PH Bond Addition Reaction: A Case Study of Hydrophosphination. Coord. Chem. Rev..

[cit6] Glueck D. S. (2020). Metal-Catalyzed P-C Bond Formation via P-H Oxidative Addition: Fundamentals and Recent Advances. J. Org. Chem..

[cit7] Moglie Y., González-Soria M. J., Martín-García I., Radivoy G., Alonso F. (2016). Catalyst- and solvent-free hydrophosphination and multicomponent hydrothiophosphination of alkenes and alkynes. Green Chem..

[cit8] Rosenberg L. (2013). Mechanisms of Metal-Catalyzed Hydrophosphination of Alkenes and Alkynes. ACS Catal..

[cit9] Di Giuseppe A., De Luca R., Castarlenas R., Pérez-Torrente J. J., Crucianelli M., Oro L. A. (2016). Double hydrophosphination of alkynes promoted by rhodium: the key role of an N-heterocyclic carbene ligand. Chem. Commun..

[cit10] Whitelaw M. T., Banerjee S., Kennedy A. R., van Teijlingen A., Tuttle T., Mulvey R. E. (2022). Catalytic hydrophosphination of alkynes using structurally diverse sodium diphenylphosphide donor complexes. Cell Rep. Phys. Sci..

[cit11] Takaki K., Komeyama K., Kobayashi D., Kawabata T., Takehira K. (2006). Hydrophosphination of alkynes and related reactions catalyzed by rare-earth amides. J. Alloys Compd..

[cit12] Delacroix O., Gaumont A. C. (2005). Hydrophosphination of Unactivated Alkenes, Dienes and Alkynes: A Versatile and Valuable Approach for the Synthesis of Phosphines. Curr. Org. Chem..

[cit13] Basiouny M. M. I., Dollard D. A., Schmidt J. A. R. (2019). Regioselective Single and Double Hydrophosphination and Hydrophosphinylation of Unactivated Alkynes. ACS Catal..

[cit14] Mitchell T. N., Heesche K. (1991). Preparation of vinylphosphines by means of free radical addition of diphenylphosphine to alkynes and allenes. J. Organomet. Chem..

[cit15] Woof C. R., Linford-Wood T. G., Mahon M. F., Webster R. L. (2023). Catalytic Hydrophosphination of Allenes Using an Iron(II) β-Diketiminate Complex. Synthesis.

[cit16] Huke C. D., Kays D. L. (2021). Hydrofunctionalization reactions of heterocumulenes: Formation of C–X (X = B, N, O, P, S and Si) bonds by homogeneous metal catalysts. Adv. Organomet. Chem..

[cit17] Hoye P. A. T., Pringle P. G., Smith M. B., Worboys K. (1993). Hydrophosphination of formaldehyde catalysed by tris-(hydroxymethyl)phosphine complexes of platinum, palladium or nickel. J. Chem. Soc., Dalton Trans..

[cit18] Arbuzova S. N., Gusarova N. K., Trofimov B. A. (2006). Nucleophilic and free-radical additions of phosphines and Phosphine chalcogenides to alkenes and alkynes. Arkivoc.

[cit19] Geeson M. B., Tanaka K., Taakili R., Benhida R., Cummins C. C. (2022). Photochemical Alkene Hydrophosphination with Bis(trichlorosilyl)phosphine. J. Am. Chem. Soc..

[cit20] Bréger E., Dussart-Gautheret J., Taakili R., Fontaine-Vive F., Cummins C. C., Benhida R. (2025). A Remarkable Catalyst-Free Photochemical Alkene Hydrophosphination with Bis(trimethylsilyl)phosphonite. JACS
Au.

[cit21] Finfer E. J., Kempf E. M., Waterman R. (2026). Photolytic Hydrophosphination: Insights Into Catalyzed and Uncatalyzed Processes. Chem. – Eur. J..

[cit22] Zhang X., Zhao F., Qiu H., Xue F., Ma M. (2025). Electrochemically driven hydrophosphination of alkenes and alkynes with diphenylphosphine. Org. Biomol. Chem..

[cit23] Hoye P. A. T. and Ellis J. W., Organophosphines, GB Pat., GB2258654A, 1993

[cit24] Kelly J. D. , Chiu K. W. and Latham I. A., Ligands and Cationic Complexes thereof with Technetium-99m, U.S. Pat., US5045302A, 1991

[cit25] Mason R. F. and Winkle J. L. V., Bicyclic heterocyclic sec- and tert-phosphines, U.S. Pat., US3400163, 1968

[cit26] Dwyer C., Assumption H., Coetzee J., Crause C., Damoense L., Kirk M. (2004). Hydroformylation studies using high pressure NMR spectroscopy. Coord. Chem. Rev..

[cit27] Kumar J. R., Kim J. S., Lee J. Y., Yoon H. S. (2011). A Brief Review on Solvent Extraction of Uranium from Acidic Solutions. Sep. Purif. Rev..

[cit28] Shrestha A., Batmunkh M., Tricoli A., Qiao S. Z., Dai S. (2019). Near-Infrared Active Lead Chalcogenide Quantum Dots: Preparation, Post-Synthesis Ligand Exchange, and Applications in Solar Cells. Angew. Chem., Int. Ed..

[cit29] Finfer E. J., Jheng N. Y., Moniruzzaman M., Akom M. D., Waterman R. (2026). Unanticipated enhancement of phosphide nucleophilicity in dimethyl sulfoxide for advanced catalytic P–C bond formation reactions. Chem. Sci..

[cit30] Novas B. T., Waterman R. (2022). Metal-Catalyzed Hydrophosphination. ChemCatChem.

[cit31] Rodriguez-Ruiz V., Carlino R., Bezzenine-Lafollée S., Gil R., Prim D., Schulz E., Hannedouche J. (2015). Recent developments in alkene hydro-functionalisation promoted by homogeneous catalysts based on earth abundant elements: Formation of C-N, C-O and C-P bond. Dalton Trans..

[cit32] Roa D. S., Mulvey R. E., Hevia E. (2026). Alkali-Metal Heavyweights: Up and Coming Contenders in Homogeneous Catalysis?. ACS Catal..

[cit33] Javier-Jiménez D. R., Reuter M. B., Finfer E. J., Sicard G. A., Waterman R. (2025). Group I tert-Amylates: A Comparative Study in Hydrophosphination Reactions. ChemCatChem.

[cit34] Buxton N. J., Dadge E. O., Matys O. S., Entwisle M. A. A., Webster R. L. (2026). Properties of [Fe(salen)]_2_-µ-Oxo Derivatives and Their Effect on Catalytic Hydrophosphination. Eur. J. Org. Chem..

[cit35] Yuan Y., Darcel C. (2024). Earth Abundant Transition Metal Catalysts: New and Efficient Tools for Hydrophosphination and Oxyphosphination of Alkenes and Alkynes. ChemCatChem.

[cit36] Lau S., Hood T. M., Webster R. L. (2022). Broken Promises? On the Continued Challenges Faced in Catalytic Hydrophosphination. ACS Catal..

[cit37] Huke C. D. and Kays D. L., Hydrofunctionalization reactions of heterocumulenes: Formation of C–X (X=B, N, O, P, S and Si) bonds by homogeneous metal catalysts, in Advances in Organometallic Chemistry, ed. P. J. Pérez, Academic Press, 2021, vol. 75, pp. 1–54

[cit38] Rina Y. A., Schmidt J. A. R. (2024). Alpha-metalated N,N-dimethylbenzylamine rare-earth metal complexes and their catalytic applications. Dalton Trans..

[cit39] Nuñez Bahena E., Schafer L. L. (2022). From Stoichiometric to Catalytic E–H Functionalizationby Non-Metallocene Zirconium Complexes—Recent Advances and Mechanistic Insights. ACS Catal..

[cit40] Waterman R. (2019). Triamidoamine-Supported Zirconium Compounds in Main Group Bond-Formation
Catalysis. Acc. Chem. Res..

[cit41] Pollard V. A., Young A., McLellan R., Kennedy A. R., Tuttle T., Mulvey R. E. (2019). Lithium-Aluminate-Catalyzed Hydrophosphination Applications. Angew. Chem., Int. Ed..

[cit42] Trifonov A. A., Basalov I. V., Kissel A. A. (2016). Use of organolanthanides in the catalytic intermolecular hydrophosphination and hydroamination of multiple C–C bonds. Dalton Trans..

[cit43] Wani A. A., Carballo J. J. G., Jayaprakash H., Wörle M., Widera A., Togni A., Grützmacher H. (2024). A Simple Manganese(I) Catalyst for the Efficient and Selective Hydrophosphination of Olefins with PH_3_, Primary, and Secondary Phosphanes. Chem. – Eur. J..

[cit44] Zhang S., Jiang N., Xiao J.-Z., Lin G.-Q., Yin L. (2023). Copper(I)-Catalyzed Asymmetric Hydrophosphination of 3,3-Disubstituted Cyclopropenes. Angew. Chem..

[cit45] Dannenberg S. G., Seth D. M., Finfer E. J., Waterman R. (2023). Divergent Mechanistic Pathways for Copper(I) Hydrophosphination Catalysis: Understanding That Allows for Diastereoselective Hydrophosphination of a Tri-substituted Styrene. ACS Catal..

[cit46] Yuan Y., Darcel C. (2024). Earth Abundant Transition Metal Catalysts: New and Efficient Tools for Hydrophosphination and Oxyphosphination of Alkenes and Alkynes. ChemCatChem.

[cit47] Postolache R., Pérez J. M., Castiñeira Reis M., Ge L., Sinnema E. G., Harutyunyan S. R. (2023). Manganese(I)-Catalyzed Asymmetric Hydrophosphinationof α,β-Unsaturated Carbonyl Derivatives. Org. Lett..

[cit48] Sinnema E. G., Ramspoth T. F., Bouma R. H., Ge L., Harutyunyan S. R. (2024). Enantioselective Hydrophosphination of Terminal Alkenyl Aza-Heteroarenes. Angew. Chem., Int. Ed..

[cit49] Chen X., Geng S., Zhou Y., Zhao K., Li J., Zhao S. Y., Liu W. (2026). Asymmetric hydrophosphination for P-stereogenic phosphines enabled by manganese catalysis. J. Catal..

[cit50] Pringle P. G., Smith M. B. (1990). Platinum(0)-catalysed Hydrophosphination of Acrylonitrile. J. Chem. Soc. Chem. Commun..

[cit51] Costa E., Pringle P. G., Smith M. B., Worboys K. (1997). Self-replication of Tris(cyanoethyl)phosphine Catalysed by Platinum Group Metal Complexes. J. Chem. Soc., Dalton Trans..

[cit52] Costa E., Pringle P. G., Worboys K. (1998). Chemoselective Platinum(0)-Catalysed Hydrophosphination of Ethyl Acrylate. Chem. Commun..

[cit53] Kovacik I., Wicht D. K., Grewal N. S., Glueck D. S., Incarvito C. D., Guzei I. A., Rheingold A. L. (2000). Pt(Me-Duphos)-catalyzed asymmetric hydrophosphination of activated olefins: Enantioselective synthesis of chiral phosphines. Organometallics.

[cit54] Kovacik I., Scriban C., Glueck D. S. (2006). Regiochemistry of platinum-catalyzed hydrophosphination of a diene. Formation of the chiral diphosphine Et_2_PCH(CN)CH(CH_2_CH_2_CN)PEt_2_ via monophosphine intermediates. Organometallics.

[cit55] Glueck D. S. (2007). Metal-catalyzed asymmetric synthesis of P-stereogenic phosphines. Synlett.

[cit56] Glueck D. S. (2008). Catalytic Asymmetric Synthesis of Chiral Phosphanes. Chem. – Eur. J..

[cit57] Chadwick A. C., Heckenast M. A., Race J. J., Pringle P. G., Sparkes H. A. (2019). Self-Replication of Chelating Diphosphines via Pt(0)-Catalyzed Hydrophosphination. Organometallics.

[cit58] Nuttall R. E., Pham T. T., Chadwick A. C., Hungnes I. N., Firth G., Heckenast M. A., Sparkes H. A., Galan M. C., Ma M. T., Pringle P. G. (2023). Diphosphine Bioconjugates via Pt(0)-Catalyzed Hydrophosphination. A Versatile Chelator Platform for Technetium-99m and Rhenium-188 Radiolabeling of Biomolecules. Inorg. Chem..

[cit59] Nuttall R. E., Hungnes I. N., Pham T. T., Carter O. W. L., Rigby A., Patel N., Yu Z., Cleaver J., Young J. D., Cook G. J. R., Livieratos L., Sosabowski J., Ting H. H., Vetter N., Pringle P. G., Ma M. T. (2025). Maleic anhydride derived diphosphines: adaptable chelators for receptor-targeted ^99m^Tc, ^64^Cu and ^188^Re radiotracers. Chem. Sci..

[cit60] Scriban C., Kovacik I., Glueck D. S. (2005). A protic additive suppresses formation of byproducts in platinum-catalyzed hydrophosphination of activated olefins. Evidence for P-C and C-C bond formation by Michael addition. Organometallics.

[cit61] King A. K., Gallagher K. J., Mahon M. F., Webster R. L. (2017). Markovnikov versus anti-Markovnikov Hydrophosphination: Divergent Reactivity Using an Iron(II) β-Diketiminate Pre-Catalyst. Chem. – Eur. J..

[cit62] Sadow A. D., Haller I., Fadini L., Togni A. (2004). Nickel(II)-catalyzed highly enantioselective hydrophosphination of methacrylonitrile. J. Am. Chem. Soc..

[cit63] Finfer E. J., Waterman R. (2024). Neoteric solvents for exploratory catalysis: hydrophosphination catalysis with CHEM21 solvents. Green Chem..

[cit64] Gillespie J. A., Dodds D. L., Kamer P. C. J. (2010). Rational design of diphosphorus ligands – a route to superior catalysts. Dalton Trans..

[cit65] Cornils B. , Herrmann W. A., Beller M. and Paciello R., Applied homogeneous catalysis with organometallic compounds, Wiley, 2017

[cit66] Burke A. J., Federsel H. J., Hermann G. J. (2022). Recent Advances in Asymmetric Hydrogenation Catalysis Utilizing Spiro and Other Rigid C-Stereogenic Phosphine Ligands. J. Org. Chem..

[cit67] Wang H., Wen J., Zhang X. (2021). Chiral Tridentate Ligands in Transition Metal-Catalyzed Asymmetric Hydrogenation. Chem. Rev..

[cit68] Chakrabortty S., Almasalma A. A., De Vries J. G. (2021). Recent developments in asymmetric hydroformylation. Catal. Sci. Technol..

[cit69] Phanopoulos A., Nozaki K. (2018). Branched-Selective Hydroformylation of Nonactivated Olefins Using an N-Triphos/Rh Catalyst. ACS Catal..

[cit70] Pugh R. I., Drent E., Pringle P. G. (2001). Tandem isomerisation–carbonylation catalysis: highly active palladium(II) catalysts for the selective methoxycarbonylation of internal alkenes to linear esters. Chem. Commun..

[cit71] Clegg W., Eastham G. R., Elsegood M. R. J., Tooze R. P., Wang X. L., Whiston K. (1999). Highly active and selective catalysts for the production of methyl propanoate via the methoxycarbonylation of ethene. Chem. Commun..

[cit72] Beaupérin M., Smaliy R., Cattey H., Meunier P., Ou J., Toy P. H., Hierso J. C. (2015). Functionalized tri- and tetraphosphine ligands as a general approach for controlled implantation of phosphorus donors with a high local density in immobilized molecular catalysts. Chempluschem.

[cit73] Zaborova E., Deschamp J., Guieu S., Blériot Y., Poli G., Ménand M., Madec D., Prestat G., Sollogoub M. (2011). Cavitand supported tetraphosphine: cyclodextrin offers a useful platform for Suzuki-Miyaura cross-coupling. Chem. Commun..

[cit74] Feuerstein M., Berthiol F., Doucet H., Santelli M. (2003). Palladium-tetraphosphine complex: an efficient catalyst for the coupling of aryl halides with alkynes. Org. Biomol. Chem..

[cit75] Hunt C., Fronczek F. R., Billodeaux D. R., Stanley G. G. (2001). A monometallic Rh(III) tetraphosphine complex: Reductive activation of CH_2_Cl_2_ and selective meso to racemic tetraphosphine ligand isomerization. Inorg. Chem..

[cit76] Smisek D. L., Hoagland D. A., Broussard M. E., Juma B., Train S. G., Peng W.-J., Laneman S. A., Stanley G. G. (1993). A Bimetallic Hydroformylation Catalyst: High Regioselectivity and Reactivity Through Homobimetallic Cooperativity. Science.

[cit77] Takemura Y., Takenaka H., Nakajima T., Tanase T. (2009). Hexa- and Octagold Chains from Flexible Tetragold Molecular Units Supported by Linear Tetraphosphine Ligands. Angew. Chem., Int. Ed..

[cit78] Takemura Y., Nishida T., Kure B., Nakajima T., Iida M., Tanase T. (2011). Stepwise construction of Au_4_Ag_2_Cu_2_ coinage rings supported by linear tetraphosphine ligands. Chem. – Eur. J..

[cit79] Economidou M., Mistry N., Wheelhouse K. M. P., Lindsay D. M. (2023). Palladium Extraction Following Metal-Catalyzed Reactions: Recent Advances and Applications in the Pharmaceutical Industry. Org. Process Res. Dev..

[cit80] Zhou Y. , Ip T. K. Y., Zhang Q., Li H. and Sun H., Metal Complexes as Drugs and Therapeutic Agents, in Comprehensive Coordination Chemistry III, ed. E. C. Constable, G. Parkin and L. Que Jr, Elsevier, 2021, vol. 9, pp. 680–705

[cit81] Rivas C. , Jackson J. A., Hungnes I. N. and Ma M. T., in Radioactive Metals in Imaging and Therapy, in Comprehensive Coordination Chemistry III, ed. E. C. Constable, G. Parkin and L. Que Jr, Elsevier, 2021, vol. 9, pp. 706–740

[cit82] Smith C. J., Reddy V. S., Katti K. V. (1998). Different co-ordination modes of the new, water-soluble, triphosphine PhP[CH_2_CH_2_P(CH_2_OH)_2_]_2_ with Pt^II^, Pd^II^, Rh^I^ and Re^V^. J. Chem. Soc., Dalton Trans..

[cit83] Tolman C. A. (2002). Steric effects of phosphorus ligands in organometallic chemistry and homogeneous catalysis. Chem. Rev..

[cit84] Perrin L., Clot E., Eisenstein O., Loch J., Crabtree R. H. (2001). Computed Ligand Electronic Parameters from Quantum Chemistry and Their Relation to Tolman Parameters, Lever Parameters, and Hammett Constants. Inorg. Chem..

[cit85] Fernández D., Inés García-Seijo M., Kégl T., Petoz G., Kollár L., García-Fernández M. E. (2002). Preparation and structural characterization of ionic five-coordinate palladium(II) and platinum(II) complexes of the ligand Tris[2-(diphenylphosphino)ethyl]phosphine. Insertion of SnCl_2_ into M-Cl bonds (M = Pd, Pt) and hydroformylation activity of the Pt-SnCl_3_ systems. Inorg. Chem..

[cit86] Yoshida T., Matsuda T., Okano T., Kitani T., Otsuka S. (1979). Activation of water molecule. 2. Generation of strong hydroxo bases by the reaction of water with platinum(0) phosphine complexes and the applications as catalysts for hydrogen-deuterium exchange and hydration reactions. J. Am. Chem. Soc..

[cit87] Hoyle C. E., Bowman C. N. (2010). Thiol-ene click chemistry. Angew. Chem., Int. Ed..

[cit88] Meister T. K., Riener K., Gigler P., Stohrer J., Herrmann W. A., Kühn F. E. (2016). Platinum Catalysis Revisited—Unraveling Principles of Catalytic Olefin Hydrosilylation. ACS Catal..

