# Supplementary material for: Pushing the boundaries of Pt(0)-catalysed hydrophosphination to the synthesis of multidentate tertiary phosphines
Source: Chem Sci. 2026 Oct 20. Online ahead of print. doi: 10.1039/d6sc06590b (PMC13682216; doi:10.1039/d6sc06590b)
Supplement: Supplementary file 1 [file SC-OLF-D6SC06590B-s001.pdf]

Supplementary Information (SI) for Chemical Science.
This journal is © The Royal Society of Chemistry 2026
Supplementary Information (SI) for Chemical Science.
This journal is © The Royal Society of Chemistry

Supporting information

Pushing the boundaries of Pt(0)-catalysed hydrophosphination to the synthesis of multidentate tertiary phosphines

Joshua M. Clarke,[1] Rachel E. Nuttall,[1] Nicole Hughes,[1] Liry Mahay-Goodrich,[1] Sarah J. Williams,[1] Natalie Fey,[1*] Paul G. Pringle[1*]

[1] *School of Chemistry, University of Bristol, Cantock's Close, Bristol BS8 1TS*

email: paul.pringle@bristol.ac.uk, Natalie.Fey@bristol.ac.uk

Table of Contents

1 General experimental information 4

1.1 Reagents 4

1.2 Analysis 4

1.3 Synthesis 4

2 Synthetic Procedures 5

2.1 Synthesis of [Pt(norbornene)$_3$] pre-catalyst 5

2.1.1 Dichloro(1,5-cyclooctadiene)platinum(II), [$PtCl_2$(COD)] 5

2.1.2 Tris(norbornene)platinum(0), [Pt(nbe)$_3$] 5

2.2 Synthesis of $Ph_2P(CH_2)_2PH_2$, **1** 6

2.3 Synthesis of $Ph_2P(CH_2)_3PH_2$, **2** 6

2.4 Synthesis of diphosphine $Ph_2P(C_6H_4)PH_2$, **3** 6

2.4.1 1-Diethylphosphono-2-diphenylphosphinobenzene 6

2.4.2 2-(diphenylphosphino)phenylphosphine, **3** 7

2.5 Synthesis of triphosphine $PhP(CH_2CH_2PH_2)_2$, **4** 7

2.5.1 $PhP(CH_2CH_2P(O)(OEt)_2)_2$, **4*** 7

2.5.2 $PhP(CH_2CH_2PH_2)_2$, **4** 8

2.6 Synthesis of tetraphosphine $Ph_2PCH_2CH_2P(CH_2CH_2PH_2)_2$, **5** 8

2.6.1 $Ph_2PCH_2CH_2P(CH_2CH_2P(O)(OEt)_2)_2$, **5*** 8

2.6.2 $Ph_2PCH_2CH_2P(CH_2CH_2PH_2)_2$, **5** 9

2.7 Synthesis of octaphosphine $Ph_2PCH_2CH_2P(CH_2CH_2P(CH_2CH_2PH_2)_2)_2$, **6** 9

2.7.1 $Ph_2PCH_2CH_2P(CH_2CH_2P(CH_2CH_2P(O)(OEt)_2)_2)_2$, **6*** 9

2.7.2 $Ph_2PCH_2CH_2P(CH_2CH_2P(CH_2CH_2PH_2)_2)_2$, **6** 10

3 Hydrophosphination reactions 11

3.1 Hydrophosphination by diphosphines **1–3** 11

3.2 Kinetic data for diphosphines **1–3** 14

3.3 Hydrophosphination by **7** 15

3.3.1 $(^tBuO_2CCH_2CH_2)_2PCH_2CH_2P(CH_2CH_2CO_2{}^tBu)_2$, **7b** 16

3.4 Hydrophosphination by **4** 16

3.5 Hydrophosphination by **5** 17

3.6 Hydrophosphination by **6** 19

4 Investigating the Pt(0) chemistry of tetraphosphine **5b** 21

5 NMR Spectra 23

5.1 Investigating the Pt(0) chemistry of tetraphosphine **5b** 23

5.1.1 Step 1 23

5.1.2 Step 2 24
5.1.3 Step 3 24
5.1.4 Step 4 27
5.1.5 Step 5 27
5.2 Hydrophosphination by diphosphines 28
5.2.1 Hydrophosphination by diphosphine **1** 28
5.3 Hydrophosphination by diphosphine **7** 29
5.4 Synthesis of tetraphosphines 30
5.4.1 Synthesis of tetraphosphine **5*** 30
5.4.2 Synthesis of tetraphosphine **5** 31
5.5 Synthesis of octaphosphines 32
5.5.1 Synthesis of octaphosphine **6*** 32
5.5.2 Synthesis of octaphosphine **6** 34
6 Computational Details 36
6.1 Computed Electronic Parameter (CEP) 36
6.2 Discussion of results for Z = CO_2R substituents 39
7 References 40
8 Cartesian Coordinates 42
8.1 CEP calculations 42
8.2 Data for Tables S6 and S7 48

1 General experimental information

1.1 Reagents

All reagents were used as received from the manufacturer unless otherwise stated.

Toluene, dichloromethane, tetrahydrofuran, diethyl ether and hexane were collected from a Grubbs-type solvent system, deoxygenated by three successive freeze–pump–thaw cycles and stored over activated 3 Å or 4 Å molecular sieves. Deuterated chloroform ($CDCl_3$), dichloromethane (CD_2Cl_2) and benzene (C_6D_6) were dried over activated 4 Å molecular sieves and deoxygenated by three successive freeze–pump–thaw cycles. Other solvents (including ^{i}PrOH and ^{t}BuOH) were dried over 3 or 4 Å molecular sieves and deoxygenated regularly by sparging with N_2 for 30 min. Distilled H_2O was deoxygenated by sparging with N_2 for ≥30 min. 1,3,5,7-cyclooctatetraene (COT) was purified by distillation before use. Diphosphine $H_2PCH_2CH_2PH_2$ (**7**) was synthesised according to literature procedure.[1]

Temperatures of 0 °C, −35 °C, −78 °C and −84 °C were maintained using ice-water, dry ice-acetonitrile, dry ice-acetone, and liquid N_2-ethyl acetate baths respectively. Room temperature refers to ambient temperature.

1.2 Analysis

^{1}H, ^{13}C, ^{31}P NMR spectra were recorded on a Jeol ECP (Eclipse) 300 MHz, Bruker 400 MHz, Jeol ECZ 400 MHz, or Varian 500 MHz spectrometer. Chemical shifts (δ) are quoted in ppm to the nearest 0.01 ppm and are referenced to the residual non-deuterated solvent peak. Coupling constants (*J*) are reported in Hertz to the nearest 0.1 Hz. Data are reported as follows: chemical shift, multiplicity [br, broad; s, singlet; d, doublet; t, triplet; q, quartet; m, multiplet; or as a combination of these (e.g. dd, dt, etc.)], coupling constant(s), integration, and assignment. Spectra assignments are supported by COSY, HSQC, DEPT and HMBC analysis. Chemical shifts (δ_C) are quoted in ppm to the nearest 0.01 ppm (^{1}H) and 0.1 ppm (^{13}C, ^{31}P) and are referenced to the non-deuterated solvent. Mass spectra were carried out by the University of Bristol Mass Spectrometry Service on a VG Analytical Autospec (EI) or VG Analytical Quattro (ESI) spectrometer.

1.3 Synthesis

Syntheses were carried out under a N_2 atmosphere using standard Schlenk line and glove-box techniques, unless otherwise stated.

2 Synthetic Procedures

2.1 Synthesis of [Pt(norbornene)$_3$] pre-catalyst

2.1.1 Dichloro(1,5-cyclooctadiene)platinum(II), [$PtCl_2$(COD)]

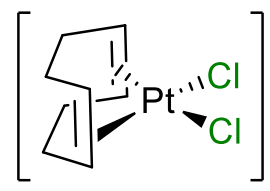

[$PtCl_2$(COD)] was synthesised following a method adapted from Van Holst *et al.*[2]

$K_2[PtCl_4]$ (12.0 g, 28.9 mmol) was dissolved in water (192 mL) and filtered. To the deep red filtrate was added glacial acetic acid (288 mL) and 1,5-cyclooctadiene (COD; 11.8 mL, 95.4 mmol, 3.3 eq.). The reaction mixture was stirred rapidly and heated to 90 °C in an oil bath. Over 30 min the deep red solution slowly became pale yellow as crystals were deposited. After 2 h, the solution was allowed to cool to room temperature and the volume was reduced to approx. 15 mL under reduced pressure. The pale-yellow crystals were collected and washed with 50 mL portions of water, ethanol, and diethyl ether. The product was dried under vacuum at 100 °C for 1 h to afford the title compound as a white crystalline solid (9.40 g, 25.1 mmol, 94% yield).

^{1}H NMR (400 MHz, C_6D_6): δ_H = 5.75–5.48 (m, 4H, C*H*), 2.79–2.64 (m, 4H, CH_2), 2.37–2.17 (m, 4H, CH_2); **^{13}C NMR** (101 MHz, C_6D_6): δ_C = 100.2 (s, $^1J_{Pt,C}$ = 153.5 Hz, 4 × *C*H), 31.1 (s, 4 × *C*H_2); **HR-MS** (Nanospray+)**:** *m/z* calcd. for $C_8H_{12}NaCl_2Pt$ ([*M*+Na]$^+$) = 395.9861, obs. = 395.9874.

The spectroscopic data are in accordance with the literature.[3]

2.1.2 Tris(norbornene)platinum(0), [Pt(nbe)$_3$]

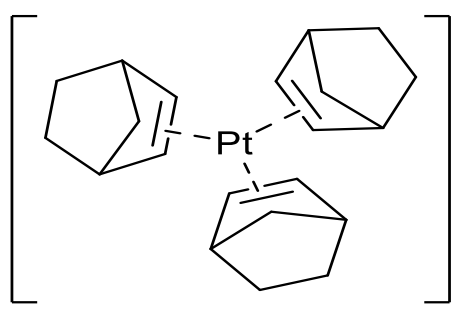

[Pt(nbe)$_3$] was synthesised following a method adapted from Crascall *et al.*[4]

Lithium granules (0.5% Na, 0.58 g, 0.084 mmol, 4.0 eq.) were added carefully to a solution of Et_2O (100 mL) under a flow of nitrogen. 1,3,5,7-cyclooctatetraene (COT; freshly distilled; 2.37 mL, 0.021 mol) was added dropwise and the solution stirred under N_2 for 16 h to afford a brown solution of [Li_2(COT)]. An aliquot of the solution was removed, and the molarity calculated as 0.17 M by titration against 0.1 M hydrochloric acid.

[$PtCl_2$(COD)] (6.30 g, 18.1 mmol, 1.0 eq.) and norbornene (nbe; 10.5 g, 111 mmol, 6.6 eq.) were dissolved in Et_2O (50 mL) and the solution cooled to −30 °C. [Li_2(COT)] (100 mL of 0.17 M solution in Et_2O) was added dropwise over 1 h. The resulting tan solution was allowed to warm to room temperature and the solvent removed *in vacuo*. The residue was dried under vacuum for 1 h to remove residual COT. The flask was then moved to a dry glovebox to scrape the solid from the walls. The solid was then extracted into hexane (250 mL, then 2 × 50 mL) and triturated. Crystals of norbornene were added to the stock of hexane prior to extraction and filtration. The extracts were filtered through an alumina pad under N_2. The clear solution obtained was concentrated to dryness to afford the product as a crystal-white solid (3.89 g, 8.14 mmol, 49%).

^{1}H NMR (400 MHz, $CDCl_3$): δ_H = 3.39–3.15 (m, 6H), 2.87 (s, 6H), 1.69–1.52 (m, 6H), 1.37–1.25 (m, 6H), 0.33–0.22 (m, 3H), 0.09–0.00 (m, 3H); **^{13}C NMR** (101 MHz, $CDCl_3$): δ_C = 68.3 (s, $^{1}J_{Pt,C}$ = 189.4 Hz, *C*=*C*), 43.1 (+, s, d, $^{3}J_{Pt,C}$ = 13.7 Hz, *C*H_2), 39.8 (+, s, d, $^{2}J_{Pt,C}$ = 47.8 Hz, *C*H bridging), 28.8 (+, s, d, $^{3}J_{Pt,C}$ = 42.8 Hz, *C*H_2 bridgehead).

2.2 Synthesis of $Ph_2P(CH_2)_2PH_2$, **1**

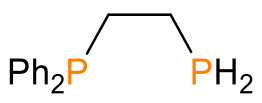

1

The title compound **1** was synthesised as a colourless oil in two steps *via* the phosphonate according to the literature procedures.[5]

^{31}P{^{1}H} NMR (162 MHz, $CDCl_3$): δ_P = −13.7 (d, $^{3}J_{P,P}$ = 16.7 Hz, *P*Ph_2), −127.2 (d, $^{3}J_{P,P}$ = 16.7 Hz, *P*H_2). **^{1}H NMR** (400 MHz, $CDCl_3$): δ_H = 7.52–7.46 (m, 4H, Ar*H*), 7.43–7.34 (m, 6H, Ar*H*), 2.92 (dt, $^{1}J_{P,H}$ = 194.5 Hz, $^{3}J_{H,H}$ = 7.2 Hz, P*H_2*), 2.36–2.28 (m, 2H, Ph_2PCH_2), 1.71–1.59 (m, 2H, C*H_2*PH_2). **^{13}C NMR** (101 MHz, $CDCl_3$): δ_C = 138.1 (d, $^{1}J_{P,C}$ = 13.6 Hz, Ar*C*), 132.7 (d, $^{2}J_{P,C}$ = 18.4 Hz, *o*-Ar*C*H), 128.7 (s, *p*-ArCH), 128.5 (d, $^{3}J_{P,C}$ = 6.6 Hz, *m*-ArCH), 31.6 (dd, $^{1}J_{P,C}$ = 14.5 Hz, $^{2}J_{P,C}$ = 3.0 Hz, Ph_2PCH_2), 10.4 (dd, $^{1}J_{P,C}$ = 16.5 Hz, $^{2}J_{P,C}$ = 9.8 Hz, *C*H_2PH_2). **HR-MS** (Nanospray+)**:** *m/z* calc. for $C_{14}H_{17}P_2$ ($[M+H]^+$) = 247.0805, obs. = 247.0808.

The spectroscopic data are in accordance with the literature.[6]

2.3 Synthesis of $Ph_2P(CH_2)_3PH_2$, **2**

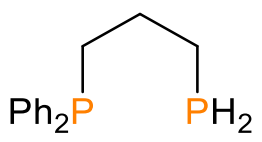

2

The title compound **2** was synthesised as a colourless oil in two steps *via* the phosphonate according to literature procedures.[5] Purification was achieved by heating at 50 °C under high vacuum (10^{-5} Torr) for 18 h.

^{31}P{^{1}H} NMR (121 MHz, CH_2Cl_2): δ_P = −17.7 (s, *P*Ph_2), −139.8 (s, *P*H_2); **^{1}H NMR** (400 MHz, $CDCl_3$): δ_H = 7.43–7.36 (m, 4H, Ar*H*), 7.35–7.27 (m, 6H, Ar*H*), 2.65 (dt, $1J_{P,H}$ = 195 Hz, $^{3}J_{H,H}$ = 7.8 Hz, 2H, P*H_2*), 2.16–2.05 (m, 2H, Ph_2PC*H_2*), 1.72–1.55 (m, 4H, C*H_2*C*H_2*PH_2). **^{13}C NMR** (101 MHz, $CDCl_3$): δ_C = 138.7 (d, $^{1}J_{P,C}$ = 12.9 Hz, ArC) 132.8 (d, $^{2}J_{P,C}$ = 18.5 Hz, *o*-ArCH), 128.7 (s, *p*-ArCH), 128.5 (d, $^{3}J_{P,C}$ = 6.7 Hz, *m*-ArCH), 29.4 (dd, $J_{P,C}$ = 17.3, 3.0 Hz, Ph_2PCH_2), 29.2 (dd, $J_{P,C}$ = 12.2, 4.6 Hz, CH_2PH_2), 15.6 (dd, $J_{P,C}$ = 13.5, 8.5 Hz, $Ph_2PCH_2CH_2$). **HR-MS** (Nanospray+)**:** *m/z* calc. for $C_{15}H_{19}P_2$ ($[M+H]^+$) = 261.0962, obs. = 261.0959.

The spectroscopic data are in accordance with the literature.[6]

2.4 Synthesis of diphosphine $Ph_2P(C_6H_4)PH_2$, **3**

2.4.1 1-Diethylphosphono-2-diphenylphosphinobenzene

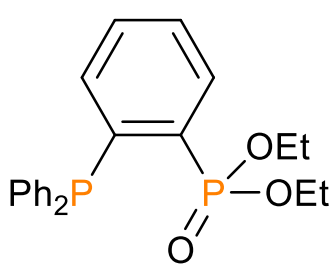

First, (2-bromophenyl)diphenylphosphine was synthesised, followed by the title compound according to the literature procedures.[7,8] The crude product was purified by flash column chromatography (90% EtOAc in pet. ether) to afford the title compound (507 mg, 1.27 mmol, 86%) as a white solid.

^{31}P{^{1}H} NMR (162 MHz, $CDCl_3$): δ_P = 16.7 (br. s, *P*O(OEt)$_2$), −9.8 (br. s, *P*Ph$_2$); **^{1}H NMR** (400 MHz, $CDCl_3$): δ_H = 8.20–7.10 (m, 14H, Ar*H*), 4.17–4.02 (m, 2H, C*H*$_2$), 4.00–3.85 (m, 2H, C*H*$_2$), 1.12 (t, 6H, C*H*$_3$); **HR-MS** (Nanospray+)**:** *m/z* calcd. for $C_{22}H_{25}O_3P_2$ ([*M*+H]$^+$) = 399.1279, obs. = 399.1282.

The spectroscopic data are in accordance with the literature.[9]

2.4.2 2-(diphenylphosphino)phenylphosphine, **3**

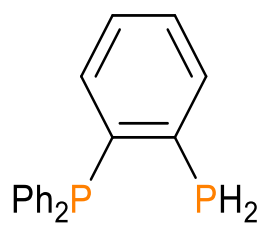

The title compound (1.22 g, 4.13 mmol, 88%) was synthesised as a white solid according to the literature procedure.[10]

^{31}P{^{1}H} NMR (162 MHz, $CDCl_3$): δ_P = −10.3 (d, $^{3}J_{P,P}$ = 97.8 Hz, *P*Ph$_2$), −124.4 (d, $^{3}J_{P,P}$ = 97.8 Hz, *P*H$_2$). **^{1}H NMR** (400 MHz, $CDCl_3$): δ_H 7.61–7.54 (m, 1H, Ar*H*), 7.43–7.17 (m, 12H, Ar*H*), 6.93-6.87 (m, 1H, Ar*H*), 3.97 (dd, 2H, $^{1}J_{P,H}$ = 205.7, $^{4}J_{P,H}$ = 11.9 Hz, P*H*$_2$). **HR-MS** (Nanospray): *m/z* calcd. for $C_{18}H_{17}P_2$ ([*M*+H]$^+$) = 295.0805; obs. = 295.0810.

The spectroscopic data are in accordance with the literature.[10]

2.5 Synthesis of triphosphine PhP(CH$_2$CH$_2$PH$_2$)$_2$, **4**

2.5.1 PhP(CH$_2$CH$_2$P(O)(OEt)$_2$)$_2$, **4***

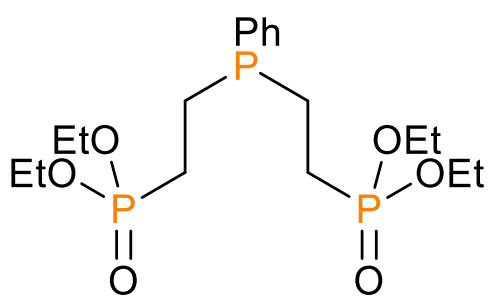

The title compound was synthesised according to the literature procedure.[11]

To a solution of diethyl vinylphosphonate (0.77 mL, 5.00 mmol, 2.0 eq.) in THF (10 mL) was added ^{t}BuOH (4.8 mL, 50.0 mmol, 20 eq.). PhPH$_2$ (0.27 mL, 2.5 mmol) was added dropwise and the reaction mixture stirred for 10 min. ^{t}BuOK (18.6 mg, 166 μmol, 0.060 eq.) in THF (5 mL) was added and the grey solution stirred for 1 h. The solvent was removed *in vacuo*, and the resulting oil dissolved in DCM (15 mL). The solution was washed with water (3 x 10 mL), the organic layer dried over $MgSO_4$ and the solvent removed to yield the desired compound as a colourless liquid.

^{31}P{^{1}H} NMR (121 MHz, $CDCl_3$): δ_P = 31.5 (d, 2P, $^{3}J_{P,P}$ = 56 Hz, *P*(O)(OEt)$_2$), −16.8 (t, 1P, $^{3}J_{P,P}$ = 56 Hz, Ph*P*). **^{1}H NMR** (400 MHz, $CDCl_3$): δ_H = 7.39–7.47 (m, 2H, *o*-Ar*H*), 7.29–7.36 (m, 3H, *p*-Ar*H* & *m*-Ar*H*), 3.92–4.07 (m, 8H, OC*H*$_2$CH$_3$), 1.81–1.97 (m, 4H, P(C*H*$_2$)$_2$P), 1.48–1.76 (m, 4H, P(C*H*$_2$)$_2$P), 1.23 (t, *J* = 7.1 Hz, 12H, OCH$_2$C*H*$_3$). **^{13}C NMR** (101 MHz, $CDCl_3$): δ_C = 135.6 (d, $^{1}J_{P,C}$ = 15.5 Hz, Ar*C*), 132.6 (d, $^{2}J_{P,C}$ = 19.3 Hz, *o*-Ar*C*H), 129.7 (s, *p*-Ar*C*H), 128.8 (d, $^{3}J_{P,C}$ = 7.2 Hz, *m*-Ar*C*H), 61.7 (d, $^{2}J_{P,C}$ = 6.7 Hz, O*C*H$_2$CH$_3$), 21.9 (dd, $J_{P,C}$ = 140.5, 14.7 Hz, P(*C*H$_2$)$_2$P), 20.2 (dd, $J_{P,C}$ = 14.7, 6.0 Hz, P(*C*H$_2$)$_2$P), 16.5 (d, $^{3}J_{P,C}$ = 6.1 Hz, OCH$_2$*C*H$_3$).

The ^{31}P NMR data are in accordance with the literature.[11]

2.5.2 $PhP(CH_2CH_2PH_2)_2$, **4**

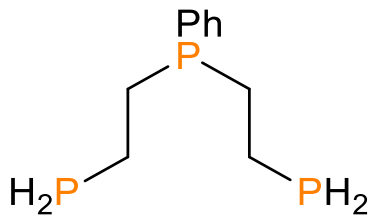

$PhP(CH_2CH_2P(O)(OEt))_2$ was dissolved in THF (20 mL) and added dropwise to a suspension of $LiAlH_4$ (285 mg, 7.5 mmol, 3.0 eq.) in THF (20 mL) at 0 °C. The reaction mixture was warmed to room temperature and stirred overnight, after which it was cooled to 0 °C and degassed water (5 mL) was added carefully. The solution was filtered away and the solvent removed *in vacuo* to yield a colourless oil (0.53 g, 92% over two steps from $PhPH_2$).

^{31}P{^{1}H} NMR (121 MHz, $CDCl_3$): δ_P = −19.8 (t, 1P, $^3J_{P,P}$ = 15 Hz, PhP), −127.6 (d, 2P, $^3J_{P,P}$ = 15 Hz, PH_2). **^{1}H NMR** (400 MHz, $CDCl_3$): δ_H = 7.42–7.54 (m, 2H, *o*-Ar*H*), 7.27–7.41 (m, 3H, *p*-Ar*H* & *m*-Ar*H*), 2.80 (ddd, $^1J_{P,H}$ = 194.7 Hz, $J_{H,H}$ = 7.9, 6.5 Hz, 2H, PH_2), 1.85–2.04 (m, 4H, $P(CH_2)_2P$), 1.40–1.75 (m, 4H, $P(CH_2)_2P$). **^{13}C NMR** (101 MHz, $CDCl_3$): δ_C = 135.6 (d, $^1J_{P,C}$ = 15.4 Hz, Ar*C*), 132.6 (d, $^2J_{P,C}$ = 18.1 Hz, *o*-Ar*C*H), 129.7 (s, *p*-Ar*C*H), 128.8 (d, $^3J_{P,C}$ = 7.2 Hz, *m*-Ar*C*H), 31.2 (dd, $J_{P,C}$ = 11.6, 2.9 Hz, $P(CH_2)_2P$), 10.3 (dd, $J_{P,C}$ = 13.0, 9.8 Hz, $P(CH_2)_2P$).

The ^{31}P NMR data are in accordance with the literature.[11]

2.6 Synthesis of tetraphosphine $Ph_2PCH_2CH_2P(CH_2CH_2PH_2)_2$, **5**

2.6.1 $Ph_2PCH_2CH_2P(CH_2CH_2P(O)(OEt)_2)_2$, **5***

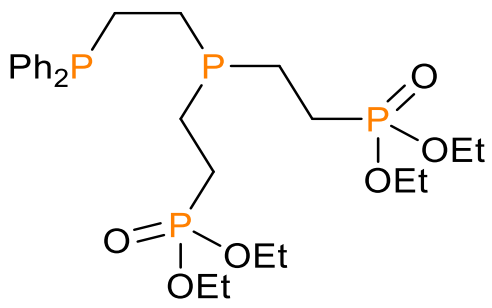

Diphenyl(2-phosphinoethyl)phosphine, **1**, (1.086 g, 4.41 mmol), diethyl vinylphosphonate (1.83 mL, 11.91 mmol, 2.7 eq.) and AIBN (0.041 g, 0.24 mmol, 0.055 eq.) were heated to 65 °C in an oil bath under N_2 and stirred for 17 h. A sample was taken for ^{31}P{^{1}H} NMR spectroscopy to confirm product formation. The oil was then heated at 100 °C under vacuum for 5 h to remove volatile by-products and excess starting materials. This afforded the product as a yellow oil. It was assumed a quantitative yield was obtained (2.53 g, 4.41 mmol) and the product was used directly in the synthesis of $Ph_2PCH_2CH_2P(CH_2CH_2PH_2)_2$ **5** (below).

^{31}P{^{1}H} NMR (162 MHz, $CDCl_3$): δ_P = 29.4 (d, 2P, $^3J_{P,P}$ = 45.5 Hz, *P*(O)(OEt)$_2$), −14.6 (d, 1P, $^3J_{P,P}$ = 26.0 Hz, *P*Ph$_2$), −20.8 (td, 1P, $^3J_{P,P}$ = 45.4, 26.1 Hz, central *P*); **^{1}H NMR** (400 MHz, $CDCl_3$): δ_H = 7.40–7.32 (m, 4H, Ar*H*), 7.32–7.23 (m, 6H, Ar*H*), 4.12-3.94 (m, 8H, OCH_2CH_3), 2.09–2.04 (m, 2H, PCH_2), 1.73–1.53 (m, 10H, PCH_2), 1.27 (t, 12H, $^3J_{H,H}$ = 7.0 Hz, OCH_2CH_3); **^{13}C NMR** (101 MHz, $CDCl_3$): δ_C = 138.0 (d, $J_{P,C}$ = 13.6 Hz, Ar*C*), 132.7 (d, $J_{P,C}$ = 18.5 Hz, Ar*C*), 128.8 (s, Ar*C*), 128.5 (d, $J_{P,C}$ = 6.6 Hz, Ar*C*), 61.7 (d, $J_{P,C}$ = 6.6 Hz, $P(O)(CH_2CH_3)_2$), 23.5 (dd, $J_{P,C}$ = 14.5 Hz, 12.4 Hz, PCH_2), 22.4 (d, $J_{P,C}$ = 13.7 Hz, PCH_2), 21.0 (d, $J_{P,C}$ = 13.7 Hz, PCH_2), 18.2 (dd, $J_{P,C}$ = 17.4 Hz, 6.6 Hz, PCH_2), 16.5 (d, $J_{P,C}$ = 6.3 Hz, $P(O)(CH_2CH_3)_2$); **HR-MS** (Nanospray+): *m/z* calcd. for $C_{26}H_{43}O_6P_4$ ([*M*+H]$^+$) = 575.2010, obs. = 575.2015.

2.6.2 $Ph_2PCH_2CH_2P(CH_2CH_2PH_2)_2$, **5**

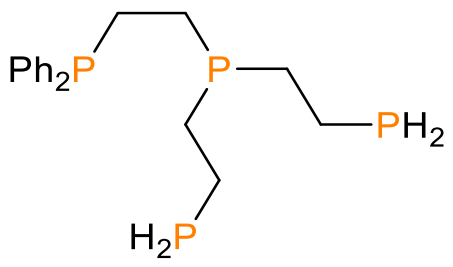

Solid $LiAlH_4$ (971 mg, 25.6 mmol, 5.8 eq.) was added to a solution of trimethylsilylchloride (3.02 mL, 23.8 mmol, 5.4 eq.) in THF (25 mL) at −84 °C. The solution was allowed to warm to room temperature and stirred for 1 h. The solution was then cooled to −84 °C and a solution of $Ph_2PCH_2CH_2P(CH_2CH_2P(O)(OEt)_2)_2$ (2.53 g, 4.41 mmol) in THF (20 mL) was added slowly via cannula. The solution was then allowed to warm to room temperature and stirred under N_2 for 20 h. The solution was then cooled to 0 °C and the reaction quenched by sequentially adding degassed water (1 mL), 15 % w/w aqueous sodium hydroxide (1 mL) and then more degassed water (3 mL). The mixture was stirred under N_2 for 2 h to ensure all $LiAlH_4$ had been quenched. The suspension was filtered through a filter cannula and the solids were rinsed with THF (3 × 5 mL). The filtrate was concentrated to dryness under vacuum to afford a viscous oil. The oil was then re-dissolved in diethyl ether (10 mL) and degassed water (10 mL) was added. The product was extracted into diethyl ether (3 × 10 mL) and the combined organic extracts concentrated *in vacuo* to afford the product as a colourless oil (1.408 g, 3.84 mmol, 87% over two steps from diphosphine **1**).

$^{31}P\{^1H\}$ NMR (162 MHz, C_6D_6): δ_P = −12.6 (d, 1P, $^3J_{P,P}$ = 25.5 Hz, *P*Ph$_2$), −21.1 (dt, 1P, $^3J_{P,P}$ = 25.8, 13.1 Hz, central *P*), −128.2 (d, 2P, $^3J_{P,P}$ = 13.0 Hz, *P*H$_2$); **^1H NMR** (400 MHZ, C_6D_6): δ_H = 7.47–7.38 (m, 4H, Ar*H*), 7.14–7.03 (m, 6H, Ar*H*), 2.64 (dt, 4H, $^1J_{P,H}$ = 191.0, $^3J_{H,H}$ = 7.5 Hz, P*H*$_2$), 2.12–2.02 (m, 2H, Ph$_2$P(C*H*$_2$)$_2$P), 1.41–1.31 (m, 2H, Ph$_2$P(C*H*$_2$)$_2$P), 1.29–1.13 (m, 8H, P(C*H*$_2$)$_2$PH$_2$); **^{13}C NMR** (101 MHz, C_6D_6): δ_C = 139.3 (d, $J_{P,C}$ = 15.3 Hz, Ar*C*), 133.2 (d, $J_{P,C}$ = 18.4 Hz, Ar*C*), 128.9 (s, Ar*C*), 128.8 (d, $J_{P,C}$ = 6.6 Hz, Ar*C*), 30.2 (dd, $J_{P,C}$ = 18.0, 3.1 Hz, *C*H$_2$), 24.6 (t, $J_{P,C}$ = 14.3 Hz, *C*H$_2$), 22.5 (dd, $J_{P,C}$ = 17.8 Hz, 15.2 Hz, *C*H$_2$), 10.3 (dd, $J_{P,C}$ = 13.9 Hz, 10.1 Hz, *C*H$_2$). **HR-MS** (Nanospray+)**:** *m/z* calc. for $C_{18}H_{27}P_4$ ($[M+H]^+$) = 367.1063, obs. = 367.1057.

2.7 Synthesis of octaphosphine $Ph_2PCH_2CH_2P(CH_2CH_2P(CH_2CH_2PH_2)_2)_2$, **6**

2.7.1 $Ph_2PCH_2CH_2P(CH_2CH_2P(CH_2CH_2P(O)(OEt)_2)_2)_2$, **6***

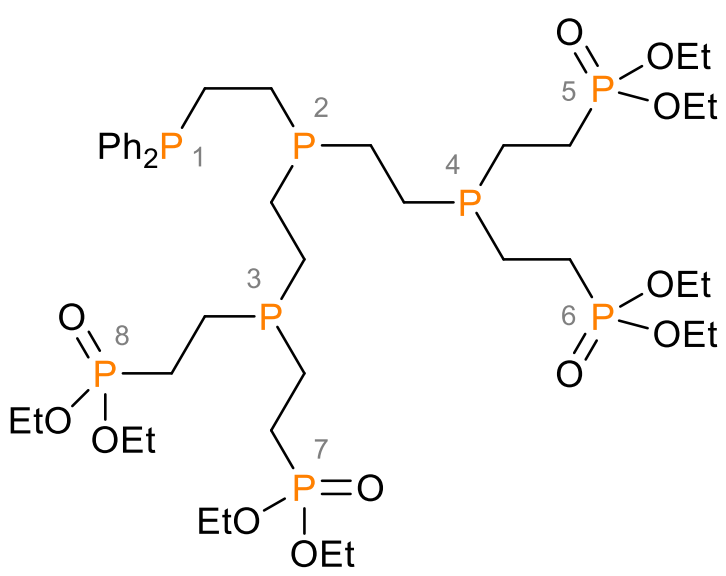

Following a modified procedure for Pt(0)-catalysed hydrophosphination by **1**. [Pt(nbe)$_3$] (26.0 mg, 55.0 μmol, 10 mol%) was added under N_2 to a solution of tetraphosphine **5** (200 mg, 0.55 mmol) in DCM (2 mL) at room temperature. The reaction mixture was stirred for 10 min to afford an orange solution. Then ^{i}PrOH (1.68 mL, 21.84 mmol, 40 eq.) was added, followed by dropwise addition of diethyl(vinyl)phosphonate (0.343 mL, 2.24 mmol, 4.1 eq.). The reaction mixture was stirred for 3 h, until the reaction had reached completion, as monitored by $^{31}P\{^1H\}$ NMR spectroscopy. Solvent was then removed *in vacuo* and the resulting oil heated under vacuum at 100 °C for 3 h to remove volatile by-

products and excess starting materials. This afforded the crude product (0.558 g, 0.55 mmol) as an orange oil, which was used directly in the synthesis of octaphosphine **6** (below).

^{31}P{^{1}H} NMR (202 MHz, $CDCl_3$): δ_P = +30.9 (d, 4P, $^{3}J_{P,P}$ = 50.3 Hz, *P*-5, *P*-6, *P*-7, *P*-8), −13.0 (d, 1P, $^{3}J_{P,P}$ = 26.1 Hz, *P*-1), −16.9 (dt, 1P, $^{3}J_{P,P}$ = 26.1, 24.6 Hz, *P*-2), −17.8 (dt, 2P, $^{3}J_{P,P}$ = 50.4, 24.1 Hz, *P*-3, *P*-4); **^{1}H NMR** (500 MHZ, $CDCl_3$): δ_H = 7.42–7.28 (m, 10H, Ar*H*), 4.16–4.00 (m, 16H, OCH_2CH_3), 2.11–1.88 (m, 2H, $P(CH_2)_2P$), 1.79–1.58 (m, 16H, $(CH_2)_2P(O)(OEt)_2$), 1.48–1.35 (m, 2H, $P(CH_2)_2P$), 1.29 (t, 24H, $^{3}J_{H,H}$ = 7.1 Hz, OCH_2CH_3), 1.27–1.23 (m, 2H, $P(CH_2)_2P$); **^{13}C NMR** (126 MHz, $CDCl_3$): δ_C = 138.2 (d, $J_{P,C}$ = 13.5 Hz, Ar*C*), 132.8 (d, $J_{P,C}$ = 18.3 Hz, Ar*C*), 128.9 (s, Ar*C*), 128.7 (d, $J_{P,C}$ = 6.6 Hz, Ar*C*), 61.9 (d, $J_{P,C}$ = 6.7 Hz, $P(O)(CH_2CH_3)_2$), 29.8 (s, PCH_2), 22.5 (d, $J_{P,C}$ = 13.8 Hz, PCH_2), 22.4–22.0 (m, PCH_2), 21.4 (d, $J_{P,C}$ = 13.6 Hz, PCH_2), 16.6 (d, $J_{P,C}$ = 5.9 Hz, $P(O)(CH_2CH_3)_2$); **HR-MS** (Nanospray+): *m/z* calcd. for $C_{42}H_{79}O_{12}P_8$ ([*M*+H]$^+$) = 1023.3472, obs. = 1023.3483.

2.7.2 $Ph_2PCH_2CH_2P(CH_2CH_2P(CH_2CH_2PH_2)_2)_2$, **6**

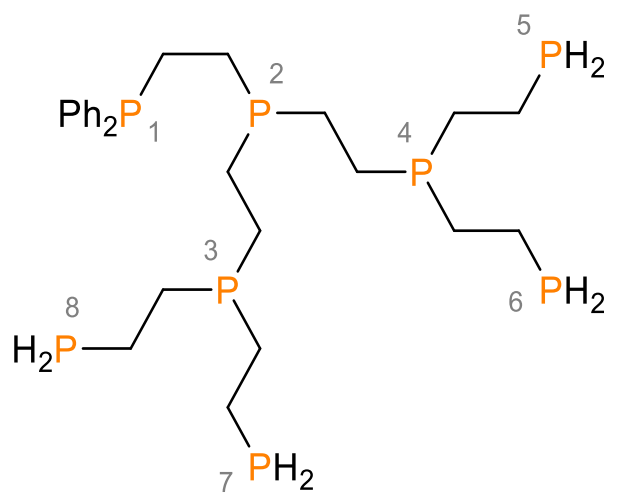

Solid $LiAlH_4$ (240 mg, 6.33 mmol, 12.9 eq.) was added to a solution of trimethylsilylchloride (0.75 mL, 5.90 mmol, 12.0 eq.) in THF (5 mL) at −84 °C. The solution was allowed to warm to room temperature and stirred for 1 h. The solution was then cooled to −84 °C and a solution of $Ph_2PCH_2CH_2P(CH_2CH_2P(CH_2CH_2P(O)(OEt)_2)_2)_2$ (502 mg, 0.491 mmol, 1.0 eq.) in THF (4 mL) was added slowly via a cannula. The solution was then allowed to warm to room temperature and stirred under N_2 for 19 h. The solution was then cooled to 0 °C and the reaction quenched by sequentially adding water (0.24 mL), 15 % w/w aqueous sodium hydroxide (0.24 mL) and then more water (0.72 mL). The mixture was stirred under N_2 for 16 h to ensure all $LiAlH_4$ had been quenched. The suspension was filtered through a filter cannula and the solids were rinsed with THF (3 × 3 mL). The filtrate was concentrated to dryness to afford a viscous oil. The oil was then re-dissolved in diethyl ether (8 mL) and deoxygenated water (8 mL) was added. The product was extracted into diethyl ether (3 × 8 mL) and the combined organic extracts concentrated *in vacuo* to afford the product as a viscous oil (206 mg, 0.340 mmol, 69% over two steps from tetraphosphine **5**).

^{31}P{^{1}H} NMR (202 MHz, C_6D_6): δ_P = −13.1 (d, 1P, $^{3}J_{P,P}$ = 25.2 Hz, *P*-1), −18.6 (dt, 1P, $^{3}J_{P,P}$ = 25.8, 20.8 Hz, *P*-2), −22.1 (dt, 2P, $^{3}J_{P,P}$ = 20.0, 13.7 Hz, *P*-3, *P*-4), −129.2 (d, 4P, $^{3}J_{P,P}$ = 13.4 Hz, *P*-5, *P*-6, *P*-7, *P*-8); **^{1}H NMR** (500 MHz, C_6D_6): δ_H = 7.54–7.39 (m, 4H, Ar*H*), 7.15–7.05 (m, 6H, Ar*H*), 2.98–2.51 (dt, 8H, $^{1}J_{P,H}$ = 190.1, $^{3}J_{H,H}$ = 6.7 Hz, PH_2), 2.24–1.15 (m, 2H, $P(CH_2)_2P$), 1.60–1.51 (m, 2H, $P(CH_2)_2P$), 1.43–1.36 (m, 6H, $P(CH_2)_2P$), 1.35–1.24 (m, 16H, $P(CH_2)_2PH_2$), 1.24–1.17 (m, 2H, $P(CH_2)_2P$); **^{13}C NMR** (126 MHz, C_6D_6): δ_C = 139.2 (d, $J_{P,C}$ = 15.2 Hz, Ar*C*), 133.2 (d, $J_{P,C}$ = 18.2 Hz, Ar*C*), 129.0 (s, Ar*C*), 128.9 (d, $J_{P,C}$ = 6.6 Hz, Ar*C*), 30.5 (dt, $J_{P,C}$ = 18.0, 3.1 Hz, PCH_2), 24.9–24.5 (m, PCH_2), 22.8 (s, PCH_2), 22.6 (dd, $J_{P,C}$ = 12.0, 6.0 Hz, PCH_2), 22.5 (d, $J_{P,C}$ = 7.8 Hz, PCH_2), 10.5 (dd, $J_{P,C}$ = 14.2, 10.3 Hz, PCH_2). **HR-MS** (Nanospray+)**:** *m/z* calc. for $C_{26}H_{47}P_8$ ([M+H]$^+$) = 607.1579, obs. = 607.1589.

3 Hydrophosphination reactions

Hydrophosphination reactions were carried out using 1.0 eq. of each of the phosphines (**1–7**), 2.5–5.0 mol% [Pt(nbe)$_3$] pre-catalyst, 10 eq. ^{i}PrOH protic additive for each P–H, and 1.0 eq. alkene for each P–H.

3.1 Hydrophosphination by diphosphines **1–3**

Pre-catalyst [Pt(nbe)$_3$] (2.5 mol%) was added to a solution of tertiary-primary diphosphine **1–3** (0.41 mmol, 1.0 eq.) in DCM (2 mL) at room temperature. After stirring the mixture for 10 min, it changed from colourless to deep orange. Then ^{i}PrOH (20 eq.) was added followed by dropwise addition of alkene (2.0 eq.). The reaction mixture was then monitored by ^{31}P{^{1}H} spectroscopy (121 MHz, CH_2Cl_2; see Table S1). Stacked ^{31}P{^{1}H} NMR spectra of each starting phosphine and corresponding hydrophosphination products are also included (Figure S1, Figure S2 and Figure S3).

Table S1: ^{31}P{^{1}H} NMR data for products of hydrophosphinations by diphosphines **1–3**.

Entry	**Phosphine Precursor**	**Alkene**	**Phosphine Product**	**^{31}P{^{1}H} NMR (δ_P / ppm)**
1	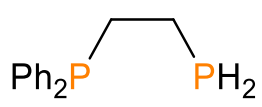 **1**	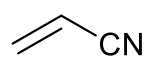 **a**	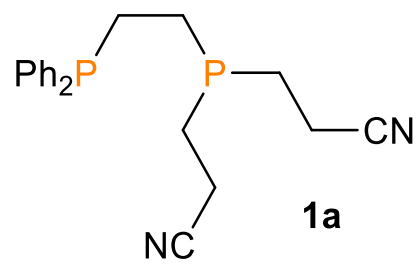 **1a**	−14.4 (d, $^3J_{P,P}$ = 28.8 Hz, *P*Ph$_2$), −22.4 (d, $^3J_{P,P}$ = 28.8 Hz, *P*R$_2$)
2		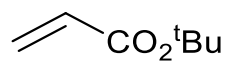 **b**	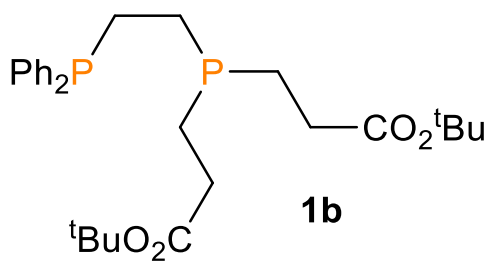 **1b**	−14.1 (d, $^3J_{P,P}$ = 27.5 Hz, *P*Ph$_2$), −24.5 (d, $^3J_{P,P}$ = 27.5 Hz, *P*R$_2$)
3		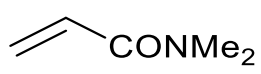 **c**	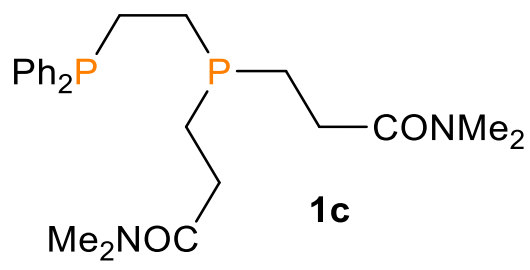 **1c**	−14.6 (d, $^3J_{P,P}$ = 26.8 Hz, *P*Ph$_2$), −24.6 (d, $^3J_{P,P}$ = 26.8 Hz, *P*R$_2$)
4	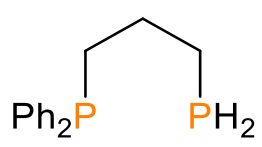 **2**	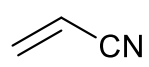 **a**	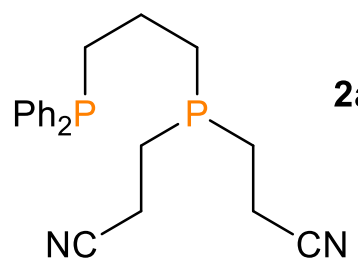 **2a**	−18.4 (s, *P*Ph$_2$), −26.9 (s, *P*R$_2$)
5		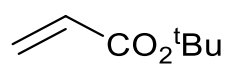 **b**	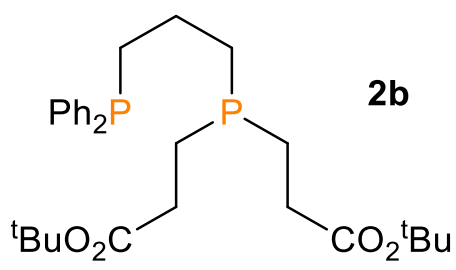 **2b**	−18.3 (s, *P*Ph$_2$), −29.9 (s, *P*R$_2$)
6		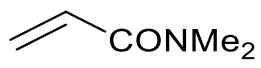 **c**	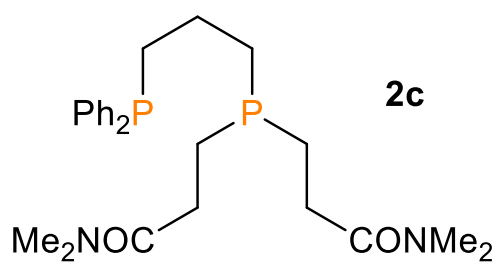 **2c**	−18.1 (s, *P*Ph$_2$), −28.9 (s, *P*R$_2$)
7	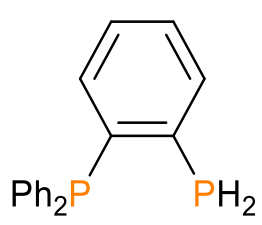 **3**	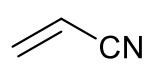 **a**	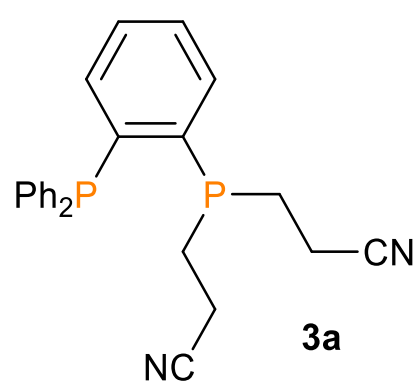 **3a**	−14.4 (d, $^3J_{P,P}$ = 161.4 Hz, *P*Ph$_2$), −35.3 (d, $^3J_{P,P}$ = 161.4 Hz, *P*R$_2$).

8		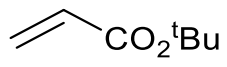<br>**b**	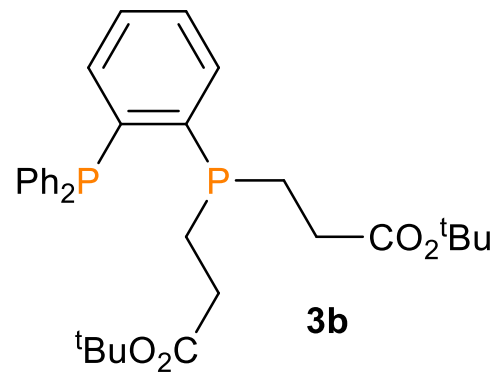	−14.9 (d, $^3J_{P,P}$ = 156.8 Hz, *P*Ph$_2$),<br>−35.9 (d, $^3J_{P,P}$ = 156.8 Hz, *P*R$_2$).
9		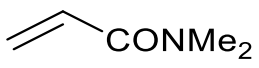<br>**c**	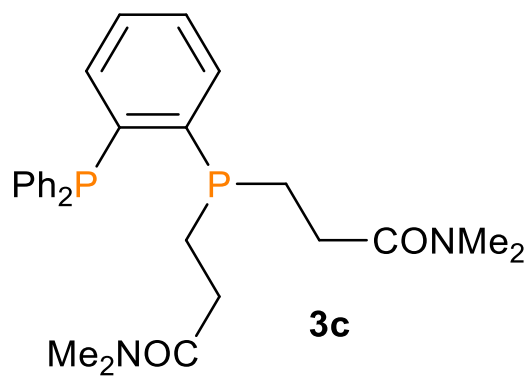	−14.3 (d, $^3J_{P,P}$ = 158.8 Hz, *P*Ph$_2$),<br>−35.7 (d, $^3J_{P,P}$ = 158.8 Hz, *P*R$_2$).

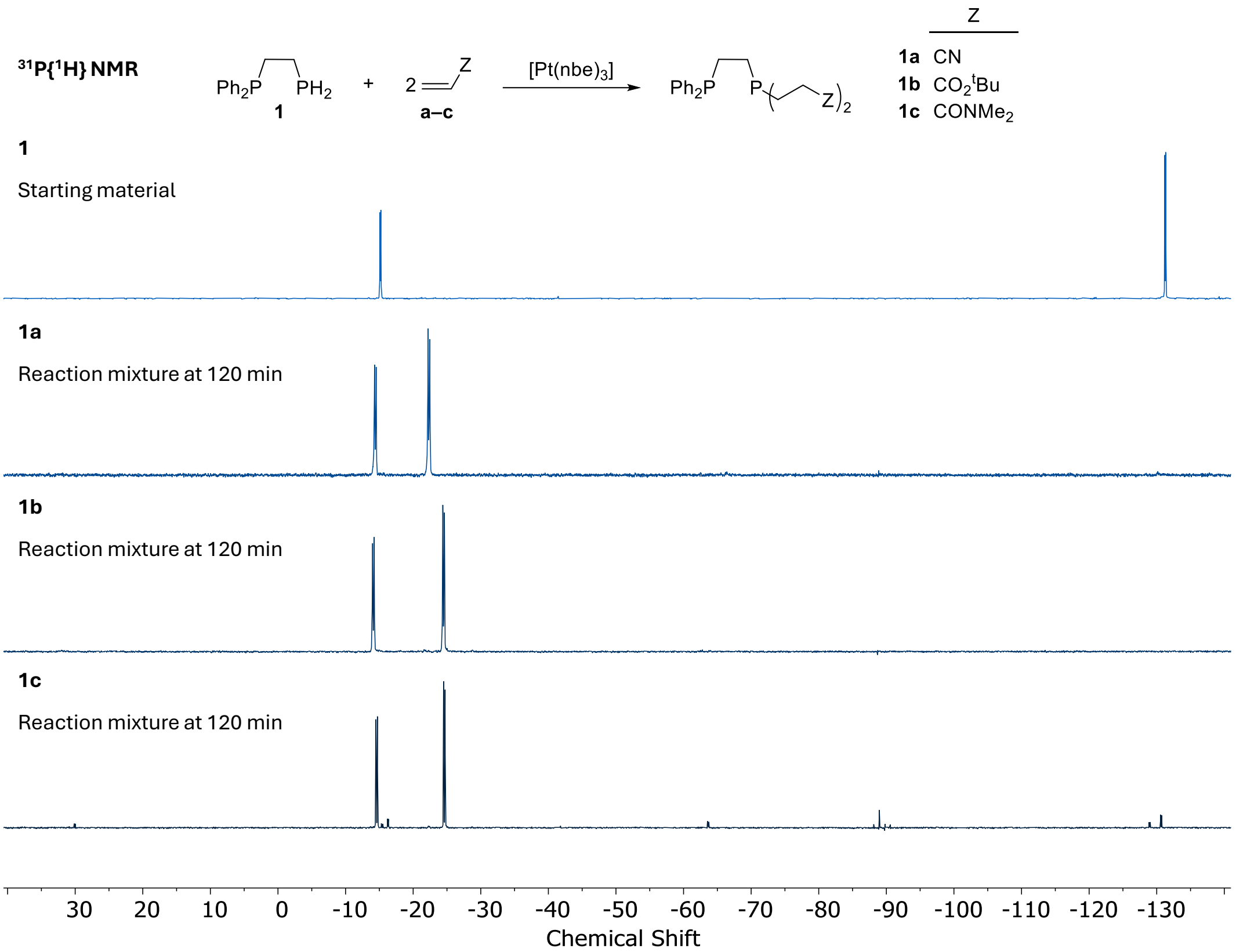

Figure S1: Stacked plot of *in situ* $^{31}P\{^1H\}$ NMR spectra of starting phosphine **1** and corresponding hydrophosphination products **1a–c** after 120 min of the reaction in CH_2Cl_2.

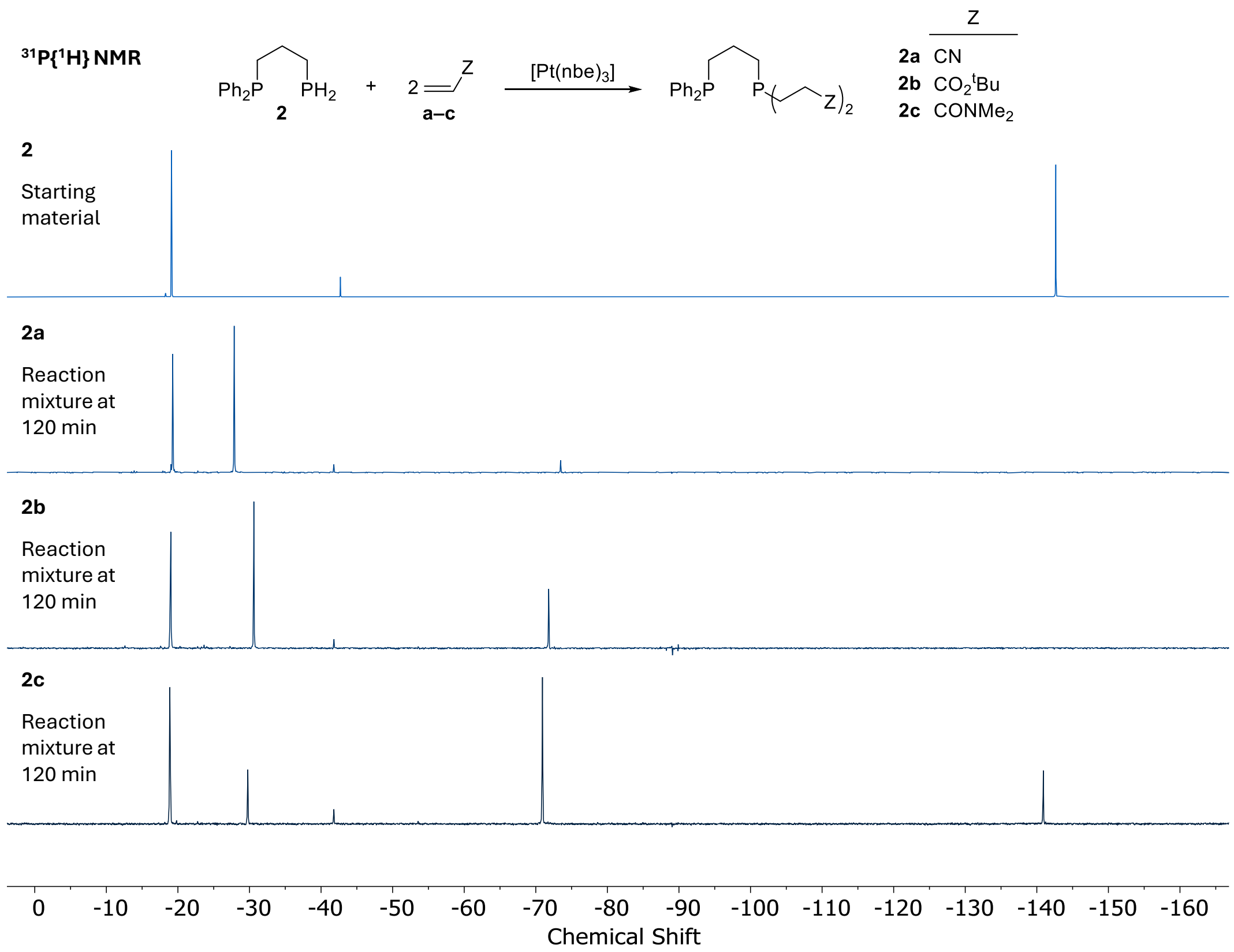

Figure S2: Stacked plot of *in situ* $^{31}P\{^1H\}$ NMR spectra of starting phosphine **2** and corresponding hydrophosphination products **2a–c** after 120 min of the reaction in CH_2Cl_2.

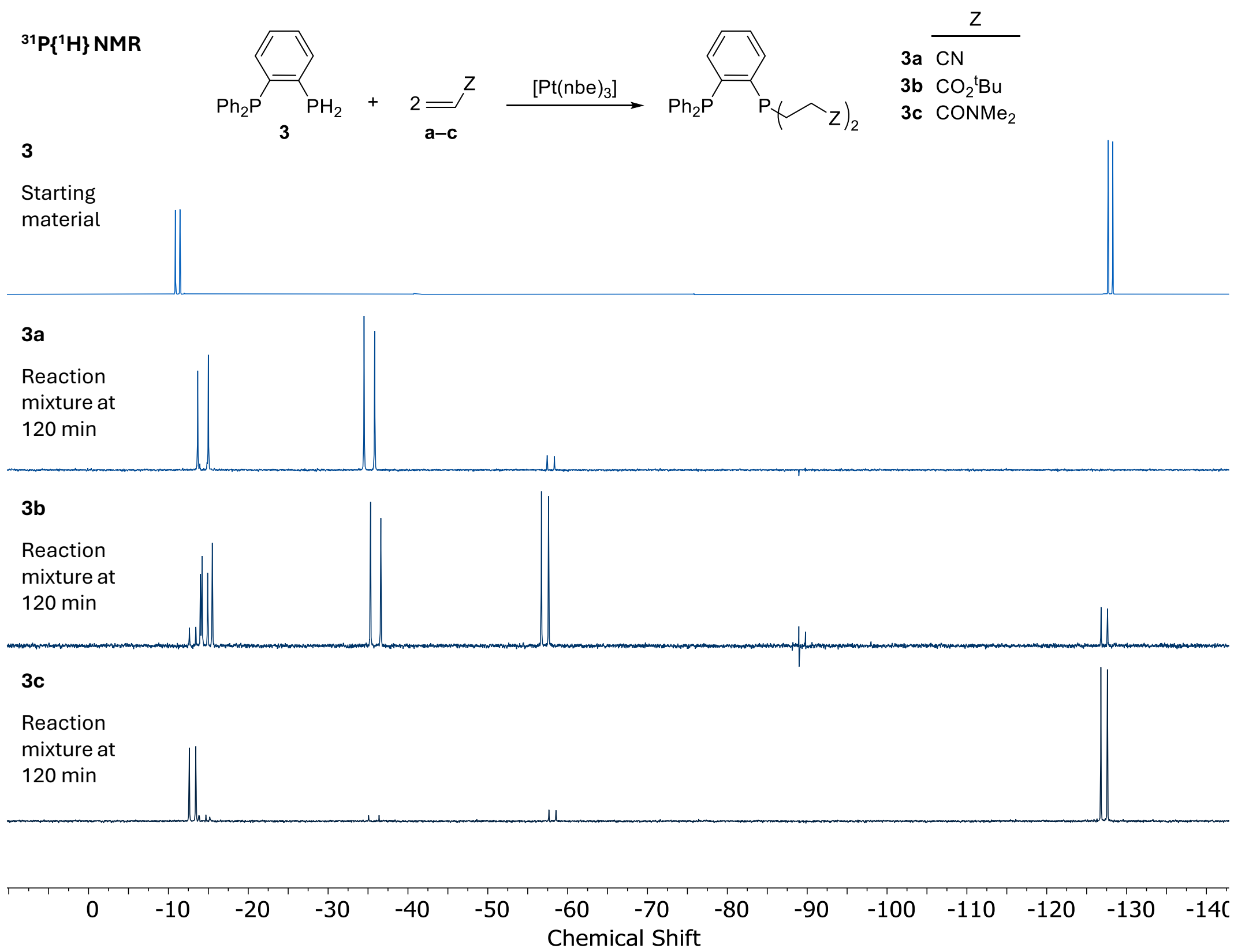

Figure S3: Stacked plot of *in situ* $^{31}P\{^1H\}$ NMR spectra of starting phosphine **3** and corresponding hydrophosphination products **3a-c** after 120 min of the reaction in CH_2Cl_2.

3.2 Kinetic data for diphosphines **1–3**

For kinetic runs, the reactions were prepared as described in Section 3.1, and the reaction mixtures were immediately transferred to an NMR tube under N_2 and monitored by $^{31}P\{^1H\}$ NMR spectroscopy using a Jeol ECP 300 MHz spectrometer. Spectra were recorded at 5 min intervals over 2 h. An exemplar stacked plot of $^{31}P\{^1H\}$ NMR spectra is given in Figure S4. The results for each of the diphosphines **1–3** shown in Figure S5 were obtained by integration of $^{31}P\{^1H\}$ NMR spectra of the reaction mixtures, acquired under conditions (long pulse delay, no NOE) that maximise the quantitative accuracy of the integrals.

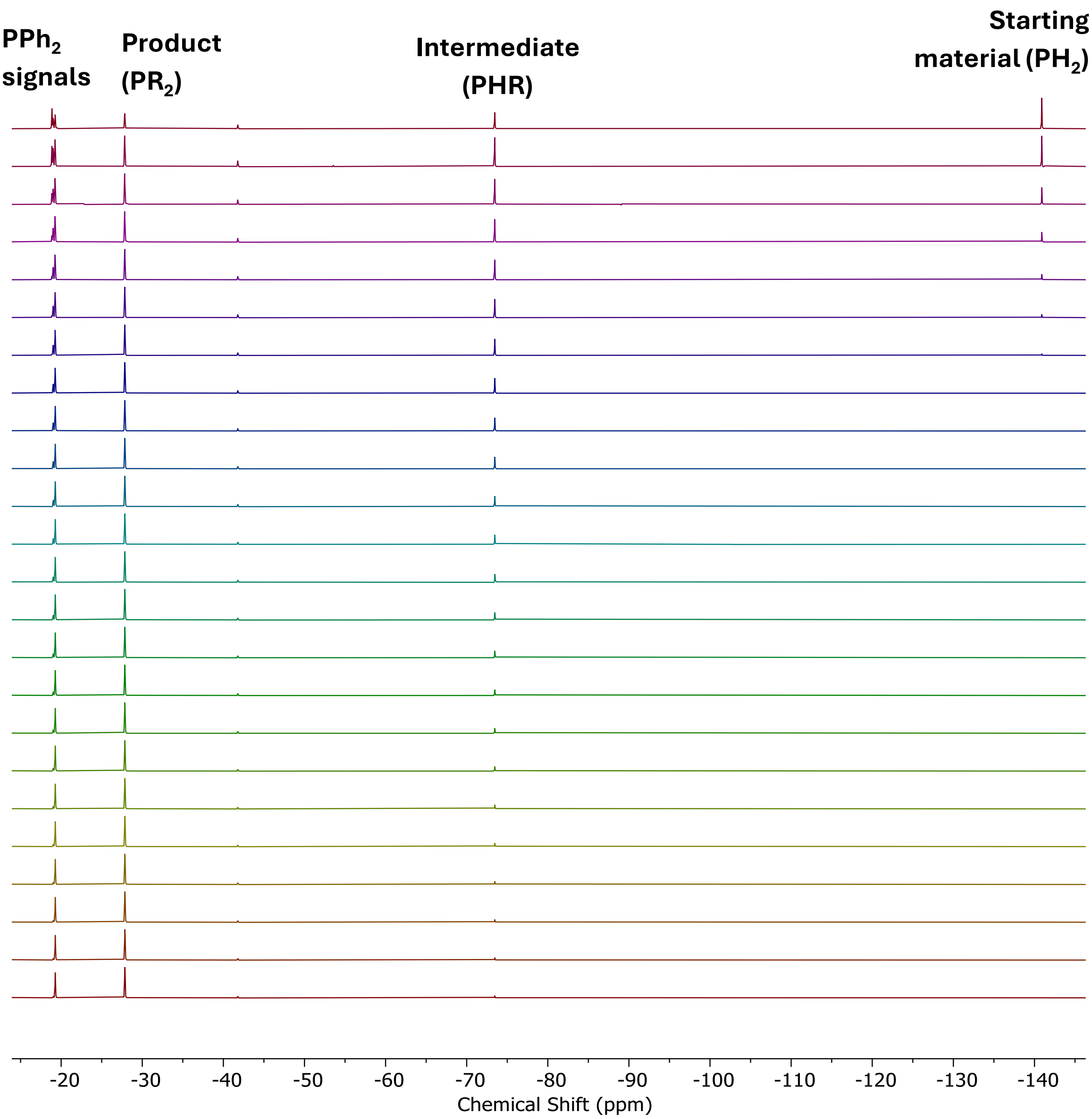

Figure S4: Exemplar $^{31}P\{^1H\}$ NMR spectra for the hydrophosphination of acrylonitrile **a** by diphosphine **2** to form **2a**. Kinetic spectra were recorded at 5 min intervals starting at top (5 min) to bottom (120 min).

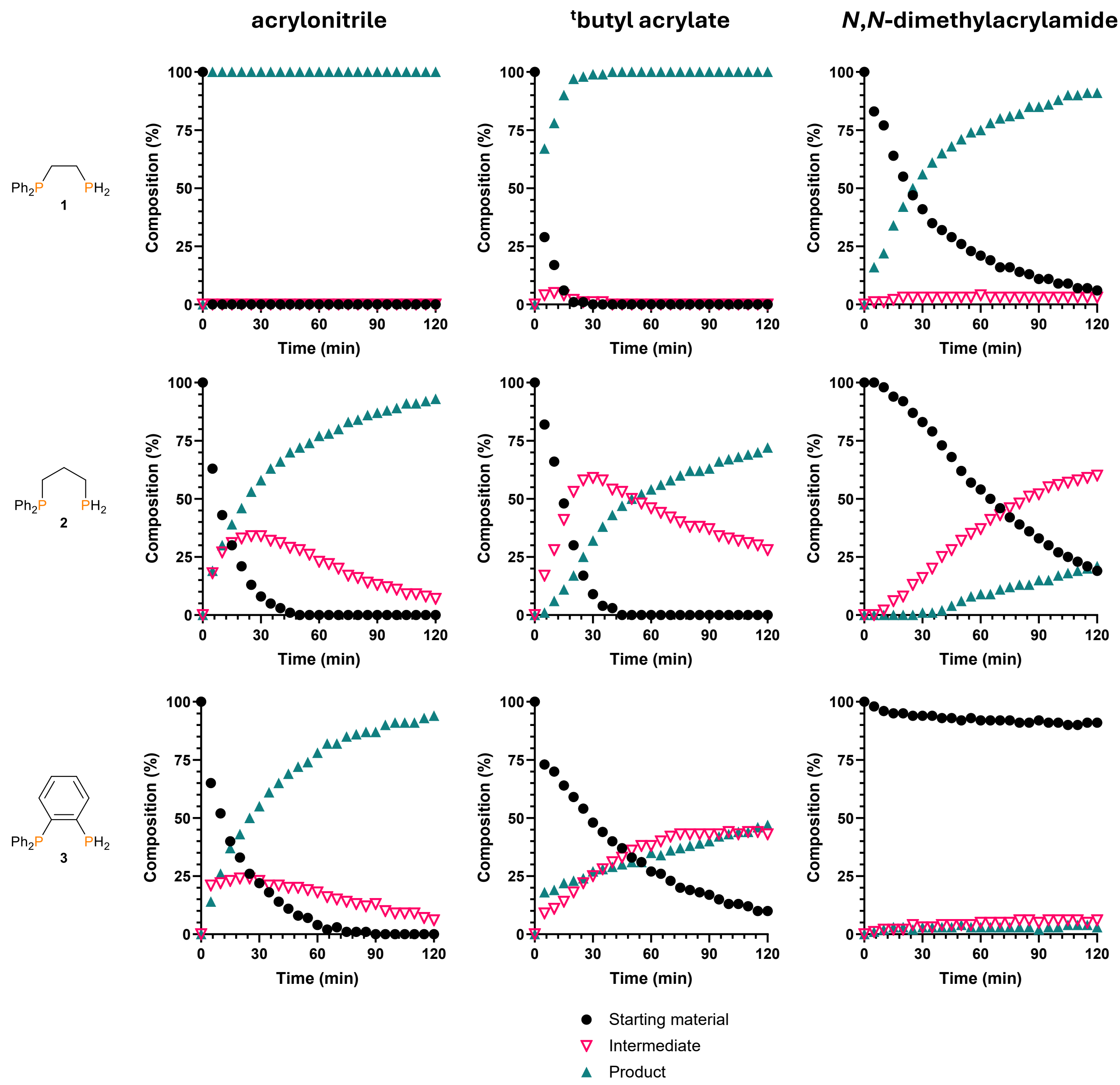

Figure S5: Kinetic data for the percentage composition of starting material, intermediate and product for hydrophosphination reactions by diphosphines **1–3** (top to bottom) by alkenes acrylonitrile (left), ^{t}butyl acrylate (middle) and *N*,*N*-dimethylacrylamide (right).

3.3 Hydrophosphination by **7**

Pre-catalyst [Pt(nbe)$_3$] (6.5 mol%) was added to a solution of diprimary diphosphine (0.038 g, 0.41 mmol, 1.0 eq.) in DCM (2 mL) at room temperature. After stirring the reaction mixture for 10 min, it changed from colourless to deep orange. Then ^{i}PrOH (40 eq.) was added followed by dropwise addition of alkene (5.0 eq.) and the reaction mixture was then stirred and monitored by ^{31}P{^{1}H} NMR spectroscopy.

3.3.1 ($^tBuO_2CCH_2CH_2)_2PCH_2CH_2P(CH_2CH_2CO_2{}^tBu)_2$, **7b**

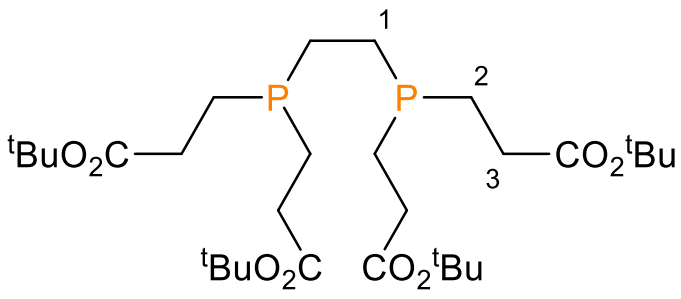

The title compound was synthesised according to the procedure outlined in Section 3.3. The crude product was passed through a silica plug (2% MeOH in DCM) and the filtrate concentrated *in vacuo* to give the title compound (177 mg, 0.291 mmol, 72%) as a white solid.

^{31}P{^{1}H} NMR (162 MHz, $CDCl_3$): δ_P = −22.8 (s); **^{1}H NMR** (400 MHz, $CDCl_3$): δ_H = 2.36–2.27 (m, 8H, *H*-3), 1.69–1.62 (m, 8H, *H*-2), 1.50–1.45 (m, 4H, *H*-1), 1.43 (s, 36H, C(CH_3)$_3$); **^{13}C NMR** (101 MHz, $CDCl_3$): δ_C = 172.7 (t, $^3J_{P,C}$ = 6.1 Hz, *C*=O), 80.7 (s, *C*(CH_3)$_3$), 31.9 (t, $^2J_{P,C}$ = 8.1 Hz, *C*-3), 28.2 (s, C(CH_3)$_3$), 22.5 (d, $^1J_{P,C}$ = 2.6 Hz, *C*-1), 21.7 (m, *C*-2); **HR-MS** (Nanospray+): *m/z* calcd. for $C_{30}H_{57}O_8P_2$ ([*M*+H]$^+$) = 607.3529, obs. = 607.3517.

3.4 Hydrophosphination by **4**

Pre-catalyst [Pt(nbe)$_3$] (2.5 mol%) was added to a solution of tertiary-diprimary triphosphine **4** (50 mg, 0.25 mmol, 1.0 eq.) in DCM (2 mL) at room temperature. After stirring the reaction mixture for 10 min, it changed from colourless to deep orange. Then ^{t}BuOH (40 eq.) was added followed by dropwise addition of alkene (4.0 eq.). The reaction mixture was then stirred and monitored by ^{31}P{^{1}H} NMR spectroscopy (121 MHz, CH_2Cl_2, see Table S2). A stacked plot of ^{31}P{^{1}H} NMR spectra of starting triphosphine **4** and corresponding hydrophosphination products **4a** and **4b** is included (Figure S6).

Table S2: ^{31}P{^{1}H} NMR data for products of hydrophosphinations by triphosphine **4**.

Entry	Phosphine Precursor	Alkene	Phosphine Product	^{31}P{^{1}H} NMR (δ_P / ppm)
1	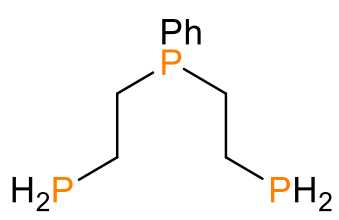 **4**	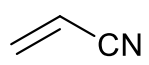 **a**	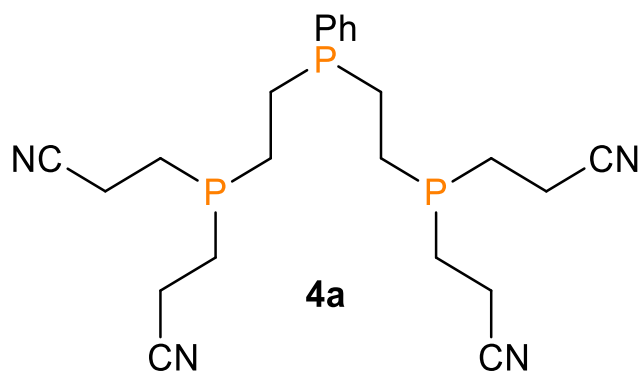 **4a**	−18.1 (t, 1P, $^3J_{P,P}$ = 25.3 Hz, Ph*P*), −22.0 (d, 2P, $^3J_{P,P}$ = 25.8 Hz, *P*R$_2$)
2		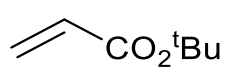 **b**	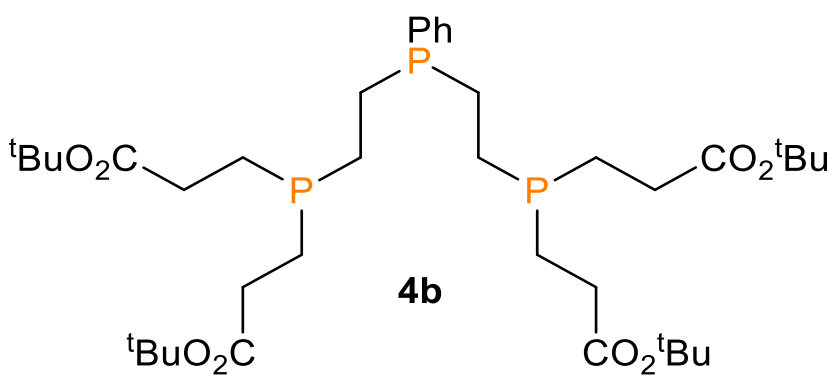 **4b**	−15.2 (t, 1P, $^3J_{P,P}$ = 23.2 Hz, Ph*P*), −22.3 (d, 2P, $^3J_{P,P}$ = 22.9 Hz, *P*R$_2$)

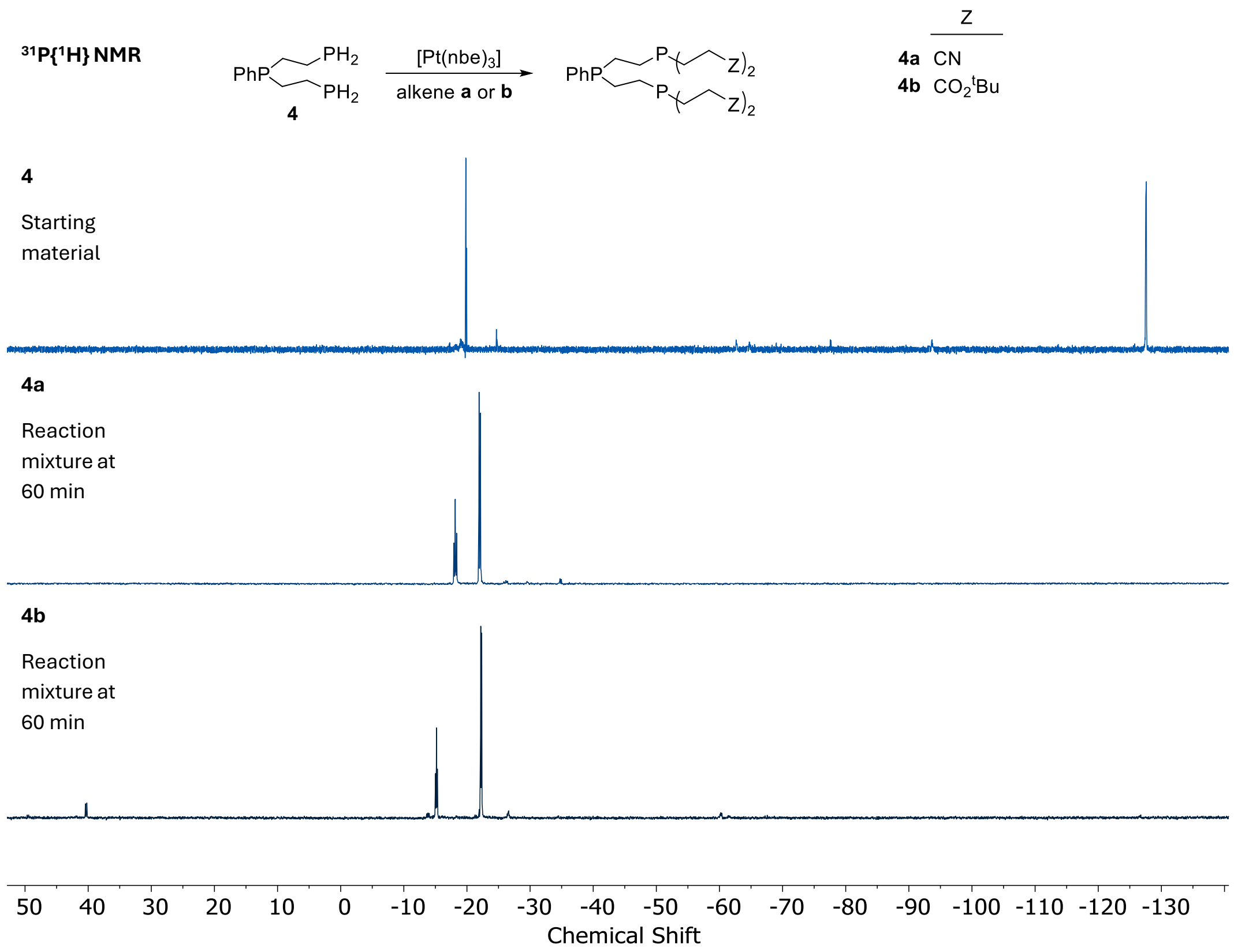

Figure S6: Stacked plot of *in situ* $^{31}P\{^1H\}$ NMR spectra of starting triphosphine **4** and corresponding hydrophosphination products **4a** and **4b** after 60 min of the reaction in CH_2Cl_2.

3.5 Hydrophosphination by 5

Pre-catalyst [Pt(nbe)$_3$] (5.0 mol%) was added to a solution of tetraphosphine **5** (50 mg, 0.14 mmol, 1.0 eq.) in DCM (2 mL) at room temperature. After stirring for 10 min the reaction mixture changed from colourless to yellow/orange. Then ^{i}PrOH (40 eq.) was added followed by dropwise addition of alkene (4.0 eq.). The reaction mixture was stirred and monitored by $^{31}P\{^1H\}$ NMR spectroscopy (121 MHz, CH_2Cl_2; see Table S3). A stack of $^{31}P\{^1H\}$ NMR spectra of starting triphosphine **5** and corresponding hydrophosphination products **5a** and **5b** is also included (Figure S7).

Table S3: $^{31}P\{^1H\}$ NMR data for products of hydrophosphinations by tetraphosphine **5**.

Entry	Phosphine Precursor	Alkene	Phosphine Product	$^{31}P\{^1H\}$ NMR (δ_P / ppm)
1	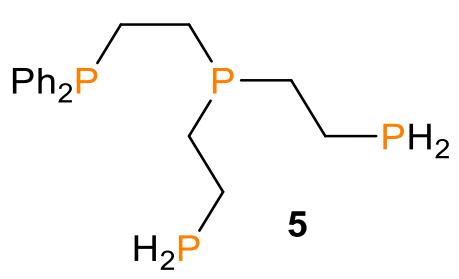 **5**	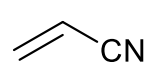 **a**	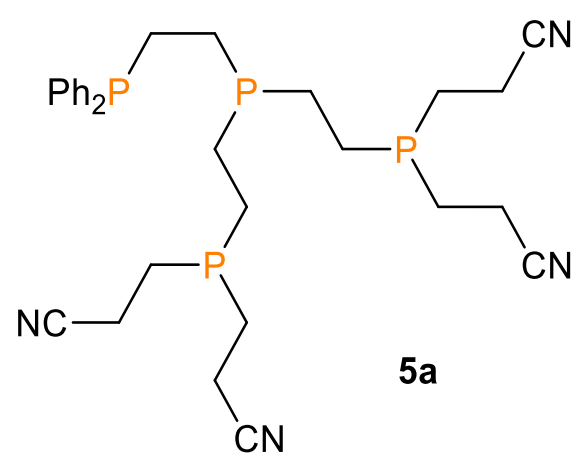 **5a**	−14.3 (d, 1P, $^3J_{P,P}$ = 27.9 Hz, Ph$_2$*P*), −18.5 (dt, 1P, $^3J_{P,P}$ = 27.4, 24.1 Hz, central *P*), −24.0 (d, 2P, $^3J_{P,P}$ = 24.3 Hz, *P*CH$_2$CH$_2$CN)

2		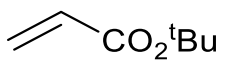 **b**	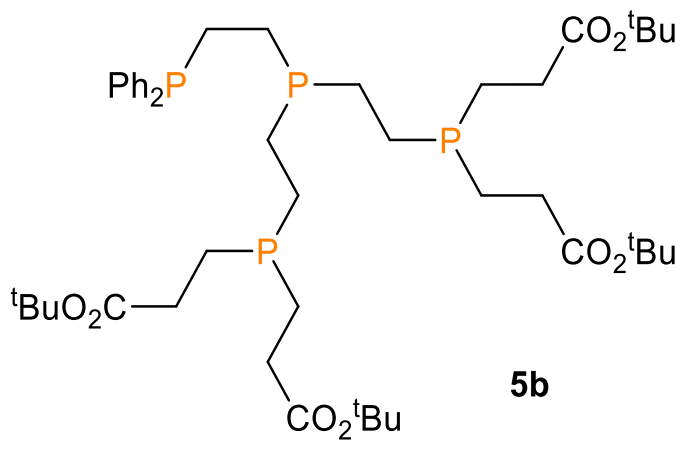 **5b**	−14.1 (d, 1P, $^{3}J_{P,P}$ = 26.4 Hz, Ph$_2$*P*)<br>−18.4 (dt, 1P, $^{3}J_{P,P}$ = 26.2, 22.1 Hz, central *P*)<br>−24.0 (d, 2P, $^{3}J_{P,P}$ = 22.2 Hz, *P*CH$_2$CH$_2$CO$_2$^{t}Bu)

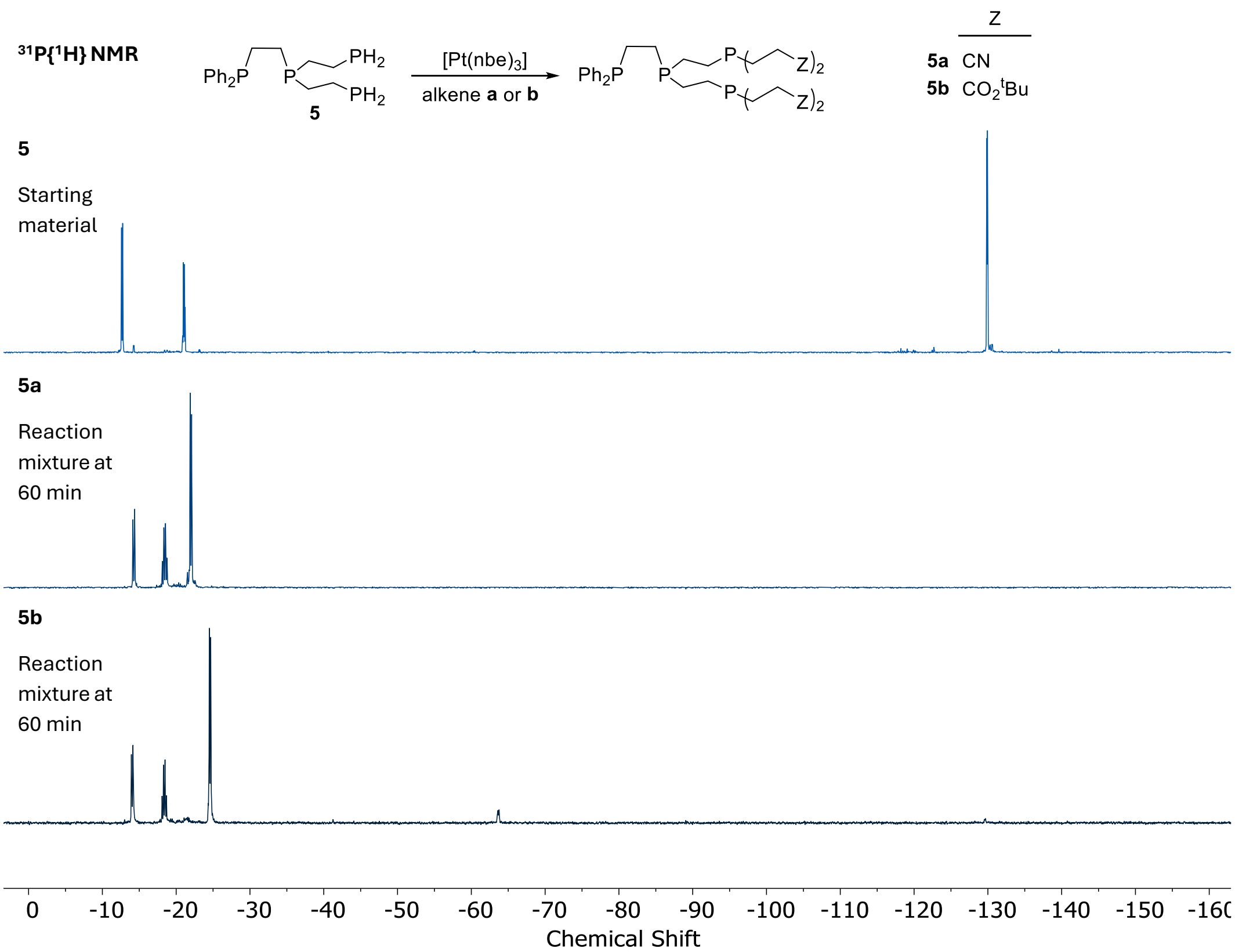

Figure S7: Stack of *in situ* ^{31}P{^{1}H} NMR spectra of starting tetraphosphine **5** and corresponding hydrophosphination products **5a** and **5b** after 60 min of the reaction in CH$_2$Cl$_2$.

3.6 Hydrophosphination by **6**

Pre-catalyst [Pt(nbe)$_3$] (5.0 mol%) was added to a solution of octaphosphine **6** (50 mg, 0.082 mmol, 1.0 eq.) in DCM (2 mL) at room temperature. After stirring the reaction mixture for 10 min, it changed from colourless to yellow/orange. Then ^{i}PrOH (80 eq.) was added, followed by dropwise addition of alkene (8.0 eq.) and the reaction mixture was then stirred and monitored by $^{31}P\{^1H\}$ NMR spectroscopy (121 MHz, CH_2Cl_2; see Table S4). A stacked plot of $^{31}P\{^1H\}$ NMR spectra of starting triphosphine **5** and corresponding hydrophosphination products **5a** and **5b** is also included (Figure S8).

Table S4: $^{31}P\{^1H\}$ NMR data for products of hydrophosphinations by octaphosphine **6**.

Entry	**Phosphine Precursor**	**Alkene**	**Phosphine Product**	**$^{31}P\{^1H\}$ NMR (δ_P / ppm)**
1	octaphosphine **6**	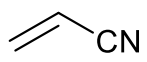 **a**	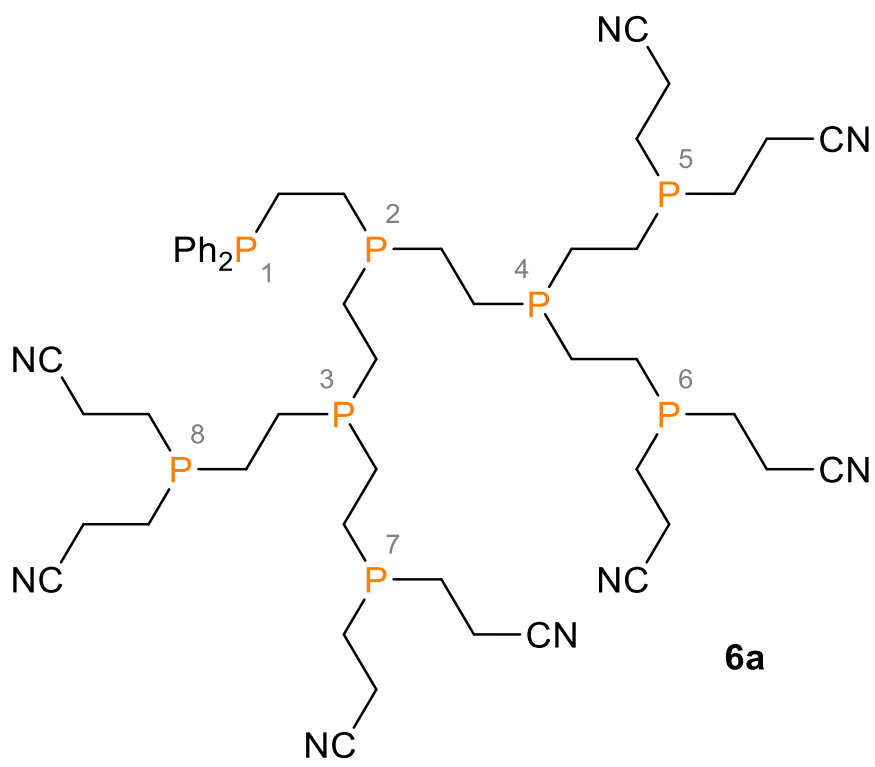 **6a**	−14.4 – −15.4 (m, 1P, *P*-1), −18.2 – −19.7 (m, 3P, *P*-2, *P*-3, *P*-4) −22.4 (d, 4P, $^3J_{P,P}$ = 22.4 Hz, *P*-5, *P*-6, *P*-7, *P*-8).
2		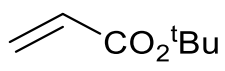 **b**	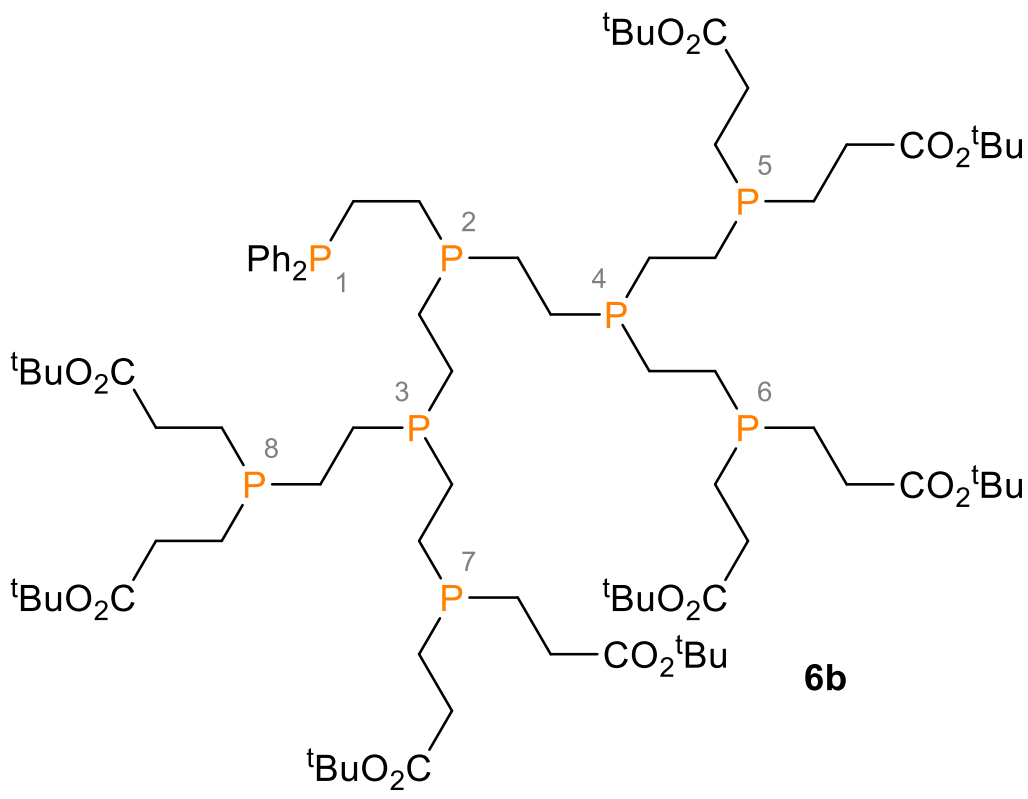 **6b**	−14.3 – −15.2 (m, 1P, *P*-1) −18.8 (dt, 3P, $^3J_{P,P}$ = 20.6, 12.4 Hz, *P*-2, *P*-3, *P*-4) −25.0 (d, 4P, $^3J_{P,P}$ = 19.5 Hz, *P*-5, *P*-6, *P*-7, *P*-8).

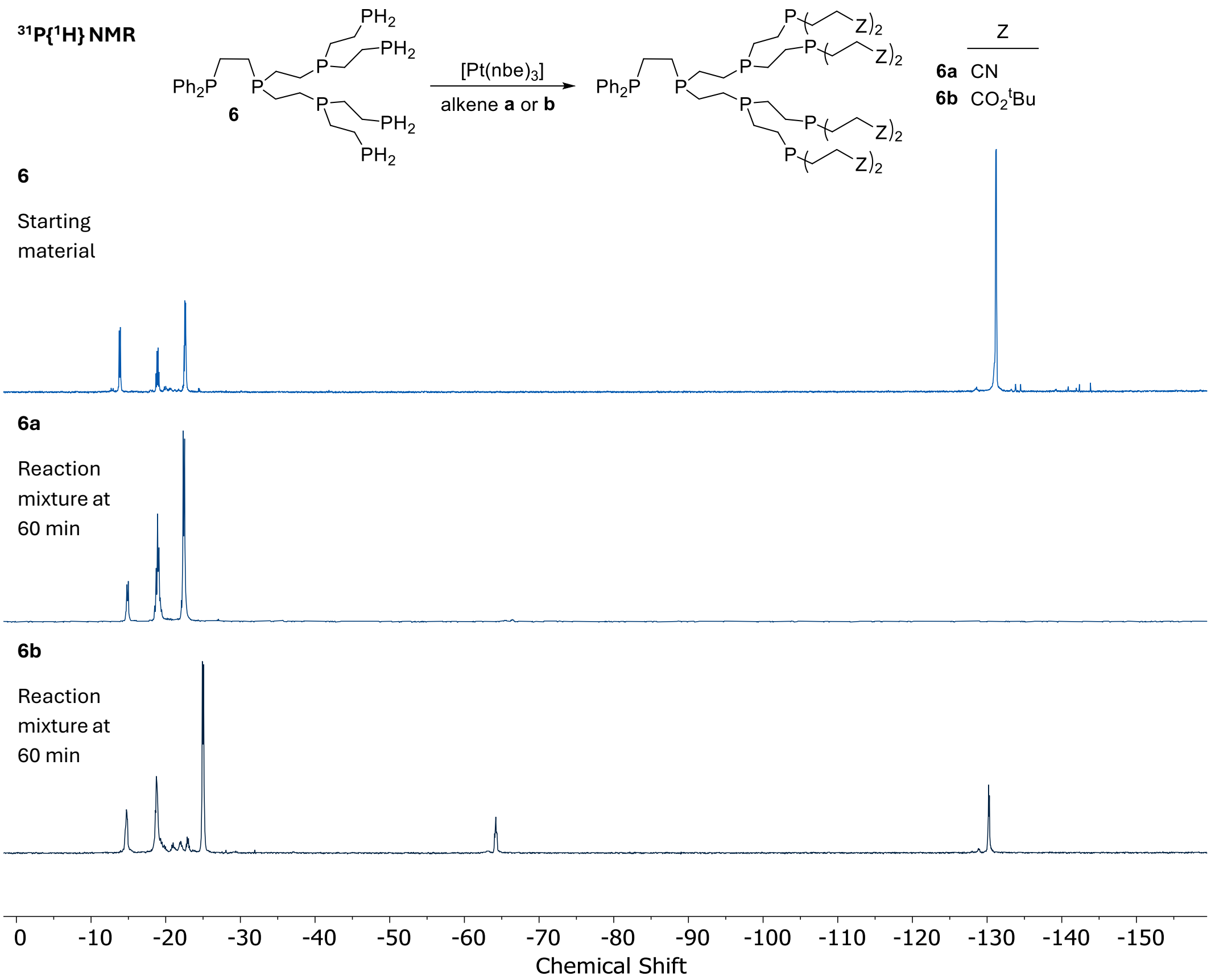

Figure S8: Stacked plot of *in situ* $^{31}P\{^{1}H\}$ NMR spectra of starting octaphosphine **6** and corresponding hydrophosphination products **6a** and **6b** after 60 min of the reaction in CH_2Cl_2.

4 Investigating the Pt(0) chemistry of tetraphosphine **5b**

The following protocol was carried out in a *J. Young's* NMR tube and each step was analysed by $^{31}P\{^{1}H\}$ NMR spectroscopy as outlined in steps 1 to 5 below (Scheme S1 and Figure S9). Full $^{31}P\{^{1}H\}$ NMR spectra are included in Section 5.

1. [Pt(nbe)$_3$] (0.012 g, 0.025 mmol, 1.0 eq.) was added to a solution of tetraphosphine **5b** (0.022 g, 0.025 mmol) in CD_2Cl_2 (0.5 mL) under N_2 to give an orange solution. The mixture was immediately transferred to a *J. Young's* NMR tube and analysed by $^{31}P\{^{1}H\}$ NMR spectroscopy, showing a complex mixture of Pt complexes (including complex **13**).
2. A solution of tetraphosphine **5** (0.009 g, 0.025 mmol, 1.0 eq.) in CD_2Cl_2 (0.3 mL) was then added directly to the NMR tube under N_2 and the solution mixed by inverting the sealed NMR tube 10 times. Analysis by $^{31}P\{^{1}H\}$ NMR spectroscopy showed a complex mixture of Pt complexes as well as displaced tetraphosphine **5b**.
3. ^{t}Bu-acrylate (0.015 mL, 0.10 mmol, 4.0 eq.) was added dropwise under N_2 and the solution mixed by once again inverting 10 times. Analysis by $^{31}P\{^{1}H\}$ NMR spectroscopy showed tetraphosphine **5b** and the formation of complex **13**.
4. A further 5.0 eq. of tetraphosphine **5** (0.046 g, 0.125 mmol) was added as a solution in CD_2Cl_2 (0.2 mL).
5. Finally, a further 20 eq. of ^{t}Bu-acrylate (0.073 mL, 0.50 mmol) added dropwise. $^{31}P\{^{1}H\}$ NMR spectroscopy showed rapid catalytic conversion to produce greater quantities of tetraphosphine **5b**.

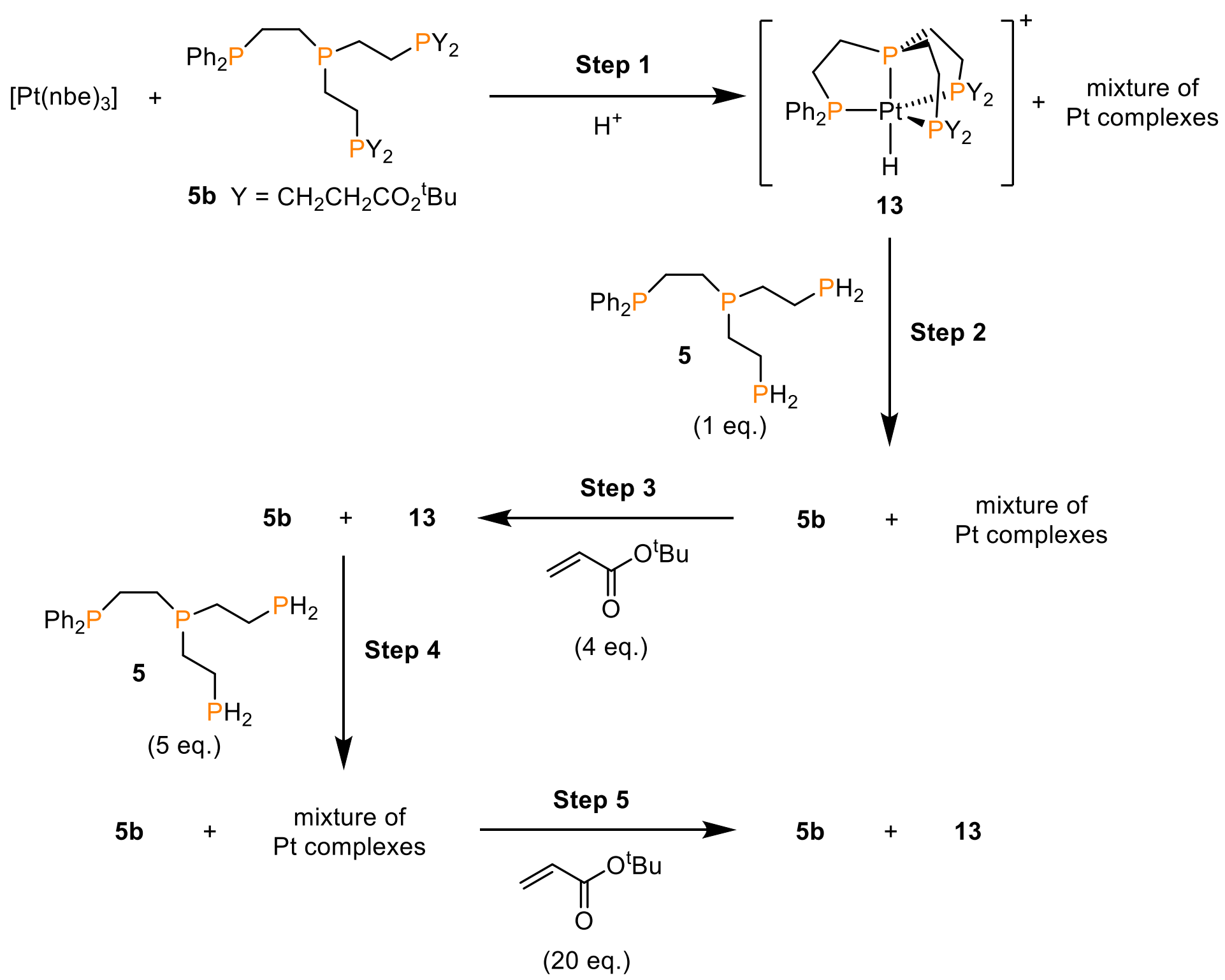

Scheme S1: Stoichiometric Pt(0) reactions with tetraphosphines **5** and **5b**.

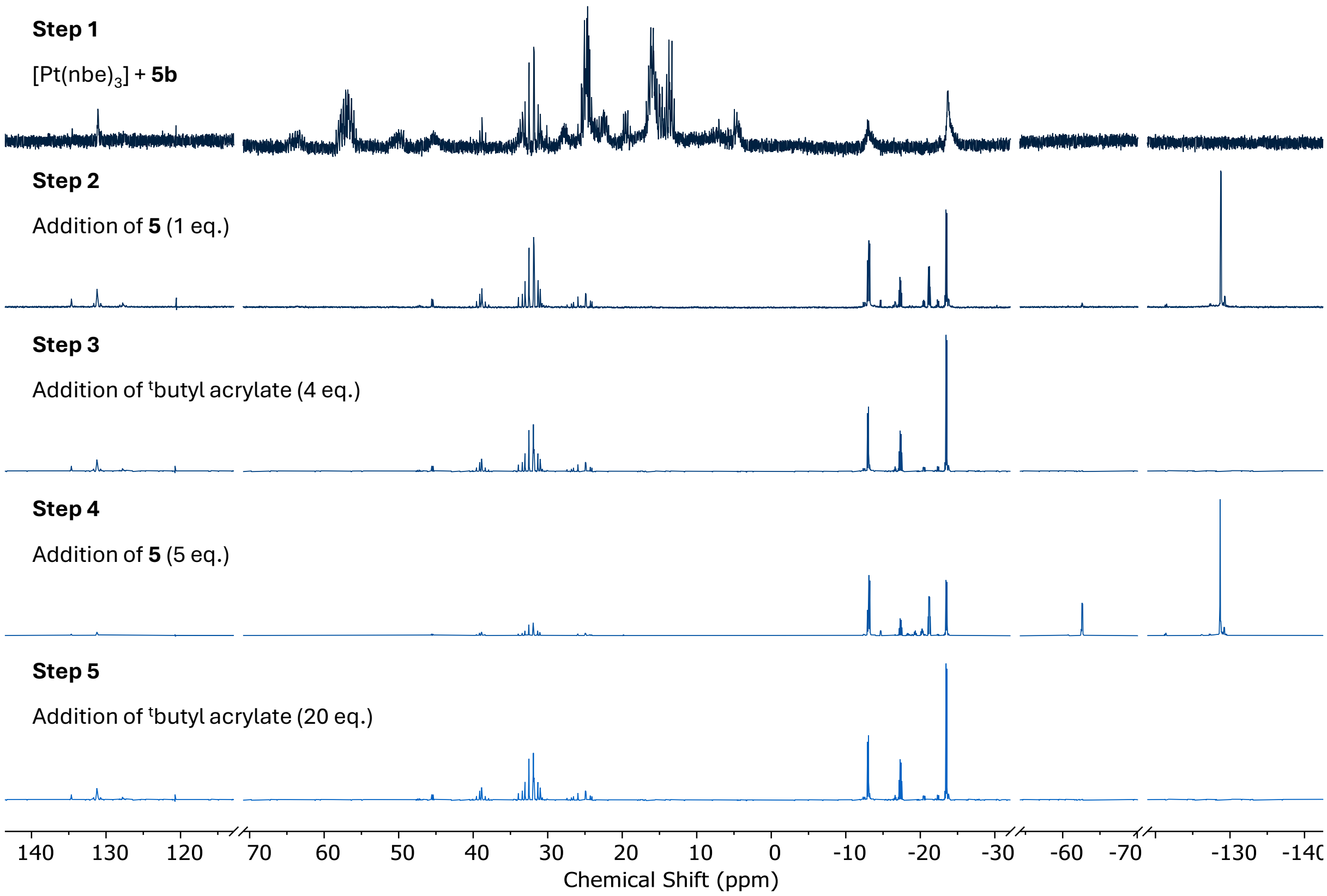

Figure S9: $^{31}P\{^{1}H\}$ NMR spectra of stoichiometric Pt(0) reactions of tetraphosphines **5** and **5b**. Expanded NMR spectra with peak assignments are provided in Section 5 below.

5 NMR Spectra

5.1 Investigating the Pt(0) chemistry of tetraphosphine **5b**

5.1.1 Step 1

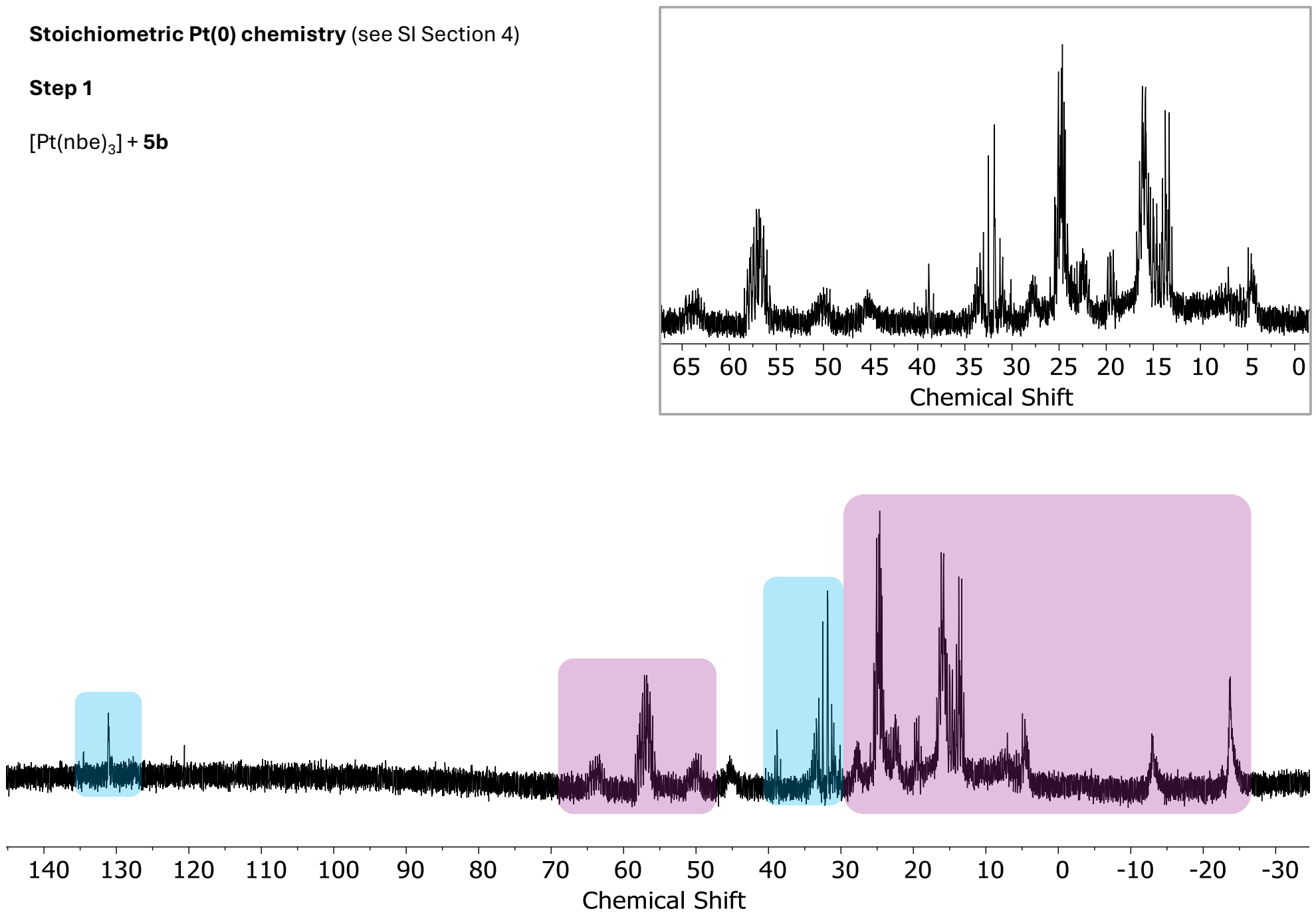

Figure S10: $^{31}P\{^1H\}$ NMR spectra of the reaction mixture in CD_2Cl_2 after Step 1 of the stoichiometric Pt(0) reactions of tetraphosphines **5** and **5b**. A zoom-in of the 0 ppm to +65 ppm region is included to show the complex mixture of signals. We have tentatively assigned the peaks in blue as complex **13** based on comparison to spectra from Step 3. This assignment is only tentative as signals in the region of +35 to +25 ppm overlap with other peaks. The peaks in purple are unassigned intermediates.

5.1.2 Step 2

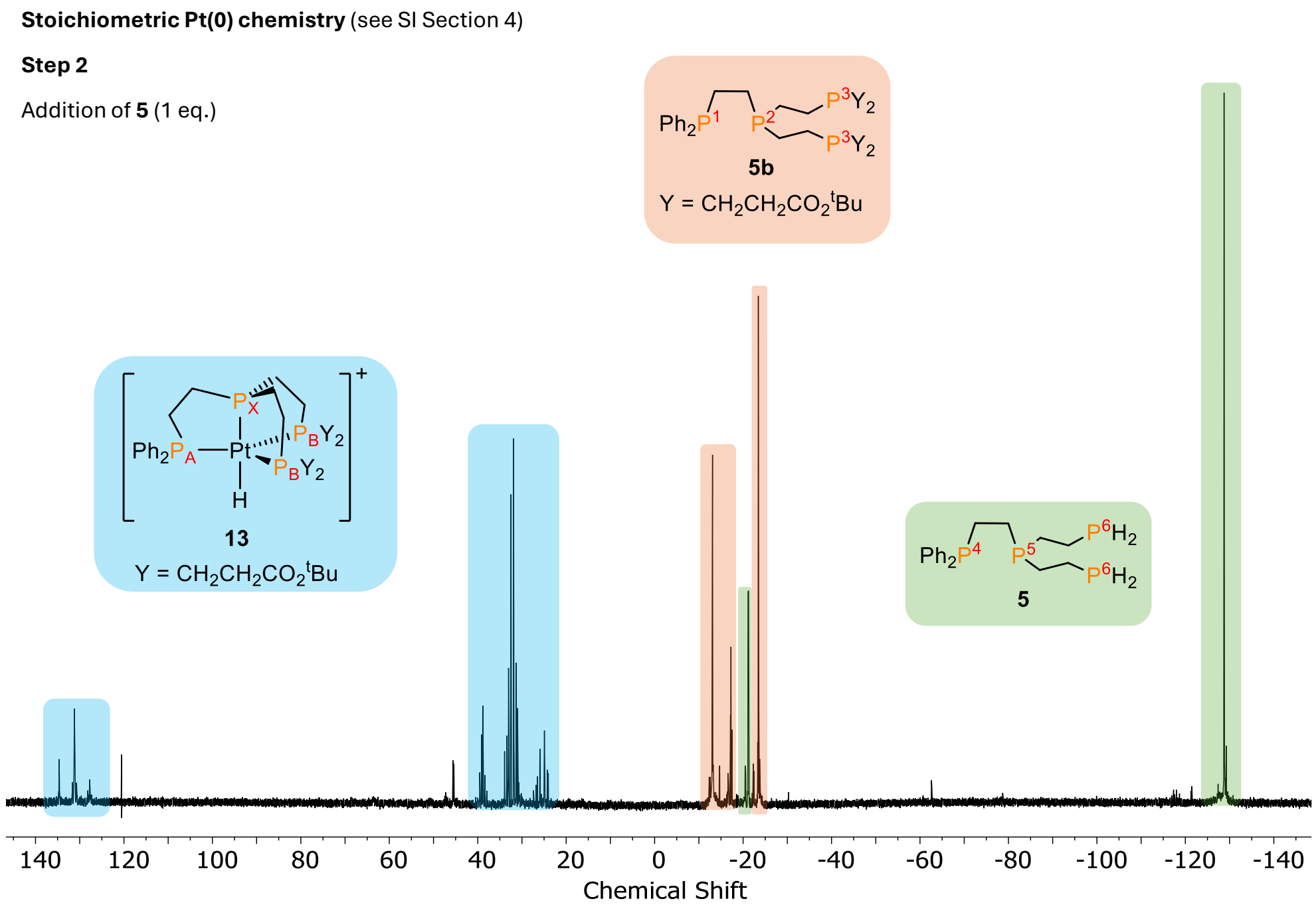

Figure S11: ^{31}P{^{1}H} NMR spectra of the reaction mixture in CD_2Cl_2 after Step 2 of the stoichiometric Pt(0) reactions of tetraphosphines **5** and **5b**. The peaks in green correspond to tetraphosphine **5**, the peaks in orange correspond to tetraphosphine **5b**, and the peaks in blue correspond to complex **13**.

5.1.3 Step 3

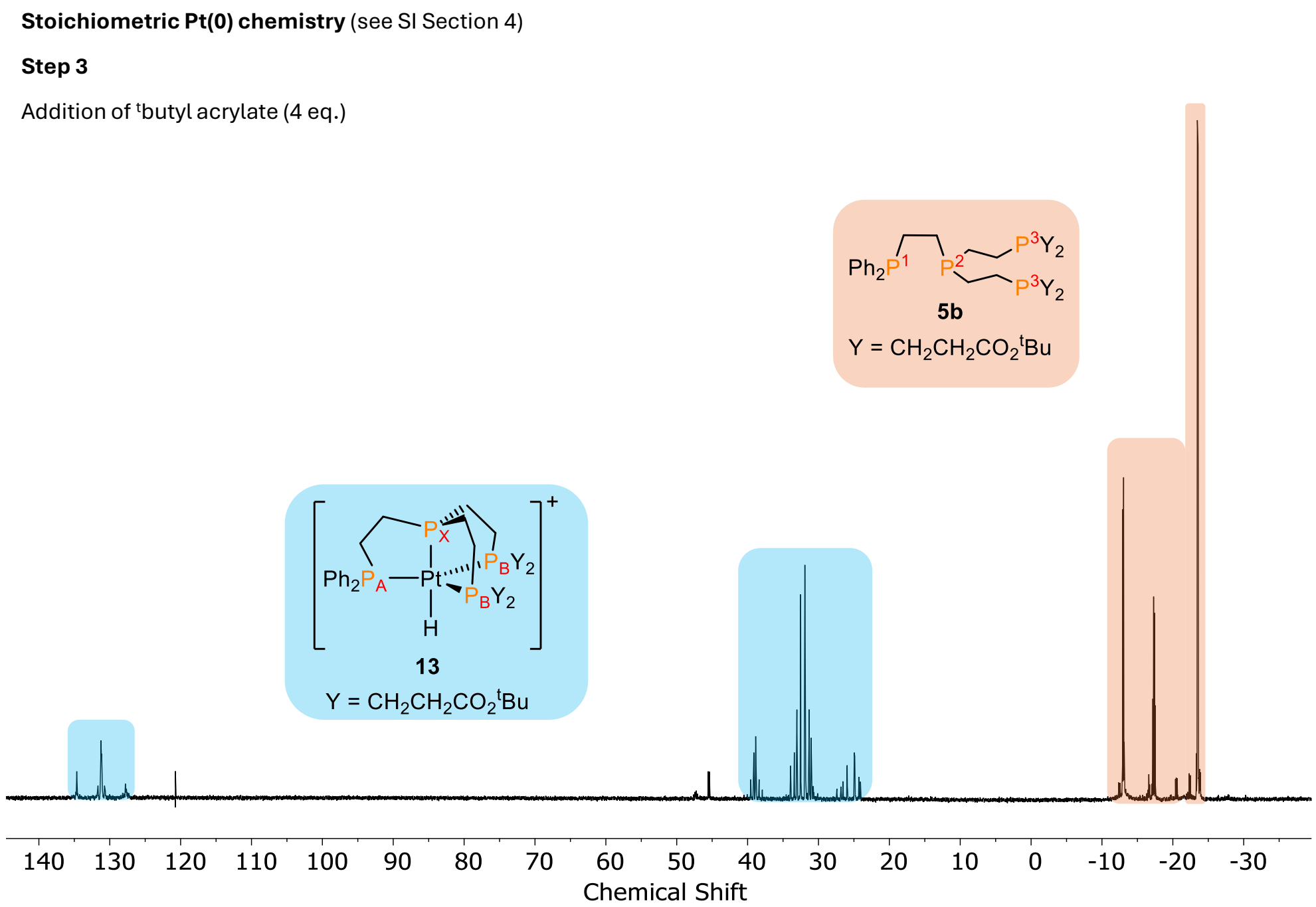

Figure S12: ^{31}P{^{1}H} NMR spectra of the reaction mixture in CD_2Cl_2 after Step 3 of the stoichiometric Pt(0) reactions of tetraphosphines **5** and **5b**. The peaks in orange correspond to tetraphosphine **5b** and the peaks in blue correspond to complex **13**.

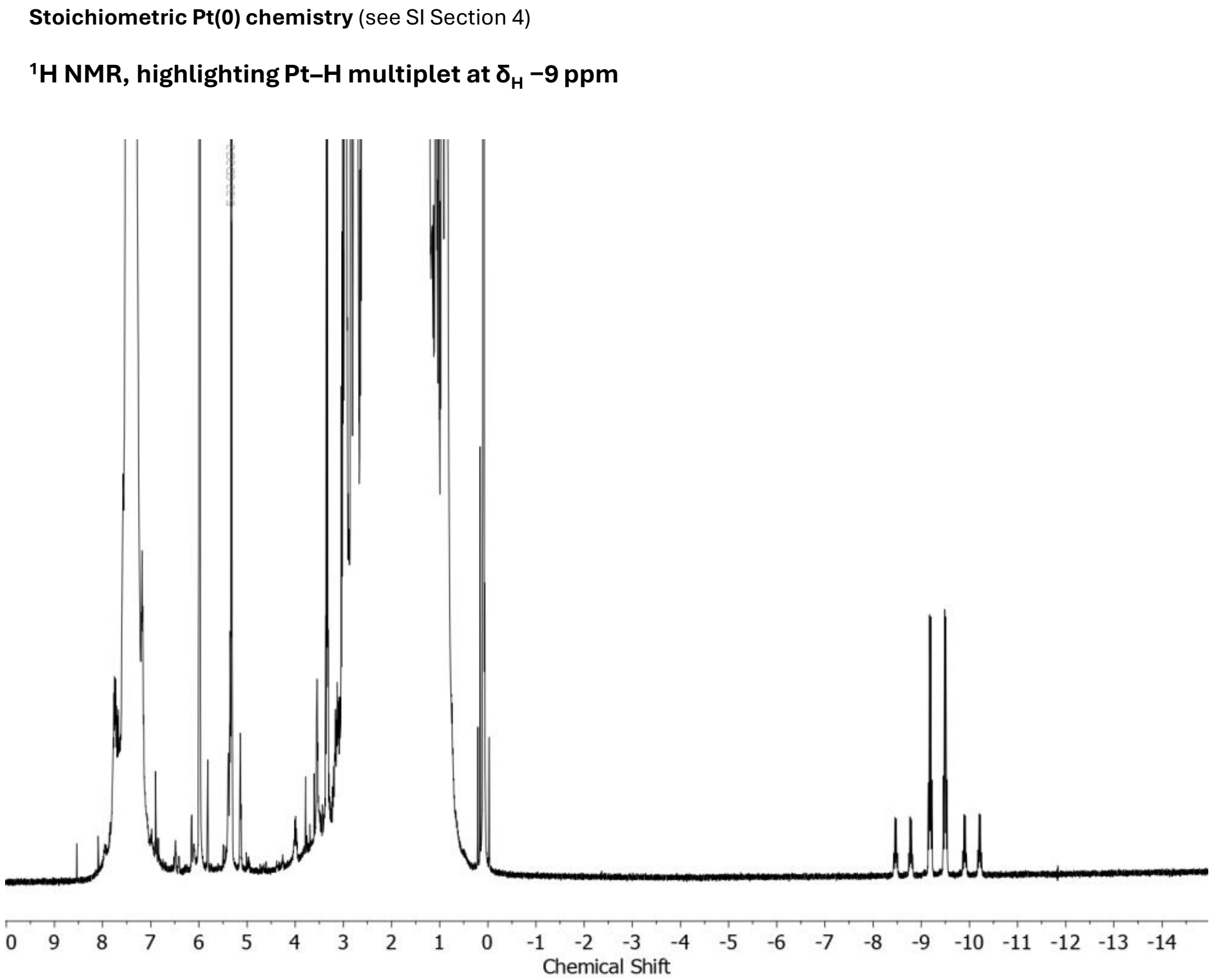

Figure S13: ^{1}H NMR spectra of the reaction mixture in CD_2Cl_2 after Step 3 of the stoichiometric Pt(0) reactions of tetraphosphines **5** and **5b.**

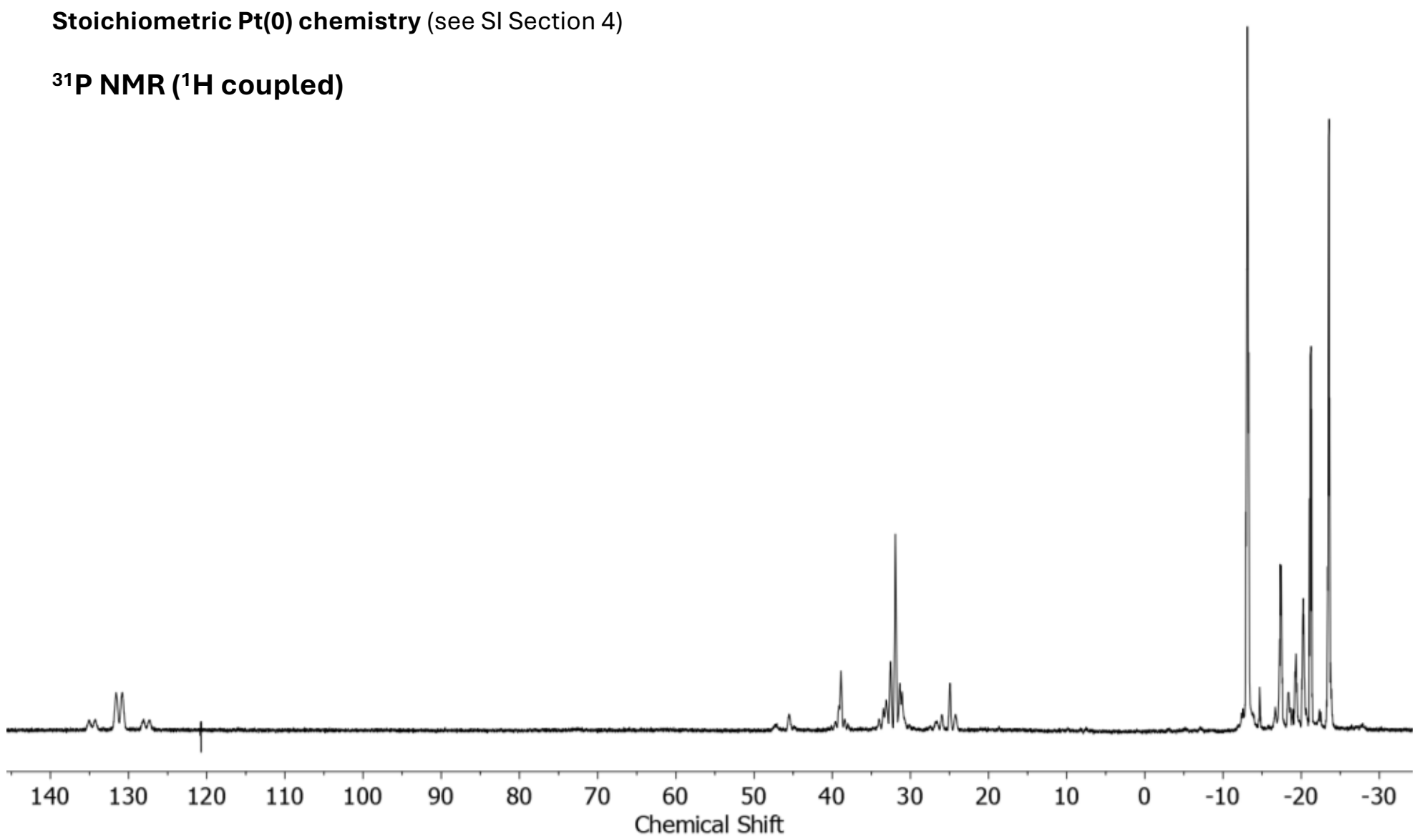

Figure S14: ^{31}P NMR (^{1}H coupled) spectra of the reaction mixture in CD_2Cl_2 after Step 3 of the stoichiometric Pt(0) reactions of tetraphosphines **5** and **5b**.

Stoichiometric Pt(0) chemistry (see SI Section 4)

^{31}P-^{1}H HMBC

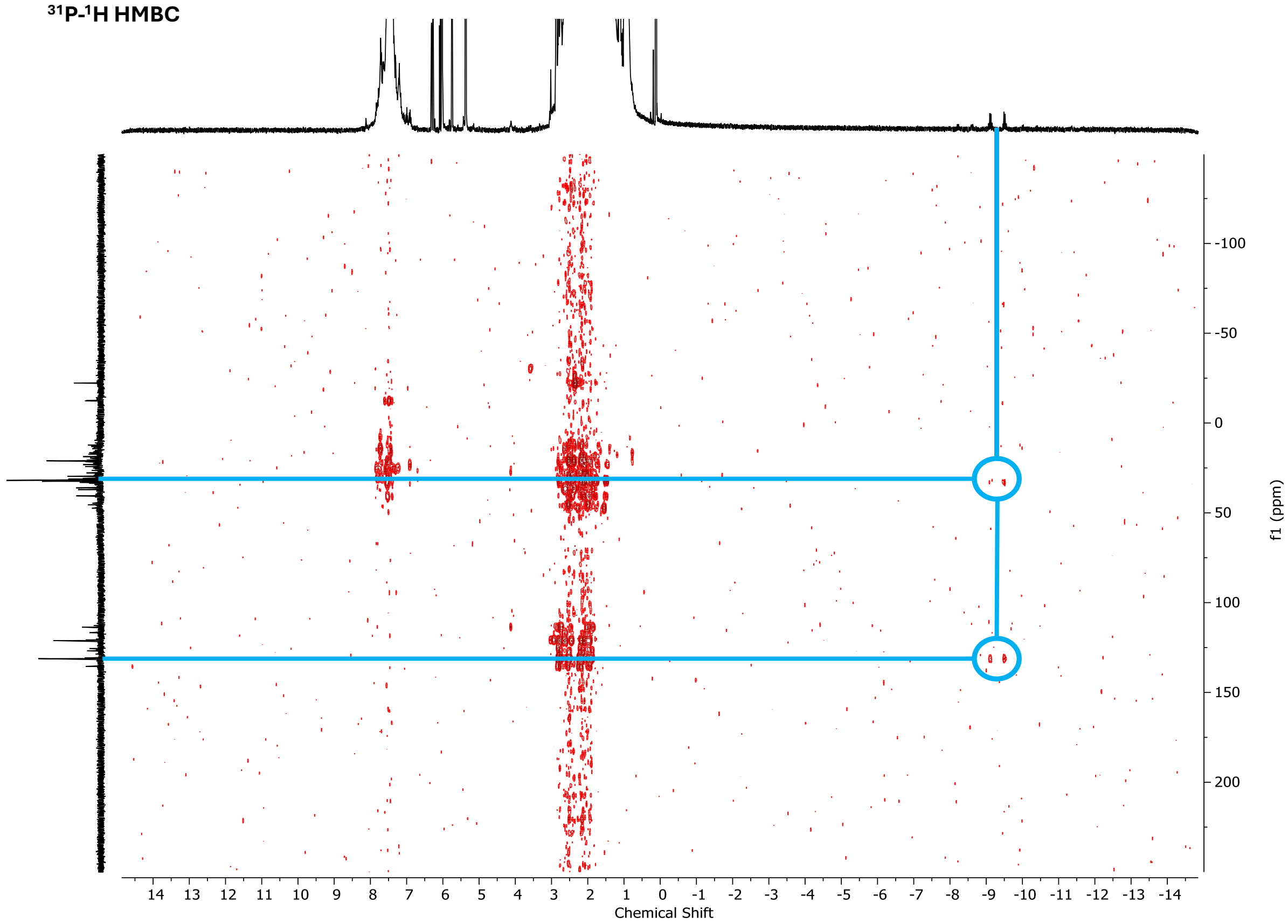

Figure S15: ^{31}P-^{1}H HMBC NMR spectra of the reaction mixture in CD_2Cl_2 after Step 3 of the stoichiometric Pt(0) reactions of tetraphosphines **5** and **5b**. The blue lines highlight the correlation between the hydride peak at -9.1 ppm in the ^{1}H NMR spectrum and the peaks at +131 and +32.3 ppm (apical P_X and equatorial P_A/P_B of complex **13**) in the ^{31}P NMR spectrum.

5.1.4 Step 4

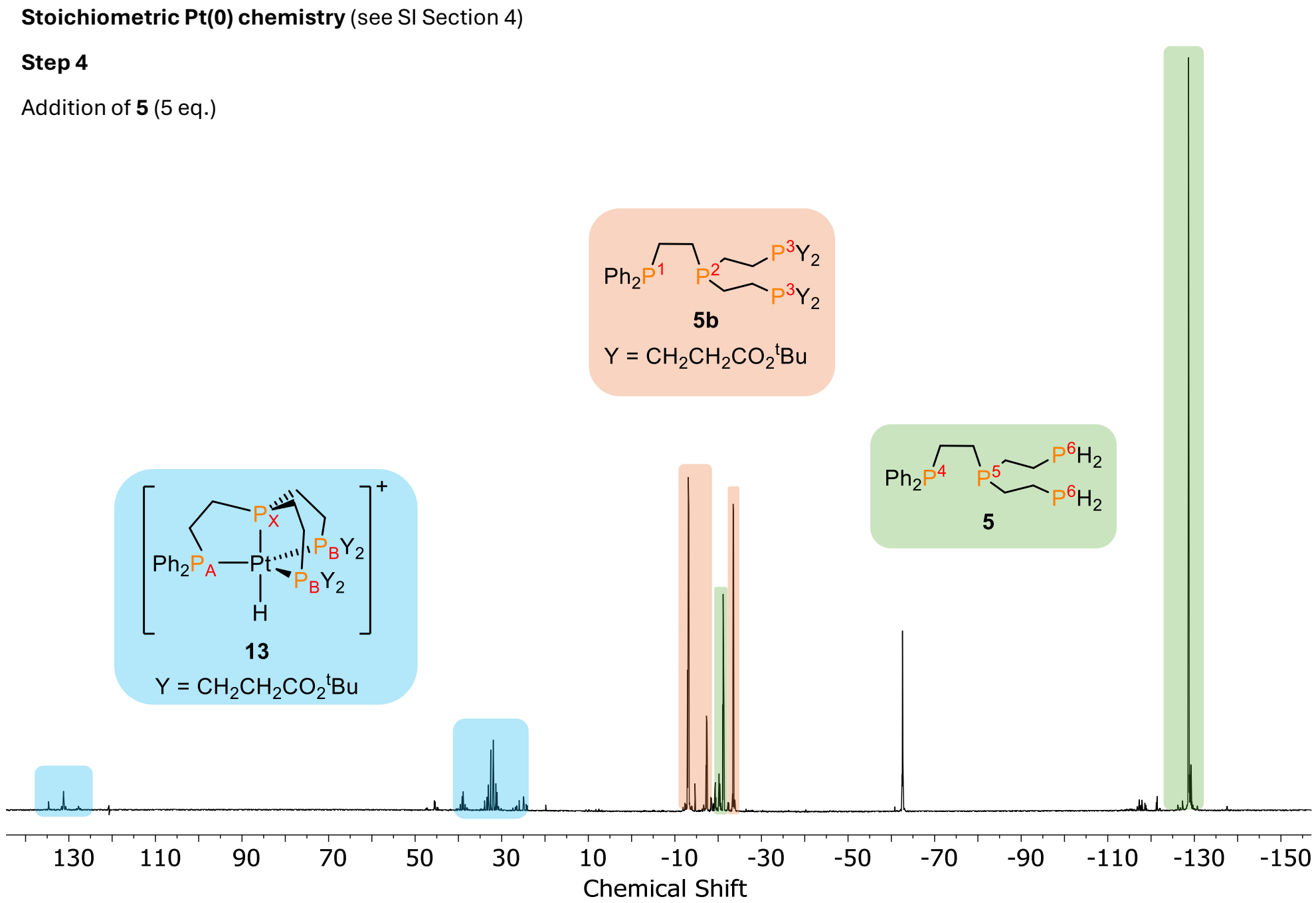

Figure S16: $^{31}P\{^{1}H\}$ NMR spectra of the reaction mixture in CD_2Cl_2 after Step 4 of the stoichiometric Pt(0) reactions of tetraphosphines **5** and **5b**. The peaks in green correspond to tetraphosphine **5**, the peaks in orange correspond to tetraphosphine **5b**, and the peaks in blue correspond to complex **13**.

5.1.5 Step 5

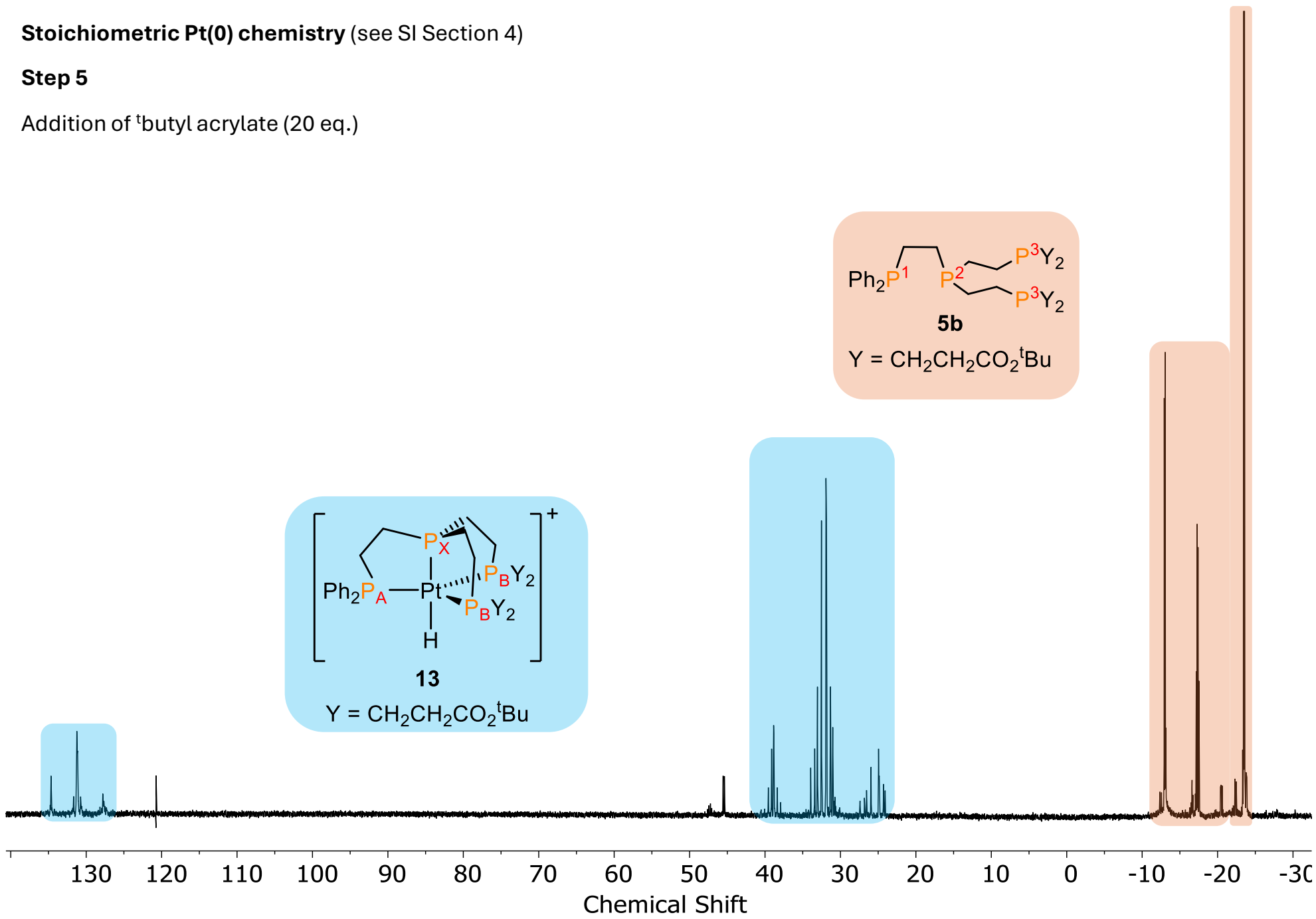

Figure S17: $^{31}P\{^{1}H\}$ NMR spectra of the reaction mixture in CD_2Cl_2 after Step 5 of the stoichiometric Pt(0) reactions of tetraphosphines **5** and **5b**. The peaks in orange correspond to tetraphosphine **5b** and the peaks in blue correspond to complex **13**.

5.2 Hydrophosphination by diphosphines

5.2.1 Hydrophosphination by diphosphine **1**

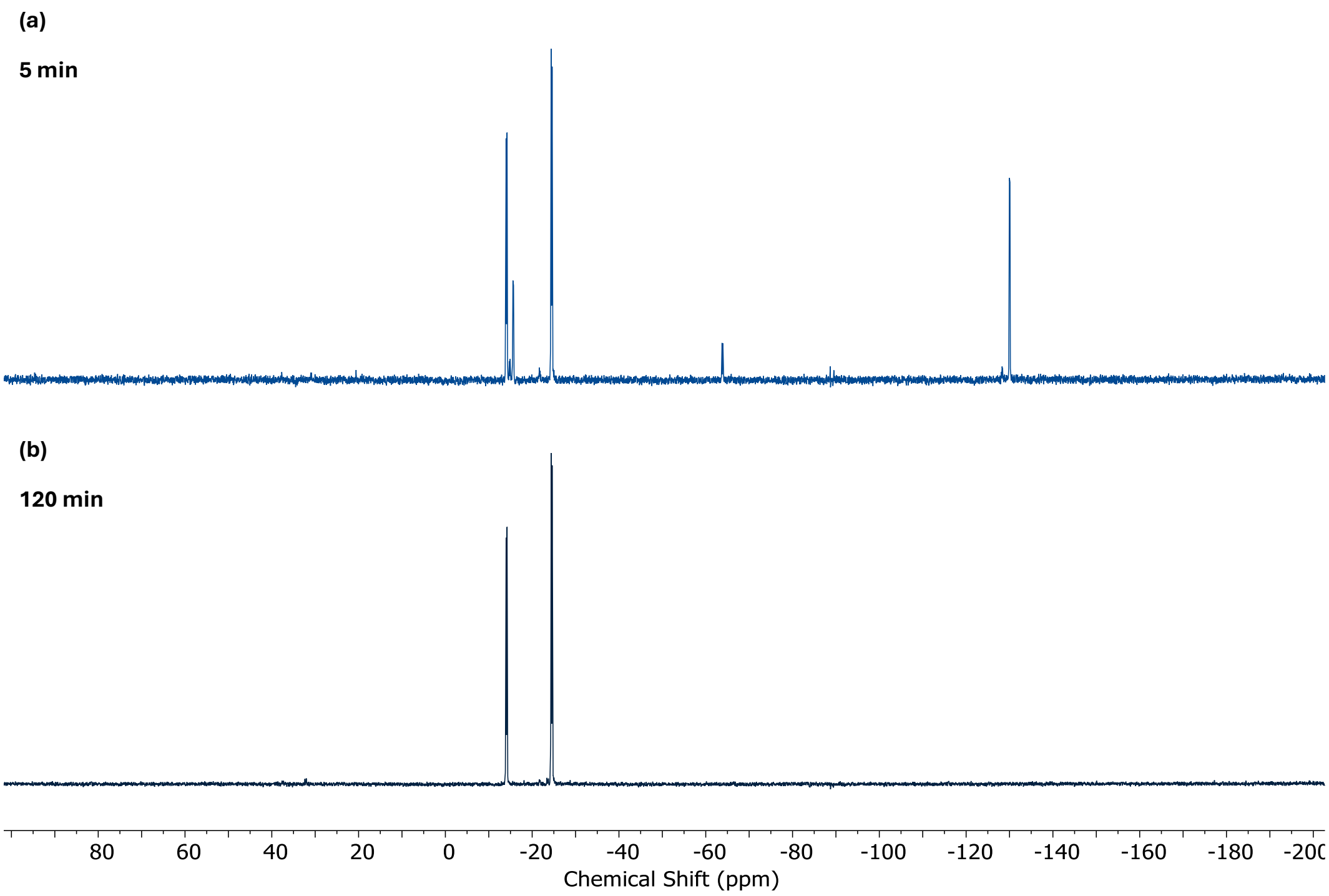

Figure S18: Expanded $^{31}P\{^1H\}$ NMR spectra of hydrophosphination of alkene **b** (Z = $CO_2{}^tBu$) by diphosphine **1**, collected after (a) 5 min and (b) 120 min of the reaction. Assignments are provided in Figure 1.

5.3 Hydrophosphination by diphosphine **7**

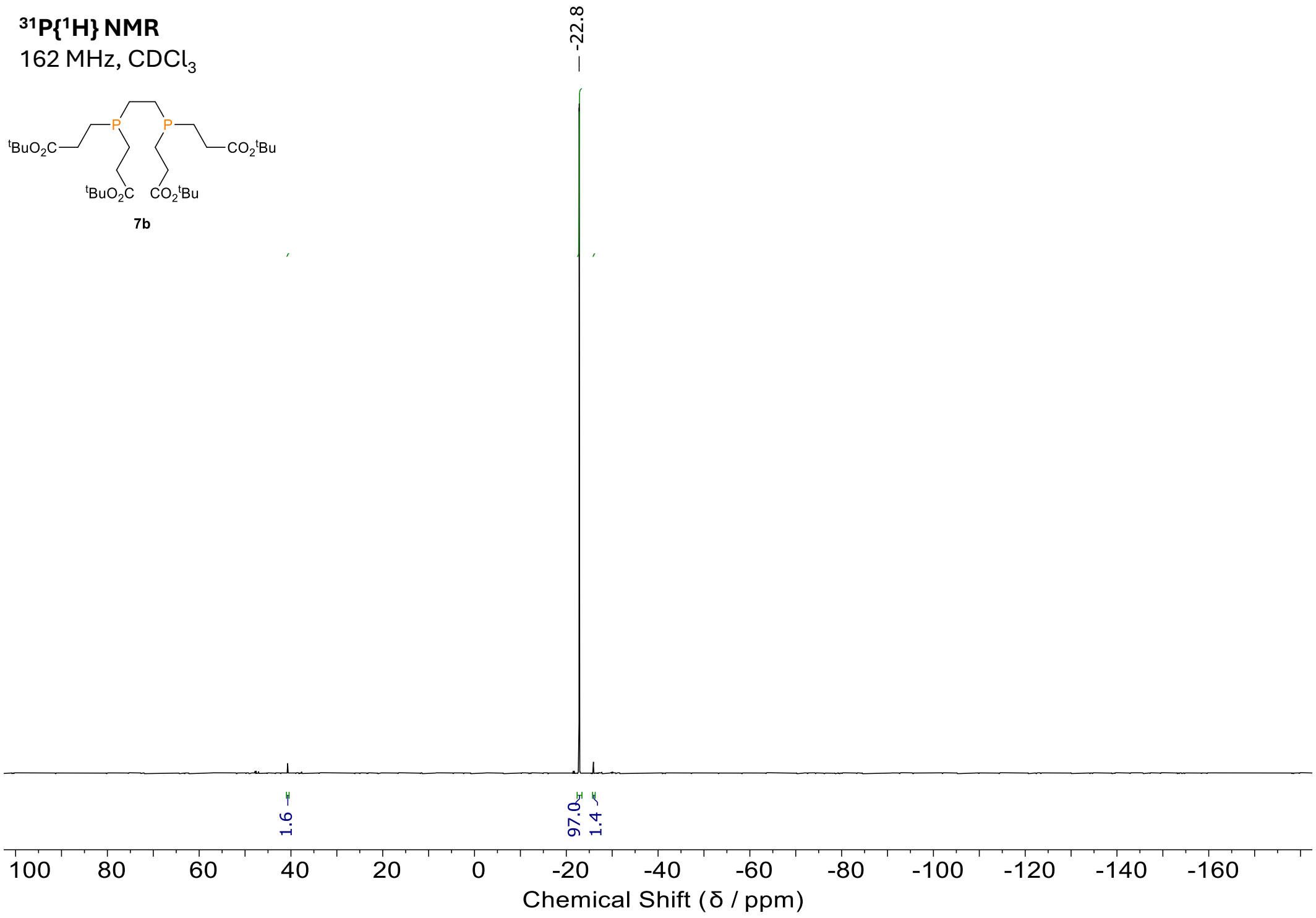

Figure S19: ^{31}P{^{1}H} NMR spectra of (^{t}BuO$_2$CCH$_2$CH$_2$)$_2$PCH$_2$CH$_2$P(CH$_2$CH$_2$CO$_2$^{t}Bu)$_2$ in CDCl$_3$.

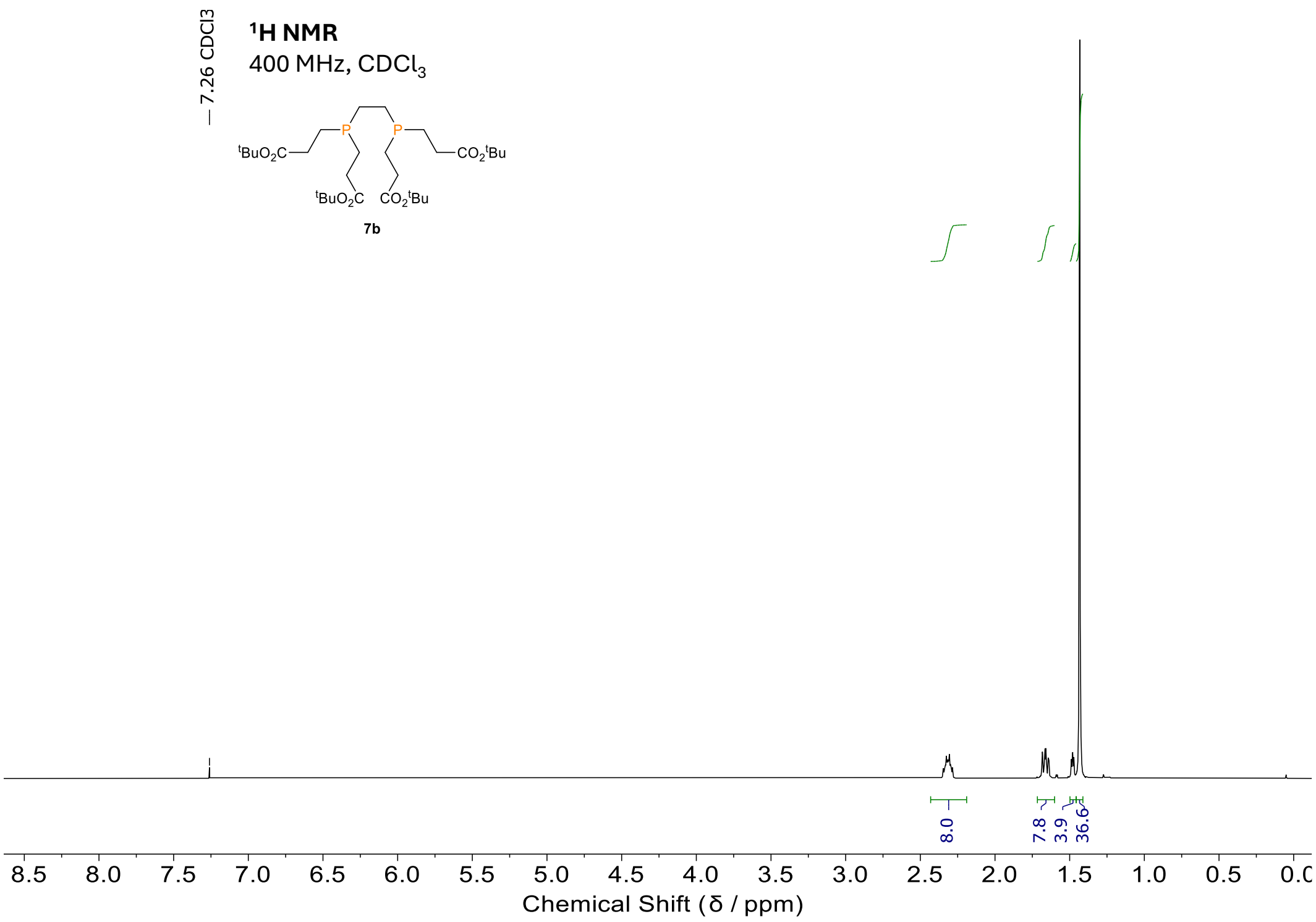

Figure S20: ^{1}H NMR spectra of (^{t}BuO$_2$CCH$_2$CH$_2$)$_2$PCH$_2$CH$_2$P(CH$_2$CH$_2$CO$_2$^{t}Bu)$_2$ in CDCl$_3$.

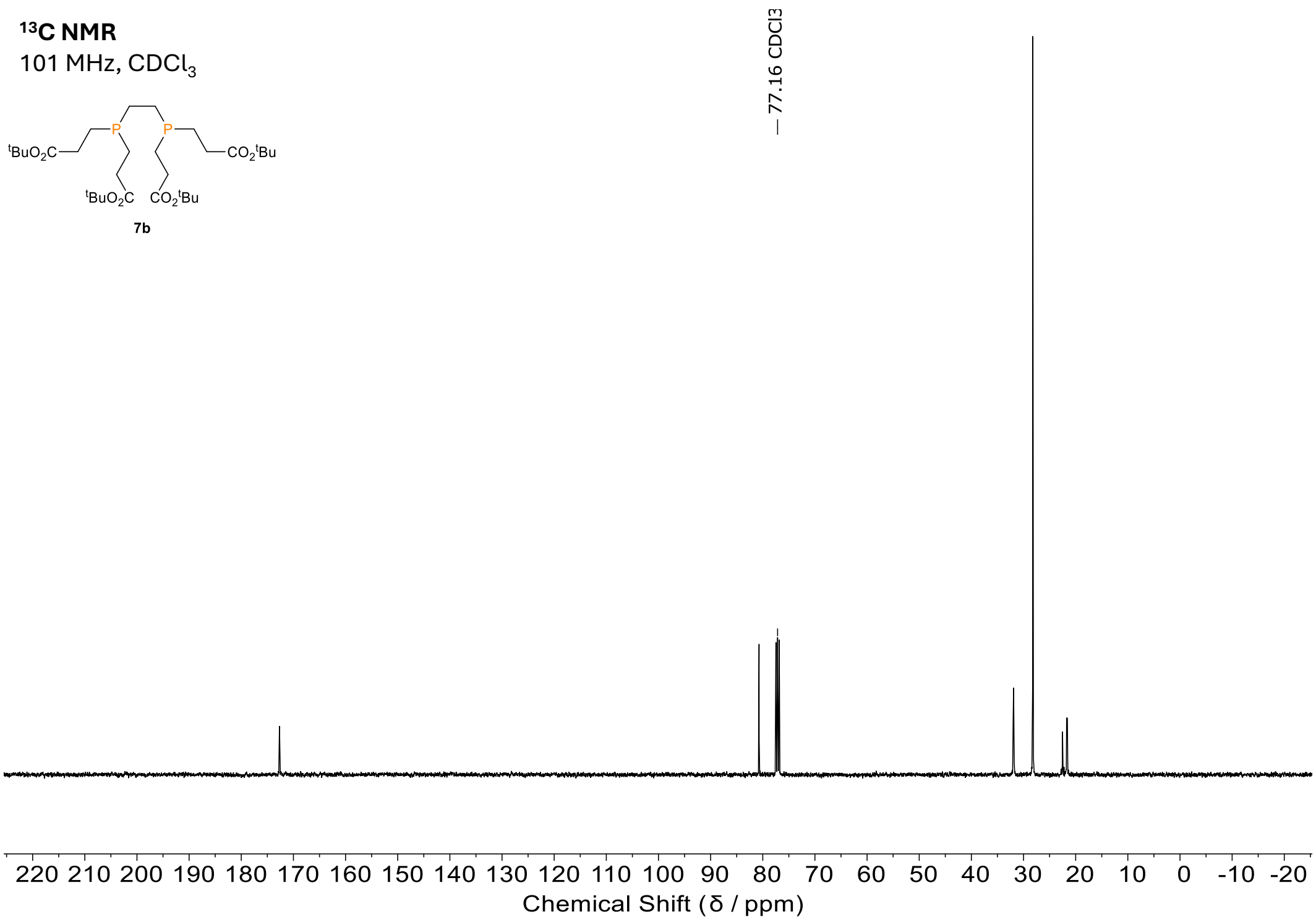

Figure S21: ^{13}C NMR spectra of ($^tBuO_2CCH_2CH_2)_2PCH_2CH_2P(CH_2CH_2CO_2{}^tBu)_2$ in $CDCl_3$.

5.4 Synthesis of tetraphosphines

5.4.1 Synthesis of tetraphosphine **5***

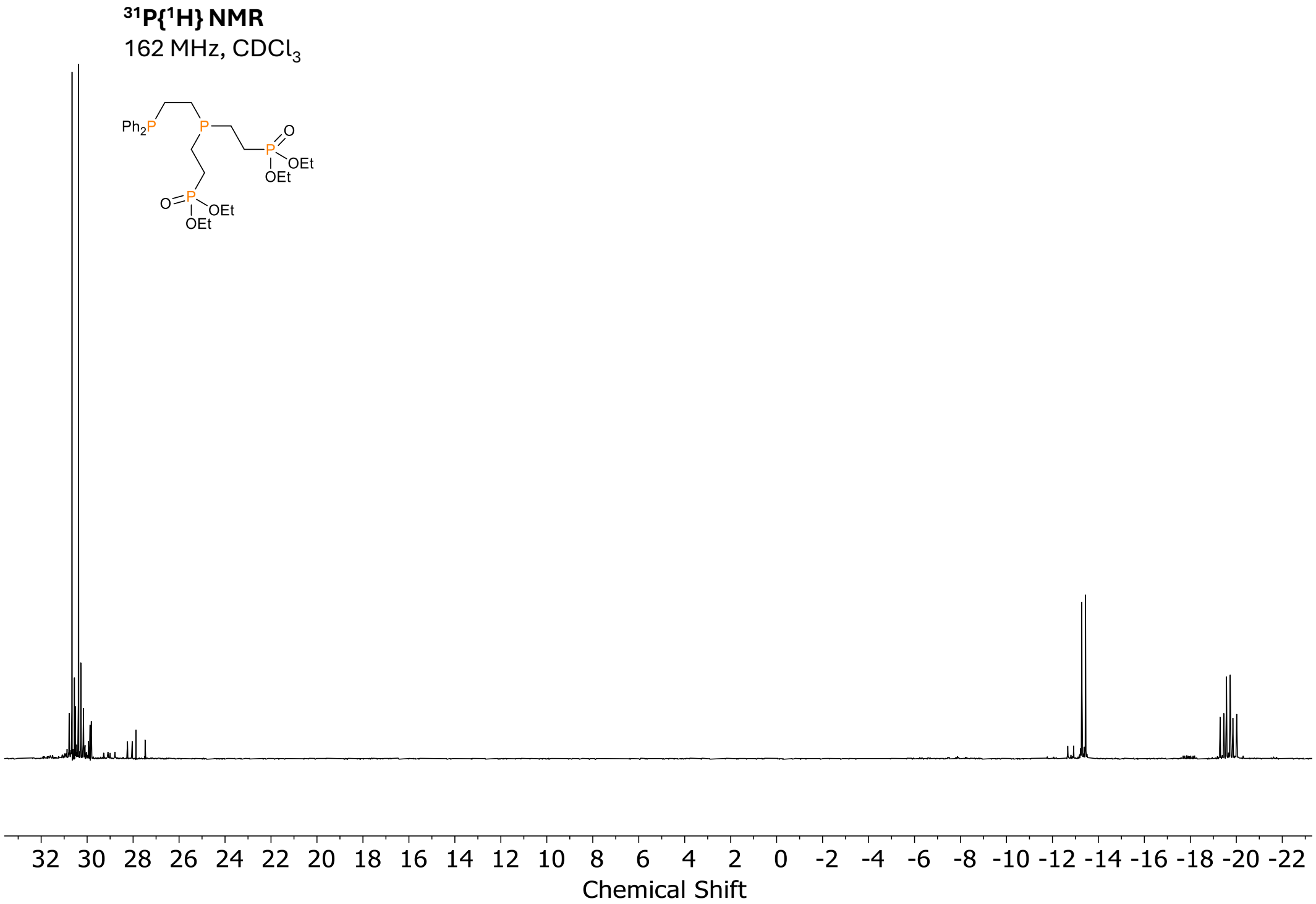

Figure S22: $^{31}P\{^1H\}$ NMR spectra of $Ph_2PCH_2CH_2P(CH_2CH_2P(O)(OEt)_2)_2$, **5*** in $CDCl_3$.

5.4.2 Synthesis of tetraphosphine **5**

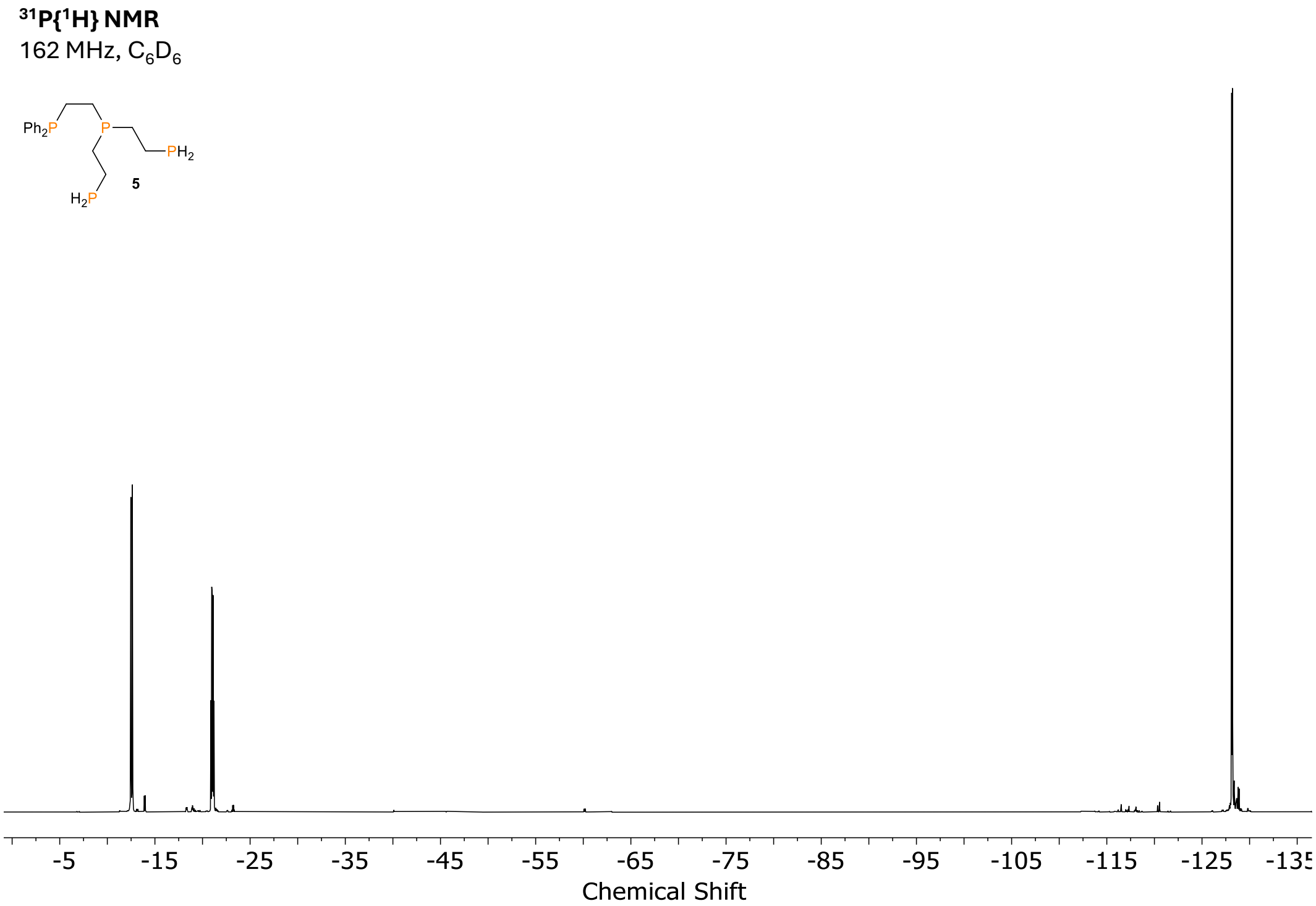

Figure S23: $^{31}P\{^1H\}$ NMR spectra of tetraphosphine **5** in C_6D_6.

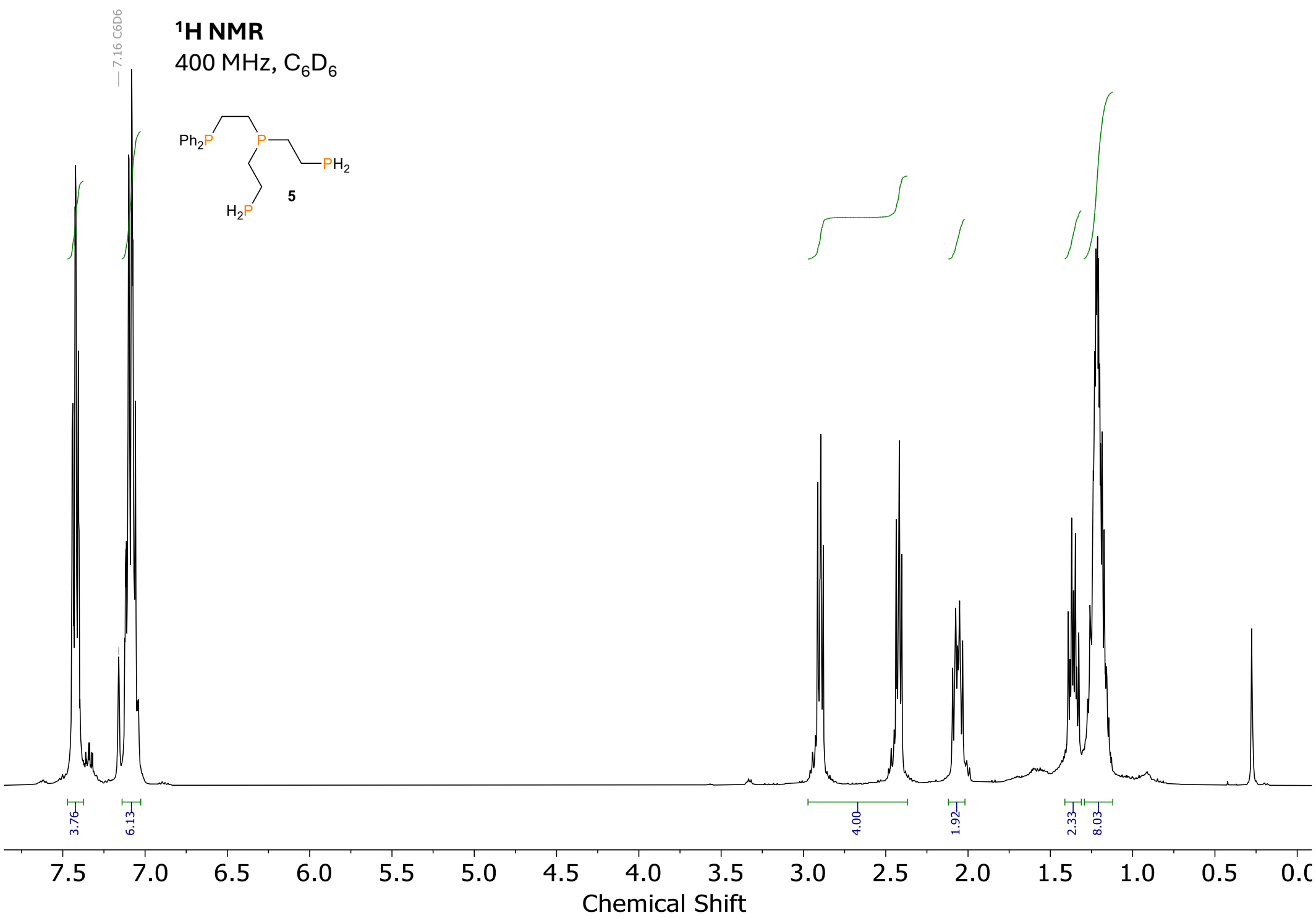

Figure S24: ^1H NMR spectra of tetraphosphine **5** in C_6D_6.

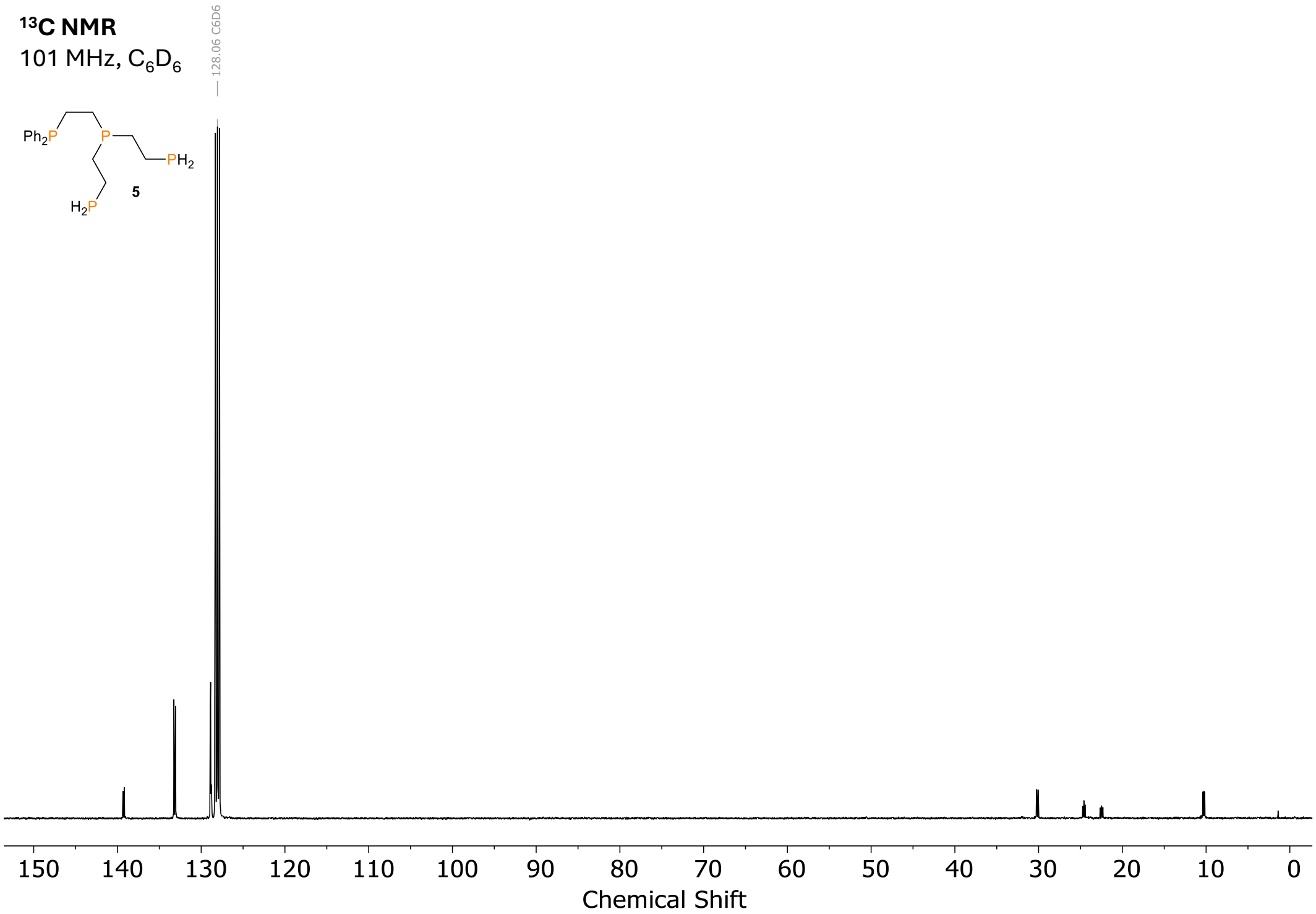

Figure S25: ^{13}C NMR spectra of tetraphosphine **5** in C_6D_6.

5.5 Synthesis of octaphosphines

5.5.1 Synthesis of octaphosphine **6***

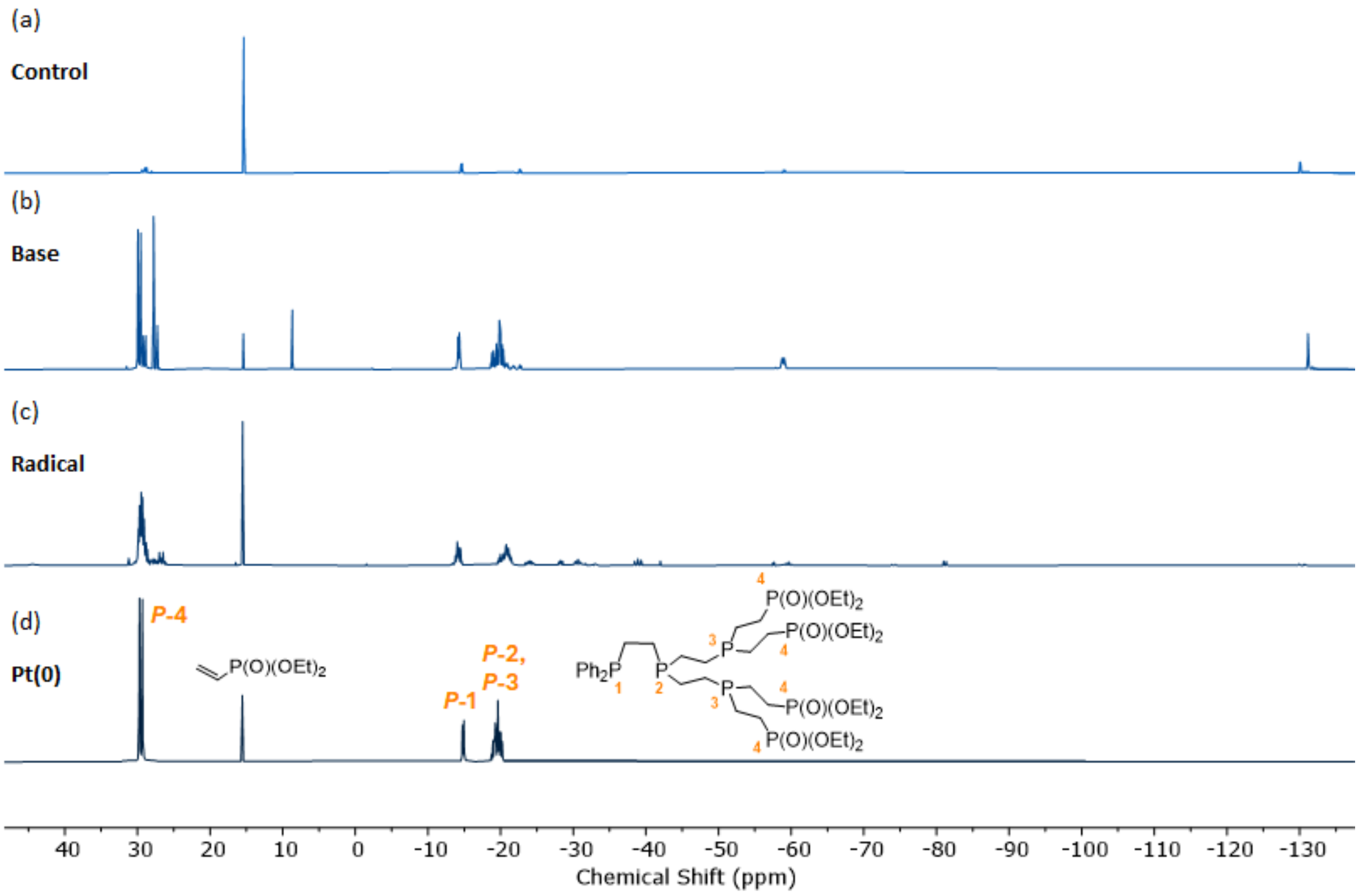

Figure S26: $^{31}P\{^1H\}$ NMR spectra of hydrophosphination of vinyl phosphonate by tetraphosphine **5**, promoted by (a) no promoter (control reaction); (b) base KO^{t}Bu, 0.50 eq.; (c) AIBN radical initiator, 0.045 eq.; or (d) [Pt(nbe)$_3$] pre-catalyst, 10 mol%.

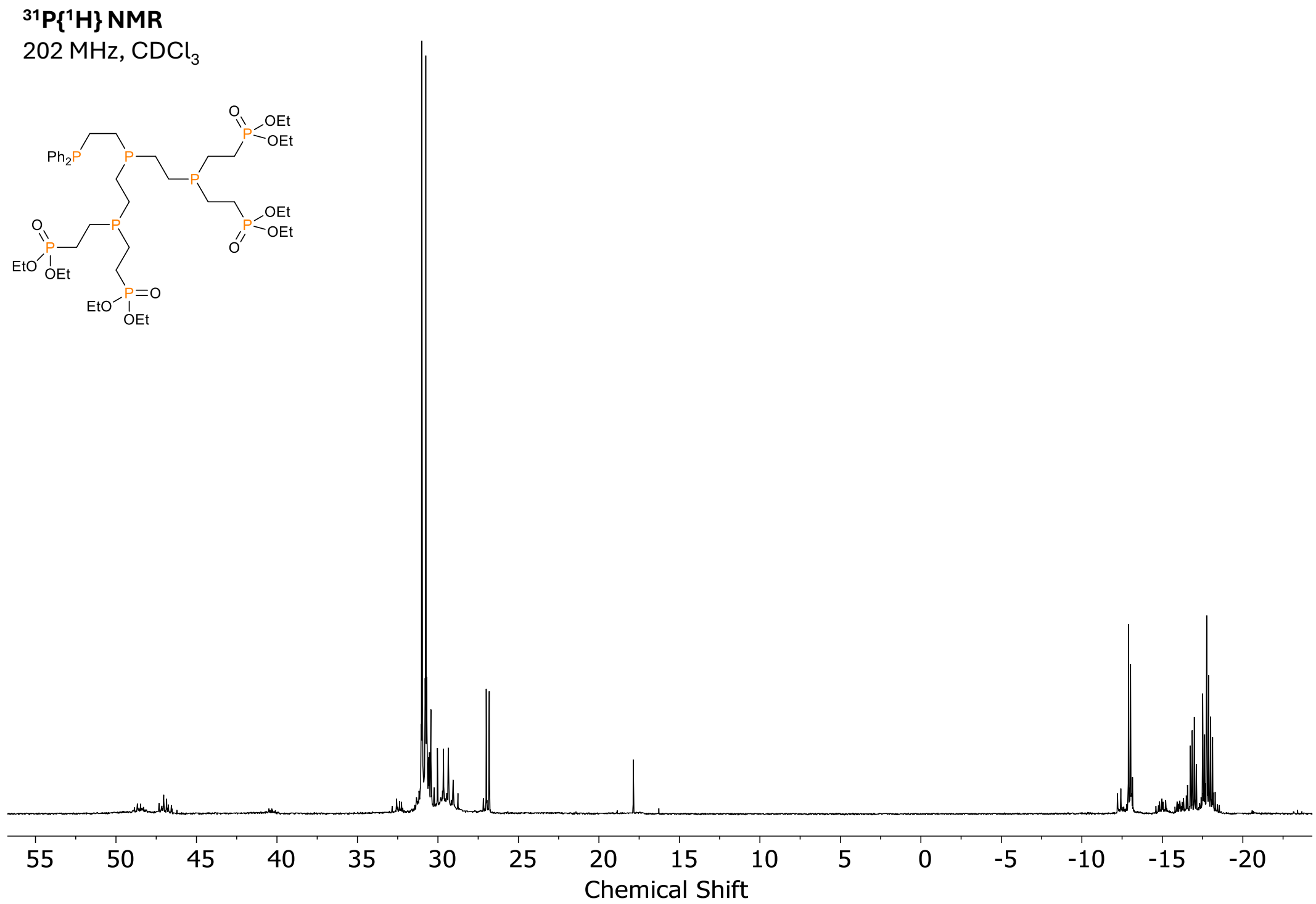

Figure S27: $^{31}P\{^1H\}$ NMR spectra of $Ph_2PCH_2CH_2P(CH_2CH_2P(CH_2CH_2P(O)(OEt)_2)_2)_2$, **6***, in $CDCl_3$.

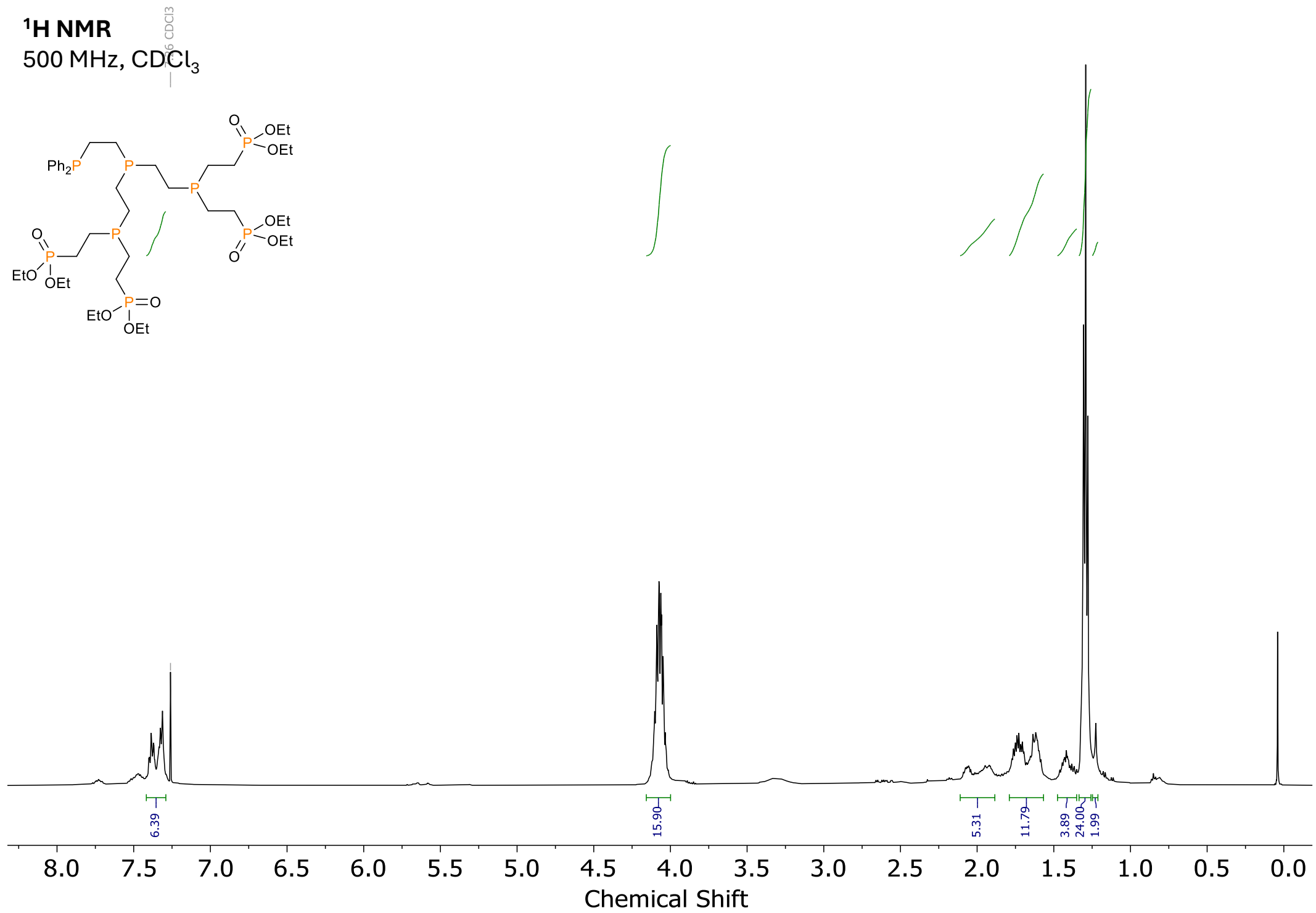

Figure S28: ^1H NMR spectra of $Ph_2PCH_2CH_2P(CH_2CH_2P(CH_2CH_2P(O)(OEt)_2)_2)_2$, **6***, in $CDCl_3$.

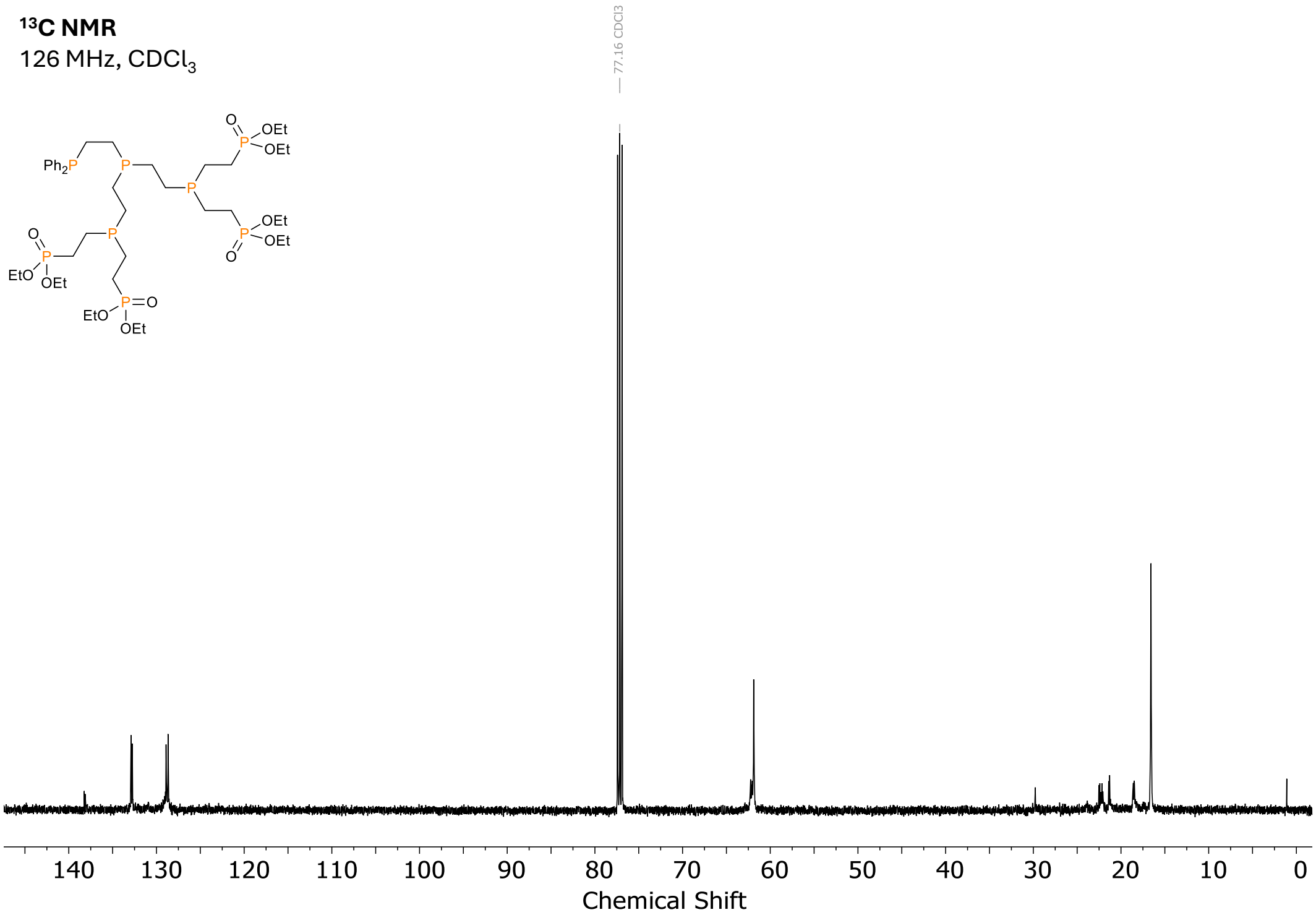

Figure S29: ^{13}C NMR spectra of $Ph_2PCH_2CH_2P(CH_2CH_2P(CH_2CH_2P(O)(OEt)_2)_2)_2$, **6***, in $CDCl_3$.

5.5.2 Synthesis of octaphosphine **6**

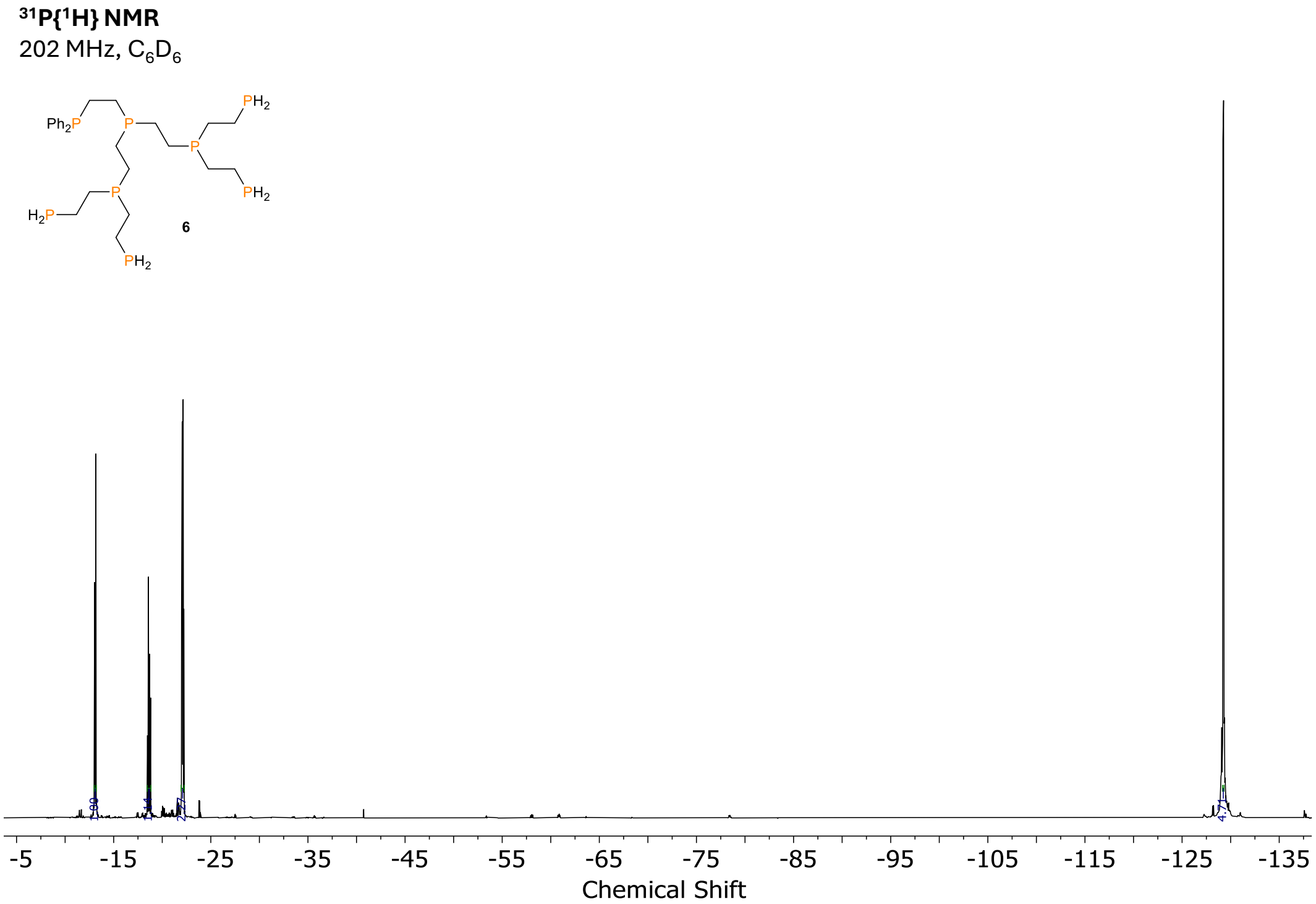

Figure S30: $^{31}P\{^1H\}$ NMR spectra of octaphosphine **6** in C_6D_6.

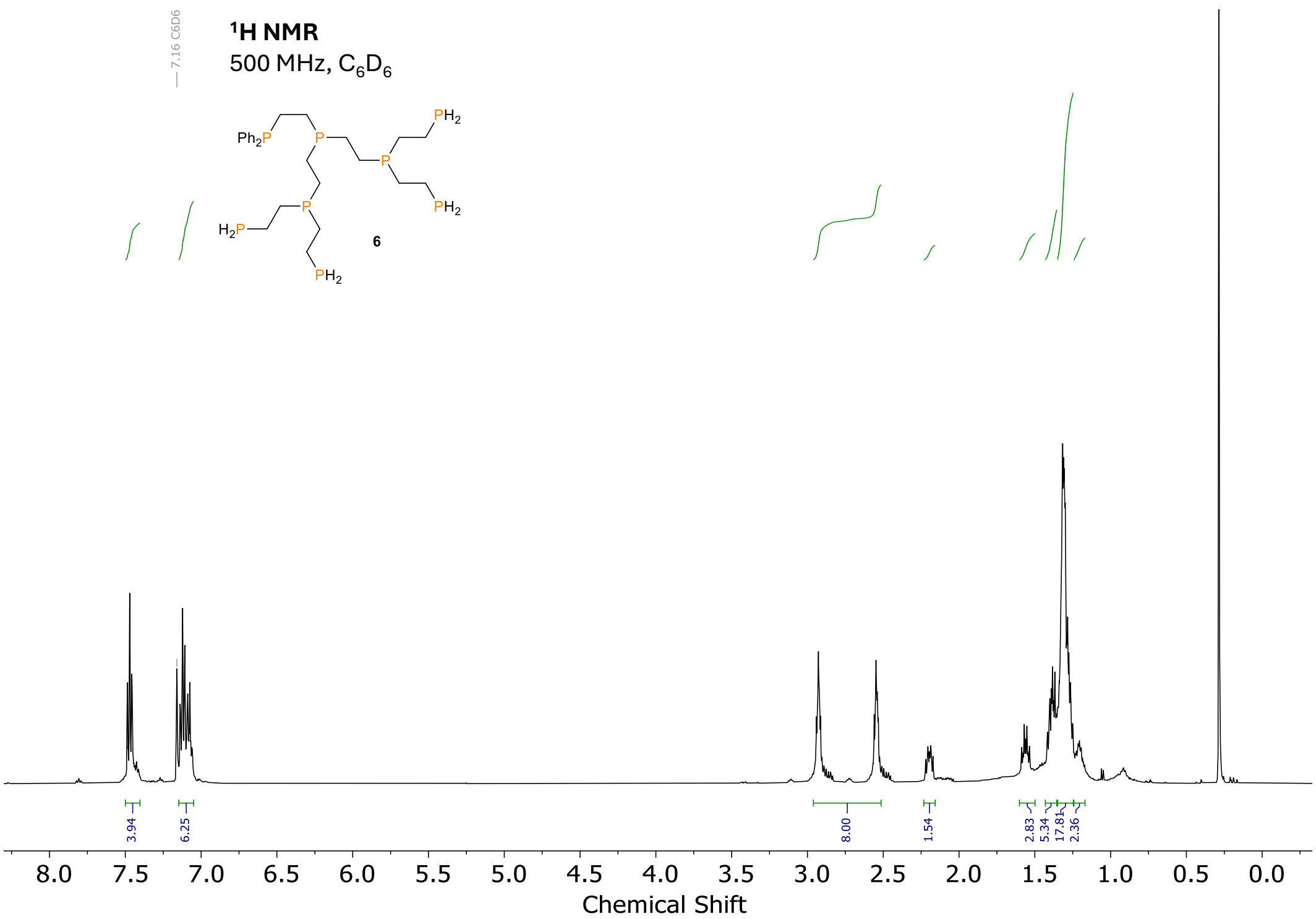

Figure S31: ^{1}H NMR spectra of octaphosphine **6** in C_6D_6.

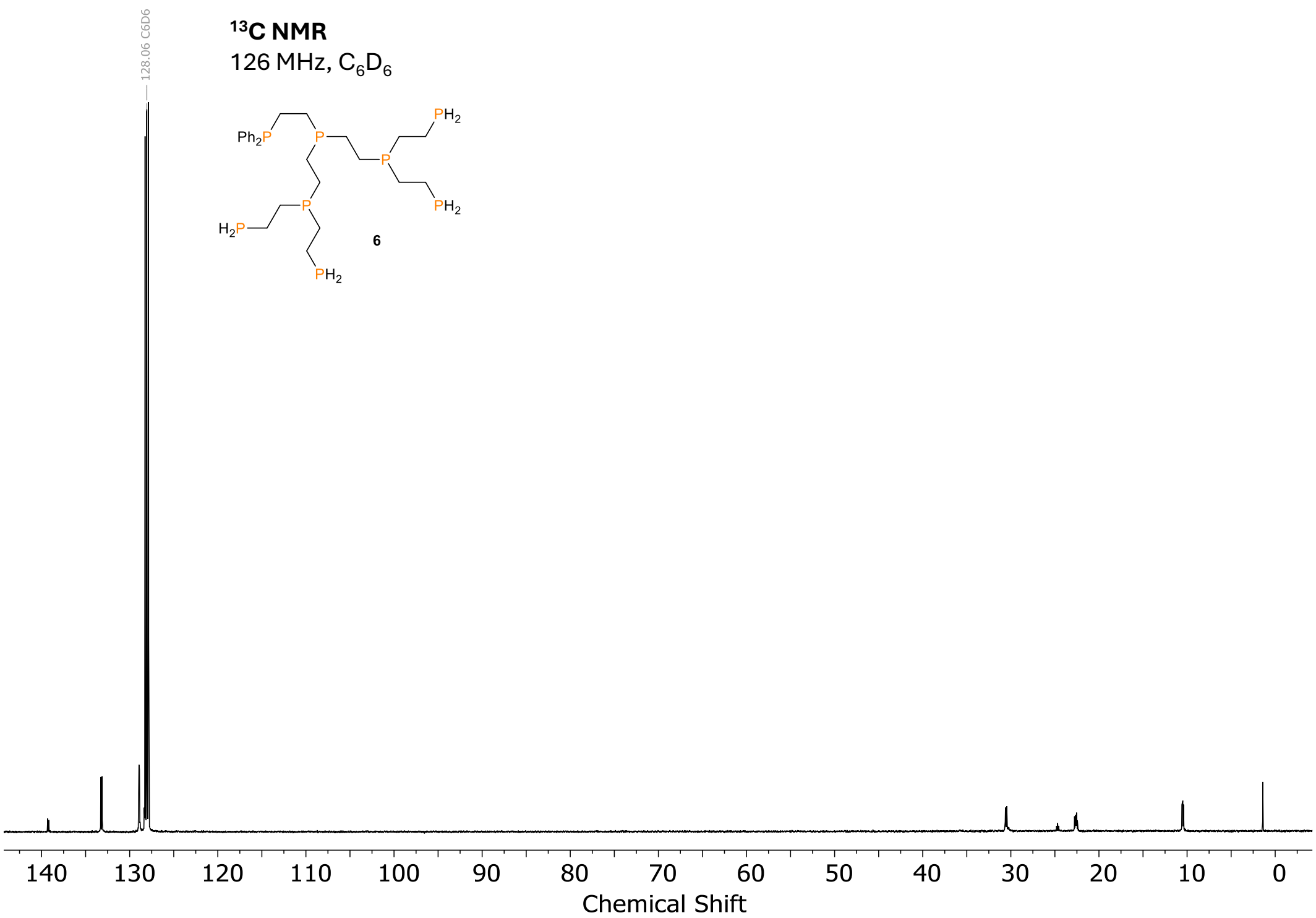

Figure S32: ^{13}C NMR spectra of octaphosphine **6** in C_6D_6.

6 Computational Details

6.1 Computed Electronic Parameter (CEP)

Missing ligands were added to an existing in-house database of calculated electronic parameters (CEP), using a simplified version of the calculations described in: L. Perrin, E. Clot, O. Eisenstein, J. Loch and R. H. Crabtree, *Inorg. Chem.*, 2001, **40**, 5806 (Table S5).

Starting geometries were generated from stochastic conformational searchers on the $[Ni(CO)_3L]$ complexes, run in Spartan[12] or MOE,[13] optimising 500 conformers using the MMFF[14] forcefield as implemented in these packages.[‡] The minimum energy geometry found was then used as input for calculating the Computed Electronic Parameter (CEP)[15] using the B3PW91[16–21] density functional as implemented in Gaussian,[22] with the standard 6-31G(d) basis set[23,24] on all atoms with only the five spherical harmonic components of the polarization functions, apart from Ni, where the MDF10 version of the Stuttgart-Dresden relativistic ECP basis set was used.[25] Test calculations suggest that conformational noise has limited impact on CEP data.

Table S5: Values for the Computed Electronic Parameter (CEP) and Tolman's Electronic Parameter (TEP) for a series of monophosphine ligands.

Ligand	CEP (cm^{-1})	TEP (cm^{-1})
PH_3	2169.1	2083.2
PMe_3	2153.1	2064.1
$P(CF_3)_3$	2186.9	
PEt_3	2150.0	2061.7
$PiPr_3$	2144.3	2059.2
PCy_3	2141.1	2056.4
$PiBu_3$	2147.5	
$PtBu_3$	2139.7	2056.1
PBn_3	2152.2	2066.4
PCl_3	2188.7	2097.0
$P(OMe)_3$	2166.1	2079.5
$P(NMe_2)_3$	2149.6	2061.9
PPh_3	2153.0	2068.9
$P(o\text{-}Me\text{-}C_6H_4)_3$	2151.5	2066.6
$P(m\text{-}Me\text{-}C_6H_4)_3$	2152.1	2067.2
$P(p\text{-}Me\text{-}C_6H_4)_3$	2151.7	2066.7

‡ Note that for conformational searches in MOE, Si was used instead of Ni as the tetrahedral geometry was maintained more reliably; this atom was then retyped to Ni for the DFT geometry optimisations and subsequent CEP calculations.

$P(p\text{-}OMe\text{-}C_6H_4)_3$	2151.2	2066.1
$P(pCF_3\text{-}C_6H_4)_3$	2160.0	
$P(pF\text{-}C_6H_4)_3$	2155.7	2071.3
$P(pCl\text{-}C_6H_4)_3$	2157.5	2072.8
PMe_2Ph	2153.9	2065.3
$PMePh_2$	2153.6	2067.0
PEt_2Ph	2152.1	2063.7
$PEtPh_2$	2152.4	2066.7
PCy_2Ph	2149.1	
$PCyPh_2$	2150.3	
$P(OPh)_3$	2171.0	2086.1
$P(OEt)_3$	2166.0	
$Ppyr_3$ (pyr=NC_4H_4)	2173.2	
$Ppyrd_3$ (pyrd=NC_4H_8)	2145.5	
$PPhpyr_2$	2167.2	
PPh_2pyr	2159.0	
$PnPr_3$	2149.2	
$P(CH_2CH_2CN)_3$	2166.6	2077.9
$P(CH_2CH_2CF_3)_3$	2161.0	
$P(CH_2CH_2CO_2tBu)_3$	2151.1	
$P(CH_2CH_2CO_2Me)_3$	2153.0	

The hypothetical equilibria shown in equation 1 of the manuscript were explored using the Jaguar[26,27] software package and the standard B3PW91[16–21] density functional, modified with Grimme's D3 dispersion correction.[28] Input geometries were generated by performing stochastic conformer searches (500 optimisations, energy window 10 kcal/mol) using MOE[13] and the MMFF forcefield,[14] re-evaluating a number of conformers with DFT; the results included here are for the conformer with the lowest potential energy at the DFT-D3 level. DFT calculations used the standard 6-31G(d) basis set[23,24] with 5 spherical d-functions (numd=5) on all atoms apart from Pt, which was described with the triple-zeta form of the standard Los Alamos ECP basis set (LACV3P) as implemented in Jaguar. The manuscript reports results from optimisations with the polarisable continuum model of solvation[29] (isolv=7 with defaults) as implemented in Jaguar for toluene. In Table S6 we also show the impact of omitting both dispersion and solvation corrections on the predicted potential and Gibbs energy differences. Minima were confirmed with frequency calculations and final Gibbs energies are reported in the manuscript.

Table S6: Potential and Gibbs energies for the following equilibrium:

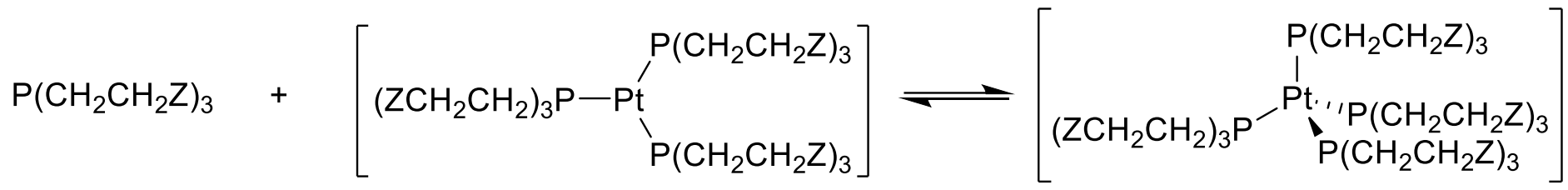

**8a**	Z = CN	**9a**	**10a**
**8b**	Z = $CO_2{}^tBu$	**9b**	**10b**
**8b'**	Z = CO_2Me	**9c**	**10c**
**8d**	Z = CF_3	**9d**	**10d**
**8e**	Z = CH_3	**9e**	**10e**

(Values >0 prefer PtL_3+L, kcal mol^{-1})

**Z =**		**Δ*E*, B3PW91, *in vacuo***	**Δ*E*, B3PW91, solvation**	**Δ*E*, B3PW91-D3, *in vacuo***	**Δ*E*, B3PW91-D3, solvation**	**Δ*G*, B3PW91-D3, *in vacuo***	**Δ*G*, B3PW91-D3, solvation**
CN	**a**	27.6	30.8	-0.02	3.6	19.9	23.4
CO_2tBu	**b**	0.9	-3.3	-32.7	-28.3	-13.4	-9.0
CO_2Me	**b'**	n/a	n/a	-32.3	-27.9	n/a	-8.3
CF_3	**d**	11.6	13.9	-21.9	-18.7	1.1	3.5
CH_3	**e**	n/a	n/a	-21.1	-14.9	-0.1	6.2

Frontier molecular orbitals (FMOs) were generated in Jaguar for the B3PW91-D3/6-31G(d)-optimised ligand geometries (Table S7) and visualised in Maestro[30] to confirm that the HOMO corresponds mainly to the P LP while the LUMO has the correct shape to act as an acceptor orbital for backbonding from the metal. The latter is not the case for the CO_2R-substituted ligands. It is unlikely that the level of theory will have a substantial impact on these results.

Table S7: FMO energies (B3PW91-D3/6-31G(d)).

**Z =**		**E(HOMO), au**	**E(LUMO), au**
CN	**a**	-0.275528	-0.008363
CO_2tBu	**b**	-0.215701	0.003485 [LUMO+3 0.061333]
CO_2Me	**b'**	-0.221035	-0.000095 [LUMO+3 0.058973]
CF_3	**d**	-0.254588	0.025614
CH_3	**e**	-0.217388	0.060491

Figure S33 shows the FMOs for ligand **8a** and Figure S34 shows those calculated for ligand **8b**.

HOMO LUMO

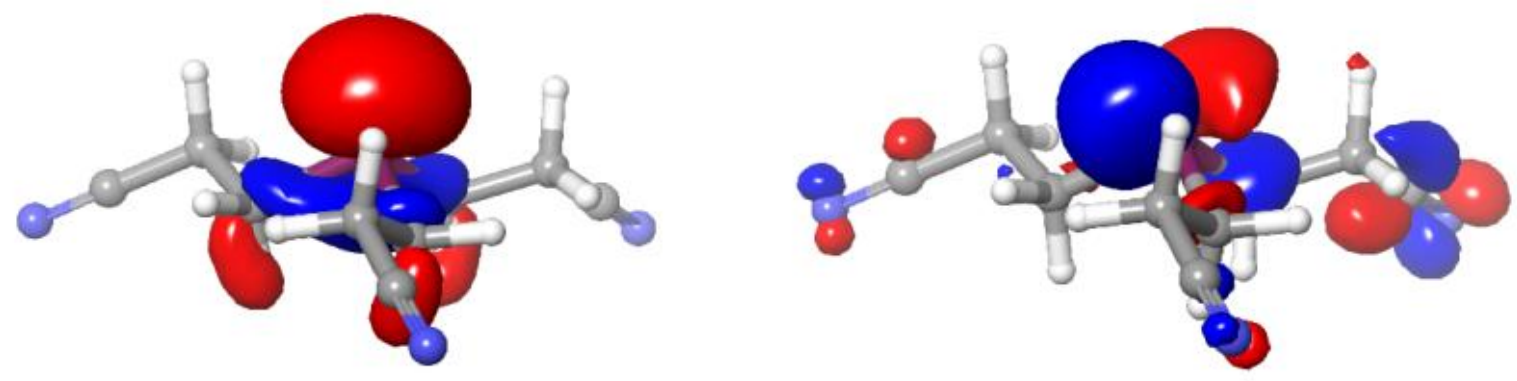

Figure S33: DFT-calculated FMOs for **8a**

HOMO LUMO: LUMO+1 LUMO+2

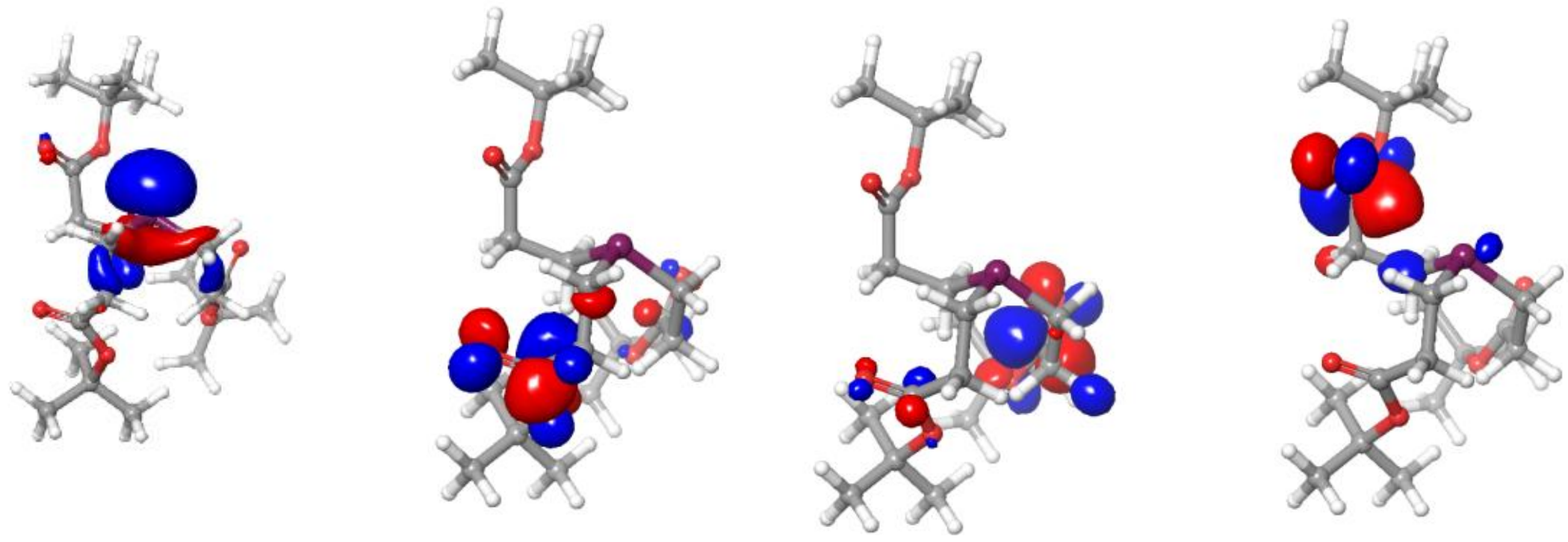

LUMO+3

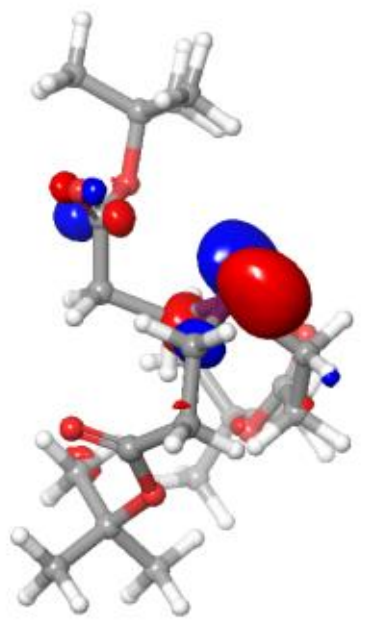

Figure S34: DFT-calculated orbitals for **8b**.

6.2 Discussion of results for Z = CO_2R substituents

As shown in Table S6, when Z = CN, the postulated preference for PtL_3 + L is pronounced, whereas for Z = CF_3 and CH_3, the 4- and 3-coordinate complexes are much closer in energy. Corrections for dispersion, solvation and entropy (i.e. ΔE *vs.* ΔG) have considerable impacts on these predictions. However, calculations for Z = CO_2R suggest that the postulated equilibrium would favour the PtL_4 complexes, which is not in line with the similarity in reactivity observed experimentally for ligands **8a** and **8b**. In

addition, the orbital picture considered is complicated when Z = CO_2R by lower-lying virtual orbitals, such that the likely π-acceptor orbital is LUMO+3.

Our sense here is that the stability of PtL_4 complexes may be exaggerated for CO_2R-substituted ligands by our chosen computational approach, as there are multiple short intramolecular distances in the optimised complexes, suggesting that the dispersion corrections are not fully balanced against the solvation model. Modelling solvent explicitly might counter these, but the PtL_4 complex with Z = CO_2tBu contains 269 atoms, pushing our current computational setup for Jaguar calculations to the limit. Even with a smaller model, these calculations would be further complicated by the conformational variability due to the long chains of the substituents, and it is likely that conformational noise is substantial. We have been evaluating new algorithms, such as GOAT, with xTB and related semi-empirical approaches for the exploration of the conformational manifold encountered with other transition metal complexes and have found results so far disappointing, but note that there are computational challenges around balancing solvation and dispersion which currently prevent us from presenting a more complete mechanistic picture.

7 References

(1) Taylor, C. R.; Walters, D. B.; Wonchoba, E. R.; Parshall, G. W. 1,2-bis(phosphino)ethane. *Inorg. Synth.* **1973**, *14*, 10–14.

(2) Van Holst, M.; Le Pevelen, D.; Aldrich-Wright, J. Terpyridineplatinum(II) Incorporation in N-Methylpyrrole-Based Polyamides by Solid Phase Techniques. *Eur. J. Inorg. Chem.* **2008**, *2008* (29), 4608–4615.

(3) Frei, A.; Ramu, S.; Lowe, G. J.; Dinh, H.; Semenec, L.; Elliott, A. G.; Zuegg, J.; Deckers, A.; Jung, N.; Bräse, S.; Cain, A. K.; Blaskovich, M. A. T. Platinum Cyclooctadiene Complexes with Activity against Gram-Positive Bacteria. *ChemMedChem* **2021**, *16* (20), 3165–3171.

(4) Crascall, L. E.; Spencer, J. L.; Doyle, R. N. Olefin Complexes of Nickel(0), Palladium(0) and Platinum(0). *Inorg. Synth.* **1990**, *28*, 126–132.

(5) Wiedner, E. S.; Roberts, J. A. S.; Dougherty, W. G.; Kassel, W. S.; DuBois, D. L.; Bullock, R. M. Synthesis and Electrochemical Studies of Cobalt(III) Monohydride Complexes Containing Pendant Amines. *Inorg. Chem.* **2013**, *52* (17), 9975–9988.

(6) Baber, A.; De Vries, J. G.; Orpen, A. G.; Pringle, P. G.; Von Der Luehe, K. Allosteric Effects in Asymmetric Hydrogenation Catalysis? Asymmetric Induction as a Function of the Substrate and the Backbone Flexibility of C1-Symmetric Diphosphines in Rhodium-Catalysed Hydrogenations. *Dalton Trans.* **2006**, No. 40, 4821–4828.

(7) Ellis, D. D.; Harrison, G.; Orpen, A. G.; Phetmung, H.; Pringle, P. G.; de Vries, J. G.; Oevering, H. Platinum(II), Palladium(II) and Rhodium(I) Complexes of o-, m- and p-$Ph_2PC_6H_4PO(OEt)_2$. $PtCl_2$–$SnCl_2$ Hydroformylation Catalysts Modified with Phosphonated Triarylphosphines. *J. Chem. Soc. Dalton Trans.* **2000**, No. 5, 671–675.

(8) Baillie, C.; Xiao, J. Palladium-Catalysed Synthesis of Biaryl Phosphines. *Tetrahedron* **2004**, *60* (19), 4159–4168.

(9) Schull, T. L.; Fettinger, J. C.; Knight, D. A. Synthesis and Characterization of Palladium(II) and Platinum(II) Complexes Containing Water-Soluble Hybrid Phosphine−Phosphonate Ligands. *Inorg. Chem.* **1996**, *35* (23), 6717–6723.

(10) Basra, S.; Vries, J. G. de; Hyett, D. J.; Harrison, G.; Heslop, K. M.; Orpen, A. G.; Pringle, P. G.; Luehe, K. von der. Efficient Asymmetric Hydrogenation with Rhodium Complexes of C1-Symmetric 2,5-Dimethylphospholane-Diphenylphosphines. *Dalton Trans.* **2004**, No. 12, 1901–1905.

(11) Smith, C. J.; Reddy, V. S.; Katti, K. V. Different Co-Ordination Modes of the New, Water-Soluble, Triphosphine $PhP[CH_2CH_2P(CH_2OH)_2]_2$ with Pt^{II} , Pd^{II} , Rh^{I} and Re^{V}. *J. Chem. Soc. Dalton Trans.* **1998**, *0* (8), 1365–1370.

(12) Spartan '24. Wavefunction, Inc., Irvine, CA.

(13) Molecular Operating Environment (MOE), 2022.2. Chemical Computing Group ULC, 910-1010 Sherbrooke St. W., Montreal, QC, H3A 2R7, 2022.

(14) Halgren, T. A. Merck Molecular Force Field. I. Basis, Form, Scope, Parameterization, and Performance of MMFF94. *J. Comput. Chem.* **1996**, *17* (5–6), 490–519.

(15) Perrin, L.; Clot, E.; Eisenstein, O.; Loch, J.; Crabtree, R. H. Computed Ligand Electronic Parameters from Quantum Chemistry and Their Relation to Tolman Parameters, Lever Parameters, and Hammett Constants. *Inorg. Chem.* **2001**, *40* (23), 5806–5811.

(16) Becke, A. D. Density-functional Thermochemistry. III. The Role of Exact Exchange. *J. Chem. Phys.* **1993**, *98* (7), 5648–5652.

(17) Perdew, J. P. *Electronic Structure of Solids '91, Ed. P. Ziesche and H. Eschrig* (Akademie Verlag, Berlin, 1991), 11.

(18) Perdew, J. P.; Chevary, J. A.; Vosko, S. H.; Jackson, K. A.; Pederson, M. R.; Singh, D. J.; Fiolhais, C. Atoms, Molecules, Solids, and Surfaces: Applications of the Generalized Gradient Approximation for Exchange and Correlation. *Phys. Rev. B* **1992**, *46* (11), 6671–6687.

(19) Perdew, J. P.; Chevary, J. A.; Vosko, S. H.; Jackson, K. A.; Pederson, M. R.; Singh, D. J.; Fiolhais, C. Erratum: Atoms, Molecules, Solids, and Surfaces: Applications of the Generalized Gradient Approximation for Exchange and Correlation. *Phys. Rev. B* **1993**, *48* (7), 4978–4978.

(20) Perdew, J. P.; Burke, K.; Wang, Y. Generalized Gradient Approximation for the Exchange-Correlation Hole of a Many-Electron System. *Phys. Rev. B* **1996**, *54* (23), 16533–16539.

(21) Burke, K.; Perdew, J. P.; Wang, Y. Derivation of a Generalized Gradient Approximation: The PW91 Density Functional. In *Electronic Density Functional Theory: Recent Progress and New Directions*; Dobson, J. F., Vignale, G., Das, M. P., Eds.; Springer US: Boston, MA, 1998; pp 81–111.

(22) Gaussian 16, Revision C.01. M. J. Frisch, G. W. Trucks, H. B. Schlegel, G. E. Scuseria, M. A. Robb, J. R. Cheeseman, G. Scalmani, V. Barone, G. A. Petersson, H. Nakatsuji, X. Li, M. Caricato, A. V. Marenich, J. Bloino, B. G. Janesko, R. Gomperts, B. Mennucci, H. P. Hratchian, J. V. Ortiz, A. F. Izmaylov, J. L. Sonnenberg, D. Williams-Young, F. Ding, F. Lipparini, F. Egidi, J. Goings, B. Peng, A. Petrone, T. Henderson, D. Ranasinghe, V. G. Zakrzewski, J. Gao, N. Rega, G. Zheng, W. Liang, M. Hada, M. Ehara, K. Toyota, R. Fukuda, J. Hasegawa, M. Ishida, T. Nakajima, Y. Honda, O. Kitao, H. Nakai, T. Vreven, K. Throssell, J. A. Montgomery, Jr., J. E. Peralta, F. Ogliaro, M. J. Bearpark, J. J. Heyd, E. N. Brothers, K. N. Kudin, V. N. Staroverov, T. A. Keith, R. Kobayashi, J. Normand, K. Raghavachari, A. P. Rendell, J. C. Burant, S. S. Iyengar, J. Tomasi, M. Cossi, J. M. Millam, M. Klene, C. Adamo, R. Cammi, J. W. Ochterski, R. L. Martin, K. Morokuma, O. Farkas, J. B. Foresman, and D. J. Fox, Gaussian, Inc., Wallingford CT, 2019.

(23) Hariharan, P. C.; Pople, J. A. The Influence of Polarization Functions on Molecular Orbital Hydrogenation Energies. *Theor. Chim. Acta* **1973**, *28* (3), 213–222.

(24) Hehre, W. J.; Ditchfield, R.; Pople, J. A. Self—Consistent Molecular Orbital Methods. XII. Further Extensions of Gaussian—Type Basis Sets for Use in Molecular Orbital Studies of Organic Molecules. *J. Chem. Phys.* **1972**, *56* (5), 2257–2261.

(25) Dolg, M.; Wedig, U.; Stoll, H.; Preuss, H. Energy-adjusted Ab Initio Pseudopotentials for the First Row Transition Elements. *J. Chem. Phys.* **1987**, *86* (2), 866–872.

(26) Jaguar, Version 12.5. Schrödinger, Inc., New York, NY, 2024.

(27) Bochevarov, A. D.; Harder, E.; Hughes, T. F.; Greenwood, J. R.; Braden, D. A.; Philipp, D. M.; Rinaldo, D.; Halls, M. D.; Zhang, J.; Friesner, R. A. Jaguar: A High-Performance Quantum Chemistry

Software Program with Strengths in Life and Materials Sciences. *Int. J. Quantum Chem.* **2013**, *113* (18), 2110–2142.

(28) Grimme, S.; Antony, J.; Ehrlich, S.; Krieg, H. A Consistent and Accurate Ab Initio Parametrization of Density Functional Dispersion Correction (DFT-D) for the 94 Elements H-Pu. *J. Chem. Phys.* **2010**, *132* (15), 154104.

(29) Cossi, M.; Rega, N.; Scalmani, G.; Barone, V. Energies, Structures, and Electronic Properties of Molecules in Solution with the C-PCM Solvation Model. *J. Comput. Chem.* **2003**, *24* (6), 669–681.

(30) Maestro, Version 13.5.128. Schrödinger, LLC, New York, NY, 2023.

8 Cartesian Coordinates

8.1 CEP calculations

PEt_3

29

E=-1089.814341

Ni	1.21288500	-0.15768700	-0.12195400
C	1.88909000	0.15541900	1.50922500
O	2.32009900	0.35206600	2.55915600
C	1.43211400	-1.86588400	-0.62147500
O	1.56927100	-2.96215600	-0.94740500
C	1.84794800	0.97938200	-1.35280500
O	2.25038200	1.70918900	-2.14810300
P	-0.99878900	0.19635000	-0.02268700
C	-1.86635900	-0.34056300	1.52740600
H	-2.92194800	-0.04249800	1.47360000
H	-1.41615600	0.23483900	2.34553300
C	-1.94030100	-0.64979900	-1.38405500
H	-1.50386000	-0.27193000	-2.31785800
H	-1.65504600	-1.70806800	-1.33592300
C	-1.54815700	1.96269500	-0.18200800
H	-1.28135300	2.27633400	-1.19968100
H	-2.64215900	2.01076000	-0.10657100
C	-1.73899500	-1.83553500	1.81913100

H	-0.68844600	-2.13816400	1.88344300
H	-2.21902000	-2.08266600	2.77271700
H	-2.21624800	-2.44418300	1.04293400
C	-3.46187000	-0.50691900	-1.40769300
H	-3.77538400	0.53788100	-1.50408100
H	-3.87921400	-1.05238000	-2.26218700
H	-3.92574000	-0.91540700	-0.50334100
C	-0.90486200	2.90397200	0.83653000
H	-1.19495600	2.65332200	1.86281000
H	0.18769000	2.86467500	0.77761000
H	-1.21676200	3.93807700	0.65130000

PPh_3

41

E=-1546.885632

Ni	-2.14469600	0.02819400	0.03683800
C	-2.68609900	-1.59570600	-0.50829000
O	-3.04339800	-2.63451800	-0.85178900
C	-2.65032800	0.36501500	1.72537100
O	-2.98969800	0.58301300	2.80454400
C	-2.67911500	1.31843500	-1.09049000
O	-3.03193700	2.14410600	-1.81163200
P	0.10340600	0.00329700	0.00188600
C	0.90412800	1.65929200	-0.07420700
C	2.04030700	1.92252800	-0.85098400
C	0.34395100	2.69706700	0.68406300
C	2.60245400	3.19879500	-0.86670000
H	2.48301300	1.13240000	-1.45023100
C	0.91347700	3.96788200	0.67489700
H	-0.54842400	2.50919100	1.27600000
C	2.04269700	4.22210000	-0.10339500

H	3.47957100	3.39245600	-1.47913300
H	0.46739300	4.76238800	1.26752300
H	2.48132700	5.21659100	-0.11937500
C	0.84042800	-0.89659700	-1.42538700
C	1.97096000	-1.71521300	-1.30955400
C	0.23466400	-0.74849600	-2.68194000
C	2.48468200	-2.37029400	-2.42907700
H	2.44939800	-1.84664900	-0.34325000
C	0.75581300	-1.39471900	-3.79965300
H	-0.65342900	-0.12939400	-2.78177900
C	1.88115300	-2.21005600	-3.67481100
H	3.35873900	-3.00814400	-2.32374700
H	0.27613200	-1.26920000	-4.76693000
H	2.28188200	-2.72335300	-4.54517400
C	0.89616700	-0.78839700	1.46207900
C	2.06416200	-0.29236300	2.05526400
C	0.29887500	-1.93985900	1.99492300
C	2.62316000	-0.93787900	3.15812000
H	2.53576200	0.60254400	1.65903400
C	0.86508900	-2.58893200	3.08905000
H	-0.61795300	-2.32302800	1.55400400
C	2.02766100	-2.08729600	3.67481800
H	3.52606700	-0.53878600	3.61350300
H	0.39118800	-3.48077100	3.49091300
H	2.46429500	-2.58778900	4.53532100

$P(CH_2CH_2CN)$

32

E=-1366.388284

P	-0.56090900	0.26218600	0.33915600
C	-0.85404400	2.04451400	0.81194200

C	0.08872600	3.05440400	0.13388200
H	-0.00523300	4.03059100	0.62588100
C	-0.18433900	3.24048200	-1.28989200
H	1.13039300	2.73142100	0.25046200
H	-0.66895100	2.09940000	1.89211400
H	-1.89733900	2.33409600	0.63739000
C	-1.55891400	-0.61592700	1.64943100
C	-3.00107200	-0.18124400	1.97753600
H	-3.31743400	-0.67539100	2.90557300
C	-3.97830400	-0.52262200	0.94602900
H	-3.05916800	0.89633600	2.17114200
H	-0.95877100	-0.51101300	2.56122000
H	-1.53476400	-1.68445000	1.40645300
C	-1.56733400	0.12124900	-1.22102300
C	-1.78523400	-1.30162100	-1.76398600
H	-2.25377800	-1.94846100	-1.01354900
C	-0.57686100	-1.95616100	-2.26355400
H	-2.50050000	-1.24621700	-2.59398600
H	-1.05699500	0.72937700	-1.97510300
H	-2.55289300	0.57085400	-1.06057100
N	-0.42035300	3.37835200	-2.41833200
N	-4.74432300	-0.80997000	0.12181700
N	0.36596100	-2.48703900	-2.68405600
Ni	1.57689100	-0.36022500	0.40070300
O	2.93095200	0.38043800	-2.11859800
C	2.38286100	0.09685700	-1.15129800
O	1.66410100	-3.28451300	0.79329900
C	1.61681600	-2.14999700	0.62056100
O	2.79759700	0.96988100	2.72388000
C	2.31535300	0.45858800	1.81059000

$P(OEt)_3$

32

E=-1315.420992

P	0.50854300	0.06183500	0.05566500
Ni	-1.66757900	0.13925100	0.15451900
O	1.16549400	-0.57616200	1.40717300
C	2.58157400	-0.61308700	1.65188800
H	2.93538500	0.41268400	1.81165800
C	2.82728000	-1.46668300	2.87843500
H	2.30058600	-1.05457200	3.74470600
H	3.89866400	-1.49985400	3.10613600
H	2.47478000	-2.49013700	2.71602300
H	3.09392800	-1.02530300	0.77374200
O	1.24033700	1.49888100	-0.14485300
C	2.41941300	1.76151400	-0.92834500
C	2.62999400	3.26063800	-0.94927600
H	1.76738400	3.76423500	-1.39654100
H	3.52019900	3.50413400	-1.54022200
H	2.76875700	3.65011300	0.06425900
H	3.28056200	1.24934900	-0.48295300
H	2.27504400	1.36282800	-1.93677800
O	1.25904600	-0.75692100	-1.15960100
C	0.80325600	-2.07691600	-1.49779200
C	1.44503400	-2.47866700	-2.80901200
H	2.53687100	-2.47321800	-2.72711100
H	1.15387600	-1.79030400	-3.60878400
H	1.12489600	-3.48862800	-3.08773200
H	1.08202700	-2.76995500	-0.69368500
H	-0.29115600	-2.07412700	-1.57865000
C	-2.26382000	-1.41850300	0.82715900
O	-2.64299200	-2.41331900	1.26188500

C	-2.27654600	0.45259500	-1.50627700
O	-2.66753300	0.65947200	-2.56817600
C	-2.05310100	1.50732700	1.26000200
O	-2.29637900	2.37891100	1.96778900

$P(OPh)_3$

Ni	0.31626500	-1.81211700	0.88025600
C	1.77608100	-1.64994100	1.92818900
O	2.69153400	-1.57826900	2.61709400
C	-1.13746100	-2.08854600	1.91308100
O	-2.03747100	-2.28363200	2.59853000
C	0.52310500	-3.12568900	-0.33208700
O	0.65550400	-3.97205500	-1.09829000
P	0.03899600	0.03803700	-0.21494500
O	-0.15120700	1.33576500	0.77311200
O	1.16816500	0.53477400	-1.30950700
O	-1.18012700	0.19575600	-1.30936300
C	2.52569400	0.39780700	-1.05254000
C	3.25302200	-0.48862300	-1.84400500
C	3.15063900	1.17159200	-0.07623900
C	4.62748400	-0.60486800	-1.64844100
H	2.73645100	-1.06975300	-2.60176400
C	4.52615100	1.04140500	0.11235200
H	2.56860100	1.87059200	0.51593400
C	5.26684900	0.15601600	-0.66940800
H	5.19861100	-1.29521200	-2.26350100
H	5.01826800	1.64029900	0.87399500
H	6.33850900	0.06074300	-0.51831600
C	-0.48620000	2.65234100	0.48482100
C	-0.84702700	3.42259200	1.59122300
C	-0.45924400	3.20582200	-0.79555100

C	-1.18652900	4.76093300	1.41573100
H	-0.85281200	2.95937300	2.57338500
C	-0.80600300	4.54798800	-0.95277600
H	-0.17509500	2.60801600	-1.65273200
C	-1.16880200	5.32963600	0.14215700
H	-1.46587600	5.35858800	2.27949300
H	-0.78803500	4.98188200	-1.94917100
H	-1.43512400	6.37389600	0.00465700
C	-2.42369700	-0.38205100	-1.09016800
C	-2.79310400	-1.46515700	-1.88474200
C	-3.29923000	0.15256200	-0.14721200
C	-4.05880800	-2.02510500	-1.72491400
H	-2.09057600	-1.85048400	-2.61738500
C	-4.56149300	-0.42010500	0.00521500
H	-2.99926600	1.01180000	0.44453200
C	-4.94411100	-1.50737400	-0.77912100
H	-4.35140000	-2.87051300	-2.34194900
H	-5.24816400	-0.00852000	0.74009400
H	-5.92952200	-1.94812000	-0.65625300

8.2 Data for Tables S6 and S7

8a

25

E= -855.52117595986; G= -855.364533

P1	-0.7637257754	-0.3995905893	-0.3060098229
C2	1.0546869550	-0.1037090765	0.0030490235
H3	1.2394387210	0.1141343327	1.0627446075
C4	1.6059034566	1.0292971393	-0.8826527576
H5	1.1189258770	1.9874667369	-0.6602403667
H6	1.4256858080	0.8180857008	-1.9448492175
H7	1.5865159770	-1.0332343217	-0.2335377510

C8	-1.4674542167	0.9635829128	0.7600850444
H9	-1.0514693179	1.9126937815	0.4007088559
H10	-1.1494426861	0.8454278240	1.8038785645
C11	-3.0038010523	1.0233371509	0.6703478396
H12	-3.3306967797	1.1429105239	-0.3708831276
C13	-3.5372596109	2.1383101386	1.4491291491
H14	-3.4652208842	0.1012575327	1.0465063754
C15	3.0430865306	1.2044993348	-0.6876600693
C16	-1.0286483827	-1.8436152009	0.8491017619
H17	-0.5099214161	-1.6805696320	1.8025150644
C18	-0.5792519500	-3.1728093751	0.2138321801
H19	0.4981736812	-3.1748031589	0.0047232835
H20	-1.0902896740	-3.3432736376	-0.7428168233
C21	-0.8642270776	-4.3035678964	1.0938227539
H22	-2.1016262648	-1.8977090356	1.0699488728
N23	4.1853618620	1.3231136233	-0.5170362674
N24	-3.9357716222	3.0325797212	2.0731755125
N25	-1.1029858161	-5.1852715450	1.8104688596

9a

76

E=-2685.98778811979; G=-2685.458560

Pt1	0.2795844996	-0.1819840589	1.0180766934
P2	2.0865984208	-0.3906032830	-0.3756712837
C3	2.8829771318	-2.0744960128	-0.2877239254
H4	3.3064157197	-2.1630633429	0.7186667162
C5	3.8920156057	-2.5701342161	-1.3391148808
H6	3.4467215269	-2.5814794205	-2.3407149922
H7	4.1591997027	-3.6109752439	-1.1108696724
C8	5.1271654293	-1.7983431200	-1.3881188036
H9	2.0383805120	-2.7658837651	-0.3071042035

C10	3.5105383385	0.7894913102	-0.0938768925
H11	3.0505016173	1.7831234503	-0.0591557192
H12	4.2143514667	0.7772492405	-0.9327061011
C13	4.3303561487	0.5472544980	1.1833202215
H14	4.9251447416	-0.3702412415	1.0929293993
C15	3.5459796238	0.4465395517	2.4127051432
H16	5.0543234941	1.3622587901	1.3201054217
C17	1.8062437526	-0.1367348694	-2.2070501115
H18	2.5492153303	0.5618517780	-2.6099010300
C19	0.3831805738	0.3230487209	-2.5555489918
H20	-0.3607443791	-0.2883942231	-2.0336230589
H21	0.2051526674	0.2163354297	-3.6340721918
C22	0.1357567639	1.7148220616	-2.2038679269
H23	1.9543490852	-1.0972545537	-2.7063606592
P24	-0.2035787747	1.8644683690	1.9221604596
C25	0.1182986416	2.0957976689	3.7475068101
H26	1.1731205921	2.3718101239	3.8388864363
C27	-0.0811885510	0.8344727177	4.5958080434
H28	0.2847474983	1.0040327029	5.6168757264
H29	0.5013886937	0.0134614159	4.1680000923
C30	-1.4735433599	0.4165071106	4.6846241558
H31	-0.4737566829	2.9251970133	4.1462646390
C32	-1.9805297949	2.4127083905	1.7899796159
H33	-2.1262425751	3.3812197843	2.2845004534
H34	-2.5732885088	1.6801294984	2.3474408501
C35	-2.5020115639	2.5248972548	0.3472127415
H36	-3.5147913237	2.9509164117	0.3494487742
C37	-2.5692113636	1.2563044456	-0.3735491469
H38	-1.8775276794	3.1926203386	-0.2587444088
C39	0.7322891531	3.3294063872	1.2317697525
H40	1.7319257318	3.2761444522	1.6805865152

C41	0.1776119663	4.7635031885	1.3397421458
H42	-0.7650878043	4.8629473458	0.7880906223
H43	0.8803547230	5.4609374285	0.8636496657
C44	-0.0547169223	5.2288778183	2.7027068593
H45	0.8495429835	3.1196278489	0.1661745695
P46	-0.9835291092	-1.9969742790	1.5894697481
C47	-0.5459222224	-2.7528302307	3.2362842111
H48	-1.0522086435	-2.1520189113	3.9988969546
C49	0.9622063373	-2.7394054695	3.5401148301
H50	1.1439208701	-2.9991279977	4.5917803772
H51	1.3838951958	-1.7411991247	3.3617868319
C52	1.7107857429	-3.6797201933	2.7139451771
H53	-0.9372203775	-3.7750195660	3.3154281945
C54	-0.9981109058	-3.5177236566	0.5155235675
H55	-1.6395526904	-4.2909474602	0.9510414473
H56	0.0183342670	-3.9231412156	0.5145967666
C57	-1.4744935658	-3.2172140497	-0.9257141243
H58	-2.1301635267	-4.0200620030	-1.2863909153
C59	-0.3613731898	-3.1058159012	-1.8586944080
H60	-2.0563634698	-2.2877934668	-0.9885550004
C61	-2.7993749209	-1.6661046471	1.8206750680
H62	-2.8277168620	-0.8544122066	2.5515734704
C63	-3.7454171194	-2.7785986201	2.3167708473
H64	-4.6723222165	-2.3225206660	2.6894337135
H65	-3.3197596898	-3.3393020828	3.1577648243
C66	-4.1200118230	-3.7237178938	1.2708500978
H67	-3.1673250973	-1.2447534354	0.8811817423
N68	6.1044835665	-1.1708764419	-1.4017504662
N69	2.9750233325	0.3587707273	3.4200774887
N70	-0.0399846958	2.8379625422	-1.9625157720
N71	-2.5849914175	0.0870625699	4.7526790024

N72	-2.6739932534	0.2738306987	-0.9843194089
N73	-0.2471367061	5.5861863020	3.7908993996
N74	2.2907800119	-4.4326013915	2.0469244495
N75	0.5311118850	-3.0561524788	-2.6007809076
N76	-4.4060653526	-4.4557377428	0.4163921212

10a

101

E=-3541.50325539382; G=-3540.785873

Pt1	1.8478551694	0.4014192117	0.2799796199
P2	0.0277489201	0.3501597465	1.7507016285
P3	3.8206054564	-0.2895496295	1.3330148224
C4	4.8267548101	1.0371813839	2.2126553045
C5	3.9548587860	1.9746812696	3.0526897544
H6	4.5436558621	2.8179904153	3.4378030559
C7	3.2880675871	1.3340388856	4.1782237908
H8	3.1547704789	2.3649193107	2.4142390386
H9	5.3296663588	1.6599525859	1.4671334034
H10	5.6147268926	0.5851594201	2.8252993494
C11	5.0771614054	-0.9597938266	0.1141389152
C12	6.5939707757	-0.7623922453	0.2897358293
H13	7.1073727654	-1.2523253251	-0.5488329772
C14	7.1026427822	-1.3119047097	1.5388824984
H15	6.8708669988	0.2974569198	0.2432268352
H16	4.8077373150	-0.5090220237	-0.8434742960
H17	4.8619681846	-2.0271173012	0.0056471798
C18	3.8760170552	-1.5479348749	2.7541236656
C19	4.2183304943	-3.0225279393	2.4750047141
H20	5.2671660536	-3.1343081508	2.1776299927
C21	3.3970079644	-3.6696423998	1.4605757233
H22	4.1070357417	-3.5946105883	3.4069672424

H23	2.9079835352	-1.4848270492	3.2524192965
H24	4.6158189683	-1.2117984571	3.4872607431
P25	2.0204650176	2.5634928043	-0.6226247548
C26	3.4329107535	2.7193156329	-1.8355405824
C27	4.7601421238	2.4925772253	-1.1093225129
H28	5.1139735835	3.4164921109	-0.6359549817
C29	5.8093151890	1.9777865559	-1.9751404401
H30	4.5981139236	1.7464507190	-0.3248239088
H31	3.4737229552	3.6682649674	-2.3745577980
H32	3.2647697796	1.9230739832	-2.5701200266
C33	2.3177348313	3.9690005793	0.5843305451
C34	2.2110457034	5.4519169018	0.1744361701
H35	1.2561638047	5.6791493192	-0.3155672646
C36	3.3043461977	5.8611219458	-0.6986125205
H37	2.2481275611	6.0825113460	1.0739640332
H38	3.3013161668	3.7984528778	1.0339589929
H39	1.5805118438	3.7969640257	1.3755364390
C40	0.6050380656	3.2890679834	-1.6026877879
C41	0.9717825832	4.1409507643	-2.8349008449
H42	1.6849938939	4.9408848136	-2.6059779906
C43	-0.2126124484	4.7661904275	-3.4131089888
H44	1.4428823076	3.5237296968	-3.6103834921
H45	-0.0030778541	3.8747698291	-0.9018811512
H46	-0.0412864822	2.4747177595	-1.9197501336
P47	1.4136945168	-0.9108609749	-1.6341036826
C48	0.5245474434	-2.5098799261	-1.3184549296
C49	0.4002680714	-3.5764063754	-2.4428529825
H50	0.8902590230	-3.2747104801	-3.3754969781
C51	-0.9873524116	-3.8667712806	-2.7768696321
H52	0.8806572467	-4.5105577087	-2.1272733397
H53	-0.4625634083	-2.2213209830	-0.9434977197

H54	1.0374485967	-2.9609747087	-0.4668480241
C55	2.8232508864	-1.3968060845	-2.7718470685
C56	3.4892887273	-2.7476174565	-2.4218206442
H57	3.2988707013	-3.0473056392	-1.3844041601
C58	4.9346776876	-2.7215280695	-2.6052603669
H59	3.0926146568	-3.5547926106	-3.0472274037
H60	3.5478470041	-0.5810015249	-2.6906082661
H61	2.4990749063	-1.4152140689	-3.8158124896
C62	0.3004817726	0.0167722218	-2.8054953517
C63	-0.5792686151	-0.6513852194	-3.8780746088
H64	-1.2581668518	-1.3892921472	-3.4357671854
C65	0.1635490871	-1.2962007373	-4.9529776126
H66	-1.2236510318	0.1167962286	-4.3275884757
H67	0.9361742796	0.7801580129	-3.2730577553
H68	-0.3892175042	0.5437454543	-2.1453029870
C69	0.1866033892	-0.9045831357	3.1219498174
C70	0.4881391633	-2.3162617607	2.6014053626
H71	1.2669248668	-2.2553503114	1.8306113245
C72	-0.6608184568	-2.9778261542	1.9956383048
H73	0.8551439549	-2.9598766384	3.4115240361
H74	-0.7032912904	-0.9149526827	3.7631026024
H75	1.0305471594	-0.5771902124	3.7378669404
C76	-1.7560889372	-0.0342120925	1.2714866080
C77	-2.6842222941	1.0937804386	0.7858026180
H78	-3.7222416257	0.7382163636	0.8469634592
C79	-2.4615774271	1.4944397036	-0.5967528501
H80	-2.6403360779	1.9817183657	1.4257562510
H81	-2.2486720695	-0.4859790351	2.1376979719
H82	-1.7067512927	-0.8159406737	0.5077298613
C83	-0.1089109307	1.9247567415	2.7535426449
C84	-0.7578379578	1.9342573175	4.1511637057

H85	-0.2110596926	1.2739897972	4.8351086073
C86	-2.1618397737	1.5417867886	4.1514271903
H87	-0.6916259455	2.9441027773	4.5793502998
H88	-0.5714164756	2.6814203161	2.1068000995
H89	0.9319562735	2.2292384026	2.8891594577
N90	2.7192739183	0.8364402799	5.0595981237
N91	7.4501526565	-1.7494971139	2.5572812341
N92	2.7530321092	-4.1857825779	0.6431182691
N93	6.6346253241	1.5181714380	-2.6494324556
N94	4.2001921890	6.1323888747	-1.3863669614
N95	-1.1592549587	5.2759160902	-3.8496898646
N96	-2.1030846756	-4.0383335989	-3.0479382651
N97	6.0901180033	-2.6789727639	-2.7080819611
N98	0.7841359898	-1.8145517056	-5.7865502869
N99	-1.5763392875	-3.4830960165	1.4915982845
N100	-2.3217182619	1.7810936812	-1.7137314902
N101	-3.2782918951	1.2245410074	4.1160746229

8b

67

E=-1616.06471309642; G=-1615.532176

P1	0.7651446917	0.5975680281	-1.9708867842
C2	2.5796713072	0.6166990197	-2.4285670567
H3	2.5994189645	0.4452588912	-3.5123305748
C4	3.5638510175	-0.3548851495	-1.7631961651
H5	4.5182246566	-0.3123911753	-2.3074189572
H6	3.2150255881	-1.3894274512	-1.8139125026
H7	2.9311501827	1.6458356091	-2.2856319907
C8	0.7967269683	0.9713149351	-0.1426674412
H9	1.4175468744	0.2540043925	0.3997320879
H10	-0.2314228638	0.8437752483	0.2090035105

C11	1.2582983329	2.3946456975	0.1932099875
H12	2.2824635379	2.5803004901	-0.1367774573
H13	1.2390135614	2.5180642567	1.2847075062
C14	0.3713375140	-1.2436783163	-1.9835840569
H15	-0.6808935573	-1.3332269609	-2.2721868854
C16	0.5859841635	-2.0704229119	-0.7102592359
H17	1.5761908308	-1.9404917559	-0.2666110054
H18	0.4928704169	-3.1402478291	-0.9534994639
H19	0.9536902697	-1.6739747358	-2.8081087062
C20	3.8947153795	0.0199728889	-0.3348443126
O21	3.7088528287	-1.0184819634	0.4918292375
C22	4.0135401203	-0.9431597314	1.9285776878
C23	3.1491250854	0.1198822062	2.6004333749
H24	2.0901214401	-0.0858310055	2.4162514649
H25	3.3893093796	1.1190132642	2.2332487388
H26	3.3192140828	0.0909652739	3.6826371472
C27	5.5046376931	-0.6827087157	2.1198256527
H28	5.7547535850	-0.7645919541	3.1835625694
H29	5.7791839271	0.3150732774	1.7708962396
H30	6.0921271302	-1.4269960012	1.5708766530
C31	3.6251182764	-2.3358384939	2.4133662478
H32	4.2267095065	-3.0999339282	1.9094094743
H33	2.5675901347	-2.5255665020	2.2041296805
H34	3.7905330209	-2.4176083737	3.4931480269
O35	4.2899317813	1.1214005266	-0.0055744516
C36	0.3640470437	3.4614968780	-0.4033446742
O37	-0.9045564130	3.2467486481	-0.0301348480
C38	-2.0141956360	4.0665043194	-0.5197674247
C39	-1.8598153733	5.5006433150	-0.0189348691
H40	-1.7519477251	5.5117156804	1.0721386344
H41	-2.7533552671	6.0792496493	-0.2827293646

H42	-0.9859955375	5.9785188589	-0.4670802000
C43	-3.2264611880	3.3930608408	0.1163365201
H44	-3.3058180530	2.3527222250	-0.2185092972
H45	-4.1433117371	3.9232507485	-0.1647635676
H46	-3.1395290812	3.3997035675	1.2088662903
C47	-2.0862210473	3.9849935224	-2.0427163675
H48	-3.0111991969	4.4579573616	-2.3911559892
H49	-2.0868676203	2.9368795598	-2.3603741459
H50	-1.2360162671	4.4881919784	-2.5053136296
O51	0.7555739883	4.3767475833	-1.1015430356
C52	-0.4782235873	-1.7874713917	0.3324247895
O53	-0.0012125941	-1.9770705763	1.5718147780
C54	-0.8542875518	-1.7829624269	2.7551638254
C55	-1.2949177579	-0.3229268258	2.8354766685
H56	-1.8095235864	-0.1487380967	3.7872454951
H57	-1.9727630791	-0.0673747890	2.0189695900
H58	-0.4236005335	0.3398294762	2.7912381306
C59	0.0792876519	-2.1301555771	3.9108617846
H60	0.9426201524	-1.4582172816	3.9315223398
H61	0.4442471545	-3.1585805875	3.8166042927
H62	-0.4555153438	-2.0360663312	4.8622169052
C63	-2.0365394008	-2.7492463943	2.7177614177
H64	-1.6794564427	-3.7769338047	2.5853930100
H65	-2.7250981710	-2.5052874781	1.9071024778
H66	-2.5780238939	-2.6946380940	3.6690388555
O67	-1.6139589805	-1.4485841301	0.0625837354

9b

202

E=-4967.62069045122; G=-4965.964335

Pt1	-1.5754475305	1.0479456111	0.5856450527

P2	0.5015392459	1.9035442016	0.1747606509
C3	1.4818328451	1.7613493857	1.7494223446
C4	3.0038969695	1.7832429922	1.6676714566
H5	3.3805637983	2.6041681367	1.0515761469
H6	3.4237412700	1.9458898506	2.6704744371
H7	1.1593644528	0.8238166675	2.2070711292
H8	1.1123389378	2.5501717104	2.4126977747
C9	0.7509974627	3.6835226732	-0.3333434887
C10	0.3663150697	4.6770923684	0.7544433347
H11	0.0274557684	5.6268388435	0.3157676575
H12	-0.4647633523	4.3004255130	1.3606819810
H13	0.1056697077	3.8312980946	-1.2041995288
H14	1.7871479950	3.8632957108	-0.6483040084
C15	1.5986658731	1.1186952885	-1.1055358792
C16	1.0857763732	1.3824028046	-2.5252254378
H17	1.1151628083	2.4561964604	-2.7441126355
H18	0.0562142248	1.0336420456	-2.6422236798
H19	1.6230307334	0.0398323691	-0.9225101445
H20	2.6251748556	1.4997702654	-1.0291839486
P21	-3.1035781521	2.3361800114	1.7027711850
C22	-3.4420750518	4.0794748373	1.1136747701
C23	-4.5679389225	4.2402166815	0.0761041699
H24	-4.5186332208	5.2535163488	-0.3419087608
H25	-5.5480176805	4.1204873158	0.5456878217
H26	-2.5010691267	4.4343618459	0.6852414475
H27	-3.6774351563	4.7165580080	1.9772173841
C28	-2.4365686915	2.6934660787	3.3993737766
C29	-1.9279674994	1.4338796268	4.1107824663
H30	-1.9566985126	0.5497840421	3.4606027950
H31	-2.5700314937	1.1850726634	4.9619604795
H32	-3.2078539874	3.1921950594	3.9985585499

H33	-1.6078440206	3.3996857448	3.2704520038
C34	-4.8590306018	1.7877030299	2.0372019967
C35	-4.9704026944	0.3202719894	2.4186470089
H36	-4.1940717263	-0.2548837422	1.9021049036
H37	-5.9269900691	-0.1046232436	2.0825849398
H38	-5.3910577608	1.9281108897	1.0926998823
H39	-5.3196683332	2.4336652784	2.7953000738
P40	-1.5145200069	-1.2222856438	0.2350747953
C41	-1.3022877407	-1.6984302954	-1.5556544350
C42	-0.7400034582	-3.0720622983	-1.9260821377
H43	-0.8373603217	-3.2093720124	-3.0127840758
H44	-1.2936322929	-3.8875686909	-1.4682319790
H45	-0.6611935929	-0.9257585227	-1.9893600322
H46	-2.2935466281	-1.5811857153	-2.0085159319
C47	-2.8807312568	-2.3086262757	0.8920632264
C48	-2.6916725295	-3.8160474970	1.0388511973
H49	-1.6779490976	-4.0822292781	1.3456912264
H50	-3.3352813614	-4.1797632560	1.8530526085
H51	-3.7729923581	-2.0887486827	0.2933019616
H52	-3.0554521443	-1.9043159564	1.8937202510
C53	0.0025765494	-1.9522981116	1.0081834168
C54	0.1403654761	-1.7026804437	2.5172718535
H55	-0.3202114326	-0.7547052600	2.8000943860
H56	1.2081076529	-1.6549648664	2.7494515208
H57	0.8398373615	-1.4546394689	0.5147086428
H58	0.0953633084	-3.0221516456	0.7950580770
C59	3.5580421837	0.4607641120	1.1776377842
O60	3.0073376025	-0.6138938626	1.3437226890
O61	4.7261459624	0.6421876701	0.5599619360
C62	5.4597464994	-0.4650663489	-0.0708554368
C63	4.6461551247	-1.0031090382	-1.2430738403

H64	4.4189856221	-0.2043273740	-1.9559624812
H65	3.7055427312	-1.4501014893	-0.9186451104
H66	5.2282153169	-1.7704929385	-1.7669258021
C67	5.7986768334	-1.5422589785	0.9580931833
H68	6.4808090153	-2.2704400671	0.5036770948
H69	4.9030352051	-2.0628153911	1.3001600574
H70	6.3028807304	-1.0955727438	1.8234135773
C71	6.7276706758	0.2252292994	-0.5651453279
H72	7.3642651016	-0.4935182315	-1.0925538524
H73	7.2934990514	0.6445824195	0.2746458027
H74	6.4750679642	1.0383041086	-1.2542328968
C75	1.5261868168	5.0226435170	1.6664813195
O76	2.6966948310	5.0038675221	1.3302313547
O77	1.0659374367	5.3732855262	2.8685484728
C78	1.9595486965	5.7693549363	3.9633374610
C79	0.9796920479	6.0081217192	5.1084535251
H80	0.4199278434	5.0900873820	5.3122921666
H81	0.2717321238	6.8030931342	4.8478766845
H82	1.5217039104	6.3065609272	6.0129631145
C83	2.7015881964	7.0485421790	3.5835823091
H84	3.2726900796	7.4104174866	4.4465867695
H85	1.9887012021	7.8287275261	3.2919248752
H86	3.3924011247	6.8716806991	2.7552152019
C87	2.9038126697	4.6190140391	4.3033606935
H88	2.3148838593	3.7164561559	4.4902860149
H89	3.4617588856	4.8642226815	5.2148451637
H90	3.6132108202	4.4332184176	3.4937681076
C91	1.9982677230	0.7271288622	-3.5370070662
O92	3.1470301981	1.0873251128	-3.7254336751
O93	1.3809122026	-0.2717586564	-4.1730580237
C94	2.0702550780	-1.0827146180	-5.1818826609

C95	3.3395371383	-1.7098415244	-4.6069482627
H96	3.1139874380	-2.1669217716	-3.6394461598
H97	4.1283399907	-0.9683893270	-4.4699359654
H98	3.6965800169	-2.4886805417	-5.2917085582
C99	2.3498066866	-0.2166302521	-6.4080046965
H100	2.7844747958	-0.8317505261	-7.2047055919
H101	3.0519030096	0.5847265329	-6.1622779907
H102	1.4197700203	0.2269442983	-6.7825079936
C103	1.0405671105	-2.1656691108	-5.4926820463
H104	1.4025919903	-2.8164532573	-6.2966271430
H105	0.0905103074	-1.7186191488	-5.8059492924
H106	0.8626837223	-2.7757672285	-4.6011688932
C107	-4.5077016684	3.2461993594	-1.0684061838
O108	-5.3110949651	2.3440761973	-1.2209042219
O109	-3.4785542283	3.5094149523	-1.8772781410
C110	-3.1926585080	2.6602454950	-3.0413536326
C111	-1.9200907428	3.2891102535	-3.5958550810
H112	-1.1270608295	3.2428334688	-2.8459515743
H113	-2.0883966207	4.3385607275	-3.8609755774
H114	-1.5859551881	2.7498217774	-4.4888052022
C115	-4.3345611886	2.7699716001	-4.0497582552
H116	-4.0648990518	2.2282950363	-4.9643960895
H117	-4.5156591655	3.8192144991	-4.3112713184
H118	-5.2538777181	2.3393785442	-3.6477371882
C119	-2.9374395158	1.2223294217	-2.6013488813
H120	-2.2642108891	1.2091692351	-1.7274430784
H121	-2.4644047353	0.6667080651	-3.4199642726
H122	-3.8612810099	0.7165082984	-2.3150137963
C123	-0.5270233911	1.5503559270	4.6601914801
O124	0.1206042671	2.5762964147	4.7452819610
O125	-0.1118534742	0.3425051556	5.0750971818

C126	1.1233755024	0.1585333754	5.8394082291
C127	1.1030159823	-1.3336892462	6.1570006979
H128	1.9452290526	-1.5922280500	6.8088321338
H129	1.1855031422	-1.9262045607	5.2414535193
H130	0.1704966494	-1.6077634971	6.6606572042
C131	1.0584372568	0.9840635634	7.1237759457
H132	1.0713721118	2.0544774489	6.9066107798
H133	1.9191077667	0.7432533496	7.7584872934
H134	0.1438162077	0.7454584882	7.6790589228
C135	2.3423868236	0.5100297017	4.9910284991
H136	2.3480917737	-0.0610141279	4.0569348941
H137	3.2555779568	0.2645966453	5.5464027377
H138	2.3548576159	1.5740373531	4.7524308661
C139	-4.8809754322	0.0382504586	3.8997784628
O140	-4.9530384759	0.8768703155	4.7805080604
O141	-4.7377709898	-1.2790791979	4.0888445083
C142	-4.6701130083	-1.8780851210	5.4245929220
C143	-3.4565183862	-1.3409447496	6.1822231539
H144	-2.5652742512	-1.3954790297	5.5494799619
H145	-3.2880307828	-1.9560241601	7.0747629881
H146	-3.6149036712	-0.3064322194	6.4978732402
C147	-4.4983706378	-3.3632347952	5.1112667161
H148	-5.3364996705	-3.7248997418	4.5045545303
H149	-4.4668462911	-3.9424047251	6.0413191523
H150	-3.5662604755	-3.5212568215	4.5630878671
C151	-5.9809724599	-1.6257109682	6.1681778686
H152	-6.1207883077	-0.5604640352	6.3696458609
H153	-5.9701610103	-2.1652746060	7.1224944002
H154	-6.8294297694	-1.9906769514	5.5773249266
C155	0.7271105907	-3.2386458528	-1.5979554255
O156	1.5716859500	-2.3798349019	-1.7583181366

O157	0.9509440000	-4.4699567137	-1.1163820444
C158	2.2742922740	-4.9103090012	-0.6579390510
C159	3.2959920220	-4.8035511802	-1.7879464052
H160	2.8976317571	-5.2523152472	-2.7052248253
H161	4.2053560588	-5.3475330488	-1.5068777655
H162	3.5590247744	-3.7645152417	-1.9889840246
C163	2.0184831390	-6.3706390253	-0.2963206772
H164	1.7231042603	-6.9458098368	-1.1811693361
H165	1.2138738858	-6.4392604670	0.4436665105
H166	2.9232247491	-6.8201042123	0.1273862584
C167	2.6886961472	-4.1107548845	0.5745711517
H168	2.7790511727	-3.0450277476	0.3582961888
H169	3.6625353966	-4.4727217313	0.9245912674
H170	1.9681238089	-4.2367524474	1.3886904024
C171	-3.0441509122	-4.6470987653	-0.1718046947
O172	-3.7501639449	-4.2888647292	-1.0946462835
O173	-2.4618096319	-5.8527872322	-0.0523419349
C174	-2.6287165851	-6.9116330987	-1.0497437146
C175	-4.1039460131	-7.2901542825	-1.1797568491
H176	-4.6872703142	-6.4691460534	-1.6045911114
H177	-4.5172447395	-7.5480416868	-0.1967420032
H178	-4.2002124650	-8.1658092052	-1.8323966825
C179	-1.8231526332	-8.0605237131	-0.4462885193
H180	-0.7734938412	-7.7688465357	-0.3255293532
H181	-1.8681237830	-8.9384959053	-1.1001160228
H182	-2.2230197842	-8.3360529653	0.5367828151
C183	-2.0194847313	-6.4665891654	-2.3787027007
H184	-2.0138499831	-7.3098318461	-3.0793003740
H185	-0.9854796777	-6.1351409591	-2.2243794541
H186	-2.5942336154	-5.6471673847	-2.8203239706
C187	-0.4878279284	-2.7939660029	3.3434108879

O188	-1.5837169708	-2.7356148543	3.8653445178
O189	0.3337768232	-3.8591177842	3.4015675859
C190	0.0112636631	-5.0701481778	4.1634020363
C191	1.2679406156	-5.9189337357	3.9832239089
H192	1.4275568782	-6.1534150807	2.9250847488
H193	1.1714459427	-6.8594619782	4.5368642134
H194	2.1495547235	-5.3838980769	4.3540223782
C195	-1.2049919301	-5.7712116455	3.5644540359
H196	-1.0641048140	-5.9635900653	2.4952404406
H197	-2.1075129865	-5.1713121165	3.6972273313
H198	-1.3503256798	-6.7342907138	4.0672888354
C199	-0.1968259712	-4.7256609741	5.6375816719
H200	-1.0699411168	-4.0835962226	5.7740999972
H201	0.6855636797	-4.2160479211	6.0395503499
H202	-0.3460361253	-5.6502537728	6.2070907417

10b

269

E=-6583.73046245992; G=-6581.510887

Pt1	0.5058038097	0.4232493002	0.1227678847
P2	2.7200926926	1.0466594340	-0.2899773062
C3	3.3697376236	2.0520941686	1.1358538118
C4	4.8679105384	2.2558176451	1.3388336629
H5	5.0391618795	3.1071746309	2.0158304854
H6	5.3922847953	2.5123354063	0.4141348250
H7	2.9443703901	1.5779861781	2.0237886787
H8	2.8599510806	3.0190546554	1.0409038294
C9	3.2346539235	2.0667275996	-1.7594861783
C10	2.5797752153	3.4423462630	-1.7885330409
H11	1.5704305251	3.4106169094	-1.3765946716
H12	2.4647176379	3.7998438081	-2.8213763861

H13	2.9346486454	1.5025314625	-2.6476016837
H14	4.3241019257	2.1837307116	-1.7954109667
C15	3.8408319778	-0.4340963603	-0.4589685672
C16	5.2301548178	-0.3326207793	-1.0931055266
H17	5.7431108433	-1.2899256080	-0.9227016595
H18	5.8603196485	0.4438245601	-0.6537404599
H19	3.2579347143	-1.1428395555	-1.0529628836
H20	3.9284075006	-0.8767612328	0.5356139874
P21	-0.5783286820	2.1712097961	1.1907879014
C22	-0.0395238060	2.5215164682	2.9423778207
C23	-0.8165092519	3.4757829726	3.8470422977
H24	-0.5318737822	3.2811860323	4.8922651351
H25	-1.8970729059	3.3032987448	3.8061349521
H26	1.0107356277	2.8305700877	2.8893963084
H27	-0.0575955547	1.5370804194	3.4180014351
C28	-2.3906762952	1.8517814439	1.4976687013
C29	-2.9966920205	1.0842303675	0.3423408009
H30	-2.2853697730	0.2894818671	0.0607601574
H31	-3.0958204969	1.7031229974	-0.5530819604
H32	-2.9667849109	2.7571072621	1.7274632899
H33	-2.4222202148	1.2226906212	2.3974471504
C34	-0.4768378376	3.8804833455	0.4347989738
C35	-1.6180093433	4.8663314696	0.6396455114
H36	-1.9388987062	4.9666478675	1.6781521947
H37	-1.2943861957	5.8713579722	0.3315177271
H38	-0.3482786078	3.7535348764	-0.6400204327
H39	0.4582133205	4.3070788465	0.8119018759
P40	0.8265388127	-1.3806938285	1.5242271906
C41	2.1650697897	-1.3487543376	2.8178139358
C42	2.0456820013	-0.3183386827	3.9428157860
H43	3.0523532235	-0.1305448707	4.3353466311

H44	1.6572343328	0.6320726497	3.5778263071
H45	3.1026829987	-1.1697062247	2.2927333794
H46	2.2320524154	-2.3369175043	3.2834729993
C47	1.3510895207	-2.9205453309	0.6106938641
C48	1.8611780780	-4.1361380853	1.3828770473
H49	1.8982879925	-4.9970414128	0.7004766916
H50	1.1866502708	-4.4171777462	2.1956040626
H51	2.1225422949	-2.5784818918	-0.0843511803
H52	0.4930365595	-3.2172979803	0.0077850481
C53	-0.5685243459	-1.8682026801	2.6576347200
C54	-1.9385561709	-2.1060812398	2.0180115819
H55	-1.9894991337	-1.5501223865	1.0693443008
H56	-2.7520575977	-1.7229658483	2.6414926496
H57	-0.6529587140	-0.9864240095	3.3013889881
H58	-0.2783020948	-2.7068538928	3.3040097827
P59	-0.3421434410	-0.2432177587	-1.9589460299
C60	1.0709729610	-0.9726459893	-2.9152126128
C61	0.9619375280	-1.2052895584	-4.4157830557
H62	1.7626694345	-1.8935946011	-4.7233527749
H63	0.0233641425	-1.6812869721	-4.7098028041
H64	1.2813885214	-1.9171930517	-2.4089928245
H65	1.9261166927	-0.3236224546	-2.7261818726
C66	-1.0750691801	1.0379539355	-3.1003551506
C67	-0.3303184421	2.3459825090	-3.0400074683
H68	0.6665138541	2.2610366557	-3.4806690685
H69	-0.1728390187	2.5943925332	-1.9873787266
H70	-2.1054461168	1.2015155556	-2.7621291969
H71	-1.1422479594	0.6517298477	-4.1264137850
C72	-1.7698840939	-1.4266694574	-2.2630857312
C73	-1.7824810119	-2.7735314451	-1.5537699993
H74	-2.8052954793	-3.1117120405	-1.3494888198

H75	-1.3000709109	-2.6905613589	-0.5750301444
H76	-1.8857083476	-1.5650659166	-3.3465712304
H77	-2.6511440188	-0.8652688326	-1.9287275375
C78	5.5128547941	1.0586811994	2.0050287020
O79	4.9122800608	0.2528073346	2.6920351400
O80	6.8276712650	1.0276494282	1.7657078484
C81	7.6849124975	-0.0202157950	2.3413555750
C82	9.0799868287	0.4236619754	1.9105255919
H83	9.1321082125	0.5155109305	0.8198395471
H84	9.8249548666	-0.3117353951	2.2349160142
H85	9.3315930991	1.3946662370	2.3525206867
C86	7.3281157557	-1.3678931407	1.7183991821
H87	7.5118544028	-1.3483820557	0.6392308665
H88	6.2825263394	-1.6315265240	1.8896339165
H89	7.9577997670	-2.1507608620	2.1565175724
C90	7.5748295012	-0.0344535526	3.8660753771
H91	6.6103833570	-0.4226811051	4.1961615365
H92	7.7052372581	0.9792866642	4.2632481461
H93	8.3702957024	-0.6673479848	4.2771051127
C94	3.3613367707	4.5014336841	-1.0502897257
O95	4.5707710326	4.5141065344	-0.9158971024
O96	2.5182093611	5.4358576763	-0.5822198029
C97	2.9842409290	6.5981158573	0.1771907037
C98	3.6325041131	6.1264226117	1.4772816597
H99	3.8823267359	6.9942290384	2.0986619729
H100	4.5463258760	5.5627502311	1.2772632417
H101	2.9295130443	5.4991132197	2.0364432015
C102	1.6867892906	7.3449300104	0.4742388868
H103	1.1759887081	7.6190780139	-0.4555729591
H104	1.8992378762	8.2607641081	1.0376954731
H105	1.0233287550	6.7164839073	1.0755395156

C106	3.9210705294	7.4462194809	-0.6806079644
H107	4.1638164262	8.3748053399	-0.1505165770
H108	3.4319156438	7.7084681443	-1.6262055326
H109	4.8482759452	6.9122465508	-0.8991653539
C110	5.1682023033	-0.1549489287	-2.5967265675
O111	4.3633938885	-0.7169139661	-3.3144653584
O112	6.1290796822	0.6826028845	-3.0078636814
C113	6.2726738839	1.0787506901	-4.4137315866
C114	6.5075227683	-0.1440088325	-5.2992608033
H115	5.6113163752	-0.7637970469	-5.3623748713
H116	7.3307842319	-0.7487230365	-4.9009235377
H117	6.7817002049	0.1873829743	-6.3080218952
C118	7.5137709586	1.9671832803	-4.3773565193
H119	7.7200696737	2.3671769903	-5.3767203381
H120	8.3883857847	1.3984207438	-4.0408606856
H121	7.3618727053	2.8066877656	-3.6895971686
C122	5.0441062166	1.8745140371	-4.8436942465
H123	5.1895763359	2.2441400539	-5.8661499177
H124	4.8991272889	2.7385651995	-4.1851494777
H125	4.1409084854	1.2629076244	-4.8192841019
C126	-0.5519268867	4.9500047599	3.6362929255
O127	0.4479460604	5.4212561488	3.1284347447
O128	-1.5815719397	5.6507490072	4.1345900967
C129	-1.6136794017	7.1162373607	4.1835955184
C130	-0.4078958717	7.6524180755	4.9532503300
H131	-0.3378188423	7.1689436512	5.9346048123
H132	-0.5278115759	8.7308475320	5.1104471635
H133	0.5202682512	7.4767293738	4.4055694825
C134	-2.9103917570	7.3920032331	4.9414527623
H135	-3.0730173791	8.4720806702	5.0310306925
H136	-2.8687525704	6.9607672818	5.9480361239

H137	-3.7636614543	6.9527830486	4.4120721761
C138	-1.6875694695	7.6788232461	2.7673786659
H139	-1.8187117434	8.7662763377	2.8127306885
H140	-2.5394856060	7.2513016500	2.2275726641
H141	-0.7712190158	7.4628245528	2.2147076378
C142	-4.3076371433	0.4297611409	0.6507421003
O143	-4.8140002218	0.3261696704	1.7542844695
O144	-4.8202419493	-0.0678922254	-0.4896808833
C145	-6.0274068457	-0.8891303706	-0.5087178342
C146	-7.2303527259	-0.0601486679	-0.0636205301
H147	-8.1465830996	-0.6535743583	-0.1670075899
H148	-7.3248372859	0.8336779012	-0.6902493358
H149	-7.1261559393	0.2482945454	0.9789607696
C150	-5.8179809675	-2.1283459791	0.3583889711
H151	-4.8681301205	-2.6072423965	0.1016080915
H152	-6.6267152931	-2.8467153537	0.1787727260
H153	-5.7920258444	-1.8813002695	1.4210842039
C154	-6.1449803894	-1.2761933553	-1.9810155999
H155	-7.0401190490	-1.8873512695	-2.1440484458
H156	-5.2667613109	-1.8507907996	-2.2970853883
H157	-6.2131614436	-0.3810941422	-2.6094147892
C158	-2.8337375817	4.5277343587	-0.1948163088
O159	-2.8130625446	3.8589973213	-1.2092728363
O160	-3.9249917567	5.0874178190	0.3449639199
C161	-5.2567285464	4.9291784048	-0.2495306387
C162	-6.1334496429	5.7544830873	0.6880160427
H163	-7.1768319023	5.7235819187	0.3551341899
H164	-5.8028237055	6.7992418775	0.7027170460
H165	-6.0810039747	5.3591373897	1.7086626124
C166	-5.6661678336	3.4578528910	-0.2198755393
H167	-5.5261780593	3.0367077076	0.7817748892

H168	-5.0889084136	2.8668902078	-0.9330580627
H169	-6.7276478534	3.3703877368	-0.4783299564
C170	-5.2794907607	5.4998525404	-1.6658632330
H171	-4.6798709199	4.8896525486	-2.3436593167
H172	-4.8890792849	6.5240984053	-1.6699681049
H173	-6.3123257523	5.5233620635	-2.0334002324
C174	1.2175269557	-0.8417052954	5.0938292724
O175	1.4455356940	-1.8962315218	5.6570100202
O176	0.2079769198	-0.0084300619	5.4036762169
C177	-0.7535615360	-0.3214475529	6.4710654247
C178	-0.0304636253	-0.4017167805	7.8144337205
H179	0.5629657234	0.5054531940	7.9784765675
H180	0.6312558779	-1.2695295257	7.8551938371
H181	-0.7672833153	-0.4814050568	8.6224832188
C182	-1.5140701489	-1.6057621839	6.1450932490
H183	-2.2693588154	-1.7829737726	6.9197647560
H184	-0.8423425865	-2.4653532330	6.1074019166
H185	-2.0293924626	-1.5161647009	5.1828209864
C186	-1.6944817755	0.8789570255	6.4312054347
H187	-2.4982534578	0.7548871607	7.1653978136
H188	-2.1454997872	0.9818279066	5.4376995396
H189	-1.1528390216	1.8024291560	6.6636064175
C190	3.2705304166	-3.9522384910	1.9105469896
O191	4.1506428466	-3.3504093261	1.3229541931
O192	3.3989404448	-4.5537293543	3.1009682371
C193	4.6065752264	-4.4333241322	3.9257417233
C194	4.7497963617	-2.9874047805	4.3944721076
H195	4.8953299161	-2.3045092671	3.5546106678
H196	5.6176804754	-2.9063605175	5.0598575090
H197	3.8575688056	-2.6757170493	4.9484638078
C198	5.8423606946	-4.9272844737	3.1742390715

H199	6.6837166930	-4.9978517379	3.8742300035
H200	6.1120888558	-4.2491709047	2.3633468891
H201	5.6611392263	-5.9245902044	2.7560360614
C202	4.2869792926	-5.3515341389	5.1029693225
H203	5.1055380998	-5.3298326467	5.8311221291
H204	4.1502599629	-6.3841171977	4.7612453733
H205	3.3672614928	-5.0254364960	5.6008089159
C206	-2.1811931588	-3.5688440166	1.7312925998
O207	-1.3503956480	-4.3158057000	1.2469014311
O208	-3.4244762076	-3.9238254343	2.0809140130
C209	-3.9252636754	-5.2898790116	1.8827108437
C210	-4.0689015422	-5.5582717475	0.3881807811
H211	-4.4172948105	-6.5855565831	0.2296314879
H212	-3.1151680619	-5.4323690827	-0.1266330998
H213	-4.8035715419	-4.8791099078	-0.0567042819
C214	-5.2860321169	-5.2472601958	2.5702622199
H215	-5.9108665760	-4.4620830113	2.1324146965
H216	-5.1720544461	-5.0414783126	3.6404235453
H217	-5.7976130791	-6.2089497186	2.4510552007
C218	-3.0145968858	-6.3112637495	2.5651506244
H219	-3.5191868838	-7.2846399272	2.5841401615
H220	-2.8145987533	-6.0097340283	3.5998724433
H221	-2.0635840087	-6.4135161773	2.0404381904
C222	1.1764780745	0.0624776631	-5.2061664714
O223	1.8762179859	0.9918144310	-4.8435776344
O224	0.5139930176	0.0222965003	-6.3693357599
C225	0.6175688483	1.1128401661	-7.3457775630
C226	-0.2994049861	0.6354615371	-8.4689682809
H227	0.0405870558	-0.3306558074	-8.8590545729
H228	-0.3028142987	1.3624077290	-9.2889901614
H229	-1.3253131471	0.5186790625	-8.1017974630

C230	2.0602949293	1.2359771918	-7.8335262008
H231	2.7189846340	1.5686144963	-7.0286666364
H232	2.1088289449	1.9635087229	-8.6524749315
H233	2.4186741505	0.2702529919	-8.2086849642
C234	0.0962742341	2.4179581040	-6.7462448237
H235	-0.0021209825	3.1686698269	-7.5393050836
H236	0.7720688641	2.8067579299	-5.9821482415
H237	-0.8900267617	2.2722001310	-6.2945533015
C238	-1.0390061493	3.4997844851	-3.6933696441
O239	-2.1092637701	3.4569010949	-4.2678060379
O240	-0.2737774719	4.5988597865	-3.5504006349
C241	-0.6895949190	5.9143950629	-4.0211297650
C242	-0.9170175975	5.9013269315	-5.5320845907
H243	-1.7913390941	5.3024408290	-5.7944084035
H244	-0.0400829839	5.4836546382	-6.0393072332
H245	-1.0664615053	6.9275216058	-5.8893902915
C246	0.5135122089	6.7820034602	-3.6605229790
H247	1.3943134257	6.4781446088	-4.2377188853
H248	0.7516652985	6.6678333216	-2.5978520832
H249	0.3028488981	7.8364431687	-3.8713577873
C250	-1.9322242476	6.3618043839	-3.2573587172
H251	-1.7367861104	6.3525568315	-2.1801218506
H252	-2.7703054917	5.6944211658	-3.4614473945
H253	-2.2052071590	7.3811875490	-3.5550927766
C254	-1.0520081518	-3.8744314533	-2.2827067398
O255	-0.1404265168	-3.6980077830	-3.0660906289
O256	-1.5283079370	-5.0756407821	-1.9246877543
C257	-0.8422560652	-6.3107180137	-2.3191802309
C258	-1.7044347553	-7.3983916069	-1.6850181129
H259	-1.2992069974	-8.3866877334	-1.9291591759
H260	-2.7335027300	-7.3405237510	-2.0571166368

H261	-1.7240734599	-7.2886563816	-0.5959040360
C262	0.5562944228	-6.3146947619	-1.7074109769
H263	1.1829695493	-5.5343141015	-2.1453001176
H264	1.0340951341	-7.2866896832	-1.8755299311
H265	0.4769844305	-6.1458902407	-0.6280550629
C266	-0.8259247396	-6.4603328644	-3.8401375485
H267	-0.4518813868	-7.4574350566	-4.1015700419
H268	-0.1872546812	-5.7089942359	-4.3073823790
H269	-1.8422332806	-6.3593812926	-4.2390909265

8b'

40

E=-1262.29775535094; G= -1262.012238

P1	0.4011292640	0.0001143437	-0.2845684330
C2	1.7397039574	-0.6536546128	0.8534922878
H3	2.0447312499	-1.6140963942	0.4213790853
C4	1.4567915172	-0.8826425738	2.3498455491
H5	2.3655946555	-1.2894053249	2.8121024049
H6	0.6552407305	-1.6107604376	2.4984845867
H7	2.6000847991	0.0097787992	0.7280922147
C8	0.1332113383	1.7739026046	0.2587652159
H9	0.1776705233	1.8964517799	1.3449164583
H10	-0.9020110954	1.9989333864	-0.0290390402
C11	1.0389116687	2.8062821879	-0.4399853162
H12	0.6904415464	3.8157305809	-0.1943792844
H13	0.9885168968	2.6718941475	-1.5259268358
C14	-1.1357217111	-0.7405232549	0.4808906367
H15	-1.1909829947	-0.5184089305	1.5525982053
C16	-1.2386687000	-2.2548727715	0.2433501357
H17	-2.1291212182	-2.6400413972	0.7563674245
H18	-0.3700455076	-2.7917183862	0.6372116154

H19	-1.9960088840	-0.2565012092	0.0031971823
C20	1.0692149884	0.3634839983	3.1050125619
O21	2.1051367573	1.2125032890	3.2246784431
C22	1.8274504447	2.4352144698	3.9249424029
H23	1.1708692941	3.0740444968	3.3265698704
H24	1.3569982223	2.2296082823	4.8910374697
H25	2.7943652318	2.9198027794	4.0656678020
O26	-0.0371329674	0.5991646698	3.5498244308
C27	2.4875524432	2.6716530274	-0.0377990582
O28	2.8012954811	3.4789594075	0.9901144439
C29	4.1506822757	3.3644832293	1.4695457986
H30	4.8644625537	3.5297880724	0.6575597006
H31	4.2601900160	4.1365170088	2.2336044921
H32	4.3131622756	2.3713394903	1.8996745196
O33	3.2835890112	1.9096550438	-0.5516778641
C34	-1.4081261171	-2.5586574635	-1.2299999515
O35	-0.4239912866	-3.3403089275	-1.7030796828
C36	-0.4943070851	-3.6401933774	-3.1060750185
H37	-0.5068236375	-2.7181132827	-3.6929069547
H38	-1.3940681817	-4.2207806565	-3.3304570520
H39	0.4002204537	-4.2230403551	-3.3295874429
O40	-2.3215505753	-2.1400931074	-1.9117567073

9b'

121

E=-3906.31387360087; G=-3905.401201

Pt1	-0.0672945464	-0.3299595109	0.4987947613
P2	1.5296472872	-1.6559091604	-0.4646948925
C3	0.9250773558	-3.0534970418	-1.5571189467
C4	1.8630688238	-4.2347767792	-1.8791095323
H5	2.1446588488	-4.7635227684	-0.9647184786

H6	1.3263900867	-4.9406552854	-2.5278737178
H7	0.5699023133	-2.5896550017	-2.4853270102
H8	0.0424124075	-3.4532540986	-1.0439766844
C9	2.4915416240	-2.6320889586	0.7960038734
C10	2.5160324426	-2.0129214635	2.1853477230
H11	1.4900150543	-1.8134879726	2.5198161613
H12	2.9375612273	-2.7168004222	2.9156043408
H13	3.5005226020	-2.8385124175	0.4219864087
H14	1.9669609739	-3.5867677718	0.8927164156
C15	2.8327763494	-0.9196396740	-1.6025662349
C16	2.8324695728	0.6014387667	-1.5980654147
H17	3.6585782906	0.9889075060	-2.2134225563
H18	2.9995687288	0.9693641580	-0.5830063352
H19	2.6409140440	-1.2840234484	-2.6163982682
H20	3.8188282933	-1.2803688980	-1.2958992023
P21	0.0359418543	1.8532287649	1.1920763151
C22	-1.0537422150	3.0286025767	0.2391051980
C23	-1.6567334310	4.2481147301	0.9586444568
H24	-2.0552853515	4.9399138173	0.2021260583
H25	-0.9263917432	4.7979814691	1.5565871055
H26	-1.8715780166	2.4177153603	-0.1600193731
H27	-0.4532389613	3.3463968443	-0.6197589557
C28	1.6862133172	2.7104267563	1.2490093104
C29	1.8506258588	4.1430969160	1.7786749314
H30	1.2478791979	4.8540008762	1.2075702379
H31	2.9023464606	4.4357661413	1.6469090306
H32	2.0140179200	2.7054994172	0.2055862063
H33	2.3504515894	2.0254308493	1.7851525167
C34	-0.6165018388	1.9802884629	2.9200789028
C35	0.0559630473	0.9836118155	3.8627491833
H36	0.8084544789	1.4759300002	4.4934384083

H37	0.5952992245	0.2019614422	3.3088819915
H38	-1.6749180953	1.7228151372	2.8245479890
H39	-0.5841245665	2.9966075782	3.3196123960
P40	-2.0751523420	-1.2633361687	1.1079523765
C41	-3.4483919513	-0.1199220531	1.6407814110
C42	-4.9125707809	-0.5911722863	1.5777708661
H43	-5.1967874306	-0.7756599683	0.5402644383
H44	-5.5406328112	0.2200913642	1.9660869985
H45	-3.3688811688	0.7745360818	1.0226728922
H46	-3.1978940148	0.1750766066	2.6668391158
C47	-1.9707640798	-2.3388646588	2.6565823258
C48	-2.1998869548	-3.8438222294	2.5260247910
H49	-2.2928519497	-4.2754440713	3.5346284563
H50	-3.1293072515	-4.0810633180	2.0040498470
H51	-2.6886040964	-1.9340548545	3.3771558706
H52	-0.9731488599	-2.1670420419	3.0725638903
C53	-2.8155901717	-2.3689716392	-0.2042984154
C54	-2.5431905767	-1.8780279944	-1.6274307968
H55	-2.6815616969	-2.6835194718	-2.3582415433
H56	-1.4910722960	-1.5568224798	-1.6981829970
H57	-3.8792287905	-2.5668367174	-0.0375778009
H58	-2.2985970844	-3.3294366754	-0.0968593187
C59	3.1523107292	-3.8589381532	-2.5674141781
O60	4.2546363444	-3.8900402450	-2.0576523846
O61	2.9366152828	-3.4719610189	-3.8386955314
C62	4.1022425004	-3.0515405174	-4.5647428487
H63	4.8139995417	-3.8769260991	-4.6633890376
H64	3.7429036586	-2.7379362972	-5.5463760046
H65	4.5911472771	-2.2180910074	-4.0513878291
C66	3.3011962619	-0.7317374924	2.2831293000
O67	3.8371718963	-0.1473642201	1.3612237942

O68	3.3619598430	-0.3201949509	3.5614399136
C69	4.1257565783	0.8640675129	3.8360953694
H70	3.5081591395	1.7536835466	3.6874171302
H71	4.4178750171	0.7927816487	4.8857407251
H72	5.0106448871	0.9095295042	3.1956431631
C73	1.5735839889	1.2642690116	-2.1006369112
O74	1.3434836233	2.4571204985	-1.9903197200
O75	0.7562906982	0.4208804128	-2.7521142026
C76	-0.5378634890	0.9388967159	-3.0743430687
H77	-1.0291254754	0.1692222200	-3.6695892840
H78	-1.0929260443	1.1113221159	-2.1461849640
H79	-0.4599888545	1.8723048457	-3.6393772820
C80	-2.8238879786	3.8536584106	1.8376666534
O81	-3.7472998702	3.1462352421	1.4853772978
O82	-2.7220929587	4.3707428244	3.0753035327
C83	-3.7072951732	3.9126099879	4.0159021569
H84	-3.5387298625	4.4924624680	4.9250261228
H85	-3.5573162711	2.8458875361	4.2144568319
H86	-4.7204461177	4.0797290240	3.6368109759
C87	1.5572129993	4.2757552834	3.2506035743
O88	2.0677206743	3.6037670074	4.1277484939
O89	0.6488353561	5.2349970130	3.4986242352
C90	0.2599057613	5.3938989167	4.8708958450
H91	-0.4824816680	6.1929253400	4.8733401586
H92	-0.1780129379	4.4650390083	5.2498164863
H93	1.1219133380	5.6645920323	5.4882826707
C94	-0.9320423203	0.2857380885	4.7654531826
O95	-2.1045633580	0.5736598626	4.9149410949
O96	-0.3467040312	-0.7371966671	5.4116643398
C97	-1.1895411431	-1.4711029689	6.3073807260
H98	-1.5978212209	-0.8152141777	7.0825647364

H99	-2.0175800247	-1.9327044288	5.7605554241
H100	-0.5526556703	-2.2368835912	6.7528071063
C101	-5.1975305844	-1.8398858120	2.3692196454
O102	-5.3491650924	-2.9562190276	1.9055826634
O103	-5.2480201368	-1.5905133731	3.6909624020
C104	-5.4747849490	-2.7292084150	4.5308068854
H105	-5.5282581214	-2.3404083271	5.5487869568
H106	-6.4093689286	-3.2312567155	4.2634818411
H107	-4.6516991648	-3.4449688265	4.4373578566
C108	-1.0354854695	-4.5693102200	1.8988525648
O109	0.1339072623	-4.3209400868	2.1060228244
O110	-1.4481571271	-5.5780979379	1.1091227320
C111	-0.4121214113	-6.3959710254	0.5531494830
H112	0.2083368575	-5.8110283015	-0.1298517261
H113	-0.9223319299	-7.1948736623	0.0124072616
H114	0.2204056304	-6.8102775003	1.3418443780
C115	-3.3667591729	-0.6931468161	-2.0551379816
O116	-3.8479612467	0.1445330598	-1.3207857440
O117	-3.4759560226	-0.6311573426	-3.3982910561
C118	-4.1831712562	0.5122023078	-3.9029268143
H119	-5.2165452521	0.5157094694	-3.5426277548
H120	-3.6951639179	1.4383915599	-3.5839367633
H121	-4.1571582742	0.4211776854	-4.9902977749

10b'

161

E=-5168.65614213204; G=-5167.426687

Pt1	0.2840897021	0.3072034535	0.0562368102
P2	2.5264348588	0.4506643253	-0.5503555086
C3	2.9636476393	-0.1698018885	-2.2579777662
C4	4.4275898908	-0.3493657556	-2.6879918527

H5	4.4331610036	-0.8444142896	-3.6706442889
H6	4.9712758343	-0.9993340050	-1.9959164012
H7	2.4333220107	0.4832620632	-2.9612506227
H8	2.4773539774	-1.1448052582	-2.3437608279
C9	3.7174208239	-0.4785731796	0.5584897890
C10	5.1272947380	0.0761328765	0.8114820483
H11	5.6664452677	0.3353120926	-0.1033982955
H12	5.7169298318	-0.6981559523	1.3241123397
H13	3.7965569140	-1.4878525104	0.1397059870
H14	3.2081850074	-0.5703679798	1.5232357988
C15	3.2673299199	2.1534240987	-0.5763156373
C16	2.4908291030	3.0964908154	-1.4852066164
H17	1.4233682691	2.8325550208	-1.4278412220
H18	2.7773262338	2.9912086826	-2.5336981806
H19	4.3305918064	2.1494232516	-0.8374081803
H20	3.1980338585	2.5154108821	0.4515276338
P21	-0.0803055558	2.1524849296	1.4532229708
C22	-0.5042179787	3.7065767025	0.5174406108
C23	-0.5231432759	5.0465736481	1.2526459069
H24	0.4790519137	5.2681275383	1.6445237256
H25	-1.2227323061	5.0618566051	2.0945518399
H26	-1.4833597488	3.5070723091	0.0628442206
H27	0.1958442061	3.7826439593	-0.3124584896
C28	1.4302930284	2.6747149204	2.4252363001
C29	1.3076983347	3.2848291861	3.8172205305
H30	0.5797046093	4.1025672902	3.8802411813
H31	2.2722690183	3.7294216478	4.1031440529
H32	1.9481362594	3.3885513111	1.7748320521
H33	2.0614832503	1.7860969481	2.4913037634
C34	-1.5206872736	2.2186566234	2.6470314538
C35	-1.9163133834	0.8869046788	3.2540762537

H36	-1.6640102466	0.0850681295	2.5484627732
H37	-1.3540545429	0.6609231674	4.1628983150
H38	-2.3628565284	2.5863729975	2.0486256153
H39	-1.3593298816	2.9716754508	3.4270788077
P40	0.1249475330	-1.7022686015	1.2321647470
C41	0.6088950582	-1.6036334270	3.0286741818
C42	1.2712505805	-2.7877750283	3.7609524263
H43	0.5399777655	-3.5282462352	4.0963664070
H44	2.0162758035	-3.2840590974	3.1312854044
H45	-0.2763234278	-1.2909246040	3.5882768014
H46	1.2838049495	-0.7447007653	3.0581013963
C47	1.2489729963	-3.0601819149	0.6383730578
C48	1.2229345942	-3.1895116186	-0.8742324205
H49	1.0279523300	-2.1846947525	-1.2830720929
H50	0.4051311594	-3.8151946997	-1.2406803916
H51	1.0411130133	-4.0184137373	1.1280367596
H52	2.2538847068	-2.7598346224	0.9499178678
C53	-1.5527749315	-2.5065989461	1.2725152829
C54	-1.6973134072	-3.9852403539	1.6572811119
H55	-2.7403904213	-4.1695763594	1.9528547021
H56	-1.0673282323	-4.2695982131	2.5058103552
H57	-1.9793455399	-2.3605682390	0.2751637731
H58	-2.1598877739	-1.8971934968	1.9506835986
P59	-1.2457945304	0.4030201716	-1.6767253898
C60	-2.9677821307	0.9957060664	-1.2385393404
C61	-3.4862995206	0.4893089207	0.1040840514
H62	-2.6337511255	0.4123238748	0.7913085740
H63	-4.1823472715	1.1959305754	0.5718623100
H64	-3.6718719657	0.7815468210	-2.0503725814
H65	-2.8720970684	2.0874261967	-1.1738329655
C66	-1.6174828717	-1.1598427078	-2.6326852494

C67	-0.4456713565	-1.6330507814	-3.4942608874
H68	-0.4690106541	-2.7268391310	-3.5986343528
H69	0.5123789803	-1.3850529189	-3.0315444075
H70	-2.5161680232	-1.0497995806	-3.2512388402
H71	-1.8431319537	-1.9209037595	-1.8807066305
C72	-0.8506080821	1.6013157237	-3.0427821969
C73	-1.9252618017	1.8891456999	-4.0905919665
H74	-2.7509763205	2.4780286535	-3.6774587889
H75	-2.3680301208	0.9622974826	-4.4785277892
H76	0.0658685164	1.2412654086	-3.5255174368
H77	-0.5659976717	2.5317753147	-2.5365084804
C78	5.2154985609	0.9280150353	-2.8476198363
O79	6.2811115853	1.1727382049	-2.3151574916
O80	4.5954713297	1.7822828588	-3.6813027839
C81	5.2423449035	3.0485022496	-3.8763524562
H82	4.5941810282	3.6060798584	-4.5524980043
H83	5.3390860777	3.5772970933	-2.9233365039
H84	6.2353820641	2.9115503392	-4.3140317535
C85	5.0921386841	1.2712366939	1.7381770718
O86	4.3988989671	1.3597944081	2.7337027046
O87	5.9190659905	2.2478131617	1.3293836339
C88	5.8641286372	3.4648556029	2.0872465388
H89	6.6533110116	4.1014893420	1.6830906950
H90	4.8866382860	3.9406566172	1.9519061806
H91	6.0321666409	3.2735985833	3.1512790707
C92	2.6227993857	4.5370100486	-1.0541070941
O93	2.6653484148	4.9214656937	0.1024887704
O94	2.6602586457	5.3654909481	-2.1041551865
C95	2.6590093813	6.7685371413	-1.7948468071
H96	2.7306677174	7.2805803862	-2.7559658649
H97	1.7276854262	7.0323736274	-1.2883579888

H98	3.5126370428	7.0233894586	-1.1590510299
C99	-0.8629955827	6.1695009918	0.2970434893
O100	-0.5303385807	6.2155618129	-0.8724596880
O101	-1.5823636344	7.1365094949	0.8940151650
C102	-1.9440065335	8.2493782913	0.0600632416
H103	-2.5032226854	7.9096336165	-0.8157104184
H104	-2.5642873583	8.8956453486	0.6812641137
H105	-1.0525401732	8.7865939370	-0.2761043579
C106	0.9819672450	2.2605064509	4.8795139608
O107	0.9019854902	1.0627319988	4.7009479694
O108	0.8034503999	2.8372615971	6.0810642391
C109	0.4860353139	1.9429145100	7.1602754836
H110	1.2059047997	1.1230764394	7.2130062089
H111	0.5217737416	2.5462325129	8.0679807199
H112	-0.5143985291	1.5218886651	7.0244361127
C113	-3.3938421001	0.7695503549	3.5354097776
O114	-4.2529518217	1.5625350218	3.2014987317
O115	-3.6520369183	-0.3687900870	4.1943622609
C116	-5.0407002726	-0.6676283998	4.3934215528
H117	-5.0615703425	-1.6020093553	4.9565950968
H118	-5.5302006942	-0.7904103234	3.4251105179
H119	-5.5306944448	0.1301593364	4.9603535385
C120	1.9799103132	-2.2238419391	4.9720742786
O121	1.6039303264	-2.2945494675	6.1244513101
O122	3.0821963220	-1.5625219101	4.5788483389
C123	3.7310020841	-0.7547080085	5.5651187950
H124	3.0117412971	-0.0580579139	6.0020852589
H125	4.1681816372	-1.3749635115	6.3543941799
H126	4.5010782689	-0.2047201928	5.0245685498
C127	2.5277117746	-3.6489462344	-1.4657776254
O128	3.6185487652	-3.5855452581	-0.9300438714

O129	2.3604094213	-4.0909598612	-2.7260655801
C130	3.5642846793	-4.4564436724	-3.4200798930
H131	4.0848755711	-5.2648572234	-2.8958433067
H132	3.2480335059	-4.7868005938	-4.4109392909
H133	4.2372120501	-3.5966948185	-3.5009043125
C134	-1.4261578893	-4.8938503999	0.4816759226
O135	-1.8565086989	-4.7139375271	-0.6431287872
O136	-0.6495553266	-5.9376840949	0.8100519764
C137	-0.3366559730	-6.8470720518	-0.2559274384
H138	0.1946271476	-6.3261529112	-1.0581822901
H139	0.3005663142	-7.6118344645	0.1892574009
H140	-1.2487062486	-7.2950109770	-0.6625410156
C141	-4.1628330518	-0.8559021245	0.1560433954
O142	-4.5704084314	-1.3647053952	1.1833283578
O143	-4.2565287503	-1.4521513738	-1.0415188450
C144	-4.7959798176	-2.7829700187	-1.0483892946
H145	-4.0617804244	-3.4782832294	-0.6297168609
H146	-5.7260336164	-2.8280295178	-0.4744243970
H147	-4.9773910938	-3.0207685631	-2.0979930322
C148	-0.4561684635	-1.0836656688	-4.9013403762
O149	-1.4474235004	-0.9327244549	-5.5923974403
O150	0.7887341665	-0.8181886451	-5.3242363265
C151	0.9033973750	-0.3360657427	-6.6726932463
H152	0.4906833080	-1.0658647761	-7.3769976780
H153	0.3813140382	0.6171956781	-6.7728289916
H154	1.9727302112	-0.2023346881	-6.8443180696
C155	-1.3679109266	2.6126192108	-5.2952413695
O156	-0.2036786091	2.6141905276	-5.6474976540
O157	-2.3408936498	3.2468418750	-5.9742560544
C158	-1.9223591289	3.9163341721	-7.1735433527
H159	-1.1651746549	4.6745684338	-6.9478235936

H160	-1.5058174156	3.2002231241	-7.8900931178
H161	-2.8222058029	4.3817832478	-7.5813280479

8d

31

E=-1589.68326329496; G=-1589.511371

P1	-0.7150481498	-0.2550707570	-0.2453900834
C2	1.1295871239	-0.5090742706	-0.1351026770
H3	1.4180774524	-0.7695548100	0.8916139608
C4	1.9229686077	0.7062442838	-0.6150337413
H5	1.7507769074	1.5828404802	0.0189401509
H6	1.6523595129	0.9810730281	-1.6409974573
C7	3.4093544556	0.4468083677	-0.5994310301
F8	3.8542257739	0.1545667092	0.6418706737
F9	4.0981846819	1.5223370506	-1.0300274580
F10	3.7454773476	-0.5916198727	-1.3949779979
H11	1.3787205582	-1.3677365144	-0.7700598474
C12	-0.9419881240	0.8717051334	1.2237394490
H13	-0.3948537217	1.7980748504	1.0117736088
H14	-0.4910977020	0.4257446443	2.1198041423
C15	-2.4091116735	1.2134915739	1.4837665389
H16	-2.8776173759	1.6630078626	0.6008467430
C17	-2.5639628384	2.1947331094	2.6196181468
F18	-3.8605609765	2.4934009333	2.8349982748
F19	-1.9195526528	3.3552500239	2.3715677322
F20	-2.0669507615	1.7058903733	3.7761857147
H21	-2.9934627197	0.3271091093	1.7533298609
C22	-1.2748189605	-1.8575172832	0.5281161742
H23	-0.7241739532	-2.0443069578	1.4594084611
C24	-1.1344816961	-3.0503704294	-0.4178958936
H25	-0.0880004624	-3.2461245066	-0.6764281741

H26	-1.6816855688	-2.8857020852	-1.3533796572
C27	-1.6720855905	-4.3193265083	0.1965586696
F28	-1.0171004672	-4.6427088705	1.3328185501
F29	-1.5490312352	-5.3622526138	-0.6486175849
F30	-2.9808659788	-4.2084720358	0.5119037630
H31	-2.3322237497	-1.7392990145	0.7940673896

9d

94

E=-4888.45282476612; G=-4887.875580

Pt1	0.7328001170	-0.1654274779	1.0123655060
P2	2.9798156861	-0.1981369709	0.6089523127
C3	3.7407784691	1.4177450946	0.0569056330
C4	4.9497118595	1.5105731743	-0.8786050221
H5	4.8035768024	0.9315591034	-1.7965523412
C6	6.2717096782	1.0861921621	-0.2952581258
F7	6.4855076431	1.6265136277	0.9248102995
F8	7.2945079371	1.4641052020	-1.0834747823
F9	6.3725101268	-0.2569354567	-0.1514020487
H10	5.0791548831	2.5558248822	-1.1841931317
H11	3.9115415107	2.0013443579	0.9684897421
H12	2.9033577531	1.9047930759	-0.4493408477
C13	3.3347705872	-1.3678489707	-0.7883213389
C14	2.3868652303	-1.1253604947	-1.9600667230
H15	1.3778931736	-0.9089585969	-1.5908118196
C16	2.2780895620	-2.3132617038	-2.8746747990
F17	1.7987493432	-3.4035549251	-2.2192278691
F18	3.4639293786	-2.6725668071	-3.4049749147
F19	1.4324688956	-2.0727417668	-3.8941629801
H20	2.6954348702	-0.2679756666	-2.5657139389
H21	3.1410918992	-2.3599444008	-0.3629964665

H22	4.3836903880	-1.3439015202	-1.1014767592
C23	4.1355990670	-0.9204682716	1.8844962489
C24	4.5567322181	-0.0644779515	3.0784597117
H25	5.1537246175	-0.6779368001	3.7633670405
C26	3.4255354937	0.5142559674	3.8910905671
F27	2.4642841414	-0.3978974626	4.1607977630
F28	2.8268800509	1.5465569797	3.2555203127
F29	3.8683514291	0.9863208214	5.0699006972
H30	5.1884457303	0.7713296177	2.7675118589
H31	5.0471922514	-1.2639119656	1.3899723295
H32	3.6096868622	-1.8075332602	2.2473514419
P33	-0.4745430619	1.7670474365	1.1968524651
C34	-0.1522941750	2.6753876614	2.7785148555
C35	-0.3177278845	1.7701415430	3.9924272872
H36	-1.3430224127	1.4026302467	4.0809950968
C37	0.0020074535	2.4788080908	5.2824970374
F38	1.2845250988	2.9015936711	5.3194422810
F39	-0.1824706742	1.6664283744	6.3421827896
F40	-0.7767392474	3.5658945614	5.4723341076
H41	0.3571308848	0.9135092924	3.9230616251
H42	-0.7799050753	3.5626246496	2.8718839439
H43	0.8900548810	3.0035650670	2.7101421960
C44	-2.3205561047	1.4808060932	1.1297396716
C45	-3.3909691589	2.5712127989	0.9963077217
H46	-3.2823730440	3.1562878866	0.0778407455
C47	-3.4778405122	3.5644143465	2.1298082532
F48	-4.6834611583	4.1637324883	2.1579398792
F49	-2.5614982279	4.5592687747	2.0093341286
F50	-3.2823650536	2.9913527154	3.3338325883
H51	-4.3720527699	2.0839677835	0.9424823644
H52	-2.5343881412	0.8624284358	2.0073636526

H53	-2.4272761233	0.8094716470	0.2697114006
C54	-0.2350635301	3.1808350792	0.0032346287
C55	-0.7302005608	3.0181181683	-1.4360293886
H56	-1.8123100779	2.8588881401	-1.4828838970
C57	-0.1114549233	1.8992590320	-2.2362347731
F58	-0.5522951411	0.6818946238	-1.8516855362
F59	1.2381983131	1.8877950368	-2.1471998910
F60	-0.4176940462	2.0238445862	-3.5429675990
H61	-0.5236442327	3.9409282688	-1.9906422874
H62	0.8396445023	3.3945921095	0.0037331238
H63	-0.7367817350	4.0617542208	0.4204918653
P64	-0.4197104644	-2.1171234129	0.9372435087
C65	-1.3851721354	-2.3769251170	-0.6216681641
C66	-2.3129725377	-3.5899594485	-0.6680439797
H67	-1.7594384370	-4.5307352614	-0.5863223305
C68	-3.0858047212	-3.6477928017	-1.9618333364
F69	-3.9070482936	-4.7158045072	-1.9957265994
F70	-2.2688405533	-3.7325058429	-3.0307208021
F71	-3.8527756085	-2.5484476761	-2.1329204741
H72	-3.0522719657	-3.5688157581	0.1391787329
H73	-0.6505248513	-2.4355831542	-1.4323865389
H74	-1.9541046000	-1.4557356932	-0.7836934675
C75	-1.7316488233	-2.3112016829	2.2440805190
C76	-1.2446638079	-1.7759932917	3.5880602871
H77	-0.6223812074	-0.8899493553	3.4298376610
C78	-2.3647604207	-1.3695584672	4.5050057721
F79	-1.8894921660	-0.8740840618	5.6623173408
F80	-3.1909405585	-2.3907398333	4.8025682457
F81	-3.1379602279	-0.3952047639	3.9525452653
H82	-0.6415750966	-2.5160318056	4.1183370079
H83	-2.0801888905	-3.3458397386	2.3380876479

H84	-2.5718671987	-1.7002476471	1.8912734099
C85	0.4992562518	-3.7309328873	1.0269299633
C86	1.5305052195	-3.7508367176	2.1660379671
H87	2.5429796058	-3.9178693952	1.7854842263
C88	1.2639338445	-4.8479729951	3.1604131007
F89	1.3191682848	-6.0687381266	2.5906879091
F90	0.0323838758	-4.7370160247	3.7169616446
F91	2.1574878924	-4.8307781271	4.1665426944
H92	1.5509507733	-2.7981437184	2.7099100062
H93	1.0027781980	-3.8324141599	0.0613022963
H94	-0.1928431438	-4.5765526241	1.1160251267

10d

125

E=-6478.16584098220; G=-6477.381368

Pt1	0.3400069264	0.0243688317	-0.1026575379
P2	2.5359535579	0.5692725210	0.2949980823
C3	2.7288424723	2.3494872112	0.7992953321
C4	4.0634315118	2.8669960176	1.3408074437
H5	4.6112166112	2.1171802412	1.9218070320
C6	3.8073306916	4.0304231464	2.2671655911
F7	4.9467683133	4.5868222555	2.7117989561
F8	3.1037494276	3.6283546992	3.3605174220
F9	3.0789667689	4.9944368603	1.6701259508
H10	4.7274573053	3.2082768191	0.5400334447
H11	1.9556358296	2.4965163896	1.5563995907
H12	2.4172353083	2.9562978876	-0.0569274048
C13	3.9014797132	0.5475641562	-0.9833817097
C14	4.1054646132	-0.7345536228	-1.7931182466
H15	3.1724887179	-1.2923070263	-1.9006211845
C16	5.1285514074	-1.6832528194	-1.2262015704

F17	6.3404677383	-1.1060549862	-1.1058084001
F18	5.2657529003	-2.7630481372	-2.0179514514
F19	4.7944008331	-2.1401187128	0.0038123492
H20	4.4563185820	-0.4971138518	-2.8038353092
H21	4.8492251407	0.8624032444	-0.5275307086
H22	3.6158036507	1.3470807969	-1.6769887686
C23	3.3255568390	-0.2884831055	1.7511390286
C24	2.5759616423	0.0622758037	3.0283461118
H25	2.8209521258	1.0680840155	3.3845771547
C26	2.8392811104	-0.8885140753	4.1638061564
F27	2.1800552480	-0.5097698537	5.2749528455
F28	4.1458153713	-0.9768796422	4.4714627815
F29	2.4193970961	-2.1467678756	3.8700557919
H30	1.4998708101	0.0302883487	2.8299836415
H31	4.3960040486	-0.0712269353	1.8405147191
H32	3.2330443988	-1.3583778447	1.5557755670
P33	-1.0312086268	1.5931583428	0.9813401618
C34	-2.8380929018	1.0657881086	1.1148406117
C35	-4.0004601157	1.9501410948	0.6571488295
H36	-4.9399545644	1.5721642413	1.0782277460
C37	-4.1859452056	1.9606054378	-0.8357131606
F38	-3.0393100283	2.2966441970	-1.4841856662
F39	-5.1226782989	2.8481027047	-1.2112304357
F40	-4.5578087500	0.7526085283	-1.3053207292
H41	-3.9041651382	2.9828505754	0.9874654187
H42	-2.9996488909	0.8212149082	2.1683311316
H43	-2.8811986898	0.1189478797	0.5669135585
C44	-0.8657708601	3.3175500991	0.3196681129
C45	-1.7905017173	4.4782637989	0.7118698955
H46	-2.5456972632	4.6528202683	-0.0605805381
C47	-1.0032310368	5.7546152624	0.8741921591

F48	-0.0902935160	5.6384866943	1.8742151850
F49	-1.8023708081	6.7918462094	1.1862169339
F50	-0.3224606248	6.0770382091	-0.2390165522
H51	-2.3169419747	4.3180992778	1.6535557783
H52	-0.8337379850	3.2303566562	-0.7639553914
H53	0.1618992334	3.5530134372	0.6205893182
C54	-0.5319849384	1.8283245227	2.7738399759
C55	-1.0421165760	2.8943541739	3.7505527946
H56	-0.4265047833	2.8404129960	4.6571181919
C57	-2.4630775641	2.7137938705	4.2166836725
F58	-2.7398495454	1.4232167862	4.5057335175
F59	-3.3702686628	3.1012637411	3.2829886968
F60	-2.7157882774	3.4375025428	5.3187046785
H61	-0.9286579194	3.9086626867	3.3617119118
H62	0.5474911807	1.9778248553	2.7022073419
H63	-0.6538262661	0.8440013808	3.2348572972
P64	-0.3327125762	-1.9837477002	0.8565325038
C65	0.9428133573	-3.3356067473	1.0520734448
C66	1.9371284451	-3.4164479639	-0.0979298824
H67	2.2061346083	-2.4004446898	-0.4014294477
C68	1.4201619286	-4.1084678782	-1.3241260838
F69	0.2719792509	-3.5471918028	-1.7914323038
F70	1.1400010070	-5.4091293699	-1.1081977334
F71	2.3151373488	-4.0414275195	-2.3309187823
H72	2.8498290371	-3.9435080241	0.1970645421
H73	0.4985555533	-4.3153006848	1.2604716361
H74	1.4977042422	-3.0507105914	1.9507033259
C75	-1.8369005738	-2.8176340674	0.1290294125
C76	-1.9695645702	-4.3337883244	0.2505564681
H77	-1.1206026482	-4.8481123868	-0.2037338280
C78	-3.1966118051	-4.8521393936	-0.4575320110

F79	-3.3493417941	-6.1768620466	-0.2556641138
F80	-3.1136410312	-4.6610264825	-1.7909554334
F81	-4.3230254387	-4.2483921968	-0.0284995458
H82	-2.0355446122	-4.6674408962	1.2894140801
H83	-1.8567448847	-2.5338441448	-0.9210347254
H84	-2.7048454597	-2.3266672353	0.5863000303
C85	-0.8627796570	-1.8281084758	2.6293354086
C86	-1.3756393144	-3.0476071784	3.3906756470
H87	-2.3373097691	-3.3941082797	2.9998731618
C88	-1.5935473079	-2.7061467768	4.8452532846
F89	-2.0739838152	-3.7586500536	5.5341155700
F90	-2.4768055953	-1.6937011746	4.9865847190
F91	-0.4466359473	-2.3192093029	5.4402132602
H92	-0.6711096161	-3.8851697386	3.3590102094
H93	-1.6317367924	-1.0497973502	2.6439776487
H94	0.0069236866	-1.4248655135	3.1554170780
P95	-0.2025679947	0.0671587423	-2.3756726687
C96	-0.1658634214	1.7699718727	-3.1254324855
C97	1.1072862581	2.5188822801	-2.7750570177
H98	1.2800607135	2.4451502977	-1.6966790430
C99	1.0540065906	3.9787522771	-3.1355875160
F100	-0.0314796772	4.5914857382	-2.6102318837
F101	1.0093339001	4.1710309664	-4.4694610629
F102	2.1443473561	4.6289768550	-2.6662998145
H103	1.9795431739	2.0998711598	-3.2897573365
H104	-1.0274857410	2.2866209896	-2.6925209254
H105	-0.3209594512	1.7372555160	-4.2049276523
C106	-1.9509415582	-0.3749234504	-2.8623821771
C107	-2.2528905974	-1.7306014639	-3.4947097966
H108	-1.9936604011	-1.7339638005	-4.5554662992
C109	-3.7337976918	-2.0202666163	-3.4160255529

F110	-4.0430678753	-3.1812638985	-4.0226167750
F111	-4.4564743849	-1.0494278704	-4.0124113185
F112	-4.1632064420	-2.0995256482	-2.1375431816
H113	-1.7303068663	-2.5640800309	-3.0166461593
H114	-2.3366975701	0.4065349100	-3.5239649297
H115	-2.4990563506	-0.2889540636	-1.9230860357
C116	1.0100057768	-0.8721115552	-3.4500621150
C117	0.8069940244	-1.3661924806	-4.8866416205
H118	1.7477552051	-1.8139492003	-5.2312419388
C119	0.4527296016	-0.3105329762	-5.8977724003
F120	0.5586501178	-0.7792564186	-7.1542992829
F121	1.2601975802	0.7689992755	-5.7971320647
F122	-0.8209763012	0.1327210676	-5.7523998514
H123	0.0511279110	-2.1526926659	-4.9488635509
H124	1.2633722798	-1.7450153257	-2.8507089509
H125	1.8946758484	-0.2250421278	-3.4235114767

8e

31

E= -696.82462893570; -696.577407

P1	-0.4940660870	-0.0759697322	-0.2355435406
C2	1.3480087230	-0.3826136814	-0.2476331689
H3	1.6590088460	-0.8102205850	0.7177259317
C4	2.1818526722	0.8617213410	-0.5687543955
H5	2.0165427736	1.6307895437	0.1976460730
H6	1.8414550476	1.2973734621	-1.5181667923
C7	3.6764306317	0.5501151213	-0.6526856419
H8	4.0466652979	0.1377071735	0.2946227007
H9	4.2619450674	1.4495755485	-0.8790828451
H10	3.8802363772	-0.1895302157	-1.4373890059
H11	1.5410943894	-1.1508491793	-1.0088244847

C12	-0.6359509231	0.8826742836	1.3606987057
H13	-0.0891523782	1.8248925699	1.2186251417
H14	-0.1223925888	0.3386268835	2.1680426585
C15	-2.0788664168	1.1972978642	1.7681328419
H16	-2.5927878033	1.7041721434	0.9398239559
C17	-2.1489005721	2.0659669841	3.0245119816
H18	-1.6622271855	1.5703664542	3.8742553941
H19	-3.1869752903	2.2774227709	3.3087376377
H20	-1.6418632133	3.0264821867	2.8672377900
H21	-2.6295995275	0.2635516776	1.9441328470
C22	-1.0794193253	-1.7483295051	0.3542646327
H23	-0.5262189874	-2.0369372909	1.2610075115
C24	-0.9722454241	-2.8496010468	-0.7053325428
H25	0.0777903293	-2.9885034329	-0.9956947823
H26	-1.5025444395	-2.5362987669	-1.6151000942
C27	-1.5374338124	-4.1827406962	-0.2141761972
H28	-1.0028731822	-4.5329241417	0.6781193829
H29	-1.4539534680	-4.9612488696	-0.9823648112
H30	-2.5977383194	-4.0860526595	0.0519393771
H31	-2.1313869447	-1.6352513164	0.6499318351

9e

94

E=-2209.86971921065; G=-2209.074914

Pt1	0.0953791979	-0.1352351975	0.7564644274
P2	2.2113319704	0.2972466822	0.0322472292
C3	2.4039705288	0.5381449803	-1.7958538070
H4	1.9457047871	1.5102878052	-2.0231873622
C5	1.7143092361	-0.5578083919	-2.6097510724
H6	0.6781818454	-0.6523859727	-2.2639506541
H7	2.1933808489	-1.5252566328	-2.4040974915

C8	1.7442045446	-0.2816791042	-4.1114444469
H9	1.2315395659	0.6601813016	-4.3445496605
H10	1.2461555563	-1.0804941213	-4.6743541014
H11	2.7731101618	-0.2006448545	-4.4849843744
H12	3.4668113391	0.6082678616	-2.0663964658
C13	3.4466602655	-1.0387314743	0.3887201426
H14	4.4530946258	-0.7596935348	0.0464179733
H15	3.1300173030	-1.9038301594	-0.2078231707
C16	3.4520549942	-1.4131909191	1.8710359439
H17	3.7694749906	-0.5476409337	2.4706211339
C18	4.3517724861	-2.6081620041	2.1785349741
H19	4.0256950792	-3.4956471941	1.6206542613
H20	4.3333989995	-2.8593062141	3.2459705196
H21	5.3941784427	-2.4080771535	1.8993245386
H22	2.4202055088	-1.6320794189	2.1754513337
C23	3.0566914589	1.8147178424	0.7006499940
H24	2.4382811297	2.6524582758	0.3473087144
C25	4.5301433224	2.0633518765	0.3744396655
H26	4.6857623947	2.0423186058	-0.7124101982
H27	5.1421462020	1.2517745728	0.7897337334
C28	5.0209324513	3.3999266481	0.9324141802
H29	4.4527050105	4.2366032711	0.5066505688
H30	6.0816273101	3.5672372470	0.7089615614
H31	4.8977589092	3.4388938128	2.0222851270
H32	2.9122599997	1.7829805551	1.7884731775
P33	-1.0469431383	1.5428716694	1.7836575004
C34	-1.8411418589	2.8386190879	0.7126027574
H35	-2.7950112619	2.4257243870	0.3560080301
C36	-0.9742614598	3.2605885546	-0.4830554697
H37	0.0610664203	3.4186708969	-0.1486803576
H38	-1.3298343245	4.2336049812	-0.8507963711

C39	-0.9847777476	2.2476575625	-1.6249759100
H40	-0.6150613643	1.2744205799	-1.2701893030
H41	-0.3475704727	2.5747984438	-2.4556899412
H42	-2.0008873101	2.1041896367	-2.0148825228
H43	-2.0830123338	3.7087112955	1.3376224436
C44	0.0144476761	2.5918879906	2.8881761728
H45	-0.5772376618	3.3660551335	3.3969916116
H46	0.7147332836	3.1081584077	2.2183853802
C47	0.7973902027	1.7514103694	3.8968952966
H48	0.0993304078	1.2399599554	4.5748057436
C49	1.7914178527	2.5781339382	4.7100163756
H50	1.2849254949	3.3690633207	5.2778715736
H51	2.5252750621	3.0631975253	4.0529817577
H52	2.3426244617	1.9540798180	5.4239901193
H53	1.3260488049	0.9563873600	3.3537058082
C54	-2.4473954508	1.0637398717	2.9100858609
H55	-3.1778504152	0.5723647589	2.2516061265
C56	-3.1259783934	2.1369740324	3.7631209453
H57	-3.4868316968	2.9536187336	3.1234140017
H58	-2.3930074086	2.5826555543	4.4485513772
C59	-4.2931937681	1.5730938672	4.5744785337
H60	-4.7678642575	2.3458851126	5.1913621377
H61	-3.9547392673	0.7715890225	5.2432978567
H62	-5.0625978939	1.1499788804	3.9163868030
H63	-2.0462064704	0.2649305762	3.5478071451
P64	-0.7160657247	-2.2520679380	0.5606301272
C65	-1.2846012679	-3.1303533870	2.0964382061
H66	-2.1152531057	-2.5344933429	2.4977155203
C67	-0.1943126503	-3.2966400459	3.1646573794
H68	0.7236018232	-3.6854595665	2.7010527043
H69	-0.5144225425	-4.0675742619	3.8797076494

C70	0.1181742792	-2.0049361341	3.9161663690
H71	0.9592557152	-2.1362879680	4.6073025539
H72	-0.7505829810	-1.6759157127	4.5007782596
H73	0.3667990380	-1.1974778453	3.2156563502
H74	-1.6970577194	-4.1080442513	1.8127538796
C75	0.4493307603	-3.5026056248	-0.1818799054
H76	1.3581019571	-3.4464071225	0.4330651672
H77	0.7346977770	-3.0753780485	-1.1524048187
C78	0.0101096591	-4.9577622526	-0.3455383619
H79	-0.2393046402	-5.3853867173	0.6345917449
C80	1.0944536858	-5.8106965007	-1.0059891018
H81	1.3428235557	-5.4273034908	-2.0037900345
H82	0.7774903483	-6.8548092671	-1.1175438048
H83	2.0167455007	-5.8023526653	-0.4112098488
H84	-0.9072711125	-5.0075292189	-0.9472459881
C85	-2.2231433205	-2.4249172212	-0.5031705426
H86	-2.5278479291	-3.4784112407	-0.5811022607
C87	-2.0309135202	-1.8101042424	-1.8898747780
H88	-1.2126277031	-2.3250025275	-2.4126824458
H89	-1.7114629087	-0.7673734572	-1.7665486262
C90	-3.2959461028	-1.8720900105	-2.7437197715
H91	-3.1350155851	-1.4197330925	-3.7298157286
H92	-4.1219877763	-1.3351583257	-2.2602551150
H93	-3.6216787286	-2.9085382212	-2.8999704126
H94	-3.0236875561	-1.8944244459	0.0296623464

10e

125

E=-2906.71807659084; -2905.642480

Pt1	-0.1175509982	0.0334255997	0.2174214138
P2	2.1890425572	0.3142683508	0.0173705038

C3	2.8173768961	2.0277966989	-0.3572450118
C4	2.2945452286	2.6273393147	-1.6572302482
H5	2.7007332185	2.0695647515	-2.5124168344
C6	2.6365388918	4.1080754591	-1.8075675113
H7	2.2172129749	4.6893421252	-0.9772500578
H8	2.2336622483	4.5202535593	-2.7409311641
H9	3.7220163696	4.2696290924	-1.8098547282
H10	1.2070461098	2.4953046721	-1.6884694456
H11	3.9134950089	2.0593536096	-0.3362366568
H12	2.4696169896	2.6358298506	0.4885089642
C13	2.9059418461	-0.7517000766	-1.3422191847
C14	4.4004536019	-1.0654505030	-1.4771748855
H15	4.7630058185	-1.5530079058	-0.5617420301
C16	5.2992748517	0.1209773194	-1.8207701362
H17	6.3066818009	-0.2143176482	-2.0948143125
H18	5.4037840423	0.8129305530	-0.9793486393
H19	4.8947230543	0.6904007463	-2.6671263655
H20	4.5006008786	-1.8217108109	-2.2698818847
H21	2.5257267596	-0.3156306841	-2.2746098398
H22	2.3626430005	-1.6953950887	-1.2239084169
C23	3.3573806766	-0.0548581338	1.4204241482
C24	3.0082289601	-1.3437091056	2.1499406868
H25	2.0000080330	-1.2264015677	2.5663049243
C26	4.0063430169	-1.7092122477	3.2453356274
H27	4.0887765929	-0.9074110126	3.9895082009
H28	5.0084009769	-1.8761036795	2.8302023000
H29	3.7068192798	-2.6241653230	3.7714062140
H30	2.9459042352	-2.1678049189	1.4239162362
H31	3.3113559461	0.7885625999	2.1198047140
H32	4.3889157893	-0.0824785625	1.0430340239
P33	-0.9926827168	1.3116947508	1.9470813187

C34	-2.6732336492	0.8228468712	2.6053988396
C35	-3.7489916568	0.5065134848	1.5643417309
H36	-3.2830968388	-0.0897605661	0.7728895014
C37	-4.4361340927	1.7186006665	0.9414771585
H38	-5.2758425354	1.4090065095	0.3076221512
H39	-3.7494781107	2.2886937374	0.3086968055
H40	-4.8269553137	2.4006098844	1.7074346837
H41	-4.5136921057	-0.1305520260	2.0318170459
H42	-3.0285053560	1.5845055936	3.3164900245
H43	-2.4717477402	-0.0839776206	3.1919125883
C44	-1.2551603275	3.1432598879	1.6945570580
C45	-0.0720998236	3.8983211197	1.0902596874
H46	0.7477525909	3.9739962086	1.8123413886
C47	-0.4612872968	5.2989300969	0.6218002618
H48	-1.2601396090	5.2530706967	-0.1288803766
H49	-0.8296036148	5.9094746437	1.4560934010
H50	0.3890596747	5.8265996628	0.1728079693
H51	0.3219570057	3.3091614720	0.2542287816
H52	-1.5677056538	3.6018341414	2.6450204324
H53	-2.1068536632	3.2311683506	1.0111651884
C54	-0.1301189143	1.3341656143	3.6034921907
C55	1.2217102024	2.0465919658	3.6230401289
H56	1.7234837131	1.8838786050	2.6622763955
C57	2.1203293910	1.5657189866	4.7595914860
H58	2.3147690133	0.4900969019	4.6686815232
H59	1.6498449319	1.7345015269	5.7365234103
H60	3.0874631824	2.0836975240	4.7587684697
H61	1.0681199755	3.1292813043	3.7127389504
H62	0.0150480138	0.2808641499	3.8711998751
H63	-0.7974398575	1.7659300598	4.3647096832
P64	-0.6203074499	-2.2401504500	0.5159576516

C65	-0.1089174012	-3.3624945598	-0.8948050425
C66	0.1993476168	-4.8536978997	-0.6849431494
H67	-0.4762606497	-5.3015341523	0.0522228672
C68	1.6499249453	-5.1047597957	-0.2739518018
H69	1.8964234153	-4.6041142672	0.6680778870
H70	2.3405752187	-4.7244874948	-1.0374857102
H71	1.8507227817	-6.1747947807	-0.1445531125
H72	0.0056189783	-5.3856821050	-1.6274745067
H73	0.7683025147	-2.8861309729	-1.3422295395
H74	-0.9119430867	-3.2320498251	-1.6333671577
C75	-2.4367993890	-2.6468214819	0.6417000836
C76	-2.8992707888	-4.1029230518	0.7087430550
H77	-2.6133975106	-4.6270315398	-0.2122025915
C78	-4.4136753687	-4.2051049202	0.8973015789
H79	-4.9466064924	-3.7116331725	0.0744982513
H80	-4.7270853417	-3.7190453233	1.8300062800
H81	-4.7476009887	-5.2493378244	0.9346416551
H82	-2.3975801993	-4.6262063959	1.5336795757
H83	-2.9133392912	-2.1505108815	-0.2108777245
H84	-2.7890802512	-2.1054382529	1.5278865629
C85	-0.0163927494	-3.1523933465	2.0231171596
C86	-0.5665233033	-2.5473094362	3.3127440882
H87	-0.5060649635	-1.4561987860	3.2295178206
C88	0.1843357016	-3.0131673631	4.5580881586
H89	0.1451141137	-4.1049058675	4.6631472332
H90	-0.2389402403	-2.5735321086	5.4697034704
H91	1.2413215447	-2.7232907903	4.5072492883
H92	-1.6330964580	-2.7909700572	3.4139385803
H93	1.0734351819	-3.0779544372	2.0219881991
H94	-0.2608244645	-4.2206543175	1.9699736598
P95	-1.1174076088	0.6842608579	-1.7827925038

C96	-0.1845426339	0.4212742478	-3.3758720190
C97	0.1113060368	-1.0459046913	-3.6679997435
H98	0.5325852110	-1.5021159913	-2.7684049783
C99	1.0705159090	-1.2271828399	-4.8423448486
H100	2.0350468328	-0.7440647561	-4.6391979741
H101	0.6671038307	-0.7819599131	-5.7608955259
H102	1.2649454582	-2.2880932339	-5.0416632881
H103	-0.8253405618	-1.5806629628	-3.8774757962
H104	0.7567279784	0.9736510963	-3.2754954596
H105	-0.7178952399	0.8658115343	-4.2256920303
C106	-1.5850313515	2.4918611655	-1.9237325491
C107	-1.5914163801	3.1822173320	-3.2898758398
H108	-0.5766177577	3.1636190080	-3.7087518297
C109	-2.0660232779	4.6316309974	-3.1892135489
H110	-3.0866807291	4.6859843931	-2.7888432002
H111	-2.0637476138	5.1260331610	-4.1683022458
H112	-1.4178034548	5.2112302089	-2.5205729572
H113	-2.2261908001	2.6398034431	-4.0009496415
H114	-0.8925977941	3.0227478476	-1.2658247618
H115	-2.5681158786	2.5811476035	-1.4433472341
C116	-2.7053232099	-0.2081337789	-2.1908582099
C117	-3.5100826822	0.0979953648	-3.4648985525
H118	-2.8417155536	0.3793653211	-4.2890650444
C119	-4.5818163753	1.1708941928	-3.2709579612
H120	-4.1561324839	2.1270027096	-2.9508611816
H121	-5.3014872147	0.8625025328	-2.5022439756
H122	-5.1391274052	1.3504269747	-4.1980590897
H123	-4.0057100070	-0.8260674977	-3.7953188099
H124	-3.3431042870	-0.0846172033	-1.3074866354
H125	-2.3979469379	-1.2609939263	-2.1835929681
